# [1,2,4]triazolo[4,3-*a*]quinoxaline as Novel Scaffold in the Imiqualines Family: Candidates with Cytotoxic Activities on Melanoma Cell Lines

**DOI:** 10.3390/molecules28145478

**Published:** 2023-07-18

**Authors:** Cindy Patinote, Sandy Raevens, Amélie Baumann, Eloise Pellegrin, Pierre-Antoine Bonnet, Carine Deleuze-Masquéfa

**Affiliations:** Institut des Biomolécules Max Mousseron (IBMM), UMR 5247 F16, (CNRS, ENSCM, Université de Montpellier), 1919 Route de Mende, 34090 Montpellier, France; cindy.patinote@umontpellier.fr (C.P.); sandy_raevens@etu.u-bourgogne.fr (S.R.); baumannamelie22@gmail.com (A.B.); eloise.pellegrin@gmail.com (E.P.); carine.masquefa@umontpellier.fr (C.D.-M.)

**Keywords:** [1,2,4]triazolo[4,3-*a*]quinoxaline, pharmacophore, Imiqualines, EAPB02303, A375 melanoma cell line, cytotoxic activities, anticancer agents

## Abstract

Cutaneous melanoma is one of the most aggressive human cancers and is the deadliest form of skin cancer, essentially due to metastases. Novel therapies are always required, since cutaneous melanoma develop resistance to oncogenic pathway inhibition treatment. The Imiqualine family is composed of heterocycles diversely substituted around imidazo[1,2-*a*]quinoxaline, imidazo[1,2-*a*]pyrazine, imidazo[1,5-*a*]quinoxaline, and pyrazolo[1,5-*a*]quinoxaline scaffolds, which display interesting activities on a panel of cancer cell lines, especially melanoma cell lines. We have designed and prepared novel compounds based on the [1,2,4]triazolo[4,3-*a*]quinoxaline scaffold through a common synthetic route, using 1-chloro-2-hydrazinoquinoxaline and an appropriate aldehyde. Cyclization is ensured by an oxidation-reduction mechanism using chloranil. The substituents on positions 1 and 8 were chosen based on previous structure–activity relationship (SAR) studies conducted within our heterocyclic Imiqualine family. Physicochemical parameters of all compounds have also been predicted. A375 melanoma cell line viability has been evaluated for 16 compounds. Among them, three novel [1,2,4]triazolo[4,3-*a*]quinoxalines display cytotoxic activities. Compounds **16a** and **16b** demonstrate relative activities in the micromolar range (respectively, 3158 nM and 3527 nM). Compound **17a** shows the best EC_50_ of the novel series (365 nM), even if EAPB02303 remains the lead of the entire Imiqualine family (3 nM).

## 1. Introduction

Skin cancers correspond to the uncontrolled proliferation of abnormal melanocytes leading to tumor genesis. They are classified according to their precursor cell type, histopathologic pattern, and clinical significance into two major categories: non-melanoma skin cancers, including squamous cell carcinoma and basal cell carcinoma, and melanoma skin cancers. This latter group, also called cutaneous melanoma, is the deadliest form of skin cancers [1].

Primary cutaneous melanoma arises into the epidermis, before invading the dermis, giving to this early development stage the name of the radial growth phase. This is due to oncogenic driver mutations that lead to activation of the MAPK (mitogen-activated protein kinases) pathway, with the main mutations of BRAF (B-rapidly accelerated fibrosarcoma) (40–50% of cases), NRAS (neuroblastoma RAS viral oncogene) (20–30% of cases), or NF1 (neurofibromatosis-1) (10–15% of cases) [2]. During the vertical growth phase, melanoma cells penetrate the dermis and are consequently able to invade other tissues and metastasize to distant organs. Its aggressiveness is mainly due to a plasticity of tumor cells creating a significant intra-tumoral heterogeneity associated with resistance to treatment and potential for dissemination [3]. Cutaneous melanoma is a multifactorial disease that depends on both environmental (sun exposition, world location, ultraviolet irradiation, etc.) and human body parameters (genetic factors, skin phototype, pigmented nevi, immunosuppressive conditions, age, etc.) [4].

The management of melanoma depends greatly on the location, depth, and stage [5]. Though early stages can achieve remarkable outcomes with surgery alone, metastatic melanoma requires therapeutic treatment intervention, such as chemotherapeutic agents, targeted therapies (vemurafenib [6] and encorafenib [7,8] for BRAF mutation, dabrafenib [9], trametinib [10], and cobimetinib [11] as MEK (mitogen-activated protein kinase kinase) inhibitors [12]), or immunotherapies (prembrolizumab, nivolumab as PD-1 (programmed cell death protein-1) inhibitors [13], ipilimumab as CTLA-4 (Cytotoxic T lymphocyte antigen-4 protein) inhibitors [14], cytokines (Interferon-α, Interleukin-2) [15]).

Even though it is largely preventable, the prognosis of cutaneous melanoma is poor in advanced and metastatic stages. In 2020, the number of new worldwide cases of malignant melanoma was estimated at 325,000 people of all ages and sexes, with 57,000 deaths [16]. The highest incidence was observed in Australia and New Zealand (73 per 100,000 person-years), followed by Northern Europe (35 per 100,000 person-years), North America (32 per 100,000 person-years), and Western Europe (19 per 100,000 person-years). Projections for 2040 suggest that melanoma burden could rise to 510,000 novel cases with 96,000 deaths [17]. Although a steady improvement in survival among patients has been reported over the last decades mainly thanks to early detection, therapy improvement, and diagnosis, the mortality rates from melanoma continue to rise due to an increasing incidence [18,19,20]. The need for novel therapeutic compounds is still relevant, since this public health burden will remain strong in the coming decades [17,21].

**Figure 1:**
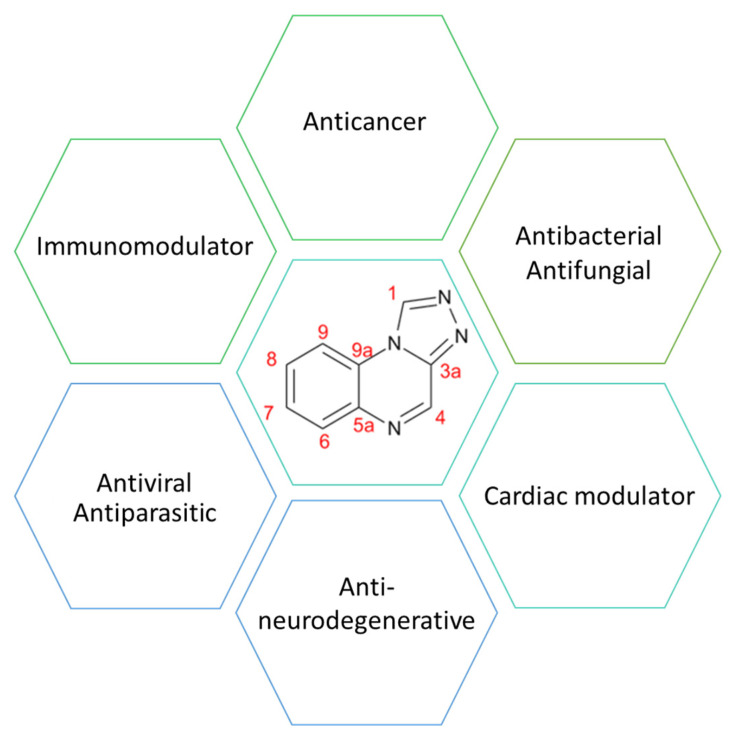
Structure, numbering, and biological activities of the [1,2,4]triazolo[4,3-*a*]quinoxaline scaffold.

The [1,2,4]triazolo[4,3-*a*]quinoxaline scaffold (Figure 1) is made up of fused triazole and quinoxaline, which are privileged fragments possessing diverse biological activities. Researchers have demonstrated the high potential of this tri-heterocycle for a wide spectrum of pharmacological properties [22], such as anticancer [23,24], antibacterial [25,26,27,28,29], antiviral [30,31], antifungal [32,33,34], antiparasitic [35], cardiac modulator [36,37,38], anti-neurodegenerative [39], or immunomodulator agents [40]. Among the recent [1,2,4]triazolo[4,3-*a*]quinoxalines displaying anticancer activities, Ezzat et al. designed and synthetized potent A2B adenosine receptor antagonists with hydrophobic aryl tails linked in the C4 position through a hydrophilic group, which afford low-micromolar-range cytotoxic activities on the human triple-negative breast adenocarcinoma MDA-MB-231 cell line [41]. El-Adl et al. prepared [1,2,4]triazolo[4,3-*a*]quinoxalines with one or two groove-binding side chains and evaluated their anti-proliferative activities against hepatocellular carcinoma HepG2, human colorectal carcinoma-116 (HCT116), and breast cancer MCF-7 (Michigan Cancer Foundation-7) cells and evaluated the most active derivatives as potent DNA intercalators [42]. Non-C1 methylated derivatives were also synthetized, and the more potent compounds were tested as topoisomerase II inhibitors [43]. They also recently highlighted chalcones and their pyrazole derivatives as potent MCF-7 antiproliferative agents with DNA-binding and topoisomerase II inhibition activities [44]. Via a hybrid pharmacophore approach, Kaneko et al. presented C1- and C4-substituted-[1,2,4]triazolo[4,3-*a*]quinoxaline based on Imiquimod and EAPB0203 [45,46,47]. Some of these exhibited low-micromolar antiproliferative effects on human acute myelocytic leukemia HL60, hepatocellular carcinoma HepG2, melanoma B16, and histiocytic lymphoma U937 cancer cell lines [48]. An extensive structure–activity relationship study allowed Alanazi et al. to obtain a novel bis([1,2,4]triazolo)[4,3-*a*:3′,4′-c]quinoxaline as a vascular endothelial growth factor receptor-2 (VEGFR-2) inhibitor and apoptosis inducer by arresting the cell cycle at the G2/M phase. The derivative upregulated the pro-apoptotic Bcl-2-associated X protein (BAX) and caspase-3 and -9 and downregulated the pro-oncogenic cell survival Bcl-2 protein [49]. *N*-Phenyl-[1,2,4]triazolo[4,3-*a*]quinoxaline-1 sulfonamide derivatives have been studied on MCF-7, cervical carcinoma HeLa, lung cancer cell line A549, and neuroblastoma IMR32, and one of these showed higher activity than the standard Etoposide [50].

**Figure 2:**
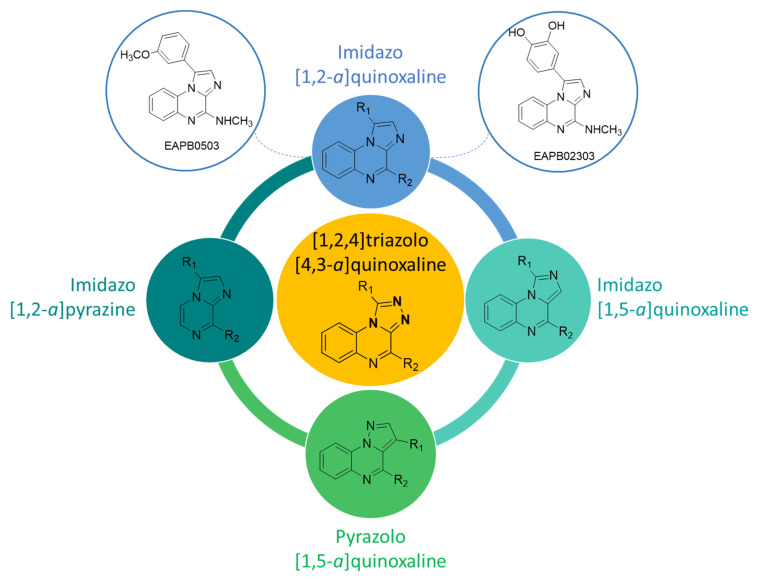
Imiqualines family.

The Imiqualines family designed by our team is composed of heterocycles diversely substituted around imidazo[1,2-*a*]quinoxaline, imidazo[1,2-*a*]pyrazine, imidazo[1,5-*a*]quinoxaline, and pyrazolo[1,5-*a*]quinoxaline scaffolds (Figure 2) [51,52,53,54,55,56]. First-generation hits belong to the imidazo[1,2-*a*]quinoxaline subfamily, such as EAPB0503 (Figure 2) [57,58,59,60,61]. Among our first-in-class compounds of the second generation, the identified lead EAPB02303 (Figure 2) displays outstanding nanomolar activities comparable to or better than those of the current best anticancer agents on a panel of human cancer cell lines, notably on melanoma cell lines [51,53]. Concerning the in vitro safety, EAPB02303 displays no toxicity on human peripheral blood mononuclear cells (hPBMCs, IC_50_ > 200 µM) cells or the human ether-à-go-go-related gene (hERG) cardiac channel. Mechanistic study using transcriptomic, proteomic, and phosphoproteomic analyses confirms an original mechanism of action of EAPB02303 that is different from a panel of well-known anti-cancer agents (tubulin or topoisomerase inhibitors, alkylating or anti-metabolic agents, etc.). Flow cytometry and immunofluorescence reveal cellular cycle arrest in mitosis and DNA accumulation. Mitotic catastrophe occurred after 24 h treatment, and apoptosis after 48 h treatment. Chemical biology tools, such as affinity chromatography using the stable isotope-labeling by amino acids in cell culture (SILAC) method, allowed the identification of protein targets in the A375 melanoma cell line. The lead profile also displays an interesting complementary immunomodulatory activity that is sought after in oncopharmacology. To assess EAPB02303 efficacy on in vivo models, the maximum tolerated dose of single and repeated intra-peritoneal administrations and pharmacokinetic parameters have been determined. Experiments on A375-human-melanoma-xenografted mice confirmed the potent anti-cancer effect of the lead. Like most of the aromatic drugs, EAPB02303 effectiveness is largely limited by its lack of solubility in conventional aqueous buffers.

We recently enlarged our Imiqualine family by designing novel compounds around the [1,2,4]triazolo[4,3-*a*]quinoxaline structure. The [1,2,4]triazolo[4,3-*a*]quinoxaline scaffold is made up of a five-membered heterocycle containing three nitrogen atoms, that is to say, one more nitrogen atom compared to the lead imidazo[1,2-*a*]quinoxaline series. The replacement of a carbon atom by a nitrogen atom could modify interactions in the active site by modulating the hydrophilic/hydrophobic global nature of compounds, and/or aromatic staking on the other hand. The main objective of the work presented herein was not only the design and synthesis of novel [1,2,4]triazolo[4,3-*a*]quinoxalines displaying the essential structural features of our reported hits, lead EAPB02303, and known drugs, but also the biological evaluation on responsive A375 melanoma cell lines. Moreover, in the process of drug development, main candidates should possess appropriate physical and chemical properties to have a better chance of successful development. The early determination of these inherent physicochemical characteristics is important for finding valuable and drugable potential candidates. Thus, additional parameters have also been predicted using ACD Labs^®^ software (version 12.01), such as aqueous solubility (at pH 7, and also logS), lipophilicity (polar surface area, partition coefficient (logP), distribution coefficient (logD)), and the ionization constant (pKa). These physicochemical properties could help to evaluate and predict the likely behaviors of our prepared compounds for future in vitro and in vivo studies.

## 2. Results and Discussion

### 2.1. Background

**Scheme 1:**
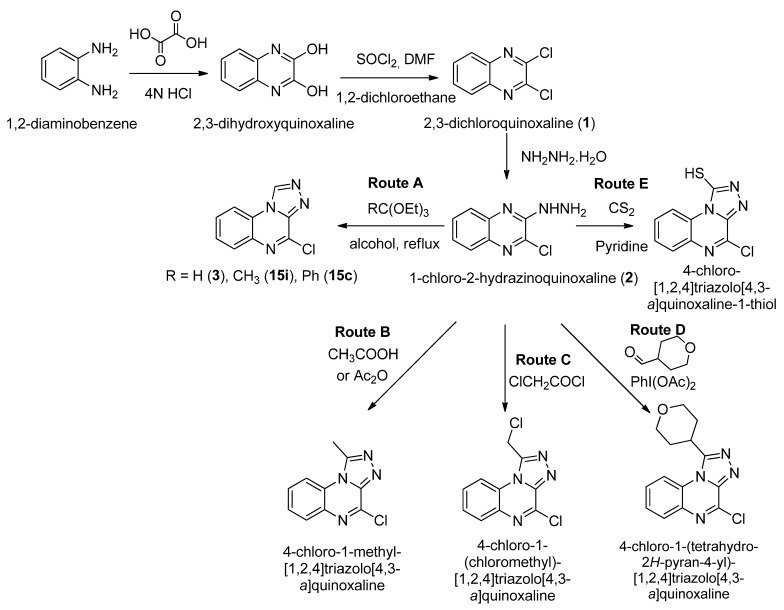
Classical synthetic routes to obtain [1,2,4]triazolo[4,3-*a*]quinoxalines.

To our knowledge, there is no published review on general synthetic pathways of the [1,2,4]triazolo[4,3-*a*]quinoxaline scaffold, but numerous approaches have been reported to prepare specific compounds (Scheme 1).

Even if the intermediate 2,3-dichloroquinoxaline is commercially available, it is also possible to prepare this intermediate by the chlorination of 2,3-dihydroxyquinoxaline carried out by thionyl chloride, which is obtained from 1,2-diaminobenzene condensed with oxalic acid in a 4N hydrochloric acid solution [41,42,44]. Some strategies have been described to build promising tri-heterocyclic compounds from 2-hydrazinoquinoxalines obtained by substituting one of the two chlorines of 2,3-dichloroquinoxaline by hydrazine hydrate. Reactions with carbonyl derivatives leading to 1-alkyl or 1-aryl[1,2,4]triazolo[4,3-*a*]quinoxalines are the most described thanks to the electrophilic character of the carbon. These strategies are interesting because they make it possible to introduce new alkyl patterns into the core structure. Triethyl orthoformate, -acetate, or -benzoate could be used to achieve cyclization of the triazole moiety (Route A, Scheme 1) [37,62,63,64,65,66]. Nevertheless, the limited diversity of this kind of reagents does not allow for the preparation of a heterocyclic compounds bank diversely substituted in position 1 as we envisaged. Carboxylic acids have been used to form the tri-heterocycle but this strategy is relatively less described (Route B, Scheme 1) [48,67]. According to the research of Ezzat et al., the triazoloquinoxaline cycle could be obtained thanks to the use of acetic anhydride in order to introduce a methyl group in position 1 [41,68]. More reactive species, such as chloride acids, also make it possible to constitute the triazole conjugated with quinoxaline (Route C, Scheme 1) [69]. Alternatively, aldehydes are also described as potent reagents to build [1,2,4]triazolo[4,3-*a*]quinoxalines with a variety of alkyl or aryl moieties (Route D, Scheme 1) [29,31,34,70]. Treatments of the hydrazinoquinoxaline intermediates with reagents like phosgene [71,72], thiophosgene [73], or carbone disulfide in pyridine [42,67] provide 1-oxo and 1-thio analogues, respectively (Route E, Scheme 1). Thioisocyanate in the presence of mercury acetate furnishes 1-alkylamino derivatives [48].

Consequently, different synthetic routes were considered by our team to build the [1,2,4]triazolo[4,3-*a*]quinoxalines, whose successes and failures are discussed below.

### 2.2. Previous Lab Strategy

**Scheme 2:**
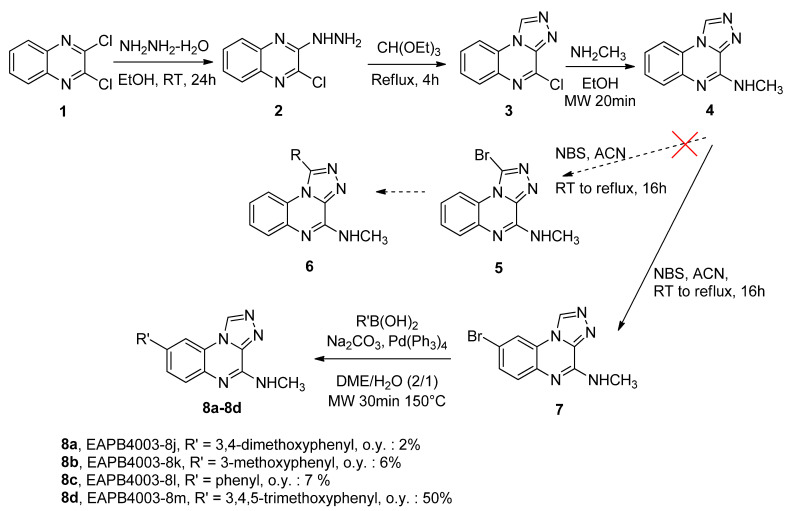
First considered synthetic route (o.y. for overall yield, dotted arrow for intended reactions, red cross for failed reaction).

A first synthetic route based on strategies used by our team for the preparation of other heterocyclic series has been considered (Scheme 2). It begins with a substitution reaction of one of the chlorines of 2,3-dichloroquinoxaline **1** by hydrazine hydrate to produce compound **2**. This reaction is carried out in ethanol at room temperature for 24 h [74]. The triazole intermediate **3** is then formed by the reaction between the hydrazine derivative **2** and triethyl orthoformate under reflux for 4 h [62]. The second chlorine is then substituted by methylamine under microwave (MW) irradiation at 140 °C for 20 min in ethanol to furnish **4**. After bromination at position 1, the obtained intermediate 1-bromo-*N*-methyl-[1,2,4]triazolo[4,3-*a*]quinoxalin-4-amine (**5**) would then have served as a common synthon for Suzuki–Miyaura coupling reactions with various boronic acids to build the novel compound series **6**. Many conditions for the bromination reaction have been attempted by varying temperature, N-bromosuccinimide (NBS) equivalents, solvents, and reaction time. The use of 1.2 eq of N-bromosuccinimide in acetonitrile, at ambient temperature then at reflux for 16 h, is the only condition that has made it possible to obtain a mainly monobrominated compound. ^1^H, ^13^C, and NOESY NMR analyses show that it is not position 1 that has been substituted, but that the used reaction conditions promote the production of the monobrominated compound **7** in position 8. Uçar et al. effectively described bromination reactions on quinoxaline and its derivatives under the conditions we used in the laboratory, confirming our conclusion from NMR analyses [75]. Indeed, NBS in the tested solvents is able to yield mono and/or di-brominated products on the aromatic ring of the quinoxaline unit. This kind of bromination reaction has also been described on phenazine in dichloromethane by Garrison et al. (1 to 4 h at room temperature [76]) and in toluene by Conda-Sheridan et al. (at 50 °C for 5 h [77]). The use of dibromine in acetonitrile (ACN) or methanol afforded the same result.

Although this strategy does not allow for obtaining a brominated intermediate for the preparation of a library of compounds variously substituted in position 1, the reaction sequence is continued, allowing us to describe unexpected new compounds for the [1,2,4]triazolo[4,3-*a*]quinoxaline series. The Suzuki–Miyaura cross-coupling reactions between **7** and appropriate aryl boronic acids according to the conditions already applied in our laboratory afforded compounds **8a**–**8d**. Purifications of these products were difficult due to their low solubility. Many solvents have been tried alone or as a mixture, such as ethyl acetate and/or cyclohexane, dichloromethane and/or methanol, or diethyl ether. Triturations simply removed a few impurities but did not produce pure products. On the one hand, silica chromatography’s purifications were not enough to properly purify the products, and on the other hand, this surely caused the loss of a large part of the quantity of the products. Preparative chromatographies were therefore carried out to better isolate the synthesized products, although the lack of solubility of the products in acetonitrile, methanol, or acetone also posed a problem. The obtained yields are quite low not only after purification but also in overall yields. If these compounds have any interesting activities, their syntheses must clearly be optimized.

### 2.3. Alternative Strategies

As the bromination conditions do not provide the desired synthon, it is therefore necessary to go back a few steps upstream to attempt a cyclization allowing for the direct obtainment of the tricyclic compounds substituted in position 1.

**Scheme 3:**
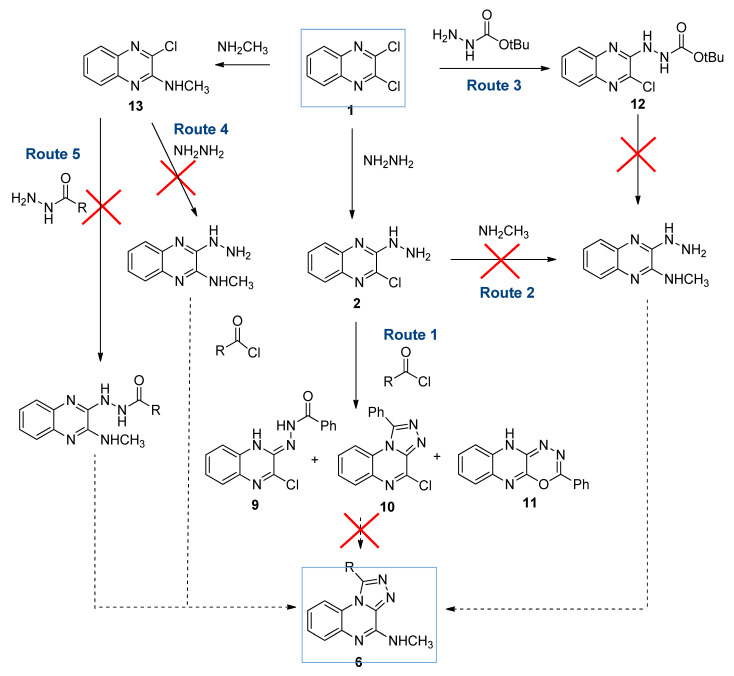
Synthetic routes considered by our team to obtain triazoloquinoxalines (dotted arrow for intended reactions, red cross for failed reactions).

The following strategy (route 1, Scheme 3) is based on the work of Shiho et al. [72] or Morten et al. [78], with the use of acid chlorides to form the functionalized triazole unit with different moieties. The cyclization reaction carried out with benzoyl chloride and intermediate **2** under several conditions in pyridine and/or acetonitrile provide the reaction intermediate **9** in trace amounts, the desired cyclized product **10** in very small amounts, and an undesired cyclized product **11**. This latter product comes from the intramolecular cyclization between the oxygen atom of the ketone function and the chlorine present on the quinoxaline unit of intermediate **9**, due to an amide–iminol tautomerism.

The presence of chlorine on the intermediate being problematic, methylamination was considered before the cyclization step (route 2, Scheme 3), but despite several attempts, none made it possible to obtain the substitution of the chlorine atom.

Since free hydrazine may be subject to parasitic reactions, we considered introducing a hydrazine that is monoprotected by a tert-butoxycarbonyl group in the presence of hydroxide potassium in acetonitrile at reflux for 24 h, as suggested by Kumar et al. [79]. Subsequent methylamination was not successful. A reverse attempt was tried but was also not successful (route 3, Scheme 3).

As described by Andrés et al., a monochloroquinoxaline derivative could react with a hydrazido reagent to produce the heterocycle under microwave irradiation [80]. After the substitution of one chlorine atom with methylamine to block a reactive position, hydrazine (route 4, Scheme 3) or a previously prepared hydrazido compound [81,82] (route 5, Scheme 3) in reaction with intermediate **13** did not allow us to obtain the desired heterocycle.

All these strategies did not allow us to obtain either a common synthon at the gram scale or in a reproducible synthetic way for future series exemplification. We therefore moved towards another strategy using aldehydes.

### 2.4. Selected General Chemical Strategy and Diversification

**Scheme 4:**
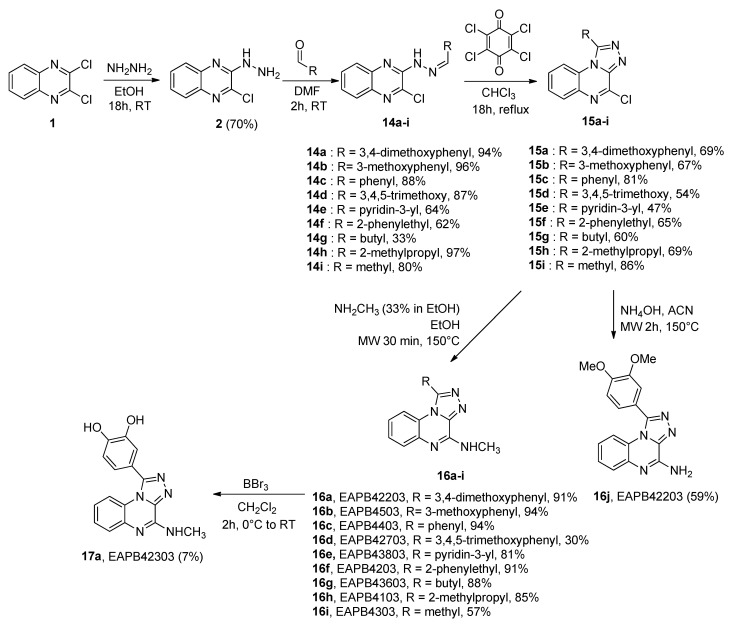
Synthetic route followed for our designed [1,2,4]triazolo[4,3-*a*]quinoxaline compounds.

A synthetic route for [1,2,4]triazolo[4,3-*a*]quinoxalines proposed by V. Krishnan et al. relied on the use of aldehydes to induce the heterocycle’s cyclization [83]. Commercial 2,3-dichloroquinoxaline 1 reacts with hydrazine hydrate in ethanol for 18 h at room temperature, giving 2. This intermediate is prepared from 5 g of reagent in order to have a large quantity to carry out the various compound syntheses for the series. It should be noted that the obtained product is a salmon-colored to red solid that does not require any downstream purification with 70% yield (Scheme 4).

The condensation between hydrazine of 2 and an aldehyde in dimethylformamide (DMF) for 2 h at ambient temperature furnishes compounds **14a**–**i** containing the hydrazone function. This reaction step allows for the introduction of a panel of aryl or alkyl substituents using corresponding aldehyde. The moieties are chosen according to the designs of the previous series prepared in the laboratory. 3,4-dimethoxybenzaldehyde allows for the introduction of the dimethoxyphenyl group into **14a**, which will be led after deprotection to a similar structure to EAPB02303, the lead compound of the Imiqualines family. The substituents of compounds **14b** (3-methoxyphenyl) and **14f** (2-phenylethyl) are, respectively, those of EAPB0503 and EAPB0203, the first-generation hits. The isobutyl group of compound **14i** is the substituent found in the imiquimod structure. The other substituents were used during the synthesis of the imidazo[1,2-*a*]- and imidazo[1,5-*a*]quinoxalinic series, except for 3-pyridine, which is introduced to test a new group. All those intermediates require purifications to be isolated, with yields varying over a range of 33 to 97%.

Subsequently, a cyclization with chloranil at reflux for 18 h allows for the production of triazoles conjugated with quinoxalines **15a**–**i**. The various 4-chloro-[1,2,4]triazolo[4,3-*a*]quinoxaline intermediates substituted in position 1 are obtained after purifications, with yields ranging from 47% to 86%. Chloranil, a tetrachlorinated derivative of quinone, is a mild commercial oxidant that allows for the formation of complex heterocycles with good yields [84]. A hypothetical mechanism for the formation of [1,2,4]triazolo[4,3-*a*]quinoxaline has been proposed by V. Krishnan [83]. The electrons from the carbonyl double bond are delocalized onto the oxygen of the chloranil. Oxygen, negatively charged, will deprotonate the hydrogen from the hydrazone function of the compound. Intramolecular cyclization of the compound is induced by delocalization of electrons. Indeed, negatively charged nitrogen forms a double bond between the hydrazone function and the quinoxaline ring. Then, the electrons of the double bond in the alpha of the new bond formed will delocalize to form a sigma bond with the carbon in the alpha of the hydrazone. The aromaticity of the triazoloquinoxaline ring is obtained by the elimination of the hydride, which reduces the last carbonyl of the chloranil derivative to hydroxyl. 2,3,5,6-tetrachlorobenzene-1,4-diol, the product of the reduction of chloranil, is eliminated during aqueous basic washing.

The last step allows for the substitution of chlorine in position 4 of **15a**–**i** under microwave assistance by methylamine in ethanol for 30 min at 150 °C to form compounds **16a**–**i** or ammonia in acetonitrile for 2 h at 150 °C to obtain compound **16j** respectively. The yields of this step vary between 30 to 94%. In order to differentiate the signals of protons in positions 6 and 9 of the triazoloquinoxaline series, and consequently the quaternary carbon C5a and C9a, it was necessary to perform a 2D NOESY NMR to observe the correlations between H9 and protons close in space. We arbitrarily chose compound 16f for this structural study. The analysis of the results shows a correlation between H9 of the quinoxaline ring and the proton H1’ of the ethylbenzene substituent (Supporting Information). This determination allows for identifying the other aromatic protons as well as carbons, and the quaternary carbons in particular.

Methyl ether’s deprotections of compound **16a** with boron tribromide in dichloromethane for 2 h furnishes the catechol **17a** (7%).

All the targeted compounds were obtained in four or five steps, with overall yields ranging from 4 to 60% (Table 1).

### 2.5. Predicted Physicochemical Parameters

Most promising drugs with noteworthy bioactivities fail to qualify as potential drug candidates due to their poor molecular drug-like properties. It is therefore important to determine the molecular structural properties at an early stage of compounds’ development. Drug-like properties, such as solubility, ionization, metabolic stability, and lipophilicity, are of critical importance for the success of drug candidates’ discovery. These data will help to anticipate and better ascertain the bioavailability, metabolism, clearance, toxicity, as well as in vitro pharmacology of novel drugs.

The use of the ACD Labs^®^ software (version 12.01) allow us to predict some of the physicochemical values of the described compounds **4**, **8a**–**d**, **16a**–**j**, **17a** (Table 1), and the lead EAPB02303.

Aqueous solubility is one of the most important physicochemical properties in modern drug discovery. It has an impact on ADME-related properties like drug uptake, distribution, and even bioavailability [85,86,87]. In our study, all the predictive results for aqueous solubility are logical. The [1,2,4]triazolo[4,3-*a*]quinoxaline scaffold **4** as well as the compound **16i** have similar aqueous solubilities. It is noted that if the size of the carbon chain is increased in position 1, the resulting compounds **16g** and **16h** will be less soluble. If this carbon chain is substituted by a phenyl, then the product **16f** is even less soluble. When the phenyl is directly grafted to the [1,2,4]triazolo[4,3-*a*]quinoxaline scaffold without a carbon chain as a linker, the aqueous solubility decreases by a factor of 10 for **16c** but does not change for **8c**. The exchange of a carbon atom by a nitrogen atom slightly improves the predictive value for **16e**. The substitution of the phenyl moiety by a single methoxy **16b** gives a prediction similar to that of phenyl derivative **16c**. The addition of two methoxy **16a** slightly increases the aqueous solubility. The trimethoxy-substituted **16d** is somewhat more soluble than the disubstituted one **16a**. The trend is more pronounced for the derivatives substituted in position 8: the trimethoxy **8d** is twice as soluble than the disubstituted **8a**, which is twice as soluble as the monomethoxy **8b** that is slightly more potent than **8c**. The replacement of the methylamine **16a** by a primary amine **16j** makes it possible to multiply the aqueous solubility by five. Obviously, the hydroxy compound is more soluble than the methoxy derivative (**17a** vs. **16a**).

Lipophilicity is represented by the partition coefficient logP and the distribution coefficient logD, which allow for the prediction of the in vivo behavior of active compounds in drug discovery [88,89]. In our study, the logP follows the trend of aqueous solubility. In other words, compounds with the lowest aqueous solubility (**16b** and **16c**) have the highest logPs, and compounds with the highest aqueous solubility values (**4** and **16i**) have the lowest logPs. In all cases, the logP values are positive, demonstrating the lipophilic nature of the prepared compounds.

LogD is a more refined measure of logP, because it takes into account the pH of the aqueous phase. The tumor microenvironment is known to be acidic. The pH values of the latter generally fluctuate between 6.4 and 7 but can also drop to 5.6 in tumors [90]. We therefore evaluated the logD values for each compound. Between pH 5.6 and 7, the value of logD follows the trend mentioned above and is almost identical to that of logP, with pH(water) = 7.4. When the pH is below 3.5, the logD values drop drastically. The logS is the inverse of the logD. These values display the same trend as logD.

The polar surface area (PSA) is the surface associated with heteroatoms (for example, oxygen, nitrogen, and phosphorous atoms) and donor/acceptor hydrogen atoms [91]. In our study, PSA correlates with the resulting lipophilicity of the prepared compounds. If the latter have all-carbon substituents (**8c**, **16c**, **16f**, **16g**, **16h**, **16i**), their PSA is identical to the basic scaffold **4**. The addition of methoxy on the phenyl increases the PSA accordingly (**8b**, **8a**, **8d** vs. **8c** and **16b**, **16a**, **16d** vs. **16c**). The substitution of a carbon atom by a nitrogen atom (**16c** vs. **16e**) logically increases the PSA, as well as the removal of a methyl (**16a** vs. **16j**).

**Figure 3:**
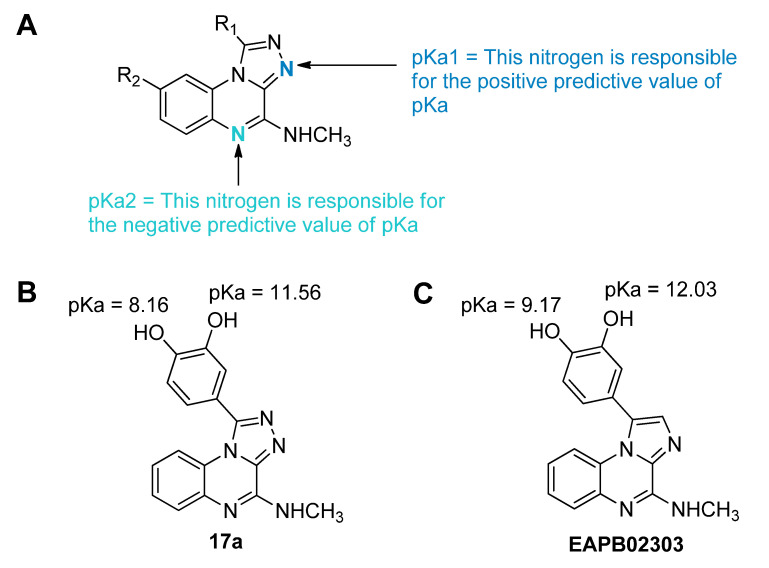
pKa value details: (**A**): [1,2,4]triazolo[4,3-*a*]quinoxaline scaffold for compounds **4**, **8a**–**d**, **16a**–**j, 17a**, (**B**): catechol moiety of **17a**, for (**C**): catechol moiety of EAPB02303.

The ionization constant (pKa) demonstrates the possibility for a molecule to ionize in various pH conditions and has a direct influence on many pharmacokinetic parameters [92]. The prepared compounds have at least two pKa. In very acidic conditions, the nitrogen in position 3 of the triazole is the first to be protonated no matter the compound (Figure 3A). In the [1,2,4]triazolo[4,3-*a*]quinoxaline series, the quinoxaline nitrogen has, no matter the compound, a negative pKa. The hydroxylated compound **17a** also has high pKas above 8 depending on the position of the hydroxyl group (Figure 3B): the predictive pKa in the para position is 8.16, whereas the predictive one in the meta position is above 11.

If the homologs EAPB02303 and **17a** of the two series are compared, it can be noted that even if the PSA is greater for **17a** due to the replacement of a carbon atom by a nitrogen atom, the resulting aqueous solubility is, however, two times lower, which is contrary to our expectations. A fortiori, the log P/D/S follow the same trend. Contrary to **17a**, EAPB02303 does not show a pKa value on the quinoxaline nitrogen. Catechol pKas are one point higher for EAPB02303 compared to **17a** (Figure 3C) and follow the same trend for meta and para positions.

All of these predictive values indicate that whatever the compound, it will be in a neutral form between pH 2.5 and 8. It should be noted that the protonation of the nitrogen in position 3 of the imidazole and the triazole directly induces a better solubility of the final compound.

### 2.6. In Vitro Cell Growth Inhibition Assay

First of all, we have evaluated compound **4** (EAPB4003, Table 2), which corresponds to the basic scaffold [1,2,4]triazolo[4,3-*a*]quinoxaline without any moiety in position 1 and methylaminated in position 4. At 10 µM, compound **4** is able to reduce A375 cell viability until 6%, which confirmed to us the SAR study interest of our novel [1,2,4]triazolo[4,3-*a*]quinoxaline subfamily. This cell line is used as our reference for Imiqualine compound screenings.

We then tested A375 cell viability for each compound (**8a**–**d**, **16a**–**j**, **17a**) at 10 µM. The residual cellular activity of compound **8a** to **8d** on A375 cell lines is unfortunately higher than 50% and showed no noteworthy efficacy (respectively, 96.9 ± 8.1% for **8a**, 95.0 ± 3.4% for **8b**, 50.7 ± 2.2% for **8c**, 90.6 ± 4.8% for **8d**). These results confirmed what we supposed for the imidazo[1,2-*a*]quinoxaline series: substitutions on position 8 of the quinoxaline with steric hindered and hydrophobic moieties cause a loss of activity [53]. Hydrophobic compounds with a linear alkyl chain in position 1, such as **16g** and **16i**, and the dimethoxyphenyl derivative **16j** do not display either activity on the A375 cell line (respectively, 76.6 ± 16.5% for **16g**, 85.4 ± 13.3% for **16i**, 98.6 ± 11.58% for **16j**). Due to the presence of the phenyl moiety in position 1 for compounds **16c** and **16f,** or a branched alkyl chain for compound **16h,** the residual cellular activity is slightly improved (respectively, 46.7 ± 2.1% for **16c**, 33.5 ± 13.3% for **16f**, 66.2 ± 5.9% for **16h**). The same trend is observed with moiety containing heteroatoms, such as in compounds **16d** (41.5 ± 0.8%) and **16e** (56.8 ± 7.0%). The best results were obtained with **16a**, **16b,** and **17a,** demonstrating the importance of the methylamine in position 4 and the concomitant presence of the 3,4-dimethoxyphenyl (2.9 ± 0.1%), 3-methoxyphenyl (11.3 ± 6.9%), or phenyl-3,4-diol (5.5 ± 1.2%) moieties in position 1. It should be noted that if the position homologs **8c** (50.7 ± 2.2%) and **16c** (46.7 ± 2.1%) demonstrate equivalent residual activity on the A375 cell line, the trisubstituted derivative **16d** (41.5 ± 0.8%) is twice as good as its **8d** (90.6 ± 4.8%) homolog. The disubstituted **16a** (2.9 ± 0.1%) and monosubstituted **16b** (11.3 ± 6.9%) derivatives demonstrate a particularly interesting activity, unlike their respective counterparts **8a** (96.9 ± 8.1%) and **8b** (95.0 ± 3.4%).

**Figure 4:**
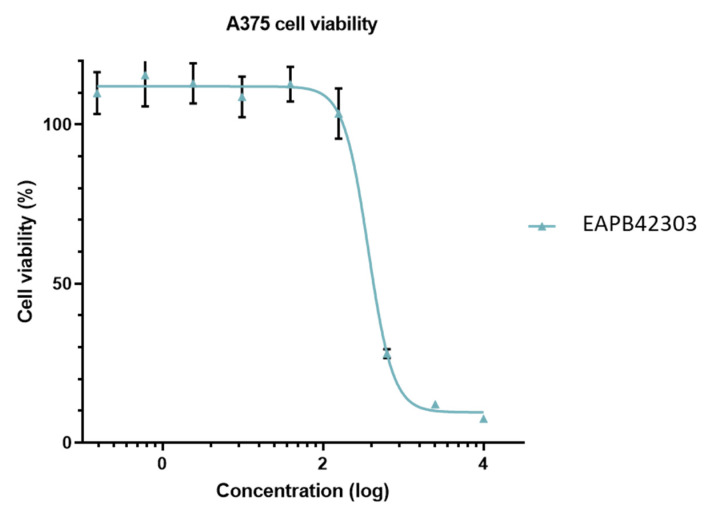
EC_50_ of compound **17a** (EAPB42303).

Among the Imiqualine family, we possess hits and leads that display very low-micromolar and nanomolar activities on multiple cancer cell lines. We are looking for compounds with interesting competitive activities. This is why we only determined the half-maximal effective concentration (EC_50_) of compounds displaying less than 20% of A375 residual cell viability at 10 µM. We then turned our attention on compounds **16a**, **16b,** and **17a**. EC_50_ was compared to our positive lead EAPB02303 and our hit EAPB0503 (Table 3) [51]. Compounds **16a** and **16b** have inhibitory activities of the order of 3 μM. The best compound of the [1,2,4]triazolo[4,3-*a*]quinoxaline series **17a** possesses an EC_50_ of 365 nM (Figure 4), equivalent to that of the hit EAPB0503. These effective concentrations confirm the importance of the 3,4-dimethoxyphenyl or 3-methoxyphenyl groups in position 1, and above all the powerful catechol moiety, if methylamine appears in position 4. These groups probably have special donor–acceptor interaction(s) with the target(s). Same observations have been highlighted with the imidazo[1,2-*a*]quinoxaline series [46,51,53,54,93].

These in vitro results clearly demonstrate the interest of a carbon atom in position 2 of the heterocycle core for antiproliferative activity on A375 cell line, even if the triazolo derivatives are not all devoid of activity. The nitrogen atom in position 2 probably disturbs interactions with the target, whether of a hydrophobic nature or of a pi-stacking nature.

## 3. Materials and Methods

### 3.1. Chemistry

#### 3.1.1. General

All solvents and reagents were obtained from Sigma Aldrich Chemical Co.^®^ (Saint Quention Fallavier, France), VWR^®^ (Rosny-sous-bois, France) and FluoroChem^®^ (Haldfield, UK) and used without further purification unless indicated otherwise. Thin-layer chromatography plates (Kieselgel 60 F254) were purchased from Merck^®^ (Saint Quentin Fallavier, Frnace). The progress of reactions was monitored by TLC exposure to UV light (254 nm and 366 nm).

Liquid chromatography mass spectroscopy (LC-MS) analyses were performed using a Micromass Q-Tof (Waters^®^) (Guyancourt, France) spectrometer coupled to an ESI (electrospray ionization mode) source and equipped with a Chromolith HighResolution RP-18th (25 × 4.6 mm) (Laboratoire de Mesures Physiques, Plateau technique de l’Institut des Biomolécules Max Mousseron, Université de Montpellier, Montpellier, France). The samples were previously separated using a gradient from 100% (H_2_O + 0.1% HCO_2_H) to 100% (ACN + 0.1% HCO_2_H) in 3 min using a flow rate of 3 mL/min and with UV detection at 214 nm. UPLC/MS analyses were recorded with an Acquity H-Class UPLC system, coupled to a Water SQDetector-2 mass spectrometer. The chromatographic separation was carried out under the same conditions as previously used with a Waters Acquity UPLC BEC C18 column (100 × 2.1 mm, 1.5 μm). Mass spectra were recorded in positive mode between 50 and 1500 Da.

Microwave-assisted organic syntheses were performed on a Biotage^®^ Initiator 2.0 Microwave. The reactions can be carried out over a temperature range of 40 to 300 °C and a pressure of up to 30 bars.

Purifications by normal-phase flash chromatography were carried out on a Biotage^®^ (Uppsala, Sweden) Selekt System. The products were detected by UV at 214 and 254 nm. The eluents available during the purifications were cyclohexane, ethyl acetate, dichloromethane, and methanol. The 25 g disposable prepackaged silica columns used were Biotage^®^ Sfär Silica D-Duo of 60 μm. The 10g and 50g disposable prepackaged silica columns used were Biotage^®^ SNAP Cartridges KP-Sil-50 µm.

Purifications by HPLC-preparative were carried out on a Gilson Armen 2250 using a Waters radial compression column of 40 × 100 mm in C18 simplified mode. The products were detected by UV at 214 nm. Separations were carried out with an H_2_O and ACN gradient.

High-Resolution Mass Spectroscopy (HRMS) analyses were carried out by direct introduction on a Synapt G2-S mass spectrometer (Waters, SN: UEB205) equipped with an ESI source (Laboratoire de Mesures Physiques, Plateau technique de l’Institut des Biomolécules Max Mousseron, Université de Montpellier, Montpellier, France). The mass spectra were recorded in positive mode, between 100 and 1500 Da. The capillary tension was 3000 V, and the cone tension was 30 V. The source and desolvation temperatures were 120 °C and 250 °C, respectively.

^1^H, ^13^C, and NOESY NMR spectra were obtained on Brüker^®^ AC-400, AC-500, and AC-600 spectrometers (Laboratoires de Mesures Physiques, Plateau technique de l’Institut des Biomolécules Max Mousseron, Université de Montpellier, IBMM, Montpellier, France). Chemical shifts are given as parts per million (ppm) using a residual dimethylsulfoxide signal for protons (δ DMSO = 2.46 ppm) and carbons (δ DMSO = 39.52 ppm). Abbreviations used for signal patterns are as follows: s, singlet; d, doublet; t, triplet; q, quartet; m, multiplet. Coupling constants are reported in Hertz (Hz).

#### 3.1.2. Procedures for Compounds’ Syntheses

Compound **2** was prepared as described by Alswah et al. [74]. Briefly, after solubilization of 2,3-dichloroquinoxaline (3 g, 15 mmol) in 75 mL of ethanol, hydrazine hydrate (1.7 mL, 33 mmol, 2.2 eq.) was added. The reaction was stirred overnight at room temperature. The resulting precipitate was filtered, and the solids were washed with ethanol and air dried to yield crude product (100% yield). C_8_H_7_ClN_4_. Mw = 194.62 g·mol^−1^. MS (ESI+, QTof, *m*/*z*): 195.0 [M + H]^+^ (Section 2.2, Scheme 2).

Compound **3** was prepared as described by Sarges et al. [62]. Briefly, a mixture of 2-chloro-3-hydrazinoquinoxaline (1 g, 5 mmol) and 20 mL of triethyl orthoformate was stirred at reflux for 4 h, cooled to room temperature, quenched with ether, and filtered. The solids were washed with ether and air dried. The compound was obtained as a white solid (66% yield). C_9_H_5_ClN_4_, Mw = 204.62 g.mol^−1^. MS (ESI+, QTof, *m*/*z*): 205.0 [M + H]^+^ (Section 2.2, Scheme 2).

*N-methyl-[1,2,4]triazolo[4,3-a]quinoxalin-4-yl*-amine (**4**). C_10_H_9_N_5_. Mw: 199.21 g·mol^−1^. A 33% (*w*/*v*) ethanol solution of methylamine (6.7 mL, 150.5 mmol) was added to a solution of 4-chloro-[1,2,4]triazolo[4,3-*a*]quinoxaline **3** (501 mg, 2.46 mmol) in 12 mL of ethanol in a microwave-adapted vial. The reaction was then submitted to microwave irradiations for 20 min at 140 °C. The resulting precipitate was filtered, and the solids were washed with distilled water and air dried to yield crude product **9**. Yield: 100%. MS (ESI+, QTof, *m*/*z*): 200.09 [M + H]^+^. HRMS calculated for C_10_H_10_N_5_ 200.0931, found 200.0934. ^1^H NMR (ppm, 500 MHz, DMSO-*d*_6_) δ 3.09 (d, 3H, NHCH_3_, *J* = 5 Hz), 7.39 (t, 1H, CH 7, *J* = 5 Hz), 7.49 (t, 1H, CH 8, *J* = 10 Hz), 7.69 (d, 1H, CH 9, *J* = 10 Hz), 8.20 (dd, 1H, CH 6, *J* = 10 Hz), 10.01 (s, 1H, CH 1). ^13^C NMR (ppm, 100 MHz, DMSO-*d*_6_) δ 26.18, 114.67, 119.92, 122.05, 122.96, 126.11, 136.61, 136.85, 144.26 (Section 2.2, Scheme 2 and Supporting Materials, S1).

*8-Bromo-[1,2,4]triazolo[4,3-a]quinoxalin-4-yl)-methyl-amine* (**7**). C_10_H_8_BrN_5_. Mw: 278.11 g·mol^−1^. NBS (430 mg, 2.41 mmol) was added to compound **4** (401 mg, 2.01 mmol) in acetonitrile. The reaction was stirred for 1 h at room temperature, then at reflux until 24 h elapsed. The solvent was removed under reduced pressure. The crude product was dissolved in ethyl acetate and successively washed with saturated aqueous chloride ammonium, carbonate sodium, water, and brine. The organic phase was dried with sodium sulphate, filtered, and concentrated under reduced pressure. Purification was performed on a flash chromatography system eluted with ethyl acetate/cyclohexane 0/10 to 6/4 (*v*/*v*). Compound **7** was obtained as a white solid. Yield: 15%. ^1^H NMR (ppm, 400 MHz, DMSO-*d*_6_) δ 3.06 (d, 3H, NHCH_3_, *J* = 4 Hz), 7.52 (d, 1H, CH 6, *J* = 7 Hz), 7.57 (dd, 1H, CH 7, *J* = 7 Hz, *J* = 1.5 Hz), 8.24 (q, 1H, NH, *J* = 4 Hz), 8.46 (d, 1H, CH 9, *J* = 1.5 Hz), 9.92 (s, 1H, CH 1). ^13^C NMR (ppm, 100 MHz, DMSO-*d*_6_) δ 27.79, 114.82, 119.39, 123.48, 128.19, 130.71, 137.05, 138.55, 138.71, 146.94 (Section 2.2, Scheme 2 and Supporting Materials, S2).

- **Procedure for the Suzuki–Miyaura cross-coupling reactions in position 8.**

**General procedure:**

After solubilization of compound **7** (1 eq.) in DME/H_2_O (2/1 (*v*/*v*), 15 mL), boronic acid (1.1 eq.), tetrakis(triphenylphosphine)palladium (0.05 eq.), and sodium carbonate (2 eq.) were added in a microwave-adapted vial (10–20 mL). The reaction was submitted to microwave irradiation for 20 min at 140 °C. Then, the crude mixture was filtrated, washed with MeOH, and concentrated under reduced pressure. An aqueous saturated solution of NaCl was added to a separating funnel containing our filtrate. Then, the aqueous phase was extracted with CH_2_Cl_2_ (2 times). The organic phase was concentrated under reduced pressure and purified on preparative chromatography with acetonitrile/H_2_O 1‰ TFA. The collected fractions were lyophilized.

*[8-(3,4-Dimethoxy-phenyl)-[1,2,4]triazolo[4,3-a]quinoxalin-4-yl]-methyl-amine* (**8a**). C_18_H_17_N_5_O_2_. Mw: 335.36 g·mol^−1^. Compound **7** (480 mg, 1.73 mmol), 3,4-dimethoxyphenylboronic acid (347 mg, 1.91 mmol), tetrakis(triphenylphosphine) palladium (100 mg, 0.087 mmol), and sodium carbonate (367 mg, 3.47 mmol). Compound **8a** was obtained as a green solid (2.1% overall yield). MS (ESI+, QToF, *m*/*z*): 336.15 [M + H]^+^. HRMS calculated for C_18_H_17_N_5_O_2_ 336.1455, found 336.1449. ^1^H NMR (ppm, 600 MHz, DMSO-*d*_6_) δ 3.12 (d, 3H, NHCH_3_, *J* = 4 Hz), 3.82 (s, 3H, OCH_3_), 3.90 (s, 3H, OCH_3_), 7.09 (d, 1H, CH 5′, *J* = 9 Hz), 7.38 (dd, 1H, CH 6′, *J* = 8 Hz, *J* = 2 Hz), 7.39 (s, 1H, CH 2′), 7.75 (d, 1H, CH 6, *J* = 9 Hz), 7.83 (dd, 1H, CH 7, *J* = 8 Hz, *J* = 2 Hz), 8.49 (d, 1H, CH 9, *J* = 2 Hz), 10.13 (s, 1H, CH 1). ^13^C NMR (ppm, 100 MHz, DMSO-*d*_6_) 26.31, 54.01, 54.13, 108.90, 110.61, 111.93, 117.41, 120.34, 123.18, 124.06, 129.76, 132.34, 133.99, 136.80, 136.98, 144.11, 147.20, 147.54 (Section 2.2, Scheme 2 and Supporting Materials, S3.)

*[8-(3-Methoxy-phenyl)-[1,2,4]triazolo[4,3-a]quinoxalin-4-yl]-methyl-amine* (**8b**). C_17_H_15_N_5_O. Mw: 305.33 g·mol^−1^. Compound **7** (250 mg, 0.90 mmol), 3-methoxyphenylboronic acid (151 mg, 0.99 mmol), tetrakis(triphenylphosphine) palladium (52 mg, 0.045 mmol), and sodium carbonate (191 mg, 1.81 mmol). Compound **8b** was obtained as a white solid (5.8% overall yield). MS (ESI+, QToF, *m*/*z*): 306.14 [M + H]^+^. HRMS calculated for C_17_H_16_N_5_O 306.1349, found 306.1358. ^1^H NMR (ppm, 500 MHz, DMSO*-d*_6_) 3.10 (d, 3H, NHCH_3_, *J* = 5 Hz), 3.87 (s, 3H, OCH_3_), 6.99 (ddd, 1H, CH 4′, *J* = 8 Hz, *J* = 3 Hz, *J* = 2 Hz), 7.41 (m, 3H, CH 2′, CH 5′, CH 6′), 7.71 (d, 1H, CH 6, *J* = 10 Hz), 7.84 (dd, 1H, CH 7, *J* = 10 Hz, *J* = 2 Hz), 8.56 (d, CH 9, *J* = 2 Hz), 10.13 (s, CH 1). ^13^C NMR (ppm, 100 MHz, DMSO*-d*_6_) δ 26.04, 53.53, 110.67, 111.37, 112.54, 117.31, 120.41, 123.71, 124.28, 128.43, 133.40, 133.69, 136.70, 136.88, 138.51, 144.35, 158.16 (Section 2.2, Scheme 2 and Supporting Materials, S4).

*Methyl-(8-phenyl-[1,2,4]triazolo[4,3-a]quinoxalin-4-yl)-amine* (**8c**). C_16_H_13_N_5_. Mw: 275.31 g·mol^−1^. Compound **7** (250 mg, 0.90 mmol), phenylboronic acid (121 mg, 0.99 mmol), tetrakis(triphenylphosphine) palladium (52 mg, 0.045 mmol), and sodium carbonate (191 mg, 1.81 mmol). Compound **8c** was obtained as a white solid (6.5% overall yield). MS (ESI+, QToF, *m*/*z*): 216.12 [M + H]^+^. HRMS calculated for C_16_H_14_N_5_ 276.1244, found 276.1245. ^1^H NMR (ppm, 400 MHz, DMSO*-d*_6_) 3.11 (d, 3H, NHCH_3_, *J* = 4 Hz), 7.42 (tt, 1H, CH 4′, *J* = 8 Hz, *J* = 1 Hz), 7.53 (td, 2H, CH 3′, CH 5′, *J* = 8 Hz, *J* = 2 Hz), 7.73 (d, 1H, CH 6, *J* = 8 Hz), 7.84 (m, 3H, CH 7, CH 2′, CH 6′), 8.57 (d, 1H, CH 9, *J* = 2 Hz), 10.12 (s, 1H, CH 1). ^13^C NMR (ppm, 100 MHz, DMSO*-d*_6_) δ 28.20 (NHCH_3_), 114.62, 122.61, 125.89, 126.34, 127.14, 128.16, 129.49, 135.79, 138.80, 139.04, 139.24, 146.50 (Section 2.2, Scheme 2 and Supporting Materials, S5).

*Methyl-[8-(3,4,5-trimethoxy-phenyl)-[1,2,4]triazolo[4,3-a]quinoxalin-4-yl]-amine* (**8d**). C_19_H_19_N_5_O_3_. Mw: 365.39 g·mol^−1^. Compound **7** (250 mg, 0.90 mmol), 3,4,5-trimethoxyphenylboronic acid (210 mg, 0.99 mmol), tetrakis(triphenylphosphine) palladium (52 mg, 0.045 mmol), and sodium carbonate (191 mg, 1.81 mmol). Compound **8d** was obtained as a white solid (49.5% overall yield). ^1^H NMR (ppm, 400 MHz, DMSO*-d*_6_) 3.08 (d, 3H, NHCH_3_, *J* = 4 Hz), 3.70 (s, 3H, OCH_3_), 3.92 (s, 6H, OCH_3_, OCH_3_), 7.08 (s, 2H, CH 2′, CH 6′), 7.67 (d, 1H, CH 6, *J* = 9 Hz), 7.83 (dd, 1H, CH 7, *J* = 8 Hz, *J* = 2 Hz), 8.32 (q, 1H, NH, *J* = 4 Hz), 8.46 (d, 1H, CH 9, *J* = 2 Hz), 10.08 (s, 3H, CH 1). ^13^C NMR (ppm, 100 MHz, DMSO*-d*_6_) δ 27.87, 56.58, 60.60, 104.78, 114.40, 122.58, 126.38, 126.67, 135.16, 135.55, 136.98, 137.81, 138.52, 138.96, 146.61, 153.76 (Section 2.2, Scheme 2 and Supporting Materials, S6).

- **Procedure for condensation with aldehydes and compound 2.**

**General procedure:**

Compound **2** (0.505 g, 2.59 mmol, 1.0 eq) was dissolved in DMF (5 mL). Aldehyde (2.59 mmol, 1.0 eq) was then added, and the resulting solution was stirred for 2 h at rt. The reaction mixture was dissolved in distillated water and extracted with ethyl acetate. The organic layer was washed twice with brine, dried with Na_2_SO_4_, and concentrated to dryness under reduced pressure. The desired product was purified by flash chromatography.

Due to their insolubilities, compounds in group **14** displayed difficulties for NMR data recording, especially for quaternary carbons, even when we tried to heat NMR tubes during analysis and/or recording on a Brüker AC-600 spectrometer. **14d**, **14e** and **14i** were the most impacted compounds.

*2-chloro-3-(2-(3,4-dimethoxybenzylidene)hydrazinyl)quinoxaline* (**14a**). C_17_H_15_ClN_4_O_2_. Mw: 342.78 g·mol^−1^. The product was purified by flash chromatography on silica gel (Cyclohexane/EtOAc 100:0 to 30:70) to afford the desired product as an orange solid. Yield: 94%. tr: 3.11 min, MS (ESI+, QTof, *m*/*z*): 343.3 [M + H]^+^. ^1^H NMR (ppm, 400 MHz, DMSO-*d*_6_) δ 11.18 (s, 1H) NH, 8.56 (s, 1H) CH 3′, 7.84–7.14 (m, 6H) CH 5/6/7/8/5′/9′, 7.05 (d, *J* = 8.3 Hz, 1H) CH 8′, 3.88 (s, 3H) OCH_3_, 3.86 (s, 3H) OCH_3_. ^13^C NMR (ppm, 100 MHz, DMSO-*d*_6_) δ 170.4, 157.4, 150.6, 147.7, 130.6, 127.7, 127.5, 126.3, 125.6, 121.5, 115.2, 111.6, 111.4, 110.4, 109.6, 55.6, 55.5 (Section 2.4, Scheme 4 and Supporting Materials, S7).

*2-chloro-3-(2-(3-methoxybenzylidene)hydrazinyl)quinoxaline* (**14b**). C_16_H_13_ClN_4_O. Mw: 312.75 g·mol^−1^. The product was purified by flash chromatography on silica gel (Cyclohexane/EtOAc 100:0 to 80:20) to afford the desired product as a red solid. Yield: 96%. tr: 3.37 min, MS (ESI+, QTof, *m*/*z*): 313.3 [M + H]^+^. ^1^H NMR (ppm, 600 MHz, DMSO-*d*_6_) δ, 8.06 (dd, *J* = 8.0, 1.3 Hz, 1H) CH 8 or 5, 7.66 (td, *J* = 7.7, 1.2 Hz, 1H) CH 6 or 7, 7.62 (t, *J* = 7.8 Hz, 1H) CH 8′, 7.58 (td, *J* = 7.3, 1.5 Hz, 1H) CH 7 or 6, 7.41 (dd, *J* = 8.5, 1.0 Hz, 1H) CH 5 or 8, 7.34–7.31 (m, 3H) CH 5′/9′/7′, 3.83 (s, 3H) OCH_3_. ^13^C NMR (ppm, 150 MHz, DMSO-*d*_6_) δ 159.5, 150.5, 142.6, 141.2, 135.1, 130.6, 129.8, 129.5, 129.00, 128.0, 126.1, 122.1, 117.1, 115.9, 115.3, 55.5 (Section 2.4, Scheme 4 and Supporting Materials, S8).

*2-(2-benzylidenehydrazinyl)-3-chloroquinoxaline* (**14c**). C_15_H_11_ClN_4_. Mw: 282.73 g·mol^−1^. The product was purified by flash chromatography on silica gel (Cyclohexane/EtOAc 100:0 to 50:50) to afford the desired product as a yellow solid. Yield: 88%). tr: 3.31 min, MS (ESI+, QTof, *m*/*z*): 283.2 [M + H]^+^. ^1^H NMR (ppm, 400 MHz, DMSO-*d*_6_) δ 11.09 (s, 1H) NH, 8.64 (s, 1H) CH 3′, 8.16–7.70 (m, 5H) 5× CH_Ar_, 7.60–7.38 (m, 4H) 4× CH_Ar_. ^13^C NMR (ppm, 150 MHz, DMSO-*d*_6_) δ 158.0, 147.7, 145.4, 141.1, 137.3, 136.7, 135.2, 131.1, 130.3, 129.3, 128.1, 127.4, 126.8, 126.6, 123.1 (Section 2.4, Scheme 4 and Supporting Materials, S9).

*2-chloro-3-(2-(3,4,5-trimethoxybenzylidene)hydrazinyl)quinoxaline* (**14d**). C_18_H_17_ClN_4_O_3_. Mw: 372.81 g·mol^−1^. The product was purified by flash chromatography on silica gel (Cyclohexane/EtOAc 100:0 to 0:100) to afford the desired product as a yellow solid. Yield: 87%. tr: 3.18 min, MS (ESI+, QTof, *m*/*z*): 373.3 [M + H]^+^. ^1^H NMR (ppm, 600 MHz, DMSO-*d*_6_) δ 11.15 (m, 1H) NH, 8.54 (s, 1H) CH 3′, 7.91–7.06 (m, 6H) 6 × CH_Ar_, 3.88 (s, 6H) 2× OCH_3_, 3.73 (s, 3H) OCH_3_. ^13^C NMR (150 MHz, DMSO) δ 157.3, 153.2, 147.3, 140.6, 130.6, 130.2, 127.6, 126.4, 126.0, 122.6, 115.3, 106.08, 104.2, 60.1, 56.0 (Section 2.4, Scheme 4 and Supporting Materials, S10).

*2-chloro-3-(2-(pyridin-3-ylmethylene)hydrazinyl)quinoxaline* (**14e**). C_14_H_10_ClN_5_. Mw: 283.72 g·mol^−1^. The product was purified by flash chromatography on silica gel (Cyclohexane/EtOAc 100:0 to 0:100) to afford the desired product as a yellow solid. Yield: 87%. tr: 2.00 min, MS (ESI+, QTof, *m*/*z*): 284.2 [M + H]^+^. ^1^H NMR (ppm, 600 MHz, DMSO-*d*_6_) δ 11.29 (s, 1H) NH, 9.00 (s, 1H) CH 3’, 8.67 (s, 1H) CH 5′, 8.63 (dd, *J* = 4.7, 1.7 Hz, 1H) CH 7’, 8.27 (s, 1H) CH 9’, 7.75 (s, 2H) CH_Ar_, 7.63 (s, 1H) CH_Ar_, 7.52 (dd, *J* = 7.8, 4.8 Hz, 1H) CH 8′, 7.42 (s, 1H) CH_Ar_. ^13^C NMR (ppm, 150 MHz, DMSO) δ 151.1, 149.5, 145.2, 134.4, 131.2, 131.1, 128.1, 125.6, 124.4 (Section 2.4, Scheme 4 and Supporting Materials, S11).

*2-chloro-3-(2-(3-phenylpropylidene)hydrazinyl)quinoxaline* (**14f**). C_17_H_15_ClN_4_. Mw: 310.78 g·mol^−1^. The product was purified by flash chromatography on silica gel (Cyclohexane/EtOAc 100:0 to 80:20) to afford the desired product as a red oil. Yield: 62%. tr: 3.50 min, MS (ESI+, QTof, *m*/*z*): 311.2 [M + H]^+^. ^1^H NMR (ppm, 400 MHz, DMSO-*d*_6_) δ 10.66 (s, 1H) NH, 7.94 (t, *J* = 5.3 Hz, 1H) CH 3′, 7.80 (d, *J* = 8.2 Hz, 1H) CH 8, 7.76 (d, *J* = 8.4 Hz, 1H) CH 5, 7.67 (t, *J* = 7.6 Hz, 1H) CH 6, 7.49 (t, *J* = 8.2 Hz, 1H) CH 7, 7.33–7.28 (m, 4H) CH 7′/8′/10′/11′, 7.23–7.17 (m, 1H) CH 9′, 2.86 (t, *J* = 7.4 Hz, 2H) CH_2_ 5′, *2.*65 (m, 2H) CH_2_ 4′. ^13^C NMR (ppm, 100 MHz, DMSO-*d*_6_) δ 151.6, 145.5, 141.4, 141.1, 137.0, 136.4, 130.9, 130.9, 128.9, 128.8, 127.9, 126.6, 126.4, 126.1, 122.7, 34.4, 32.8 (Section 2.4, Scheme 4 and Supporting Materials, S12).

*2-chloro-3-(2-pentylidenehydrazinyl)quinoxaline* (**14g**). C_13_H_15_ClN_4_. Mw: 262.74 g·mol^−1^. The product was purified by flash chromatography on silica gel (Cyclohexane/EtOAc 100:0 to 80:20) to afford the desired product as an orange oil. Yield: 33%. tr: 3.32 min, MS (ESI+, QTof, *m*/*z*): 263.2 [M + H]^+^. ^1^H NMR (ppm, 400 MHz, DMSO-*d*_6_) δ 10.36 (s, 1H) NH, 7.90 (t, *J* = 5.6 Hz, 1H) CH 3′, 7.83–7.41 (m, 4H) CH 5/6/7/8, 2.36 (dd, *J* = 3.3, 1.3 Hz, 2H) CH_2_ 4′, 1.55 (dt, *J* = 14.7, 7.3 Hz, 2H) CH_2_ 5′, 1.41 (qd, *J* = 14.2, 7.2 Hz, 2H) CH_2_ 6′, 0.94 (t, *J* = 7.3 Hz, 3H) CH_3_ 7′. ^13^C NMR (ppm, 100 MHz, DMSO-*d*_6_) δ 130.8, 130.6, 129.8, 128.0, 127.9, 124.5, 117.0, 32.3, 28.9, 22.2, 14.0 (Section 2.4, Scheme 4 and Supporting Materials, S13).

*2-chloro-3-(2-(3-methylbutylidene)hydrazinyl)quinoxaline* (**14h**). C_13_H_15_ClN_4_. Mw: 262.74 g·mol^−1^. The product was purified by flash chromatography on silica gel (Cyclohexane/EtOAc 100:0 to 75:25) to afford the desired product as a beige solid. Yield: 97%. tr: 3.27 min, MS (ESI+, QTof, *m*/*z*): 263.2 [M + H]^+^. ^1^H NMR (ppm, 400 MHz, DMSO-*d*_6_) δ 10.60 (s, 1H) NH, 7.92 (t, *J* = 5.7 Hz, 1H) CH 3′, 7.78 (d, *J* = 8.5 Hz, 1H) CH 8, 7.75 (d, *J* = 8.9 Hz, 1H) CH 5, 7.66 (t, *J* = 7.3 Hz, 1H) CH 6, 7.48 (t, *J* = 7.2 Hz, 1H) CH 7, 2.23 (t, *J* = 6.0 Hz, 2H) CH_2_ 4′, 1.93–1.80 (m, 1H) CH 5′, 0.97 (s, 3H) CH_3_, 0.96 (s, 3H) CH_3_. ^13^C NMR (ppm, 100 MHz, DMSO-*d*_6_) δ 151.7, 145.1, 140.7, 136.5, 135.9, 130.4, 127.5, 126.1, 125.6, 40.8, 26.6, 22.6 (Section 2.4, Scheme 4 and Supporting Materials, S14).

*2-chloro-3-(2-ethylidenehydrazinyl)quinoxaline* (**14i**). C_10_H_9_ClN_4_. Mw: 220.66 g·mol^−1^. The product was purified by flash chromatography on silica gel (Cyclohexane/EtOAc 100:0 to 70:30) to afford an orange solid. Yield: 80% (purity 75%). tr: 2.25 min, MS (ESI+, QTof, *m*/*z*): 221.2 [M + H]^+^. Due to its instability, **14i** was quickly used for the cyclization step. NMR recording was not possible. **14i** showed degradation for each test (Section 2.4, Scheme 4 and Supporting Materials, S15).

- **Procedure for the cyclization with chloranil.**

**General procedure:**

Chloranil was added to hydrazinyl derivative **14** in CHCl_3_. The reaction mixture was heated to reflux for 24 h. After being cooled, the crude mixture was diluted in 200 mL of CHCl_3_ and washed with NaOH 10%, then with water, and finally with brine. The organic layer was dried with Na_2_SO_4_, filtered, and concentrated under reduced pressure. The residue was purified through flash chromatography on silica gel to afford the desired product.

*4-chloro-1-(3,4-dimethoxyphenyl)-[1,2,4]triazolo[4,3-a]quinoxaline* (**15a**). C_17_H_13_ClN_4_O_2_. Mw: 340.76 g·mol^−1^. Chloranil (0.687 g, 2.77 mmol, 1.2 eq) was added to **14a** (0.798 g, 2.573 mmol, 1.0 eq) in CHCl_3_ (40 mL). The residue was purified through flash chromatography on silica gel (Cyclohexane/EtOAc 100:0 to 50:50) to afford the desired product as a brown solid. Yield: 69%. tr: 2.73 min, MS (ESI+, QTof, *m*/*z*): 341.3 [M + H]^+^. ^1^H NMR (ppm, 600 MHz, DMSO-*d*_6_) δ 8.04 (dd, *J* = 8.0, 1.3 Hz, 1H) CH 6, 7.66 (t, *J* = 7.9 Hz, 1H) CH 7, 7.59 (td, *J* = 7.9, 1.2 Hz, 1H) CH 8, 7.49 (dd, *J* = 8.4, 0.9 Hz, 1H) CH 9, 7.35 (d, *J* = 1.8 Hz, 1H) CH 2′, 7.32 (dd, *J* = 8.2, 1.9 Hz, 1H) CH 6′, 7.26 (d, *J* = 8.3 Hz, 1H) CH 5′, 3.91 (s, 3H) OCH_3_, 3.76 (s, 3H) OCH_3_. ^13^C NMR (ppm, 150 MHz, DMSO-*d*_6_) δ 150.9, 150.8, 148.9, 142.4, 141.2, 135.1, 129.7, 129.4, 127.9, 126.2, 122.9, 119.5, 115.9, 113.1, 111.9, 55.7, 55.6 (Section 2.4, Scheme 4 and Supporting Materials, S16).

*4-chloro-1-(3-methoxyphenyl)-[1,2,4]triazolo[4,3-a]quinoxaline* (**15b**). C_16_H_11_ClN_4_O. Mw: 310.74 g·mol^−1^. Chloranil (0.591 g, 2.4 mmol, 1 eq) was added to **14b** (0.752 g, 2.4 mmol, 1.0 eq) in CHCl_3_ (50 mL). The residue was purified through flash chromatography on silica gel (Cyclohexane/EtOAc 100:0 to 60:40) to afford the desired product as a white solid. Yield: 67%. tr: 2.92 min, MS (ESI+, QTof, *m*/*z*): 311.2 [M + H]^+^. ^1^H NMR (ppm, 400 MHz, DMSO-*d*_6_) δ 8.04 (dd, *J* = 7.4, 0.7 Hz, 1H) CH 6, 7.66 (t, *J* = 7.7 Hz, 1H) CH 7, 7.62 (t, *J* = 7.7 Hz, 1H) CH 5′, 7.58 (t, *J* = 7.4 Hz, 1H) CH 8, 7.40 (d, *J* = 8.4 Hz, 1H) CH 9, 7.34 (s, 1H) CH 2′, 7.31 (dd, *J* = 7.9, 3.0 Hz, 2H) CH 4′ and CH 6′, 3.83 (s, 3H) OCH_3_. ^13^C NMR (ppm, 400MHz, DMSO) δ 159.5, 150.5, 142.5, 141.2, 135.1, 130.5, 129.7, 129.5, 128.9, 127.9, 126.1, 122.0, 117.0, 115.8, 115.3, 55.5 (Section 2.4, Scheme 4 and Supporting Materials, S17).

*4-chloro-1-phenyl-[1,2,4]triazolo[4,3-a]quinoxaline* (**15c**). C_15_H_9_ClN_4_. Mw: 280.71 g·mol^−1^. Chloranil (0.7 g, 2.85 mmol, 1.2 eq) was added to **14c** (0.583 g, 2.06 mmol, 1.0 eq) in CHCl_3_ (40 mL). The residue was purified through flash chromatography on silica gel (Cyclohexane/EtOAc 100:0 to 70:30) to afford the desired product as a beige solid. Yield: 81%. tr: 2.80 min, MS (ESI+, QTof, *m*/*z*): 281.3 [M + H]^+^. ^1^H NMR (ppm, 600 MHz, CDCl_3_) δ 8.06 (dd, *J* = 8.1, 1.2 Hz, 1H) CH 6, 7.71 (m, 3H) CH 2′/4′/6′, 7.65 (m, 2H) CH 3′/5′, 7.60 (t, *J* = 7.7 Hz, 1H) CH 7, 7.54 (d, *J* = 8.2 Hz, 1H) CH 9, 7.41 (td, *J* = 7.4, 1.2 Hz, 1H) CH 8. ^13^C NMR (ppm, 600 MHz, CDCl_3_) δ 151.3, 143.1, 142.6, 135.9, 131.6, 130.4, 130.2, 129.7, 129.5, 128.4, 127.6, 125.9, 116.1 (Section 2.4, Scheme 4 and Supporting Materials, S18).

*4-chloro-1-(3,4,5-trimethoxyphenyl)-[1,2,4]triazolo[4,3-a]quinoxaline* (**15d**). C_18_H_15_ClN_4_O_3_. Mw: 370.79 g·mol^−1^. Chloranil (0.654 g, 2.66 mmol, 1.2 eq) was added to **14d** (0.826 g, 2.2 mmol, 1.0 eq) in CHCl_3_ (40 mL). The residue was purified through flash chromatography on silica gel (Cyclohexane/EtOAc 100:0 to 0:100) to afford the desired product as a yellow solid. Yield: 54%. tr: 2.84 min, MS (ESI+, QTof, *m*/*z*): 371.3 [M + H]^+^. ^1^H NMR (ppm, 500 MHz, DMSO-*d*_6_) δ 8.06 (dd, *J* = 7.9, 1.5 Hz, 1H) CH 6, 7.68 (td, *J* = 7.7, 1.4 Hz, 1H) CH 7, 7.65 (td, *J* = 7.5, 1.2 Hz, 1H) CH 8, 7.50 (dd, *J* = 8.4, 1.2 Hz, 1H) CH 9, 7.10 (s, 2H) CH 2′ and CH 6′, 3.82 (s, 6H) 2× OCH_3_, 3.79 (s, 3H) OCH_3_. ^13^C NMR (ppm, 125 MHz, DMSO-*d*_6_) δ 153.8, 151.1, 141.6, 139.9, 135.6, 130.4, 129.9, 128.5, 126.6, 123.2, 116.6, 107.9, 60.8, 56.7 (Section 2.4, Scheme 4 and Supporting Materials, S19).

*4-chloro-1-(pyridin-3-yl)-[1,2,4]triazolo[4,3-a]quinoxaline* (**15e**). C_14_H_8_ClN_5_. Mw: 281.70 g·mol^−1^. Chloranil (0.487 g, 2.98 mmol, 1.2 eq) was added to **14e** (0.468 g, 1.65 mmol, 1.0 eq) in CHCl_3_ (40 mL),. The residue was purified through flash chromatography on silica gel (Cyclohexane/EtOAc 100:0 to 0:100) to afford the desired product as a brown solid. Yield: 47%. tr: 2.08 min, MS (ESI+, QTof, *m*/*z*): 282.3 [M + H]^+^. ^1^H NMR (ppm, 600 MHz, DMSO-*d*_6_) δ 8.97 (dd, *J* = 4.6, 2.3 Hz, 1H) CH 2′, 8.94 (dd, *J* = 4.9, 1.7 Hz, 1H) CH 4′, 8.25 (ddd, *J* = 6.7, 4.4, 2.3 Hz, 1H) CH 6′, 8.09–8.05 (m, 1H) CH 6, 7.77–7.74 (m, 1H) CH 5′, 7.69 (td, *J* = 7.8, 1.2 Hz, 1H) CH 7, 7.59 (ddd, *J* = 8.7, 7.4, 1.5 Hz, 1H) CH 8, 7.37 (dd, *J* = 8.5, 1.0 Hz, 1H) CH 9. ^13^C NMR (ppm, 150 MHz, DMSO-*d*_6_) δ 152.1, 150.0, 148.4, 142.9, 141.1, 137.7, 135.1, 129.9, 129.6, 128.2, 126.0, 124.4, 124.1, 115.7 (Section 2.4, Scheme 4 and Supporting Materials, S20).

*4-chloro-1-phenethyl-[1,2,4]triazolo[4,3-a]quinoxaline* (**15f**). C_17_H_13_ClN_4_. Mw: 308.77 g.mol^−1^. Chloranil (0.37 g, 1.51 mmol, 1 eq) was added to **14f** (0.468 g, 1.51 mmol, 1.0 eq) in CHCl_3_ (50 mL). The residue was purified through flash chromatography on silica gel (Cyclohexane/EtOAc 100:0 to 60:40) to afford the desired product as a brown oil. Yield: 65%. tr: 3.14 min, MS (ESI+, QTof, *m*/*z*): 309.3 [M + H]^+^. ^1^H NMR (ppm, 600 MHz, DMSO-*d*_6_) δ 8.33 (d, *J* = 8.0 Hz, 1H) CH 9, 8.03 (dd, *J* = 8.0, 1.4 Hz, 1H) CH 6, 7.86–7.78 (m, 1H) CH 8, 7.72 (t, *J* = 7.2 Hz, 1H) CH 7, 7.40 (d, *J* = 7.3 Hz, 2H) CH 4′ and CH 8′, 7.32 (t, *J* = 7.5 Hz, 2H) CH 5′ and CH 7′, 7.22 (t, *J* = 7.3 Hz, 1H) CH 6′, 3.80 (t, *J* = 7.8 Hz, 2H) CH_2_ 1′, 3.33 (t, *J* = 7.8 Hz, 2H) CH_2_ 2′. ^13^C NMR (ppm, 400 MHz, DMSO) δ 152.1, 142.7, 141.3, 140.3, 134.9, 130.2, 129.2, 128.6, 128.3, 127.7, 126.3, 126.2, 116.8, 31.6, 29.5 (Section 2.4, Scheme 4 and Supporting Materials, S21).

*1-butyl-4-chloro-[1,2,4]triazolo[4,3-a]quinoxaline* (**15g**). C_13_H_13_ClN_4_. Mw: 260.72 g.mol^−1^. Chloranil (0.18 g, 0.723 mmol, 1 eq) was added to **14g** (0.190 g, 0.723 mmol, 1.0 eq) in CHCl_3_ (50 mL). The residue was purified through flash chromatography on silica gel (Cyclohexane/EtOAc 100:0 to 60:40) to afford the desired product as a white solid. Yield: 60%. tr: 2.83 min, MS (ESI+, QTof, *m*/*z*): 261.2 [M + H]^+^. ^1^H NMR (ppm, 400 MHz, DMSO-*d*_6_) δ 8.30 (d, *J* = 8.4 Hz, 1H) CH 9, 8.03 (dd, *J* = 8.0, 1.5 Hz, 1H) CH 6, 7.82 (td, *J* = 8.4, 1.1 Hz, 1H) CH 8, 7.72 (td, *J* = 8.0, 1.1 Hz, 1H) CH 7, 3.47 (t, *J* = 7.5 Hz, 2H) CH_2_ 1′, 1.93 (m, 2H) CH_2_ 2′, 1.52 (m, 2H) CH_2_ 3′, 0.98 (t, *J* = 7.4 Hz, 3H) CH_3_ 4′. ^13^C NMR (ppm, 100 MHz, DMSO-*d*_6_) δ 152.6, 142.6, 141.3, 134.9, 130.1, 129.2, 127.6, 126.3, 116.6, 27.9, 27.3, 21.6, 13.7 (Section 2.4, Scheme 4 and Supporting Materials, S22).

*4-chloro-1-isobutyl-[1,2,4]triazolo[4,3-a]quinoxaline* (**15h**). C_13_H_13_ClN_4_. Mw: 260.72 g·mol^−1^. Chloranil (0.59 g, 2.4 mmol, 1 eq) was added to **14h** (0.634 g, 2.4 mmol, 1.0 eq) in CHCl_3_ (50 mL). The residue was purified through flash chromatography on silica gel (Cyclohexane/EtOAc 100:0 to 50:50) to afford the desired product as a white solid. Yield: 69%. tr: 2.77 min, MS (ESI+, QTof, *m*/*z*): 261.2 [M + H]^+^. ^1^H NMR (ppm, 400 MHz, DMSO-*d*_6_) δ 8.27 (d, *J* = 8.1 Hz, 1H) CH 9, 8.03 (dd, *J* = 8.0, 1.4 Hz, 1H) CH 6, 7.83 (td, *J* = 8.6, 1.5 Hz, 1H) CH 8, 7.72 (td, *J* = 7.3, 0.9 Hz, 1H) CH 7, 3.36 (d, *J* = 7.0 Hz, 2H) CH_2_ 1′, 2.29–2.41 (m, 1H) CH 2′, 1.08 (s, 3H) CH_3_ 3′ or 4′, 1,07 (s, 3H) CH_3_ 3′ or 4′.^13^C NMR (ppm, 100 MHz, DMSO-*d*_6_) δ 151.8, 142.7, 141.4, 135.0, 130.2, 129.3, 127.7, 126.3, 116.6, 36.1, 25.8, 22.3 (Section 2.4, Scheme 4 and Supporting Materials, S23).

*4-chloro-1-methyl-[1,2,4]triazolo[4,3-a]quinoxaline* (**15i**). Chloranil (0.58 g, 2.36 mmol, 1 eq) was added to C_10_H_7_ClN_4_. Mw: 218.6 g·mol^−1^. To **14i** (0.434 g, 1.97 mmol, 1.0 eq) in CHCl_3_ (50 mL). The residue was purified through flash chromatography on silica gel (Cyclohexane/EtOAc 100:0 to 0:100) to afford the desired product as a brown solid. Yield: 86%. tr: 1.94 min, MS (ESI+, QTof, *m*/*z*): 219.1 [M + H]^+^. ^1^H NMR (ppm, 600 MHz, DMSO-*d*_6_) δ 8.35 (dd, *J* = 8.4, 1.0 Hz, 1H) CH 9, 8.02 (dd, *J* = 8.0, 1.5 Hz, 1H) CH 6, 7.80 (ddd, *J* = 8.5, 7.3, 1.6 Hz, 1H) CH 8, 7.71 (ddd, *J* = 8.5, 7.3, 1.6 Hz, 1H) CH 7, 3.10 (s, 3H) CH_3_. ^13^C NMR (ppm, 150 MHz, DMSO-*d*_6_) δ 149.5, 142.6, 141.2, 134.8, 130.1, 129.1, 127.7, 126.4, 116.5, 14.6 (Section 2.4, Scheme 4 and Supporting Materials, S24).

- **Procedure for the introduction of the methylamino group.**

**General procedure:**

A 33% (*w*/*v*) ethanol solution of methylamine was added to a solution of compound **15** in ethanol in a microwave-adapted vial. The reaction was submitted to microwave irradiation for 30 min at 150 °C. The solvent was removed under reduced pressure. The crude mixture was dissolved in ethyl acetate and washed 3 times with saturated aqueous sodium bicarbonate. The organic layer was dried on sodium sulphate, filtered, and concentrated under reduced pressure. The residue was purified through flash chromatography on silica gel to afford the desired product.

*1-(3,4-dimethoxyphenyl)-N-methyl-[1,2,4]triazolo[4,3-a]quinoxalin-4-amine* (**16a**). C_18_H_17_N_5_O_2_. Mw: 335.14 g·mol^−1^. A 33% (*w*/*v*) ethanol solution of methylamine (1.52 mL, 32 mmol, 20 eq) was added to a solution of **15a** (0.550 g, 1.61 mmol) in ethanol (15 mL) in a microwave-adapted vial. The residue was purified through flash chromatography on silica gel (Cyclohexane/EtOAc 100:0 to 0:100) to afford the desired product as a white solid. Yield: 91% (100% purity). tr: 2.48 min, MS (ESI+, QTof, *m*/*z*): 336.4 [M + H]^+^. HRMS for C_18_H_18_N_5_O_2,_ calculated 336.1455, found 336.1464. ^1^H NMR (ppm, 600 MHz, DMSO-*d*_6_) δ 8.27 (q, *J* = 4.6 Hz, 1H) NH, 7.62 (dd, *J* = 8.1, 1.3 Hz, 1H) CH 9, 7.37 (td, *J* = 6.9, 1.2 Hz, 1H) CH 8, 7.33 (d, *J* = 1.9 Hz, 1H) CH 2′, 7.29–7.26 (m, 2H) CH 6/6′, 7.21 (d, *J* = 8.3 Hz, 1H) CH 5′, 7.05 (ddd, *J* = 8.5, 7.4, 1.4 Hz, 1H) CH 7, 3.89 (s, 3H) OCH_3_, 3.76 (s, 3H) OCH_3_, 3.07 (d, *J* = 4.7 Hz, 3H) NHCH_3_.^13^C NMR (ppm, 150 MHz, DMSO-*d*_6_) δ 150.6, 150.0, 148.9, 146.5, 139.5, 137.9, 127.2, 126.5, 123.2, 122.9, 122.5, 120.3, 115.5, 113.3, 111.9, 55.8, 55.6, 27.4 (Section 2.4, Scheme 4 and Supporting Materials, S25).

*1-(3-methoxyphenyl)-N-methyl-[1,2,4]triazolo[4,3-a]quinoxalin-4-amine* (**16b**). C_17_H_15_N_5_O. Mw: 305.33 g·mol^−1^. A 33% (*w*/*v*) ethanol solution of methylamine (0.304 mL, 6.44 mmol, 20 eq) was added to a solution of **15b** (0.1 g, 0.322 mmol) in ethanol (12 mL) in a microwave-adapted vial. The desired product was obtained as a brown solid and used without further purification. Yield: 94% (98% purity). tr: 2.70 min, MS (ESI+, QTof, *m*/*z*): 306.3 [M + H]^+^. HRMS for C_17_H_16_N_5_O calculated 306.1349, found 306.1357. ^1^H NMR (ppm, 400 MHz, DMSO-*d*_6_) δ 8.28 (q, *J* = 4.5 Hz, 1H) NH, 7.62 (dd, *J* = 8.1, 0.9 Hz, 1H) CH 9, 7.58 (t, *J* = 7.9 Hz, 1H) CH 5′, 7.37 (td, *J* = 7.2, 1.0 Hz, 1H) CH 8, 7.33 (d, *J* = 1.8 Hz, 1H) CH 2′, 7.30 (d, *J* = 7.8 Hz, 1H) CH 6′, 7.27 (dd, *J* = 8.5, 2.4 Hz, 1H) CH 4′, 7.21 (dd, *J* = 8.3, 0.8 Hz, 1H) CH 6, 7.03 (td, *J* = 7.2, 1.3 Hz, 1H) CH 7, 3.82 (s, 3H) OCH_3_, 3.08 (d, *J* = 4.7 Hz, 3H) NHCH_3_. ^13^C NMR (ppm, 100 MHz, DMSO-*d*_6_) δ 159.9, 150.2, 146.9, 140.0, 138.4, 130.8, 130.2, 127.7, 127.0, 123.5, 122.9, 122.6, 117.1, 115.8, 115.7, 55.9, 27.8 (Section 2.4, Scheme 4 and Supporting Materials, S26).

*N-methyl-1-phenyl-[1,2,4]triazolo[4,3-a]quinoxalin-4-amine* (**16c**). C_16_H_13_N_5_. Mw: 275.31 g·mol^−1^. A 33% (*w*/*v*) ethanol solution of methylamine (0.51 mL, 11.6 mmol, 10 eq) was added to a solution of **15c** (0.326 g, 1.16 mmol) in ethanol (15 mL) in a microwave-adapted vial. The residue was purified through flash chromatography on silica gel (Cyclohexane/EtOAc 100:0 to 25:75) to afford the desired product as a green solid. Yield: 41%. tr: 2.58 min, MS (ESI+, QTof, *m*/*z*): 276.3 [M + H]^+^. HRMS for C_16_H_14_N_5_ calculated 276.1244, found 276.1246. ^1^H NMR (600 MHz, DMSO) δ 8.28 (q, *J* = 4.6 Hz, 1H) NH, 7.76 (d, *J* = 1.4 Hz, 1H) CH 2′ or 6′, 7.75 (d, *J* = 1.9 Hz, 1H) CH 2′ or 6′, 7.72 (t, *J* = 2.4 Hz, 1H) CH 4′, 7.68 (t, *J* = 1.7 Hz, 1H) CH 5′ or 3′, 7.67 (t, *J* = 1.6 Hz, 1H) CH 5′ or 3′, 7.63 (dd, *J* = 8.1, 1.4 Hz, 1H) CH 9, 7.37 (td, *J* = 7.3, 1.3 Hz, 1H) CH 8, 7.15 (dd, *J* = 8.4, 1.3 Hz, 1H) CH 6, 7.00 (td, *J* = 8.5, 1.5 Hz, 1H) CH 7, 3.08 (d, *J* = 4.8 Hz, 1H) NHCH_3_. ^13^C NMR (151 MHz, DMSO) δ 149.9, 146.4, 139.6, 138.0, 130.8, 130.0, 129.1, 128.5, 127.2, 126.6, 123.0, 122.3, 115.2, 27.33 (Section 2.4, Scheme 4 and Supporting Materials, S27).

*N-methyl-1-(3,4,5-trimethoxyphenyl)-[1,2,4]triazolo[4,3-a]quinoxalin-4-amine* (**16d**). C_19_H_19_N_5_O_3_. Mw: 365.39 g·mol^−1^. A 33% (*w*/*v*) ethanol solution of methylamine (1.09 mL, 23 mmol, 20 eq) was added to a solution of **15d** (0.426 g, 1.15 mmol) in ethanol (12 mL) in a microwave-adapted vial. The residue was purified through flash chromatography on silica gel (EtOAc/MeOH 100:0 to 97:3) to afford the desired product as a white solid. Yield: 30%. tr: 2.59 min, MS (ESI+, QTof, *m*/*z*): 366.4 [M + H]^+^. HRMS for C_19_H_20_N_5_O_3_ calculated 366.1561, found 366.1565. ^1^H NMR (600 MHz, DMSO) δ 8.28 (d, *J* = 3.4 Hz, 1H) NH, 7.63 (d, *J* = 7.6 Hz, 1H) CH 9, 7.39 (t, *J* = 7.4 Hz, 1H) CH 8, 7.28 (d, *J* = 8.1 Hz, 1H) CH 6, 7.12–7.05 (m, 3H) CH 7/2′/6′, 3.81 (s, 3H) OCH_3_, 3.79 (s, 6H) 2× OCH_3_, 3.07 (s, *J* = 3.2 Hz, 3H) NHCH_3_. ^13^C NMR (151 MHz, DMSO) δ 153.3, 149.9, 146.4, 139.4, 139.2, 138.0, 127.3, 126.5, 123.6, 123.1, 122.5, 115.62, 107.6, 60.3, 56.2, 27.4 (Section 2.4, Scheme 4 and Supporting Materials, S28).

*N-methyl-1-(pyridin-3-yl)-[1,2,4]triazolo[4,3-a]quinoxalin-4-amine* (**16e**). C_15_H_12_N_6_. Mw: 276.30 g·mol^−1^. A 33% (*w*/*v*) ethanol solution of methylamine (0.6 mL, 14 mmol, 20 eq) was added to a solution of **15e** (0.197 g, 0.70 mmol) in ethanol (12 mL) in a microwave-adapted vial. The residue was purified through flash chromatography on silica gel (EtOAc/MeOH 100:0 to 97:3) to afford the desired product as a yellow solid. Yield: 81%. tr: 1.91 min, MS (ESI+, QTof, *m*/*z*): 277.3 [M + H]^+^. HRMS for C_15_H_13_N_6_ calculated 277.1196, found 277.1205. ^1^H NMR (ppm, 600 MHz, DMSO-*d*_6_) δ 8.97 (dd, *J* = 2.1, 0.7 Hz, 1H) CH 2’, 8.90 (dd, *J* = 4.9, 1.7 Hz, 1H) CH 4′, 8.4 (s, 1H) NH, 8.27–8.24 (m, 1H) CH 6′, 7.72 (ddd, *J* = 7.9, 4.9, 0.7 Hz, 1H) CH 5’, 7.66 (dd, *J* = 8.1, 1.2 Hz, 1H) CH 9, 7.40 (td, *J* = 8.3, 1.4 Hz, 1H) CH 8, 7.14 (dd, *J* = 8.4, 1.2 Hz, 1H) CH 6, 7.06 (td, *J* = 8.3, 1.4 Hz, 1H) CH 7, 3.09 (d, *J* = 4.6 Hz, 3H) CH_3_. ^13^C NMR (ppm, 150 MHz, DMSO-*d*_6_) δ 151.6, 150.1, 147.6, 146.4, 139.9, 137.9, 137.8, 127.5, 126.5, 125.1, 123.9, 122.9, 122.7, 115.3, 27.5 (Section 2.4, Scheme 4 and Supporting Materials, S29).

*N-methyl-1-phenethyl-[1,2,4]triazolo[4,3-a]quinoxalin-4-amine* (**16f**). C_18_H_17_N_5_. Mw: 303.36 g·mol^−1^. A 33% (*w*/*v*) ethanol solution of methylamine (0.805 mL, 18 mmol, 20 eq) was added to a solution of **15f** (0.282 g, 0.91 mmol) in ethanol (15 mL) in a microwave-adapted vial. The desired product was obtained as a yellow solid and used without further purification. Yield: 91%. tr: 2.89 min, MS (ESI+, QTof, *m*/*z*): 304.3 [M + H]^+^. HRMS for C_18_H_18_N_5_ calculated 304.1557, found 304.1550. ^1^H NMR (ppm, 600 MHz, DMSO-*d*_6_) δ 8.15 (d, *J* = 3.9 Hz, 1H) NH, 8.07 (d, *J* = 8.1 Hz, 1H) CH 9, 7.64 (d, *J* = 7.9 Hz, 1H) CH 6, 7.45 (t, *J* = 7.7 Hz, 1H) CH 7, 7.38 (d, *J* = 7.3 Hz, 2H) CH 4′/8′, 7.34–7.30 (m, 3H) CH 8/5′/7′, 7.23 (t, *J* = 7.2 Hz, 1H) CH 6′, 3.72 (t, *J* = 7.9 Hz, 2H) CH_2_ 1′, 3.29 (t, *J* = 7.9 Hz, 2H) CH_2_ 2′, 3.04 (d, *J* = 4.3 Hz, 3H) CH_3_. ^13^C NMR (ppm, 150 MHz, DMSO-*d*_6_) δ 150.9, 146.4, 140.5, 139.7, 137.8, 128.4, 128.3, 127.0, 126.3, 126.1, 123.3, 122.9, 116.0, 31.84, 29.5, 27.2 (Section 2.4, Scheme 4 and Supporting Materials, S30).

*1-butyl-N-methyl-[1,2,4]triazolo[4,3-a]quinoxalin-4-amine* (**16g**). C_14_H_17_N_5_. Mw: 255.32 g·mol^−1^. A 33% (*w*/*v*) ethanol solution of methylamine (0.36 mL, 7.59 mmol, 20 eq) was added to a solution of **15g** (0.99 g, 0.38 mmol) in ethanol (12 mL) in a microwave-adapted vial. The desired product was obtained as a white solid and used without further purification. Yield: 88%. tr: 2.54 min, MS (ESI+, QTof, *m*/*z*): 256.3 [M + H]^+^. HRMS for C_14_H_18_N_5_ calculated 256.1557, found 256.1562. ^1^H NMR (ppm, 600 MHz, DMSO-*d*_6_) δ 8.12 (q, *J* = 4.5 Hz, 1H) NH, 8.04 (d, *J* = 7.7 Hz, 1H) CH 9, 7.63 (dd, *J* = 8.1, 1.3 Hz, 1H) CH 6, 7.45 (td, *J* = 7.6, 0.9 Hz, 1H) CH 7, 7.32 (td, *J* = 7.8, 1.4 Hz, 1H) CH 8, 3.40 (t, *J* = 7.4 Hz, 2H) CH2 1′, 3.03 (d, *J* = 4.8 Hz, 3H) NHCH_3_, 1.95–1.80 (m, 2H) CH_2_ 2′, 1.57–1.45 (m, 2H) CH_2_ 3′, 0.97 (t, *J* = 7.4 Hz, 3H) CH_3_ 4′, ^13^C NMR (ppm, 100 MHz, DMSO-*d*_6_) δ 151.6, 146.5, 139.7, 137.9, 127.01, 126.4, 123.4, 122.9, 115.9, 28.1, 27.4, 27.2, 21.74, 13.7 (Section 2.4, Scheme 4 and Supporting Materials, S31).

*1-isobutyl-N-methyl-[1,2,4]triazolo[4,3-a]quinoxalin-4-amine* (**16h**). C_14_H_17_N_5_. Mw: 255.33 g·mol^−1^. A 33% (*w*/*v*) ethanol solution of methylamine (1.5 mL, 31.9 mmol, 20 eq) was added to a solution of **15h** (0.416 g, 1.57 mmol) in ethanol (13 mL) in a microwave-adapted vial. The residue was purified through flash chromatography on silica gel (Cyclohexane/EtOAc 100:0 à 30:70) to afford the desired product as a white solid. Yield: 85%. tr: 2.48 min, MS (ESI+, QTof, *m*/*z*): 256.3 [M + H]^+^. HRMS for C_14_H_18_N_5_ calculated 256.1557, found 256.1562. ^1^H NMR (ppm, 400 MHz, DMSO-*d*_6_) δ 8.11 (q, *J* = 4.2 Hz, 1H) NH, 7.99 (d, *J* = 8.2 Hz, 1H) CH 9, 7.62 (dd, *J* = 8.1, 1.2 Hz, 1H) CH 6, 7.44 (t, *J* = 7.5 Hz, 1H) CH 7, 7.32 (*J* = 8.3, 1.3 Hz, 1H) CH 8, 3.28 (d, *J* = 7.0 Hz, 2H) CH_2_ 1′, 3.03 (d, *J* = 4.8 Hz, 3H) NHCH_3_, 2.31 (d, *J* = 13.2, 6.6 Hz, 1H) CH 2′, 1.06 (s, 3H) CH_3_ 3′ or 4′, 1.04 (s, 3H) CH_3_ 3′ or 4′. ^13^C NMR (ppm, 100 MHz, DMSO-*d*_6_) δ 150.7, 146.5, 139.7, 137.9, 127.0, 126.4, 123.4, 122.9, 115.9, 36.1, 27.2, 25.9, 22.3 (Section 2.4, Scheme 4 and Supporting Materials, S32).

*N,1-dimethyl-[1,2,4]triazolo[4,3-a]quinoxalin-4-amine* (**16i**). C_11_H_11_N_5_. Mw: 213.24 g·mol^−1^. A 33% (*w*/*v*) ethanol solution of methylamine (1.06 mL, 32 mmol, 20 eq) was added to a solution of **15i** (0.353 g, 1.61 mmol) in ethanol (14 mL) in a microwave-adapted vial. The residue was purified through flash chromatography on silica gel (EtOAc/MeOH 100:0 to 97:3) to afford the desired product as a white solid. Yield: 57%. tr: 1.62 min, MS (ESI+, QTof, *m*/*z*): 214.2 [M + H]^+^. HRMS for C_11_H_12_N_5_ calculated 214.1087, found 214.1095. ^1^H NMR (ppm, 600 MHz, DMSO-*d*_6_) δ 8.12 (d, *J* = 4.6 Hz, 1H) NH, 8.10 (dd, *J* = 8.3, 1.0 Hz, 1H) CH 9, 7.63 (dd, *J* = 8.1, 1.3 Hz, 1H) CH 6, 7.45 (td, *J* = 7.4, 1.2 Hz, 1H) CH 7, 7.31 (td, *J* = 8.5, 1.4 Hz, 1H) CH 8, 3.03 (s, 3H) CH_3_ NHCH_3_, 3.03 (s, 3H) CH_3_ 1′. ^13^C NMR (ppm, 150 MHz, DMSO-*d*_6_) δ 148.3, 146.4, 139.7, 137.8, 127.1, 126.2, 123.5, 122.9, 115.8, 27.3, 14.5 (Section 2.4, Scheme 4 and Supporting Materials, S33).

- **Procedure for the introduction of the amino group.**

*1-(3,4-dimethoxyphenyl)-[1,2,4]triazolo[4,3-a]quinoxalin-4-amine* (**16j**). C_17_H_15_N_5_O_2_. Mw: 321.34 g·mol^−1^. A 30% (*w*/*v*) ammoniac aqueous solution (2.13 mL, 16.3 mmol, 8 eq) was added to a solution of **15a** (0.695 g, 2.04 mmol) in acetonitrile (12 mL) in a microwave-adapted vial. The reaction was submitted to microwave irradiation for 2 h at 140 °C. The solvent was removed under reduced pressure. The crude mixture was dissolved in dichloromethane. The organic layer was washed with saturated aqueous NaCl, dried with Na_2_SO_4_, and concentrated to dryness under reduced pressure. The residue was purified through flash chromatography on silica gel (EtOAc/MeOH 100:0 to 97:3) to afford the desired product as a white solid. Yield: 59%. tr: 2.14 min, MS (ESI+, QTof, *m*/*z*): 322.2 [M + H]^+^. HRMS for C_17_H_16_N_5_O_2_ calculated 322.1299, found 322.1308. ^1^H NMR (ppm, 600 MHz, DMSO-*d*_6_) δ 7.58 (s, 2H) NH_2_, 7.55 (dd, *J* = 8.1, 1.1 Hz, 1H) CH 9, 7.37 (td, *J* = 7.3, 1.3 Hz, 1H) CH 8, 7.34 (d, *J* = 1.7 Hz, 1H) CH 2′, 7.30 (dd, *J* = 8.4, 1.2 Hz, 1H) CH 6′, 7.28 (d, *J* = 1.2 Hz, 1H) CH 6, 7.22 (d, *J* = 8.3 Hz, 1H) CH 5′, 7.06 (td, *J* = 7.2, 1.3 Hz, 1H) CH 7, 3.90 (s, 3H) OCH_3_ 7′ ou 8′, 3.77 (s, 3H) OCH_3_ 7′ ou 8′. ^13^C NMR (ppm, 150 MHz, DMSO-*d*_6_) δ 150.6, 149.9, 148.9, 147.5, 139.2, 137.9, 127.1, 125.9, 123.5, 122.8, 122.5, 120.4, 115.4, 113.4, 111.9, 55.7, 55.6 (Section 2.4, Scheme 4 and Supporting Materials, S34).

- Procedure for the cleavage of catechol protections.

*4-(4-(methylamino)-[1,2,4]triazolo[4,3-a]quinoxalin-1-yl)benzene-1,2-diol* (**17a**). C_16_H_13_N_5_O_2_. Mw: 307.31 g·mol^−1^. After solubilization of **16a** (0.479 g, 1.43 mmol, 1 eq) in dichloromethane, boron tribromide (5.71 mL, 5.71 mmol, 2 eq/protecting group) was slowly added at 0 °C. The reaction was allowed to warm up to room temperature and stirred for 2 h. Methanol was added to quench the reaction, which was then concentrated under reduced pressure. This process was repeated four times. To neutralize the HBr salt, the crude mixture was stirred with Amberlyst A21 resin at 50 °C for 30 min. The supernatant was recovered, and the resin was washed 3 times with MeOH. The crude mixture was purified by preparative HPLC eluted with H_2_O/ACN to yield the desired product as a white solid. Yield: 6.6% (purity 96%). tr: 1.95 min, MS (ESI+, QTof, *m*/*z*): 308.3 [M + H]^+^. HRMS for C_16_H_14_N_5_O_2_ calculated 308.1142, found 308.1146. ^1^H NMR (ppm, 600 MHz, DMSO-*d*_6_) δ 8.89 (s, 1H) NH, 7.69 (d, *J* = 8.0 Hz, 1H) CH 9, 7.41 (t, *J* = 7.7 Hz, 1H) CH 8, 7.34 (d, *J* = 7.8 Hz, 1H) CH 6′, 7.12 (t, *J* = 7.6 Hz, 1H) CH 7, 7.04 (d, *J* = 1.6 Hz, 1H) CH 2′, 7.00–6.96 (m, 2H) CH 5′/6, 3.12 (d, *J* = 3.7 Hz, 3H) NHCH_3_. ^13^C NMR (ppm, 150 MHz, DMSO-*d*_6_) δ 150.7, 147.9, 146.2, 145.8, 139.5, 127.4, 124.8, 123.1, 122.9, 121.4, 118.4, 116.9, 116.1, 115.6, 27.9 (Section 2.4, Scheme 4 and Supporting Materials, S35).

### 3.2. Cell lines and Culture Techniques

#### 3.2.1. General

The A375 melanoma human cancer cell line was obtained from American Type Culture Collection (Rockville, MD, USA). Cells were cultured in DMEM medium (Pan Biotech, Aidenbach, Germany) containing 10% heat-inactived (56 °C) fetal bovine serum (FBS) (Pan Biotech, Germany), 100 U/mL penicillin, and 100 µg/mL streptomycin (Pan Biotech, Germany). Cells were maintained in a humidified atmosphere of 5% CO_2_ in air at 37 °C.

#### 3.2.2. In Vitro Cytotoxicity Assay

Prior to the experiments, the number of cells by well, the doubling time, and the MTT concentration were optimized. In all the experiments, A375 cells were seeded at a final concentration of 3500 cells/well in 96-well microtiter plates and allowed to attach overnight. Each evaluated compound, whose purity (determined by UPLC) as equal or superior to 95%, was diluted in DMSO to obtain a stock solution at 10^−2^ M. Cells were exposed (i) to vehicle controls (0.15% DMSO/culture medium (*v*/*v*)) and (ii) to the synthesized compounds (**4**, **8a**–**d**, **16a**–**j**, **17a**) at a concentration range from 10^−5^ to 4.10^−11^ M dissolved in a culture medium (final concentration of 0.15% DMSO). Each treatment was performed in triplicate. After 96 h of incubation, the cell culture medium was removed, 100 μL per well of a 0.5 mg/mL MTT (3-[4,5-dimethylthiazol-2-yl]-2,5-diphenyltetrazolium bromide) solution in fresh medium was added, and plates were incubated for 4 h at 37 °C. This colorimetric assay is based on the ability of live and metabolically unimpaired cells to reduce MTT to a blue formazan product. At the end of the incubation period, 100 µL of 10% SDS/0.01 M hydrochloric acid solution was added to each well. After overnight incubation at 37 °C and vigorous shaking to solubilize formazan crystals, the optical density was measured at 570 nm in a microculture plate reader (PowerWave HT). Cell survival was expressed as a percent of the vehicle control. Live cells percent = (measured specific activity − mean positive control specific activity)/(mean negative control activity − mean positive control specific activity) × 100. The negative control corresponded to 0.15% DMSO treatment, and for the positive control, 1% SDS was added to control wells 15 min before the addition of MTT. EC_50_ values were determined by a non-linear regression analysis of the inhibition curve generated by mean replicate values. The analysis was performed using GraphPad Prism software (version 9.2.0). The EC_50_ values defined the concentrations of drugs required to obtain 50% of the maximum effect on cell growth.

## 4. Conclusions

We have enlarged the Imiqualine family by designing novel compounds around the [1,2,4]triazolo[4,3-*a*]quinoxaline scaffold. Position 1 was substituted by various moieties corresponding to ones of our best hits and led through a general synthetic pathway using 1-chloro-2-hydrazinoquinoxaline and an appropriate aldehyde. Cyclization was ensured by an oxidation-reduction mechanism using chloranil. Position 8 was also substituted via another strategy due to unexpected bromination. These novel compounds were evaluated on the A375 melanoma cell line. Compound **17a** (EAPB42303) displayed an interesting activity and became the hit of the novel [1,2,4]triazolo[4,3-*a*]quinoxaline series.

## Figures and Tables

**Table 1:** Predictive physicochemical parameters of prepared compounds.

Compounds	Overall Yields	pKa1	pKa2	LogP	PSA	LogD (at pH 5.6)	LogS (mol/L) (at pH 5.6)	Aqueous Solubility (mg/mL at pH 7)
**EAPB02303**	-	4.32	-	3.55	82.68	3.50	−4.26	1.70 × 10^−2^
**4**	85%	2.09	−4.03	2	55.11	2	−3.21	12.0 × 10^−2^
**8a**	2%	1.94	−4.23	2.86	73.57	2.85	−4.34	1.52 × 10^−2^
**8b**	6%	1.91	−4.24	3.53	64.34	3.50	−4.62	7.23 × 10^−3^
**8c**	7%	1.98	−4.17	3.91	55.11	3.65	−4.76	4.84 × 10^−3^
**8d**	50%	1.81	−4.34	2.07	82.80	2.05	−4.00	3.64 × 10^−2^
**16a**	59%	1.58	−4.04	4.74	73.57	4.75	−5.36	1.45 × 10^−3^
**16b**	60%	1.56	−4.11	5.23	64.34	5.25	−5.55	8.67 × 10^−4^
**16c**	29%	1.74	−4.06	5.24	55.11	5.25	−5.47	9.23 × 10^−4^
**16d**	14%	1.31	−4.21	4.33	82.80	4.30	−5.23	2.17 × 10^−3^
**16e**	24%	2.51	−0.34	3.83	68.00	3.80	−4.60	6.98 × 10^−3^
**16f**	37%	2.49	−3.77	3.37	55.11	3.30	−4.50	9.50 × 10^−3^
**16g**	17%	2.55	−3.69	3.22	55.11	3.25	−3.96	2.79 × 10^−2^
**16h**	57%	2.49	−3.77	3.06	55.11	3.10	−3.88	3.35 × 10^−2^
**16i**	39%	2.50	−3.67	1.69	55.11	1.70	−3.06	19.0 × 10^−2^
**16j**	46%	1.29	−4.26	3.58	87.56	3.55	−4.77	5.47 × 10^−3^
**17a**	4%	1.31	−4.26	3.91	95.57	3.90	−4.65	7.07 × 10^−3^

**Table 2:** A375 cell viability at 10 µM for compounds **4**, **8a**–**d**, **16a**–**j**, **17a**.

Compounds	Imiqualines References	Cell Viability at 10 µM (%)
**4**	EAPB4003	6.0 ± 1.2
**8a**	EAPB4003-8j	96.9 ± 8.1
**8b**	EAPB4003-8k	95.0 ± 3.4
**8c**	EAPB4003-8l	50.7 ± 2.2
**8d**	EAPB4003-8m	90.6 ± 4.8
**16a**	EAPB42203	2.9 ± 0.1
**16b**	EAPB4503	11.3 ± 6.9
**16c**	EAPB4403	46.7 ± 2.1
**16d**	EAPB42703	41.5 ± 0.8
**16e**	EAPB43803	56.8 ± 7.0
**16f**	EAPB4203	33.5 ± 13.3
**16g**	EAPB43603	76.6 ± 16.5
**16h**	EAPB4103	66.2 ± 5.9
**16i**	EAPB4303	85.4 ± 13.3
**16j**	EAPB42203	98.6 ± 11.58
**17a**	EAPB42303	5.5 ± 1.2

**Table 3:** EC_50_ on A375 melanoma cell line for compounds **16a**, **16b**, and **17a**.

Compounds	Imiqualines References	EC_50_ (nM)
	EAPB0503 [53]	383
	EAPB02303 [53]	3
**16a**	EAPB42203	3158
**16b**	EAPB4503	3527
**17a**	EAPB42303	365

## Data Availability

Not applicable.

## Supplementary Materials

The following supporting information can be downloaded at: https://www.mdpi.com/article/10.3390/molecules28145478/s1, S1: N-methyl-[1,2,4]triazolo[4,3-*a*]quinoxalin-4-yl-amine (**4**), S2: 8-Bromo-[1,2,4]triazolo[4,3-*a*]quinoxalin-4-yl)-methyl-amine (**7**), S3: [8-(3,4-Dimethoxy-phenyl)-[1,2,4]triazolo[4,3-*a*]quinoxalin-4-yl]-methyl-amine (**8a**), S4: [8-(3-Methoxy-phenyl)-[1,2,4]triazolo[4,3-*a*]quinoxalin-4-yl]-methyl-amine (**8b**), S5: Methyl-(8-phenyl-[1,2,4]triazolo[4,3-*a*]quinoxalin-4-yl)-amine (**8c**), S6: Methyl-[8-(3,4,5-trimethoxy-phenyl)-[1,2,4]triazolo[4,3-*a*]quinoxalin-4-yl]-amine (**8d**), S7: 2-chloro-3-(2-(3,4-dimethoxybenzylidene)hydrazinyl)quinoxaline (**14a**), S8: 2-chloro-3-(2-(3-methoxybenzylidene)hydrazinyl)quinoxaline (**14b**), S9: 2-(2-benzylidenehydrazinyl)-3-chloroquinoxaline (**14c**), S10: 2-chloro-3-(2-(3,4,5-trimethoxybenzylidene)hydrazinyl)quinoxaline (**14d**), S11: 2-chloro-3-(2-(pyridin-3-ylmethylene)hydrazinyl)quinoxaline (**14e**), S12: 2-chloro-3-(2-(3-phenylpropylidene)hydrazinyl)quinoxaline (**14f**), S13: 2-chloro-3-(2-pentylidenehydrazinyl)quinoxaline (**14g**), S14: 2-chloro-3-(2-(3-methylbutylidene)hydrazinyl)quinoxaline (**14h**), S15: 2-chloro-3-(2-ethylidenehydrazinyl)quinoxaline (**14i**), S16: 4-chloro-1-(3,4-dimethoxyphenyl)-[1,2,4]triazolo[4,3-*a*]quinoxaline (**15a**), S17: 4-chloro-1-(3-methoxyphenyl)-[1,2,4]triazolo[4,3-*a*]quinoxaline (**15b**), S18: 4-chloro-1-phenyl-[1,2,4]triazolo[4,3-*a*]quinoxaline (**15c**), S19: 4-chloro-1-(3,4,5-trimethoxyphenyl)-[1,2,4]triazolo[4,3-*a*]quinoxaline (**15d**), S20: 4-chloro-1-(pyridin-3-yl)-[1,2,4]triazolo[4,3-*a*]quinoxaline (**15e**), S21: 4-chloro-1-phenethyl-[1,2,4]triazolo[4,3-a]quinoxaline (**15f**), S22: 1-butyl-4-chloro-[1,2,4]triazolo[4,3-a]quinoxaline (**15g**), S23: 4-chloro-1-isobutyl-[1,2,4]triazolo[4,3-a]quinoxaline (**15h**), S24: 4-chloro-1-methyl-[1,2,4]triazolo[4,3-a]quinoxaline (**15i**), S25: 1-(3,4-dimethoxyphenyl)-N-methyl-[1,2,4]triazolo[4,3-*a*]quinoxalin-4-amine (**16a**), S26: 1-(3-methoxyphenyl)-N-methyl-[1,2,4]triazolo[4,3-*a*]quinoxalin-4-amine (**16b**), S27: N-methyl-1-phenyl-[1,2,4]triazolo[4,3-*a*]quinoxalin-4-amine (**16c**), S28: N-methyl-1-(3,4,5-trimethoxyphenyl)-[1,2,4]triazolo[4,3-*a*]quinoxalin-4-amine (**16d**), S29: N-methyl-1-(pyridin-3-yl)-[1,2,4]triazolo[4,3-*a*]quinoxalin-4-amine (**16e**), S30: N-methyl-1-phenethyl-[1,2,4]triazolo[4,3-a]quinoxalin-4-amine (**16f**), S31: 1-butyl-N-methyl-[1,2,4]triazolo[4,3-*a*]quinoxalin-4-amine (16g), S32: 1-isobutyl-N-methyl-[1,2,4]triazolo[4,3*-a*]quinoxalin-4-amine (**16h**), S33: N,1-dimethyl-[1,2,4]triazolo[4,3-*a*]quinoxalin-4-amine (**16i**), S34: 1-(3,4-dimethoxyphenyl)-[1,2,4]triazolo[4,3-*a*]quinoxalin-4-amine (**16j**), S35: 4-(4-(methylamino)-[1,2,4]triazolo[4,3-*a*]quinoxalin-1-yl)benzene-1,2-diol (**17a**). MR spectra, HPLC/MS and HRMS data are available in the Supplementary Materials.

## Sample Availability

Samples of the compounds are available from the authors.

## Abbreviations

MAPK	Mitogen-activated protein kinases
BRAF	B-rapidly accelerated fibrosarcoma
NRAS	Neuroblastoma RAS viral oncogene
NF1	Neurofibromatosis-1
MEK	Mitogen-activated protein kinase kinase
PD-1	Programmed cell death protein-1
CTLA-4	Cytotoxique T lymphocyte antigen-4 protein
VEGFR-2	Vascular endothelial growth factor receptor-2
BAX	Bcl-2-associated X protein
hPBMCs	Human peripheral blood mononuclear cells
hERG	Human ether-à-go-go-related gene
SILAC	Stable isotope labeling by amino acids in cell culture
logS	Aqueous solubility
logP	Partition coefficient
logD	Distribution coefficient
pKa	Ionization constant
MW	Micro-wave
NBS	N-bromosuccinimide
NMR	Nuclear magnetic resonance
NOESY	Nuclear Overhauser effect epectroscopy
EtOH	Ethanol
ACN	Acetonitrile
RT	Room temperature
DME	Dimethoxyethane
DMF	Dimethylformamide
2D NOESY	Two-dimensional nuclear Overhauser effect spectroscopy
ADME	Absorption distribution metabolism elimination
PSA	Polar surface area
SAR	Structure–activity relationship
EC_50_	Half-maximal effective concentration
TLC	Thin-layer chromatography
UV	Ultra-violet
LC-MS	Liquid chromatography mass spectroscopy
ESI	Electrospray ionization
UPLC/MS	Ultra-performance liquid chromatography/mass spectroscopy
Da	Dalton
HPLC	High-performance liquid chromatography
HRMS	High-resolution mass spectroscopy
V	Volt
ppm	Parts per million
DMSO	Dimethylsulfoxide
s	Singlet
d	Doublet
t	Triplet
q	Quartet
m	Multiplet
Mw	Molecular weight
MeOH	Methanol
EtOAc	Ethyl acetate
DMEM	Dulbecco’s Modified Eagle Medium
FBS	Fetal bovine serum
MTT	Thiazolyl blue tetrazolium bromide
SDS	Sodium dodecyl sulfate
MDA-MB-231	Human triple-negative breast adenocarcinoma cell line
HepG2	Hepatocellular carcinoma (HCC) type
HCT116	Human colorectal carcinoma-116
MCF-7	Michigan Cancer Foundation-7 breast cancer
DNA	Desoxyribonucleic acid
HL60	Human acute myelocytic leukemia
