# Supplementary material for: [1,2,4]triazolo[4,3-a]quinoxaline as Novel Scaffold in the Imiqualines Family: Candidates with Cytotoxic Activities on Melanoma Cell Lines
Source: Molecules. 2023 Jul 18;28(14):5478. doi: 10.3390/molecules28145478 (PMC10384284; doi:10.3390/molecules28145478)
Supplement: Supplementary file 1 [file molecules-28-05478-s001.zip › molecules-2500945-supplementary.pdf]

molecules

MDPI

Supporting Materials

Article

[1,2,4]triazolo[4,3-*a*]quinoxaline as novel scaffold in the Imiqualines family : candidates with cytotoxic activities on melanoma cell lines.

Cindy Patinote [1], Sandy Raevens [1], Amélie Baumann [1], Eloise Pellegrin [1], Carine Deleuze-Masquéfa [1], Pierre-Antoine Bonnet [1,*].

1 Institut des Biomolécules Max Mousseron (IBMM), UMR 5247 F16, (CNRS, ENSCM, Université de Montpellier), 1919 route de Mende, 34090 Montpellier, France. cindy.patinote@umontpellier.fr, carine.masquefa@umontpellier.fr
* Correspondence: pierre-antoine.bonnet@umontpellier.fr

Compounds descriptions

S1. N-methyl-[1,2,4]triazolo[4,3-a]quinoxalin-4-yl-amine (**4**)....... 3
S2. 8-Bromo-[1,2,4]triazolo[4,3-a]quinoxalin-4-yl)-methyl-amine (**7**)....... 5
S3. [8-(3,4-Dimethoxy-phenyl)-[1,2,4]triazolo[4,3-a]quinoxalin-4-yl]-methyl-amine (**8a**)....... 8
S4. [8-(3-Methoxy-phenyl)-[1,2,4]triazolo[4,3-a]quinoxalin-4-yl]-methyl-amine (**8b**)....... 10
S5. Methyl-(8-phenyl-[1,2,4]triazolo[4,3-a]quinoxalin-4-yl)-amine (**8c**)....... 13
S6. Methyl-[8-(3,4,5-trimethoxy-phenyl)-[1,2,4]triazolo[4,3-a]quinoxalin-4-yl]-amine (**8d**)....... 15
S7. 2-chloro-3-(2-(3,4-dimethoxybenzylidene)hydrazinyl)quinoxaline (**14a**)....... 17
S8. 2-chloro-3-(2-(3-methoxybenzylidene)hydrazinyl)quinoxaline (**14b**)....... 19
S9. 2-(2-benzylidenehydrazinyl)-3-chloroquinoxaline (**14c**)....... 21
S10. 2-chloro-3-(2-(3,4,5-trimethoxybenzylidene)hydrazinyl)quinoxaline (**14d**)....... 23
S11. 2-chloro-3-(2-(pyridin-3-ylmethylene)hydrazinyl)quinoxaline (**14e**)....... 25
S12. 2-chloro-3-(2-(3-phenylpropylidene)hydrazinyl)quinoxaline (**14f**)....... 27
S13. 2-chloro-3-(2-pentylidenehydrazinyl)quinoxaline (**14g**)....... 29
S14. 2-chloro-3-(2-(3-methylbutylidene)hydrazinyl)quinoxaline (**14h**)....... 31
S15. 2-chloro-3-(2-ethylidenehydrazinyl)quinoxaline (**14i**)....... 33
S16. 4-chloro-1-(3,4-dimethoxyphenyl)-[1,2,4]triazolo[4,3-a]quinoxaline (**15a**)....... 34
S17. 4-chloro-1-(3-methoxyphenyl)-[1,2,4]triazolo[4,3-a]quinoxaline (**15b**)....... 36
S18. 4-chloro-1-phenyl-[1,2,4]triazolo[4,3-a]quinoxaline (**15c**)....... 38
S19. 4-chloro-1-(3,4,5-trimethoxyphenyl)-[1,2,4]triazolo[4,3-a]quinoxaline (**15d**)....... 40

S20. 4-chloro-1-(pyridin-3-yl)-[1,2,4]triazolo[4,3-a]quinoxaline (**15e**). 42
S21. 4-chloro-1-phenethyl-[1,2,4]triazolo[4,3-a]quinoxaline (**15f**). 44
S22. 1-butyl-4-chloro-[1,2,4]triazolo[4,3-a]quinoxaline (**15g**). 46
S23. 4-chloro-1-isobutyl-[1,2,4]triazolo[4,3-a]quinoxaline (**15h**). 48
S24. 4-chloro-1-methyl-[1,2,4]triazolo[4,3-a]quinoxaline (**15i**). 50
S25. 1-(3,4-dimethoxyphenyl)-N-methyl-[1,2,4]triazolo[4,3-a]quinoxalin-4-amine (**16a**). 52
S26. 1-(3-methoxyphenyl)-N-methyl-[1,2,4]triazolo[4,3-a]quinoxalin-4-amine (**16b**). 56
S27. N-methyl-1-phenyl-[1,2,4]triazolo[4,3-a]quinoxalin-4-amine (**16c**). 59
S28. N-methyl-1-(3,4,5-trimethoxyphenyl)-[1,2,4]triazolo[4,3-a]quinoxalin-4-amine (**16d**). 62
S29. N-methyl-1-(pyridin-3-yl)-[1,2,4]triazolo[4,3-a]quinoxalin-4-amine (**16e**). 65
S30. N-methyl-1-phenethyl-[1,2,4]triazolo[4,3-a]quinoxalin-4-amine (**16f**). 68
S31. 1-butyl-N-methyl-[1,2,4]triazolo[4,3-a]quinoxalin-4-amine (**16g**). 71
S32. 1-isobutyl-N-methyl-[1,2,4]triazolo[4,3-a]quinoxalin-4-amine (**16h**). 74
S33. N,1-dimethyl-[1,2,4]triazolo[4,3-a]quinoxalin-4-amine (**16i**). 77
S34. 1-(3,4-dimethoxyphenyl)-[1,2,4]triazolo[4,3-a]quinoxalin-4-amine (**16j**). 80
S35. 4-(4-(methylamino)-[1,2,4]triazolo[4,3-a]quinoxalin-1-yl)benzene-1,2-diol (**17a**). 83

*S1. N-methyl-[1,2,4]triazolo[4,3-a]quinoxalin-4-yl-amine (**4**).*

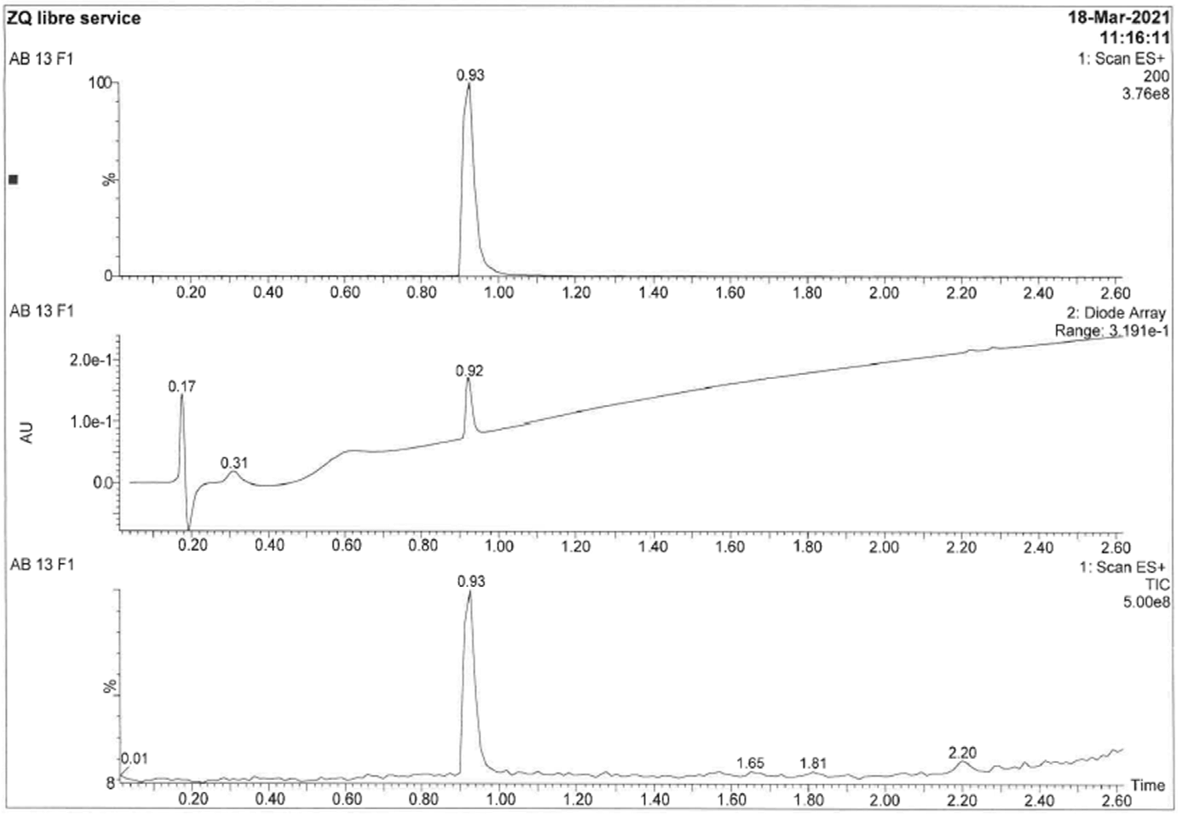

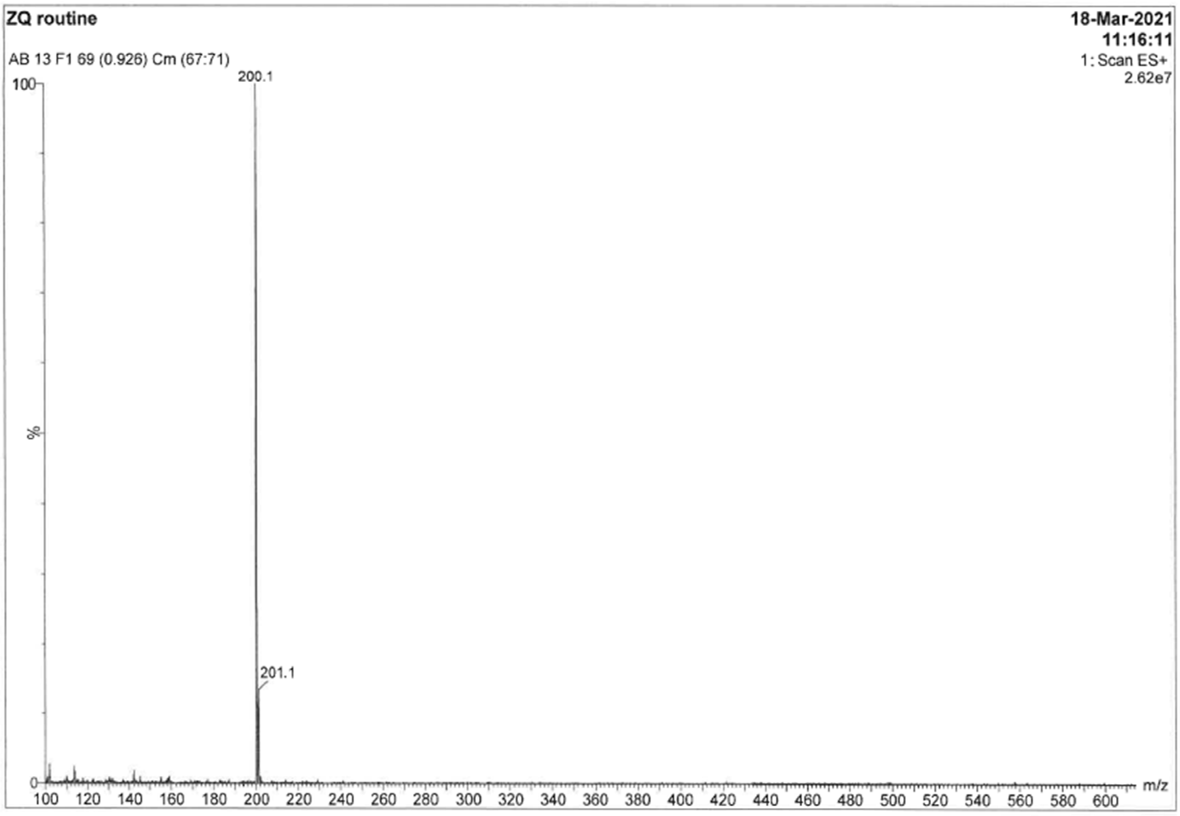

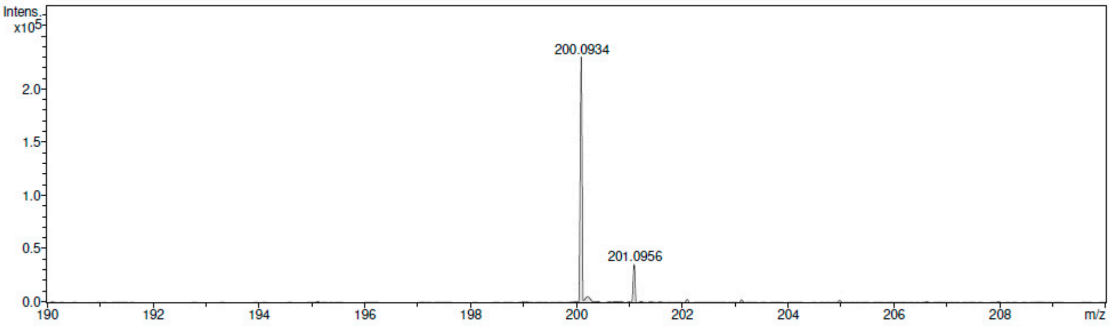

Meas. m/z	#	Ion Formula	m/z	err [ppm]	mSigma	# Sigma	Score	rdb	e⁻ Conf	N-Rule
200.0934	1	C10H10N5	200.0931	1.5	16.3	1	100.00	8.5	even	ok

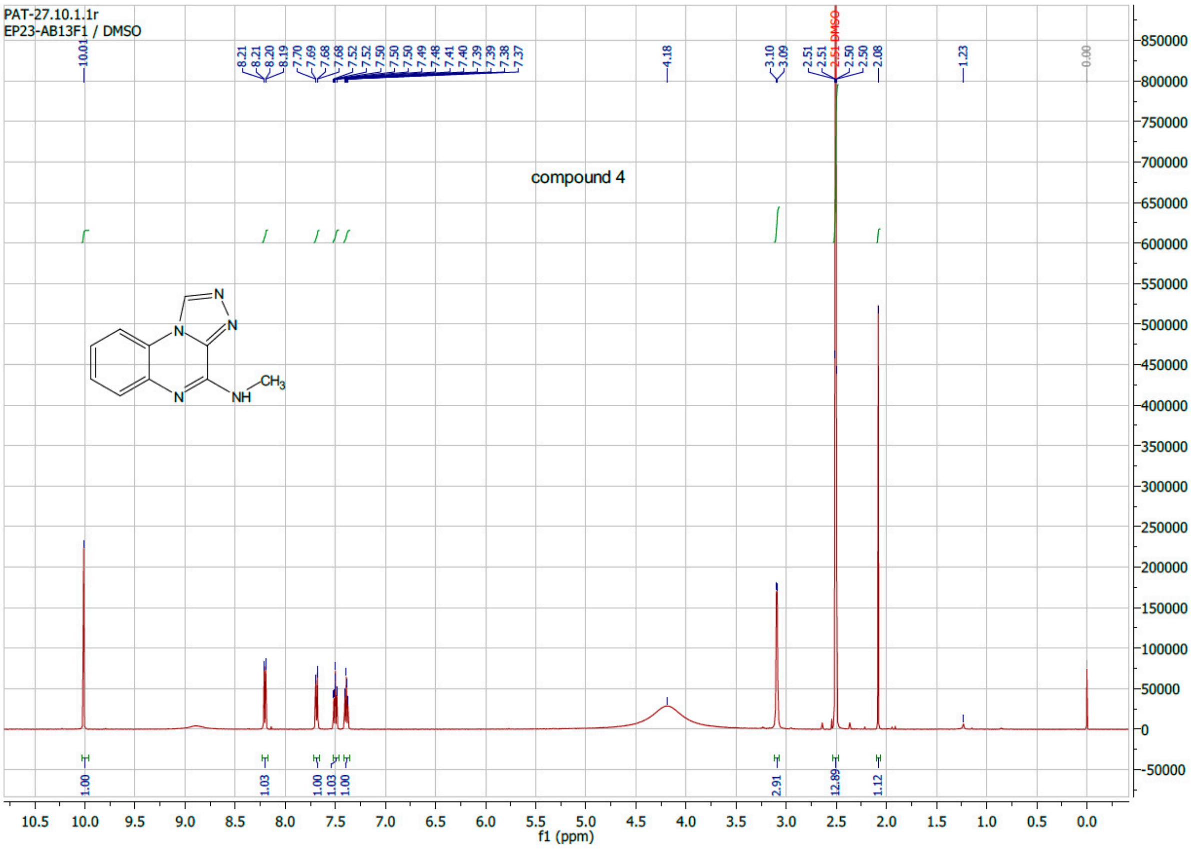

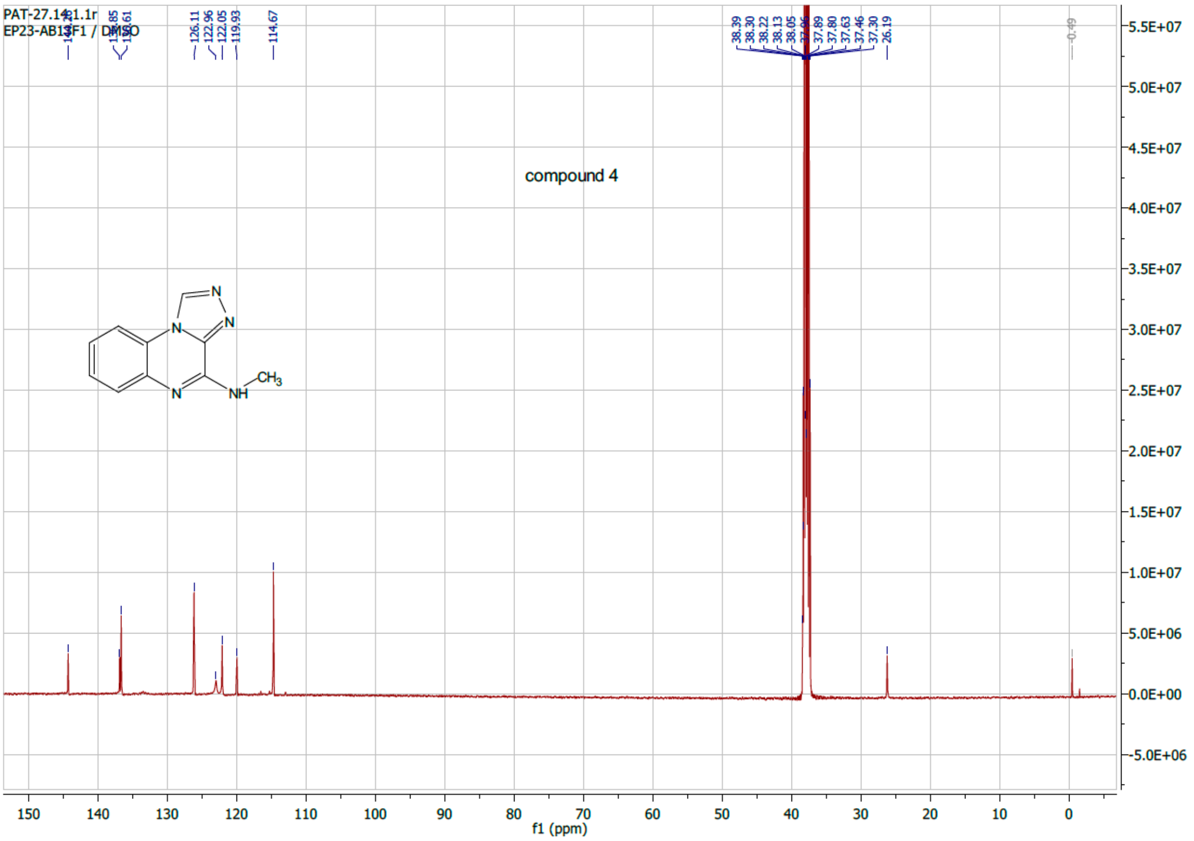

*S2. 8-Bromo-[1,2,4]triazolo[4,3-a]quinoxalin-4-yl)-methyl-amine (**7**).*

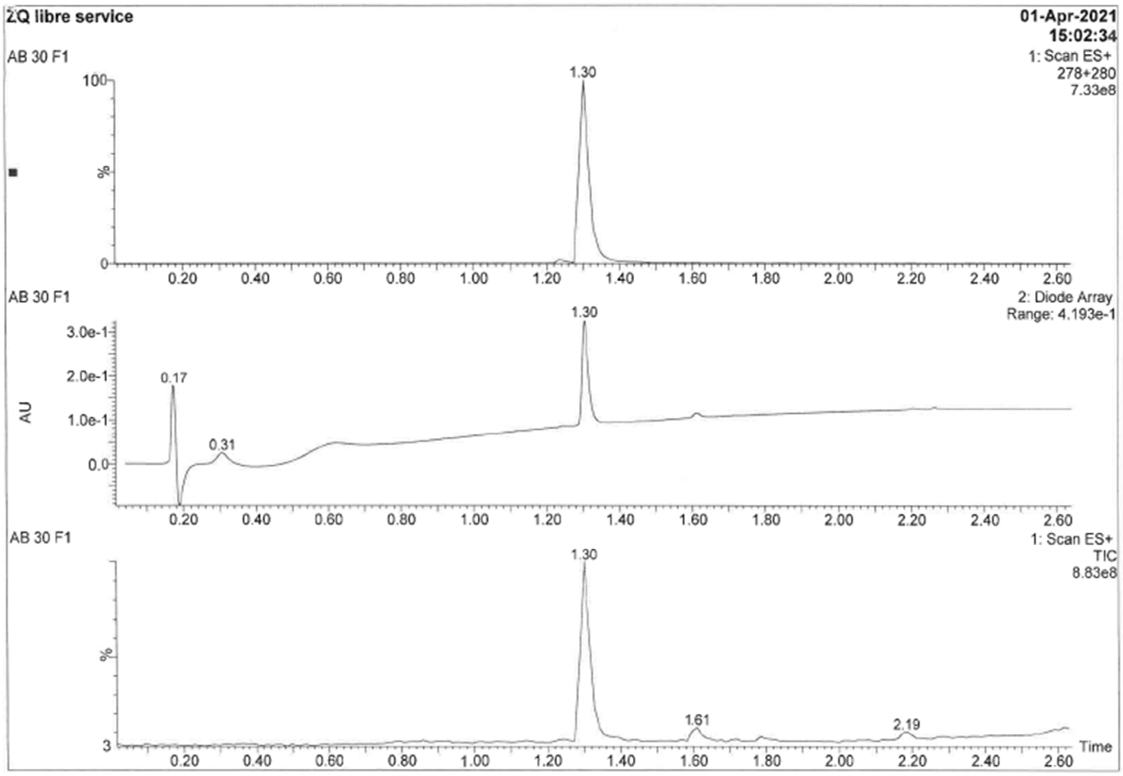

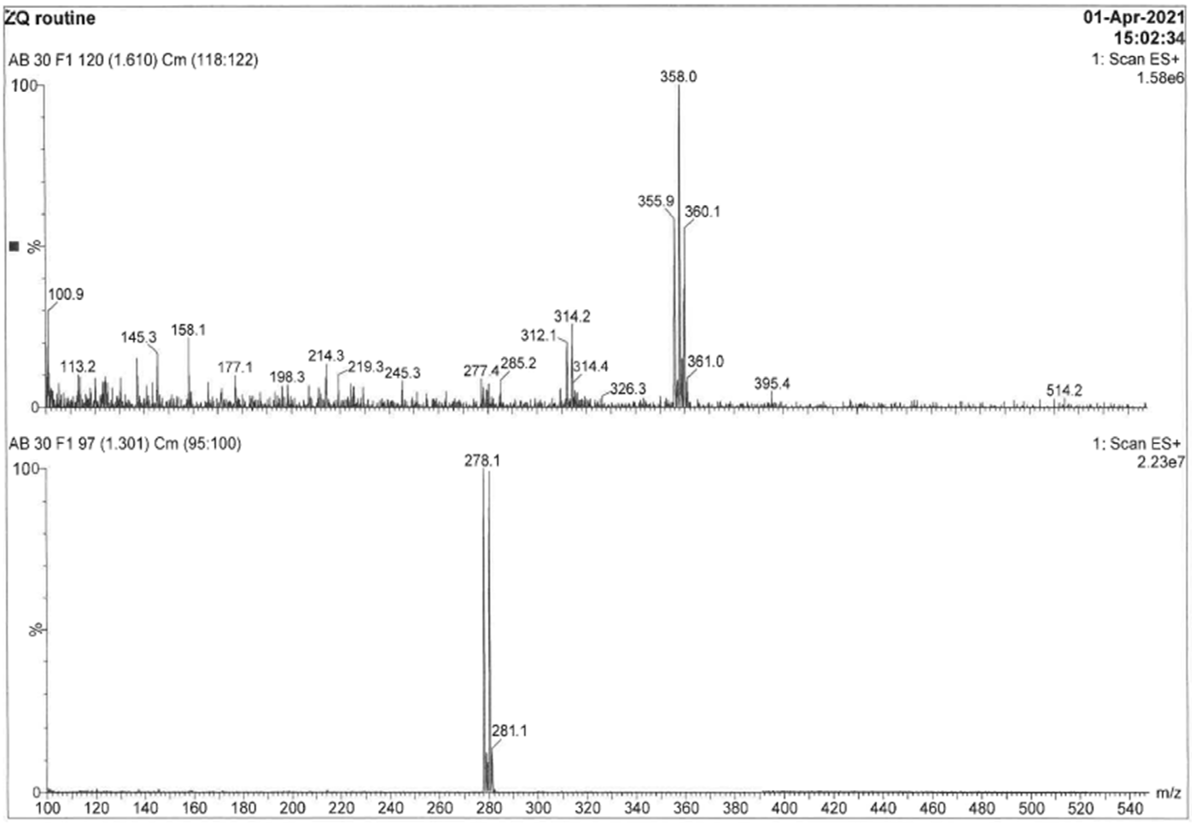
ZQ routine
01-Apr-2021
15:02:34
AB 30 F1 120 (1.610) Cm (118:122)
1: Scan ES+
1.58e6
358.0
355.9
360.1
100.9
314.2
312.1
145.3
158.1
113.2
177.1
214.3
198.3
219.3
245.3
277.4
285.2
314.4
361.0
326.3
395.4
514.2
AB 30 F1 97 (1.301) Cm (95:100)
1: Scan ES+
2.23e7
278.1
281.1
m/z

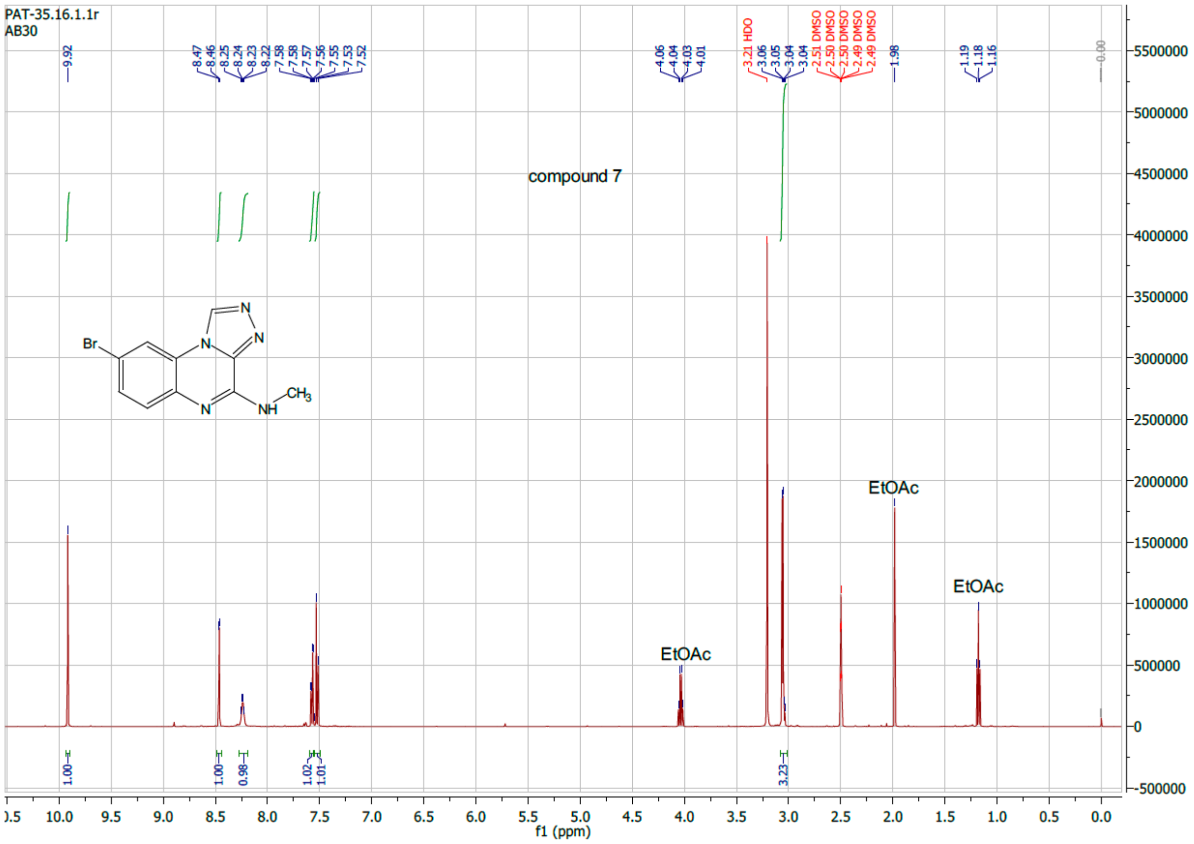
PAT-35.16.1.1r
AB30
compound 7
EtOAc
EtOAc
EtOAc
3.21 HDO
2.51 DMSO
2.50 DMSO
2.50 DMSO
2.49 DMSO
2.49 DMSO
Br
N
N
N
N
NH
CH3
f1 (ppm)

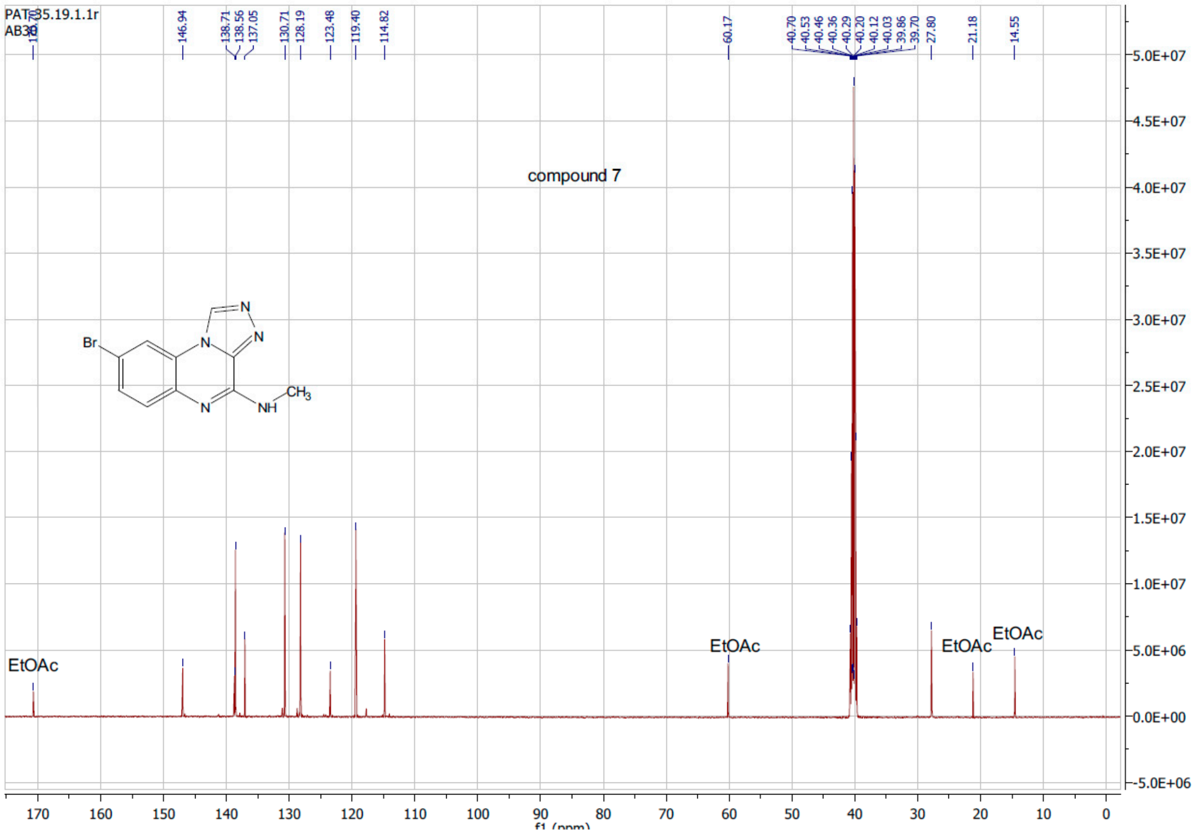
PAT-35.19.1.1r
AB30
146.94
138.71
138.56
137.05
130.71
128.19
123.48
119.40
114.82
60.17
40.70
40.53
40.46
40.36
40.29
40.20
40.12
40.03
39.86
39.70
27.80
21.18
14.55
compound 7
EtOAc
EtOAc
EtOAc
EtOAc
Br
N
N
N
N
NH
CH3
f1 (ppm)

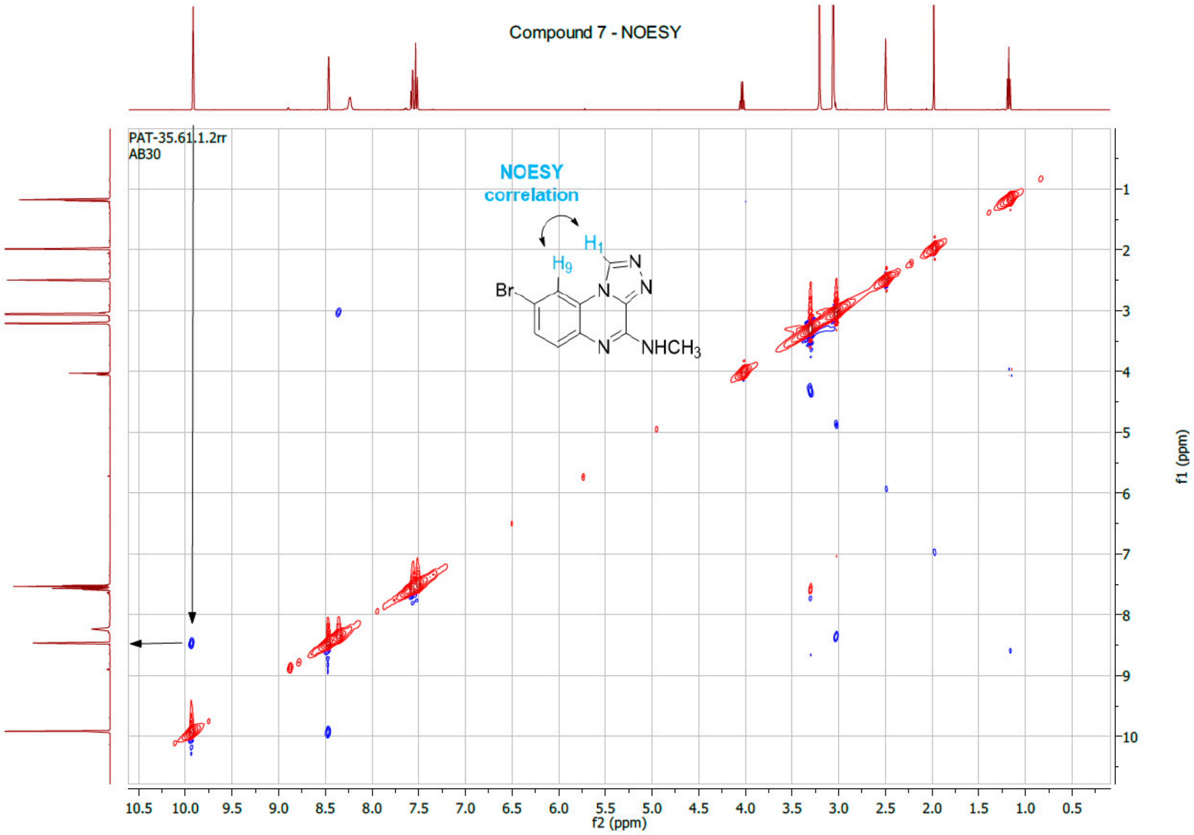
Compound 7 - NOESY
PAT-35.61.1.2rr
AB30
NOESY
correlation
H1
H9
Br
N
N
N
N
NHCH3
f2 (ppm)
f1 (ppm)

*S3. [8-(3,4-Dimethoxy-phenyl)-[1,2,4]triazolo[4,3-a]quinoxalin-4-yl]-methyl-amine (**8a**).*

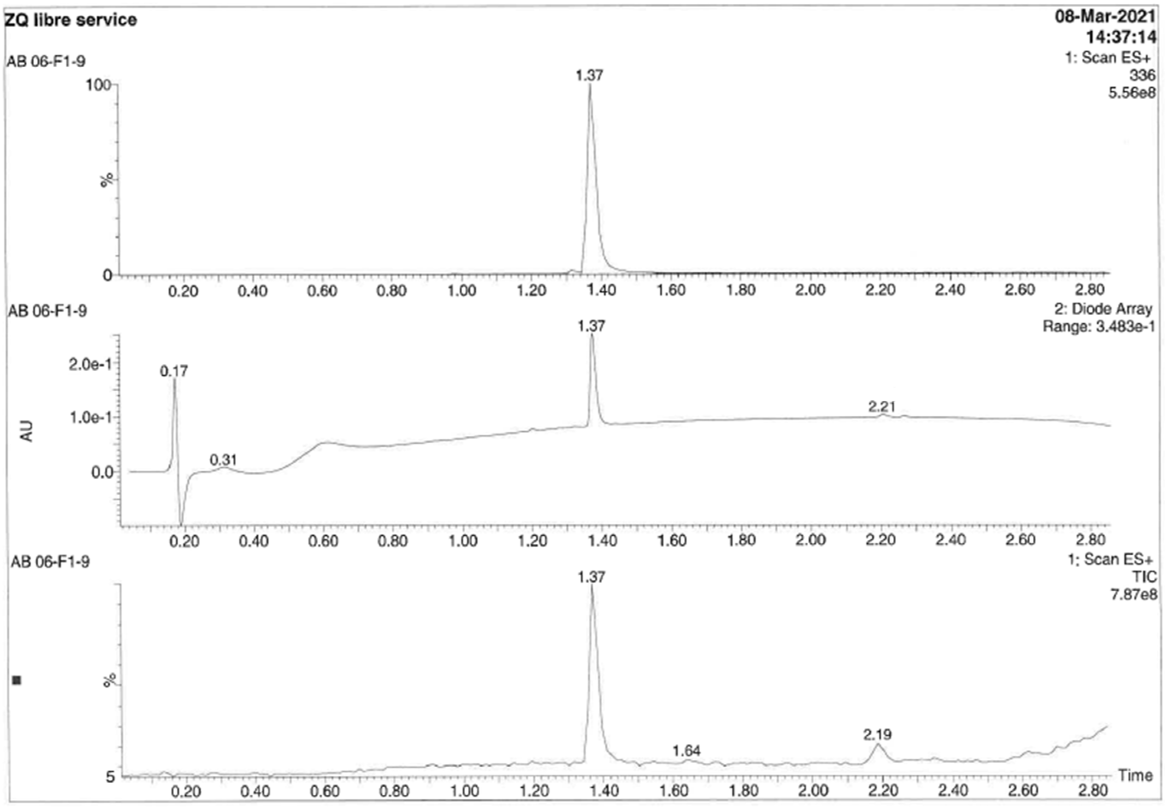

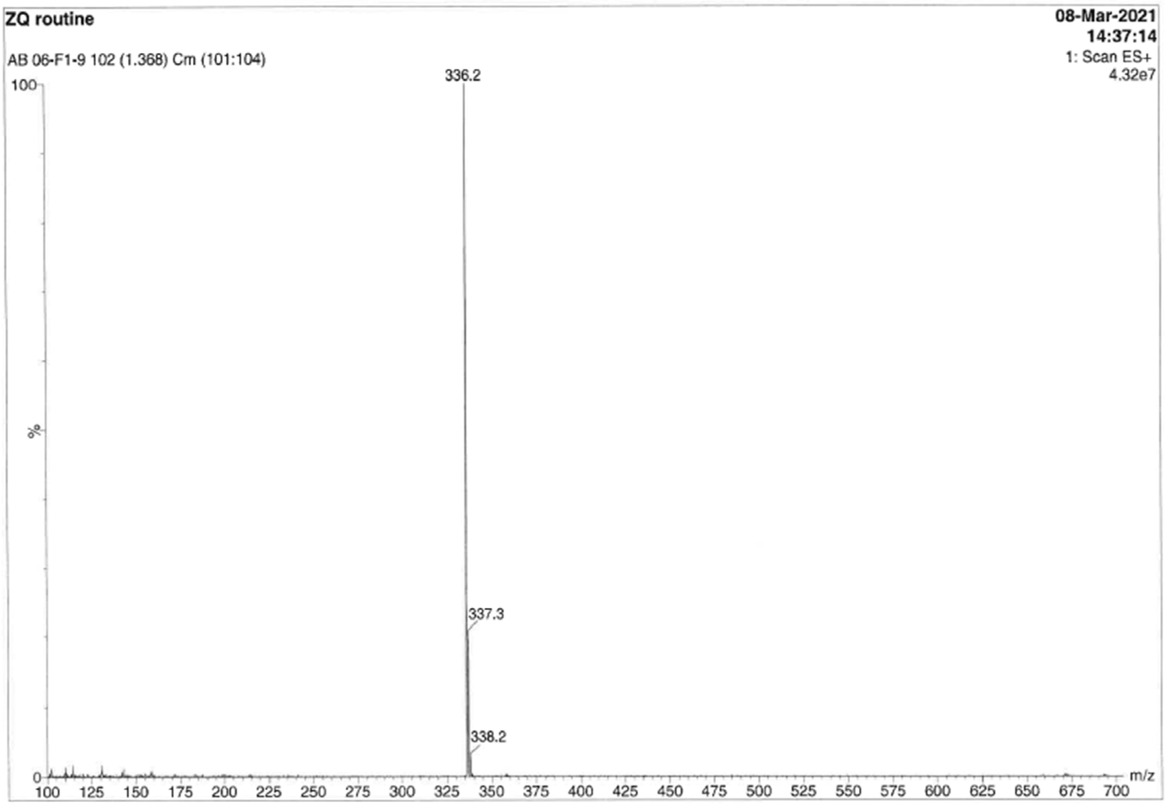

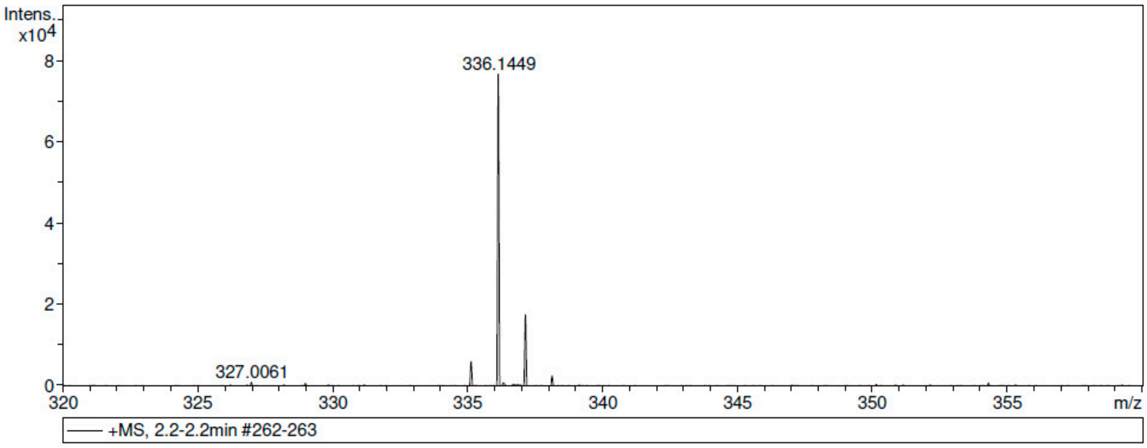

Meas. m/z	#	Ion Formula	m/z	err [ppm]	mSigma	# Sigma	Score	rdb	e⁻ Conf	N-Rule
336.1449	1	C18H18N5O2	336.1455	-1.9	8.2	1	100.00	12.5	even	ok
	2	C17H22NO6	336.1442	-2.1	20.6	2	74.52	7.5	even	ok

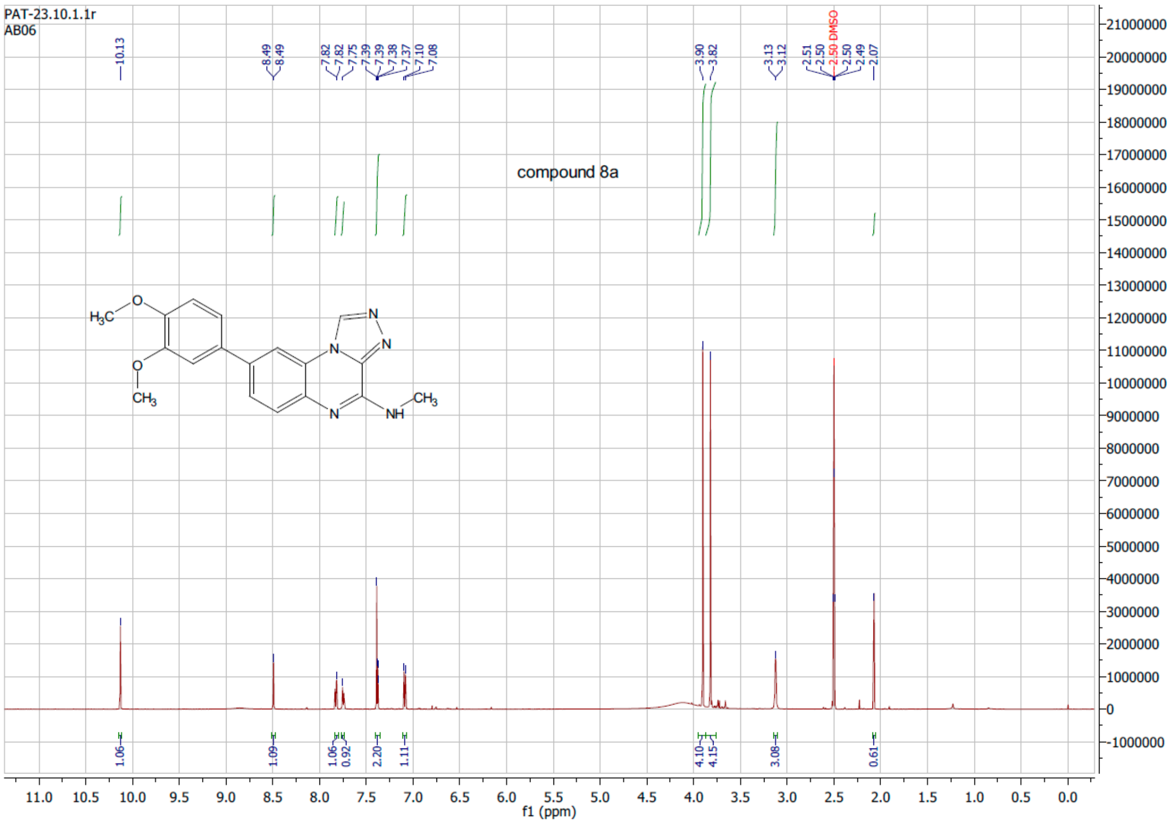

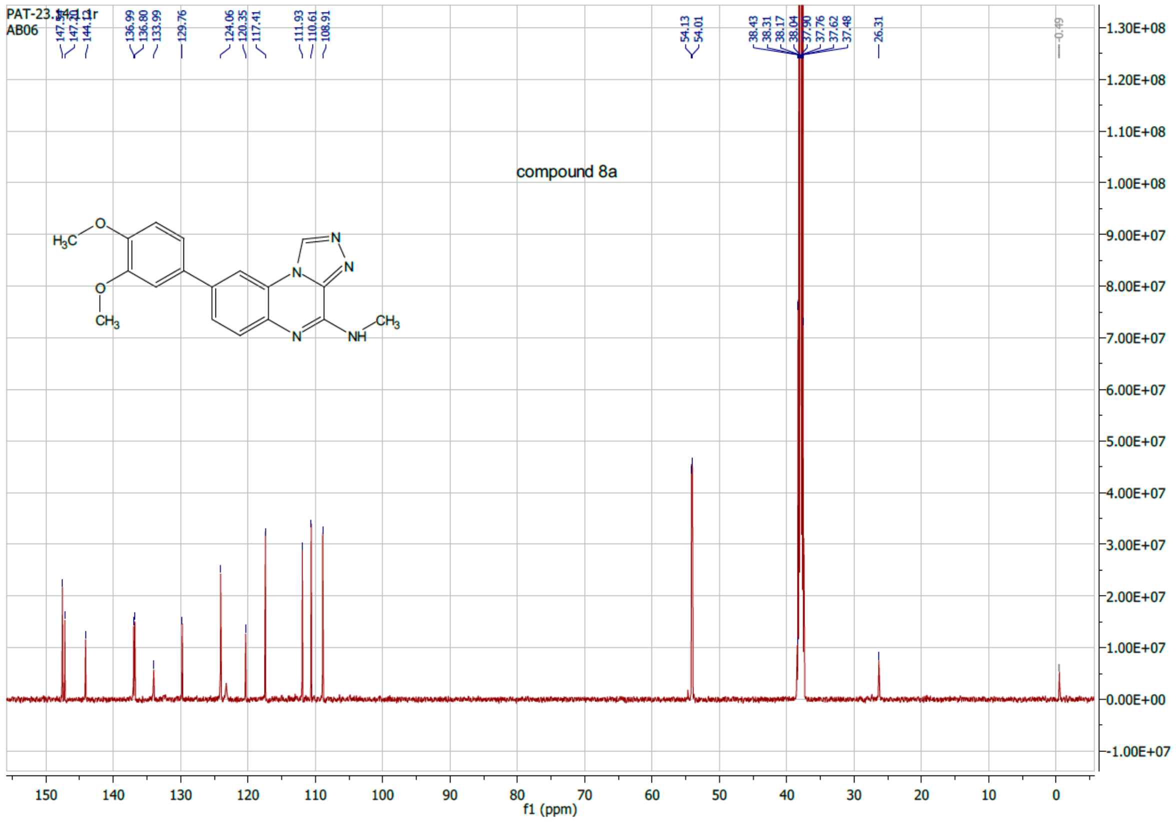

*S4. [8-(3-Methoxy-phenyl)-[1,2,4]triazolo[4,3-a]quinoxalin-4-yl]-methyl-amine (**8b**).*

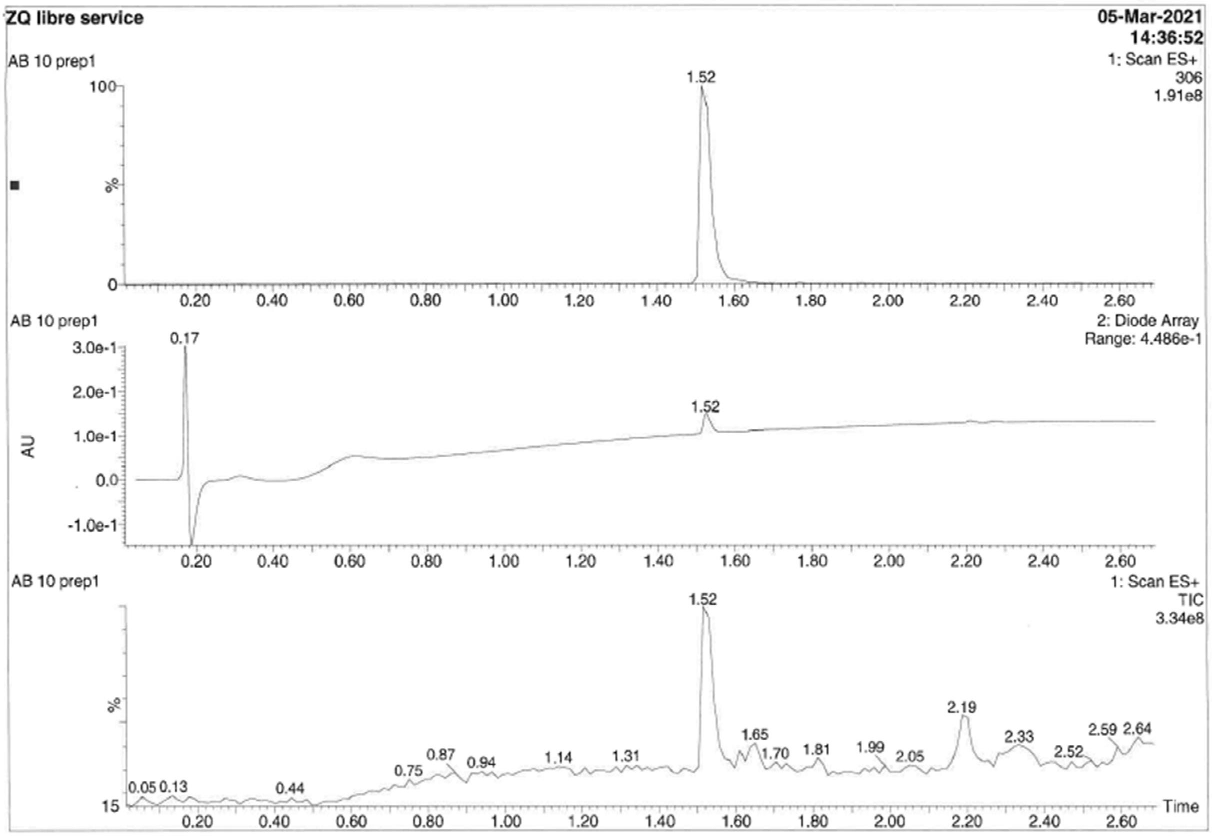

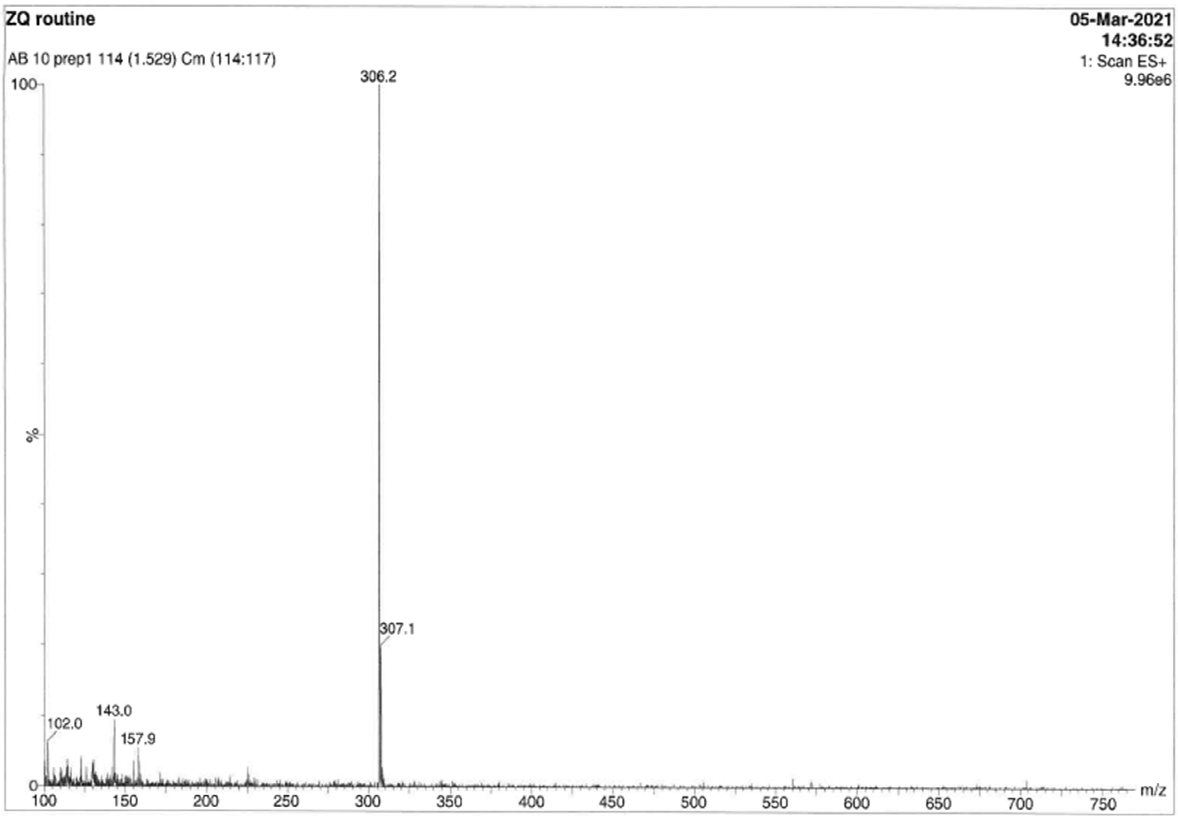

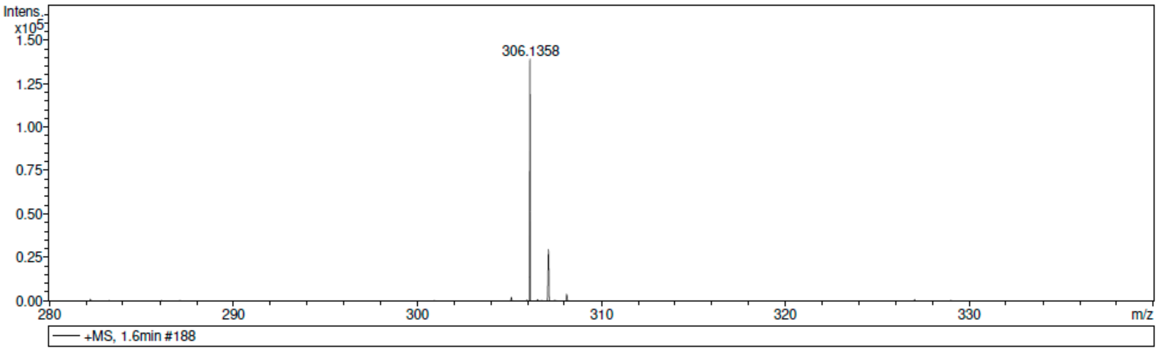

Meas. m/z	#	Ion Formula	m/z	err [ppm]	mSigma	# Sigma	Score	rdb	e⁻ Conf	N-Rule
306.1358	1	C17H16N5O	306.1349	-2.7	7.6	1	100.00	12.5	even	ok

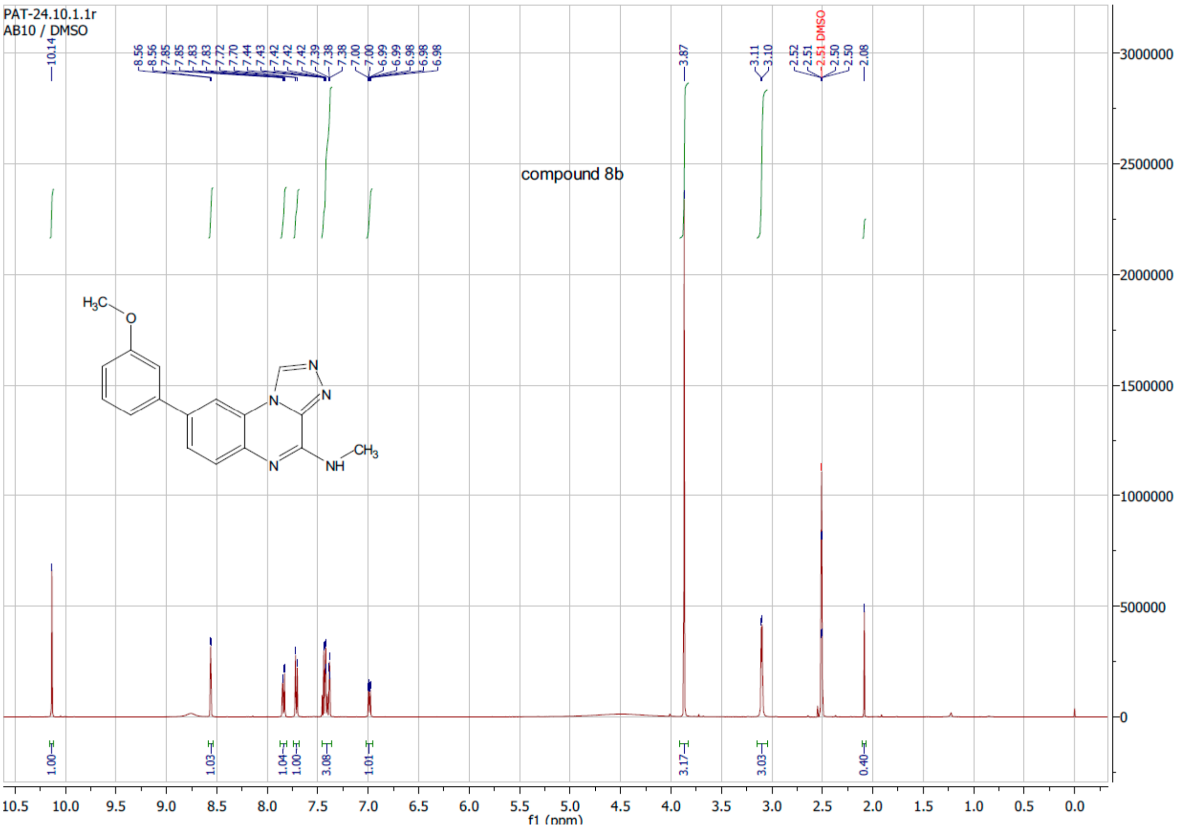
PAT-24.10.1.1r
AB10 / DMSO
10.14
8.56 8.56 7.85 7.85 7.83 7.83 7.72 7.70 7.44 7.43 7.42 7.42 7.42 7.42 7.39 7.38 7.38 7.00 7.00 6.99 6.99 6.98 6.98 6.98
3.87
3.11 3.10
2.52 2.51 2.51 DMSO 2.50 2.50
2.08
compound 8b
1.00 1.03 1.04 1.00 3.08 1.01 3.17 3.03 0.40
f1 (ppm)

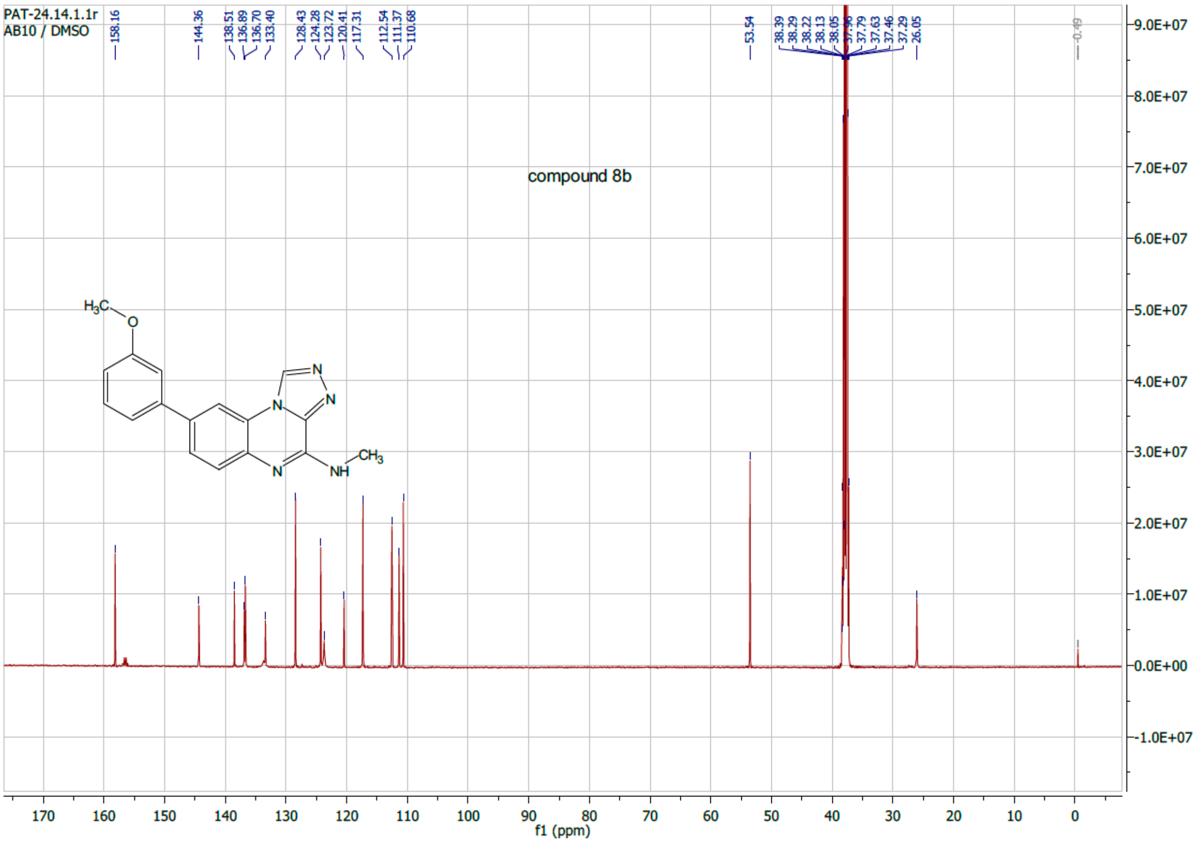
PAT-24.14.1.1r
AB10 / DMSO
158.16
144.36
138.51 136.89 136.70 133.40
128.43 124.28 123.72 120.41 117.31
112.54 111.37 110.68
53.54
38.39 38.29 38.22 38.13 38.05 37.96 37.79 37.63 37.46 37.29
26.05
-0.49
compound 8b
f1 (ppm)

*S5. Methyl-(8-phenyl-[1,2,4]triazolo[4,3-a]quinoxalin-4-yl)-amine (**8c**).*

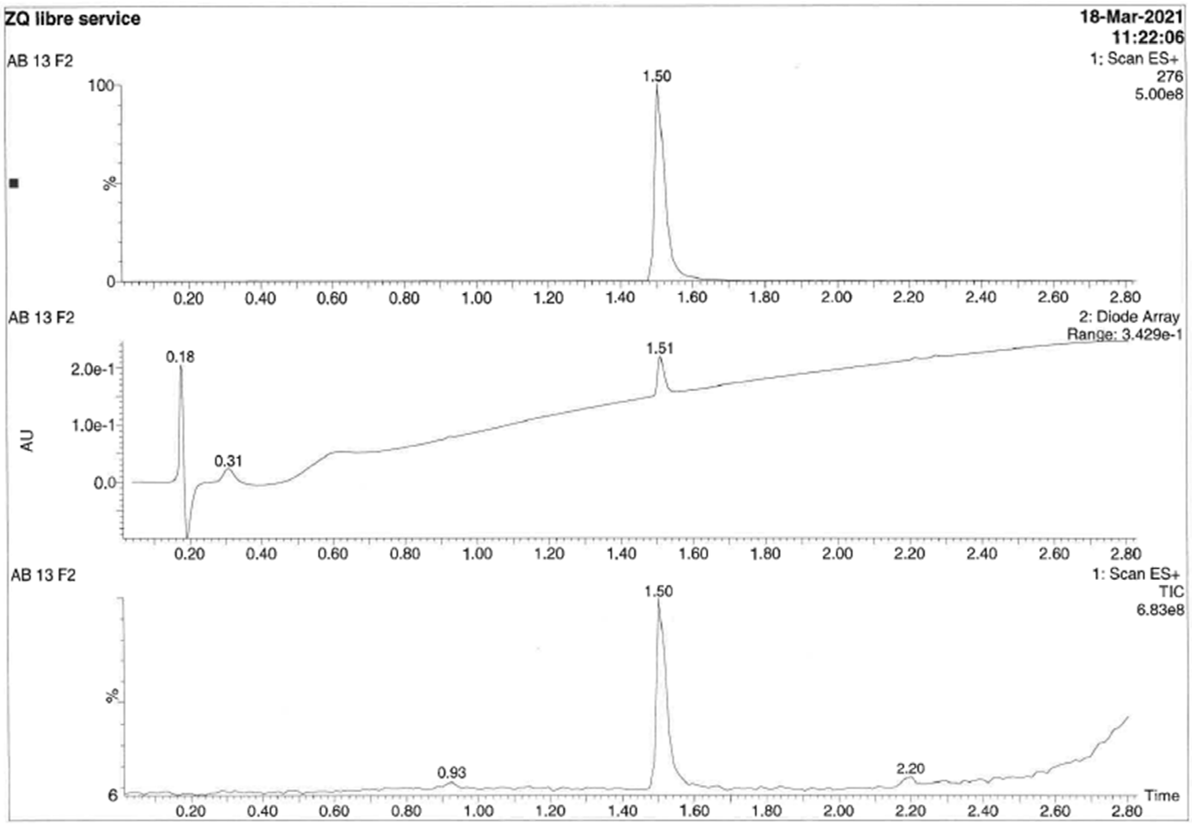

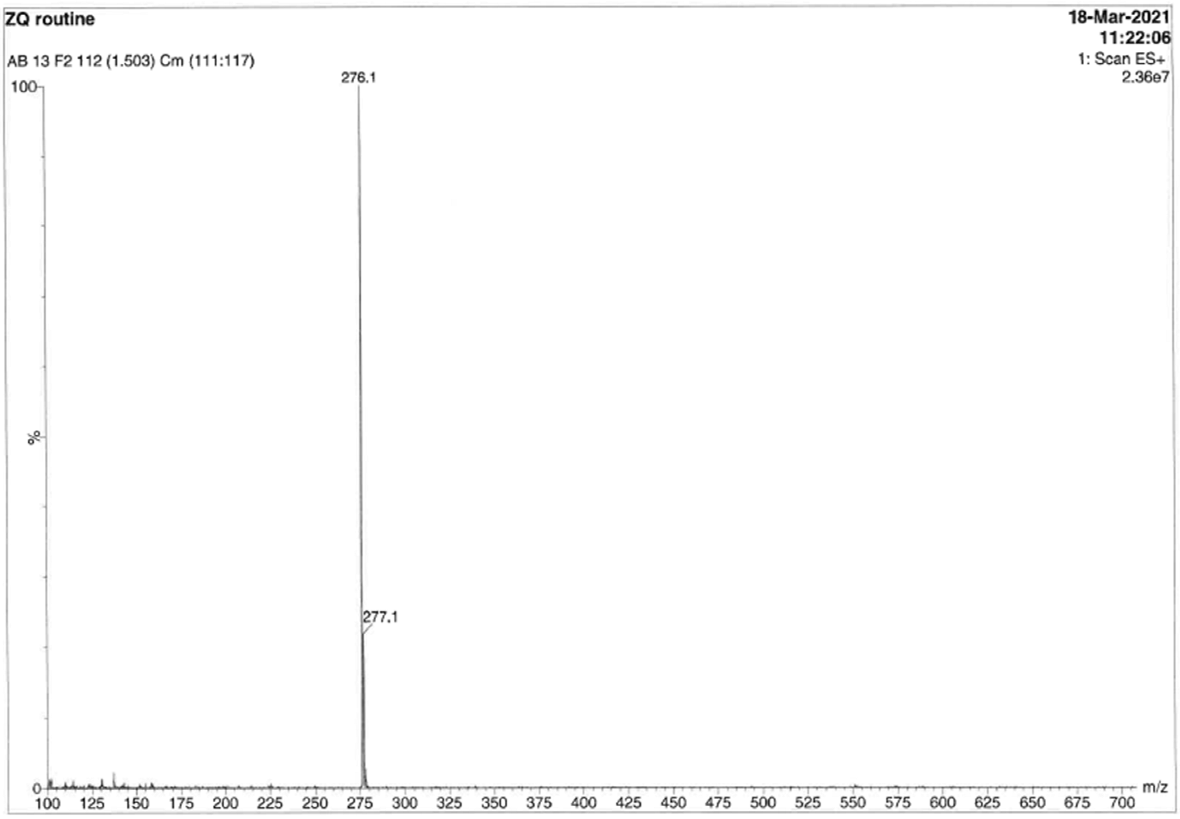

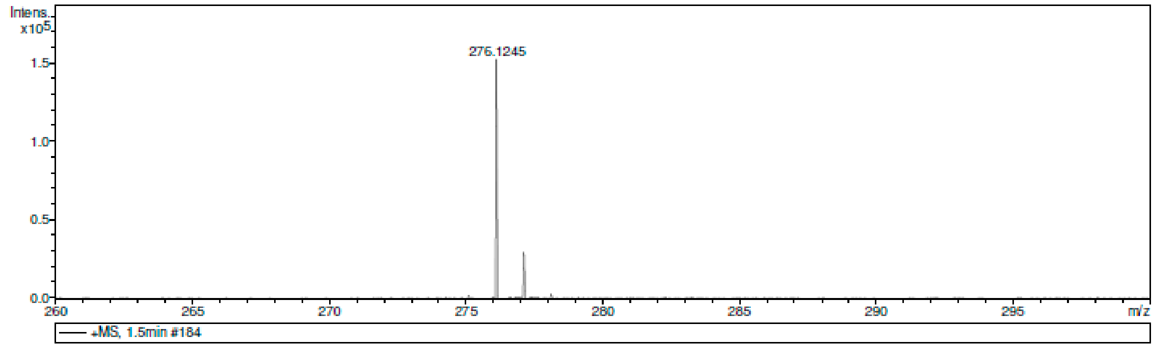

Meas. m/z	#	Ion Formula	m/z	err [ppm]	mSigma	# Sigma	Score	rdb	e⁻ Conf	N-Rule
276.1245	1	C16H14N5	276.1244	-0.3	1.7	1	100.00	12.5	even	ok

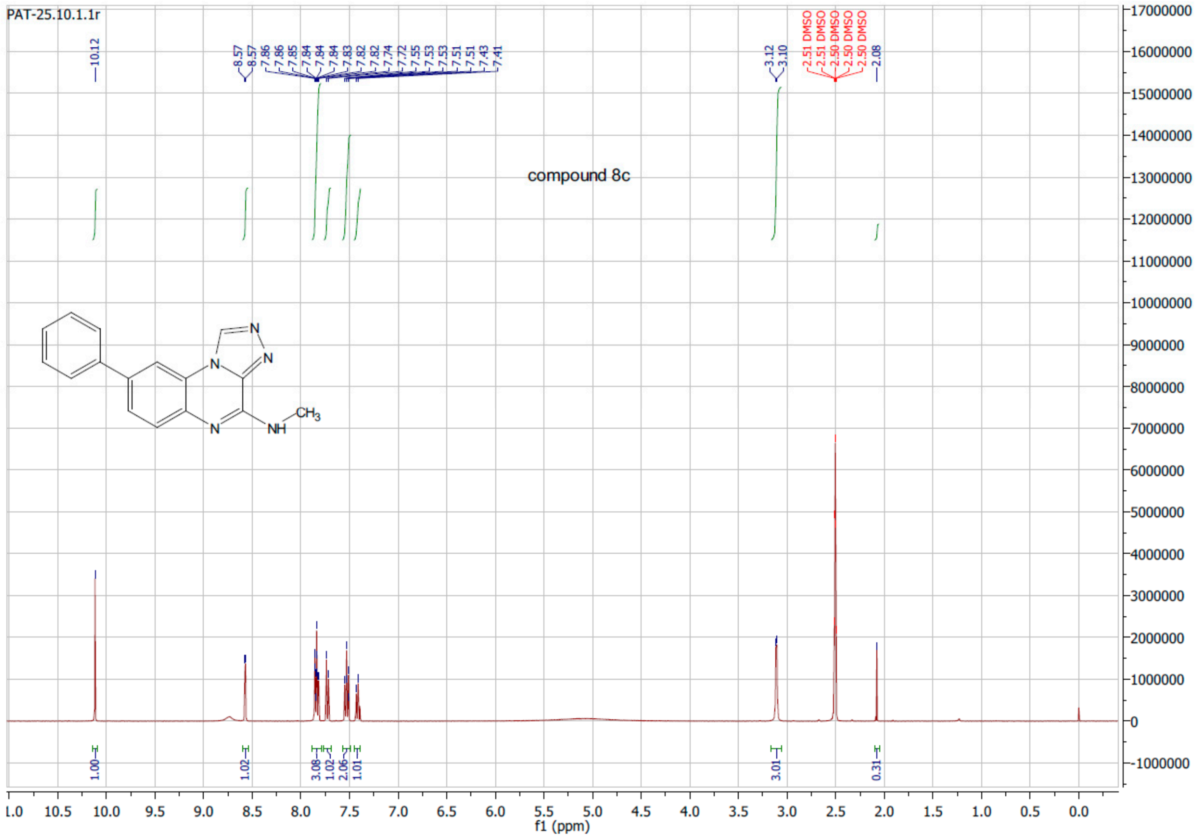

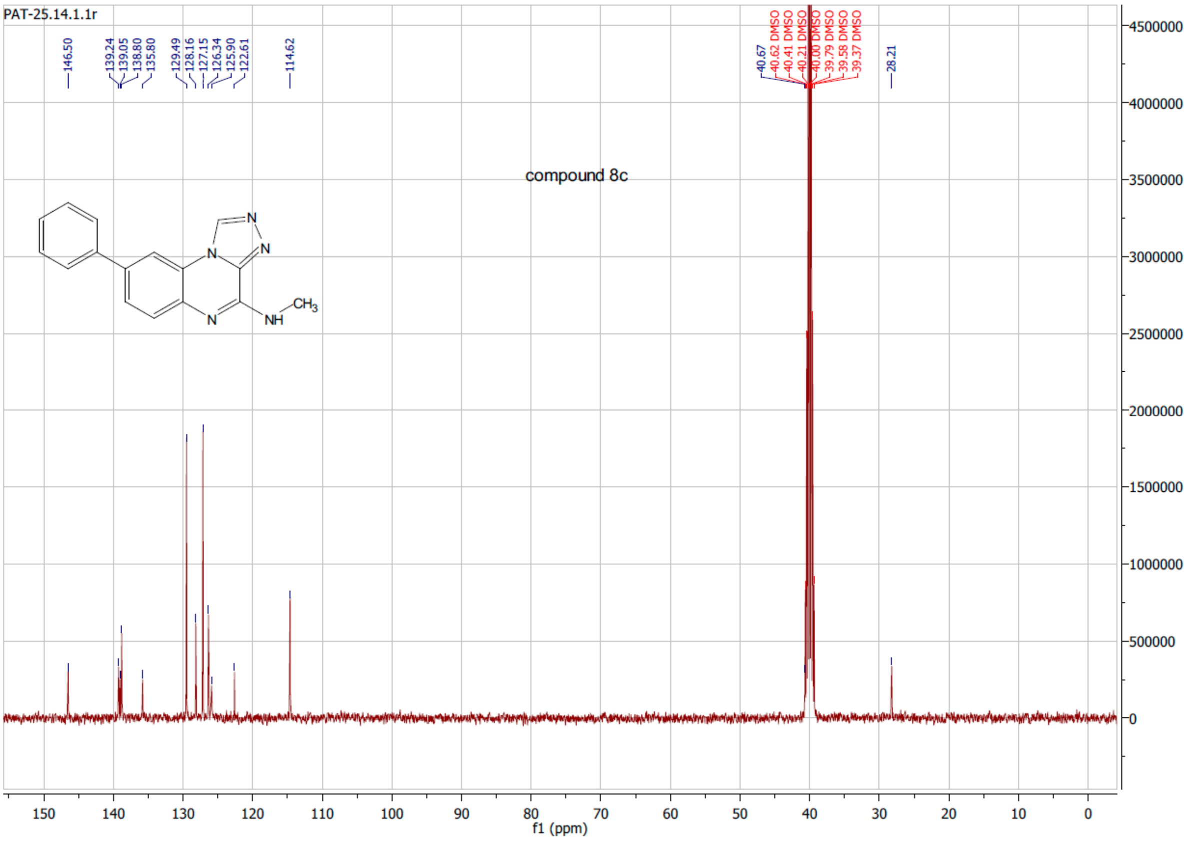

*S6. Methyl-[8-(3,4,5-trimethoxy-phenyl)-[1,2,4]triazolo[4,3-a]quinoxalin-4-yl]-amine (**8d**).*

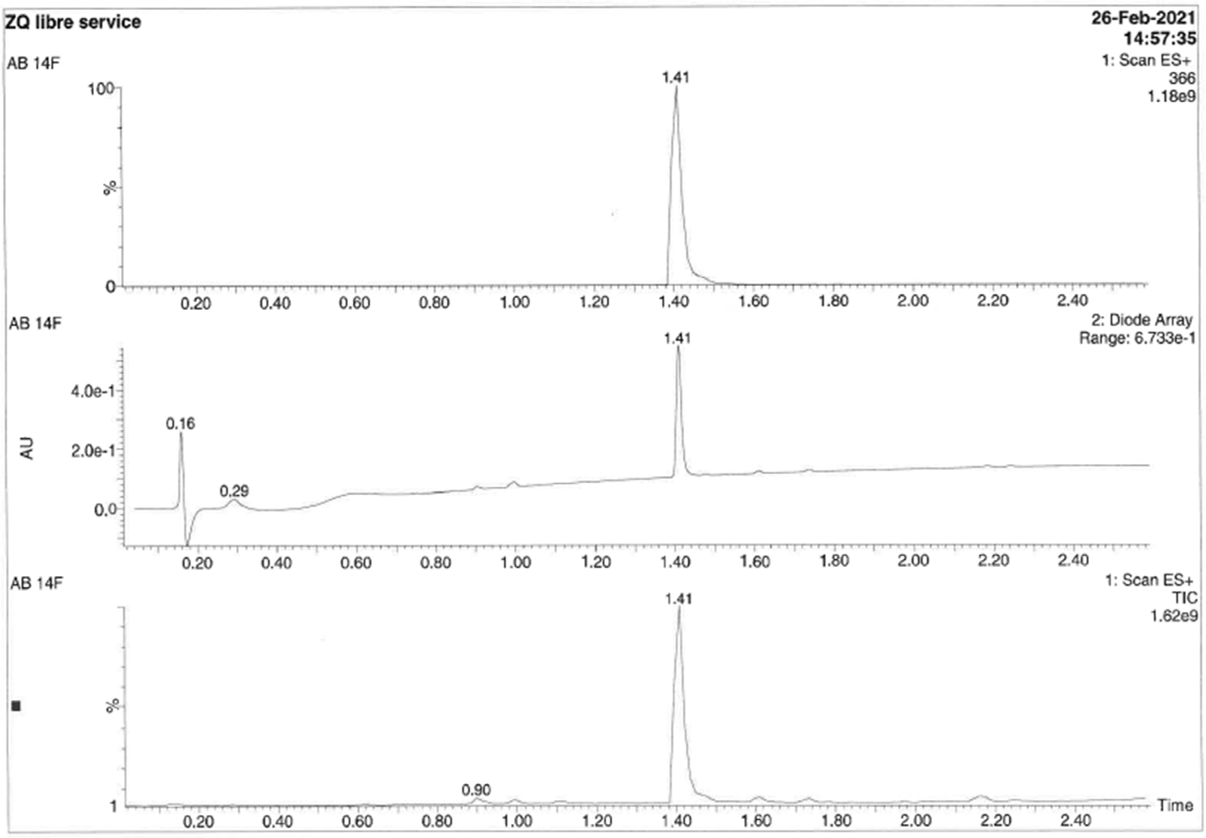

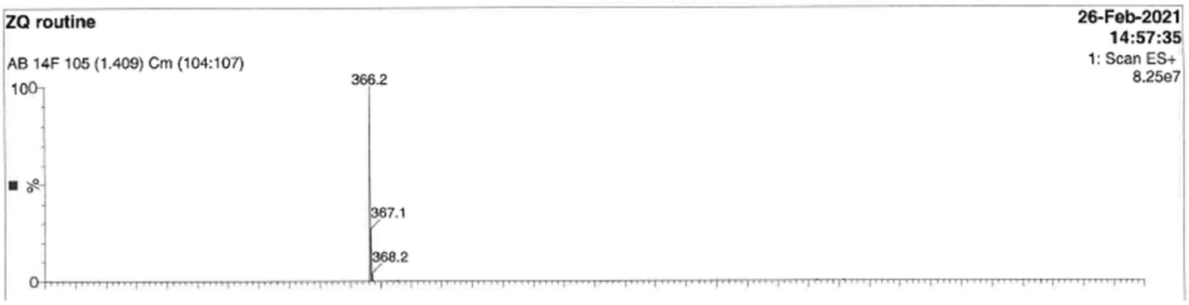

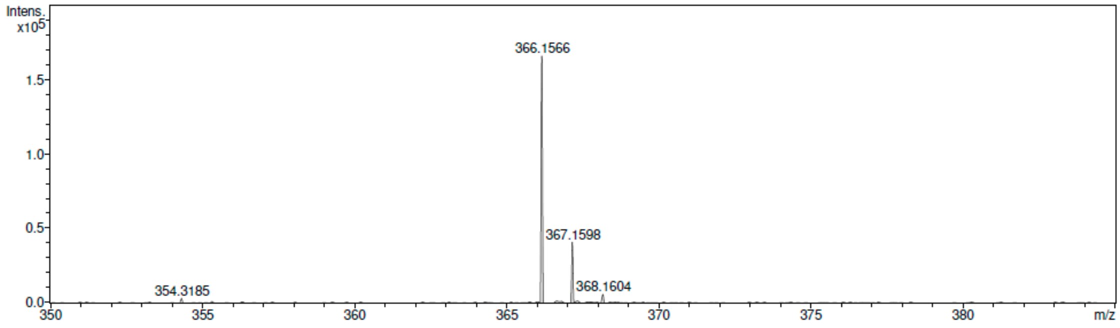

Meas. m/z	#	Ion Formula	m/z	err [ppm]	mSigma	# Sigma	Score	rdb	e⁻ Conf	N-Rule
366.1566	1	C19H20N5O3	366.1561	-1.4	10.4	1	100.00	12.5	even	ok

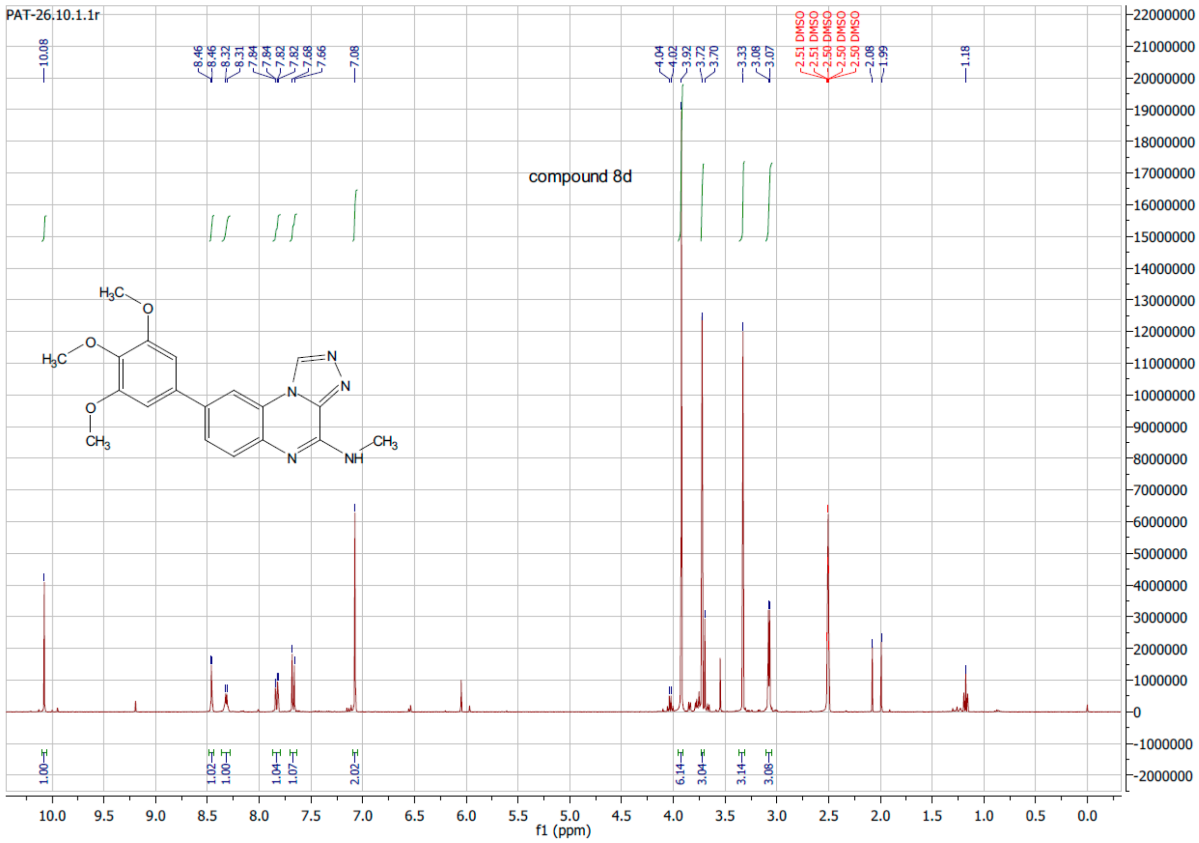

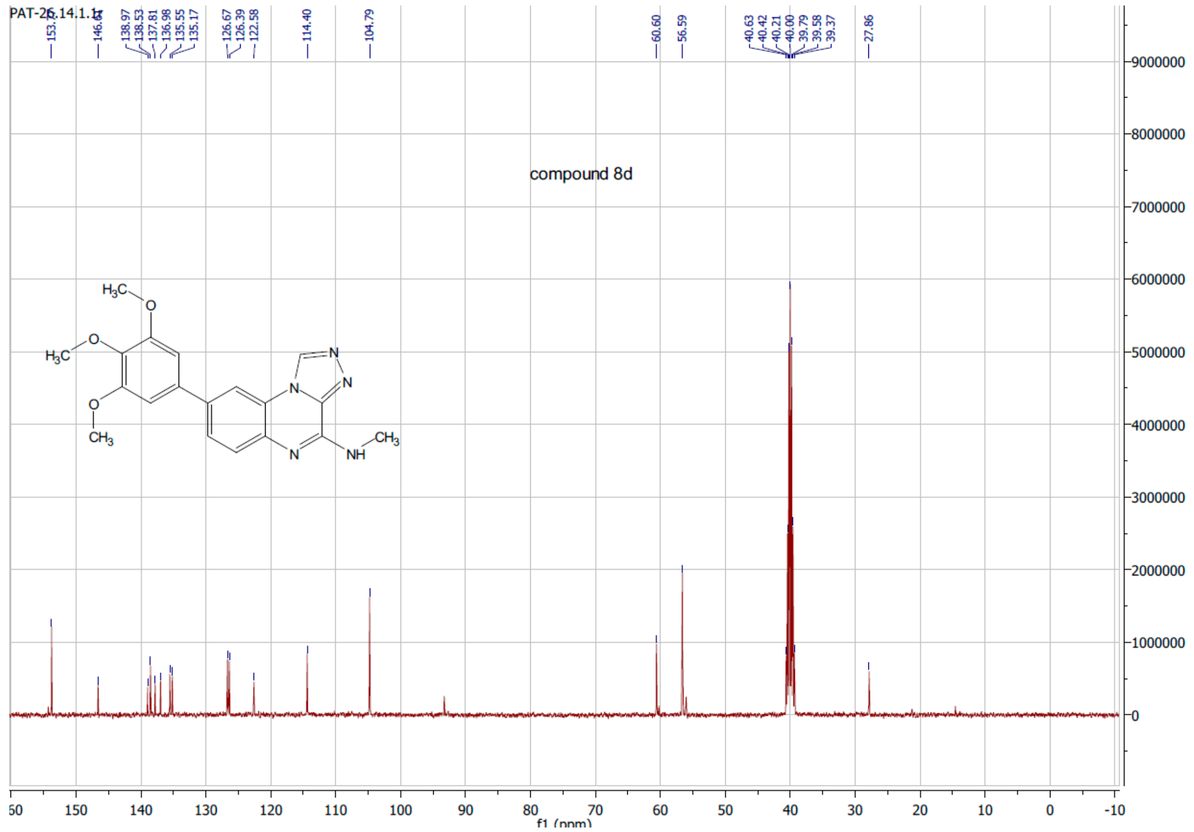

*S7. 2-chloro-3-(2-(3,4-dimethoxybenzylidene)hydrazinyl)quinoxaline (**14a**).*

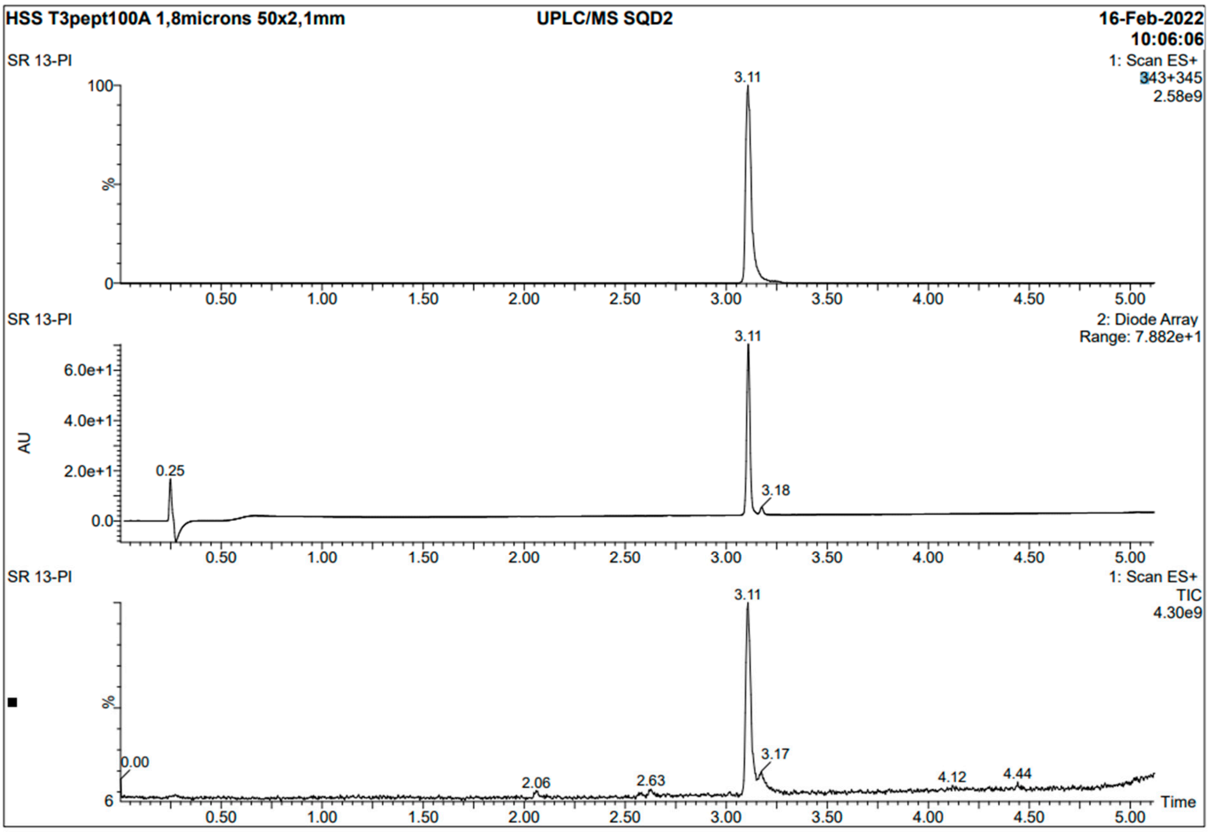

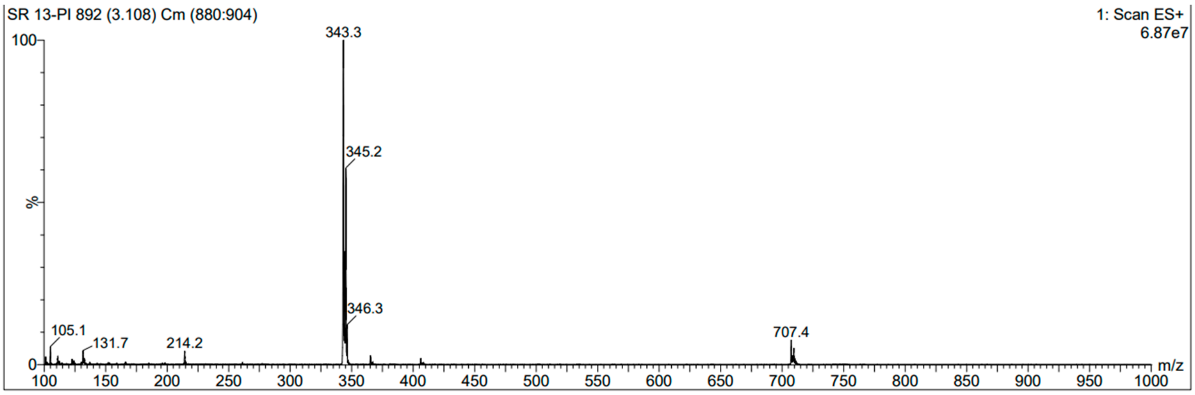
SR 13-PI 892 (3.108) Cm (880:904)
1: Scan ES+
6.87e7
100
%
0
343.3
345.2
346.3
105.1
131.7
214.2
707.4
m/z
100 150 200 250 300 350 400 450 500 550 600 650 700 750 800 850 900 950 1000

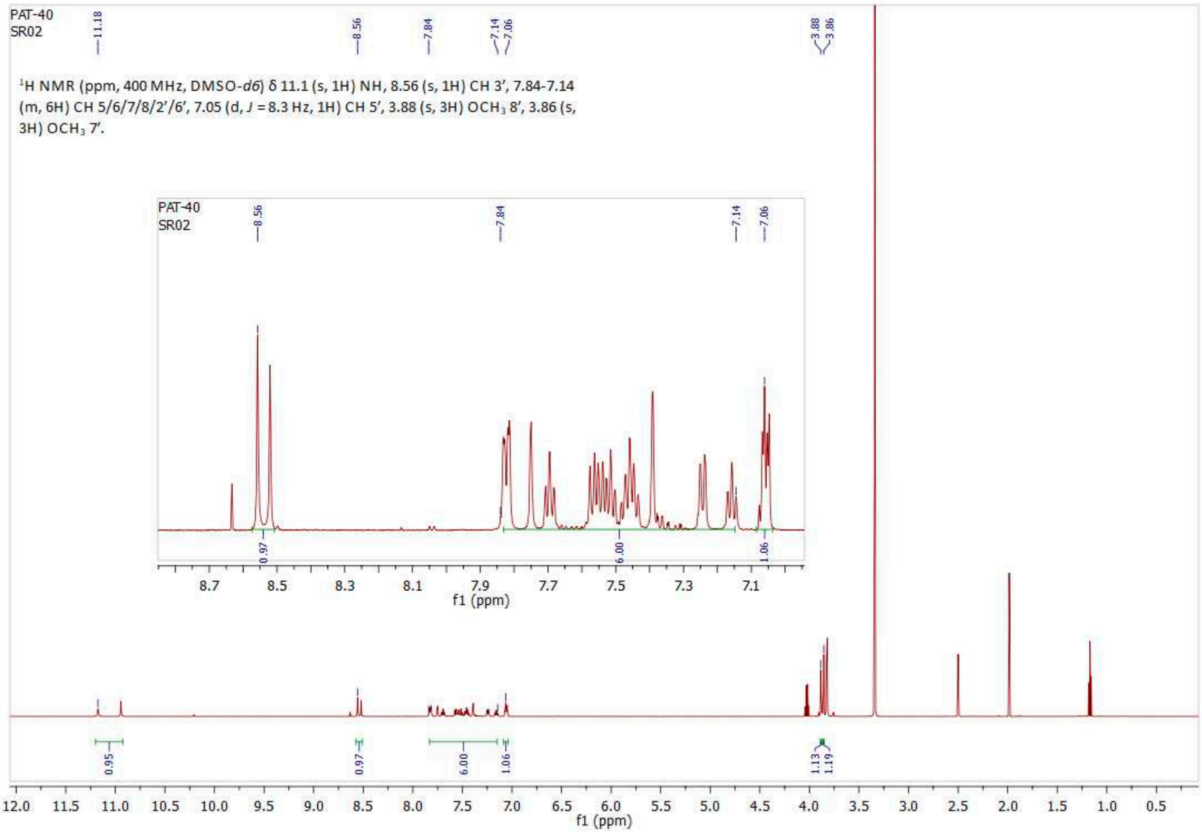
PAT-40
SR02
11.18
8.56
7.84
7.14
7.06
3.88
3.86
^{1}H NMR (ppm, 400 MHz, DMSO-*d6*) δ 11.1 (s, 1H) NH, 8.56 (s, 1H) CH 3′, 7.84-7.14 (m, 6H) CH 5/6/7/8/2′/6′, 7.05 (d, *J* = 8.3 Hz, 1H) CH 5′, 3.88 (s, 3H) OCH_3 8′, 3.86 (s, 3H) OCH_3 7′.
PAT-40
SR02
8.56
7.84
7.14
7.06
0.97
6.00
1.06
8.7 8.5 8.3 8.1 7.9 7.7 7.5 7.3 7.1
f1 (ppm)
0.95
0.97
6.00
1.06
1.13
1.19
12.0 11.5 11.0 10.5 10.0 9.5 9.0 8.5 8.0 7.5 7.0 6.5 6.0 5.5 5.0 4.5 4.0 3.5 3.0 2.5 2.0 1.5 1.0 0.5
f1 (ppm)

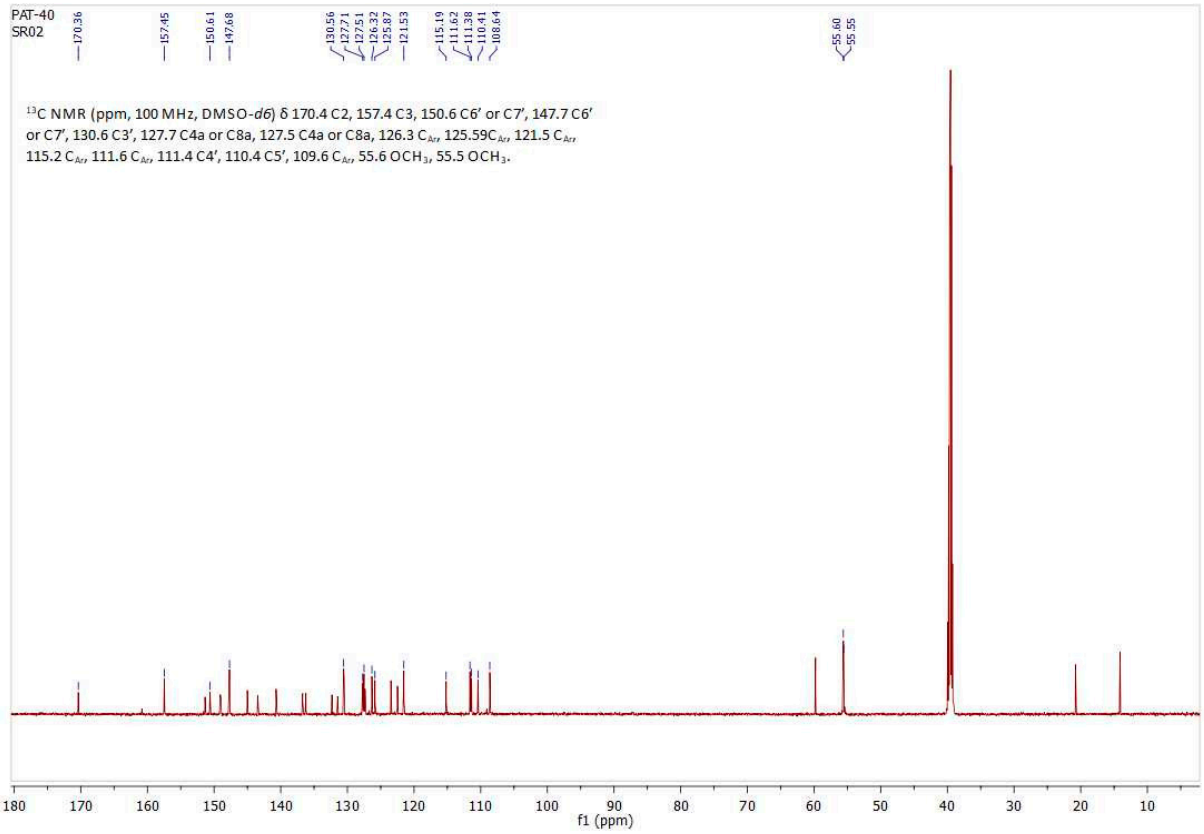

*S8. 2-chloro-3-(2-(3-methoxybenzylidene)hydrazinyl)quinoxaline (**14b**).*

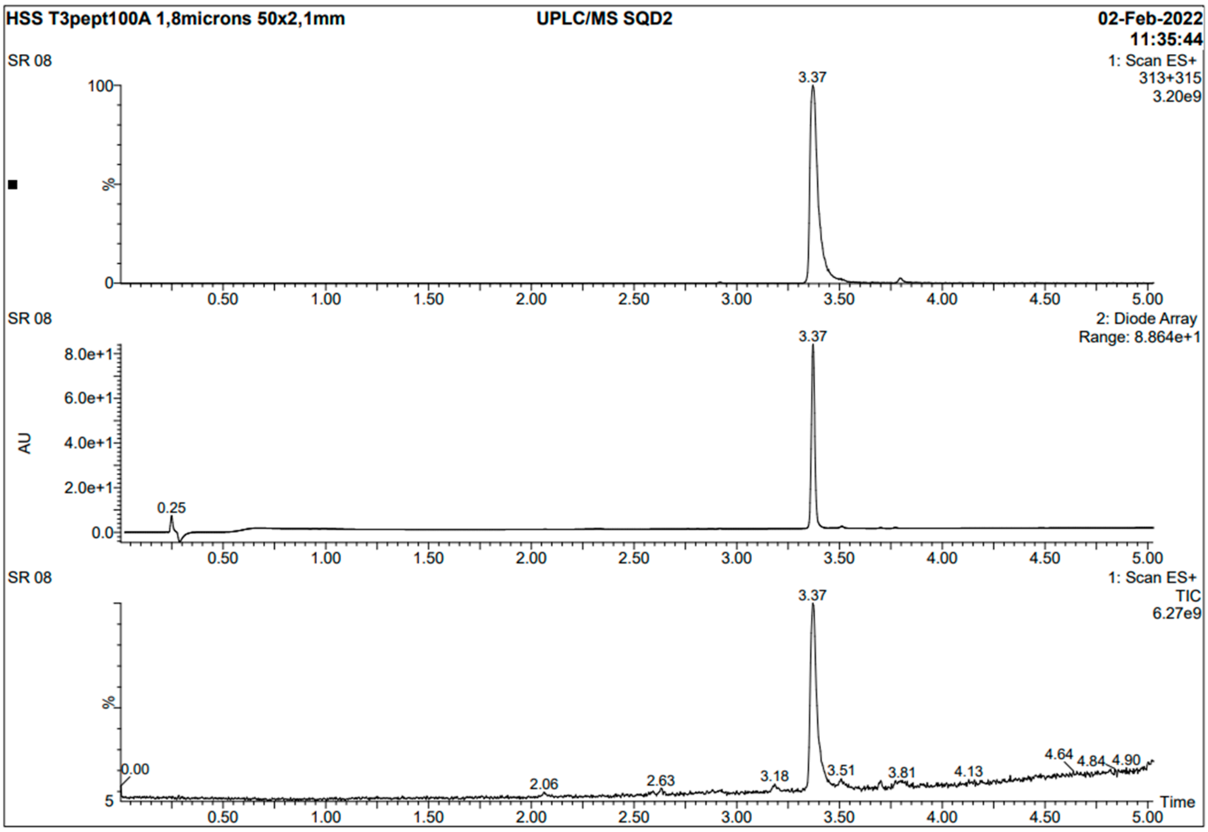

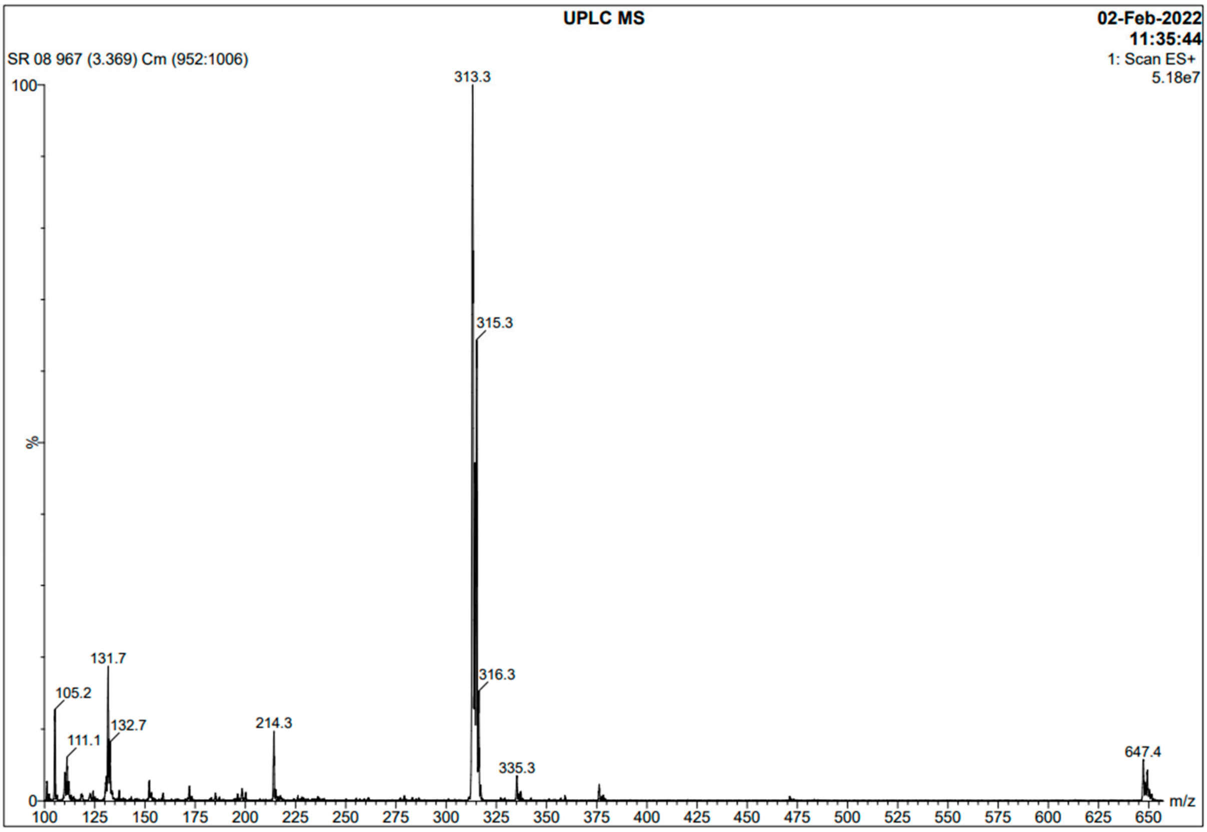

UPLC MS
02-Feb-2022
11:35:44
SR 08 967 (3.369) Cm (952:1006)
1: Scan ES+
5.18e7
313.3
315.3
316.3
335.3
131.7
105.2
111.1
132.7
214.3
647.4
%
m/z

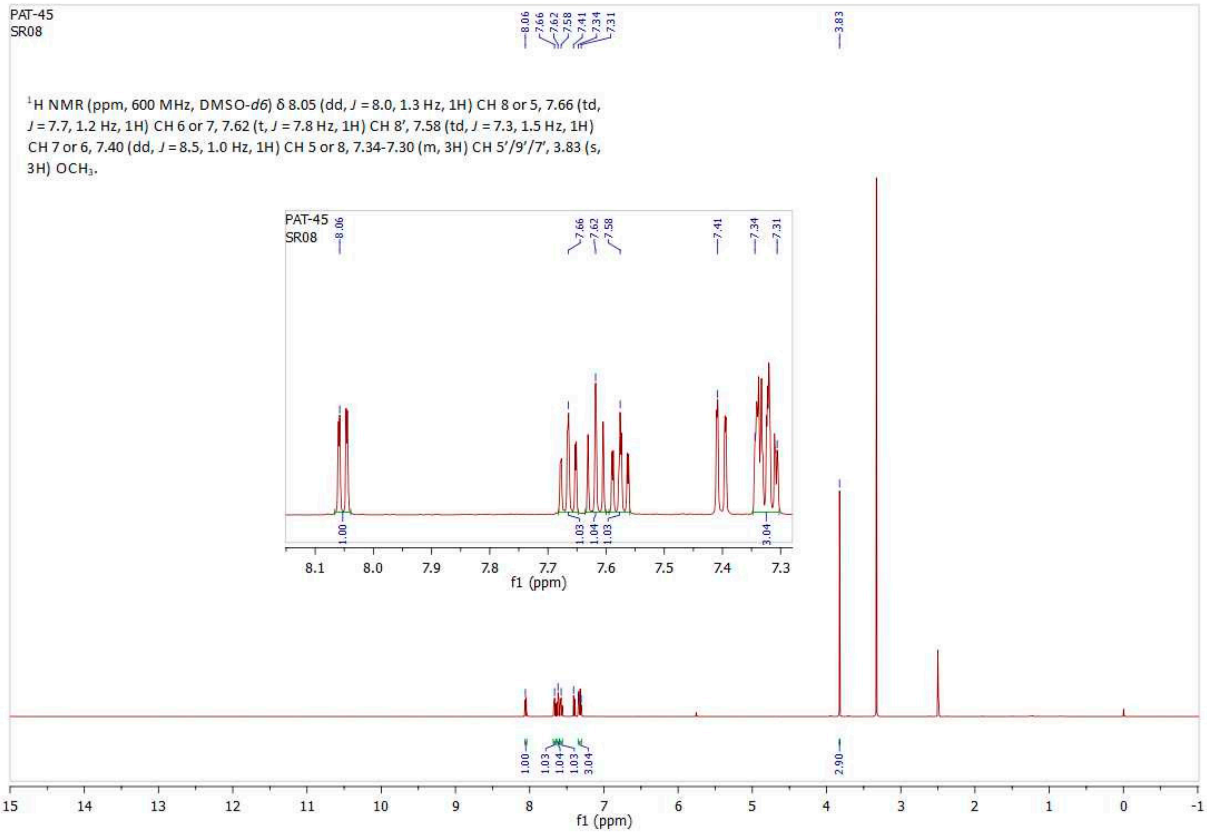

PAT-45
SR08
^{1}H NMR (ppm, 600 MHz, DMSO-d6) δ 8.05 (dd, J = 8.0, 1.3 Hz, 1H) CH 8 or 5, 7.66 (td, J = 7.7, 1.2 Hz, 1H) CH 6 or 7, 7.62 (t, J = 7.8 Hz, 1H) CH 8', 7.58 (td, J = 7.3, 1.5 Hz, 1H) CH 7 or 6, 7.40 (dd, J = 8.5, 1.0 Hz, 1H) CH 5 or 8, 7.34-7.30 (m, 3H) CH 5'/9'/7', 3.83 (s, 3H) OCH3.
f1 (ppm)

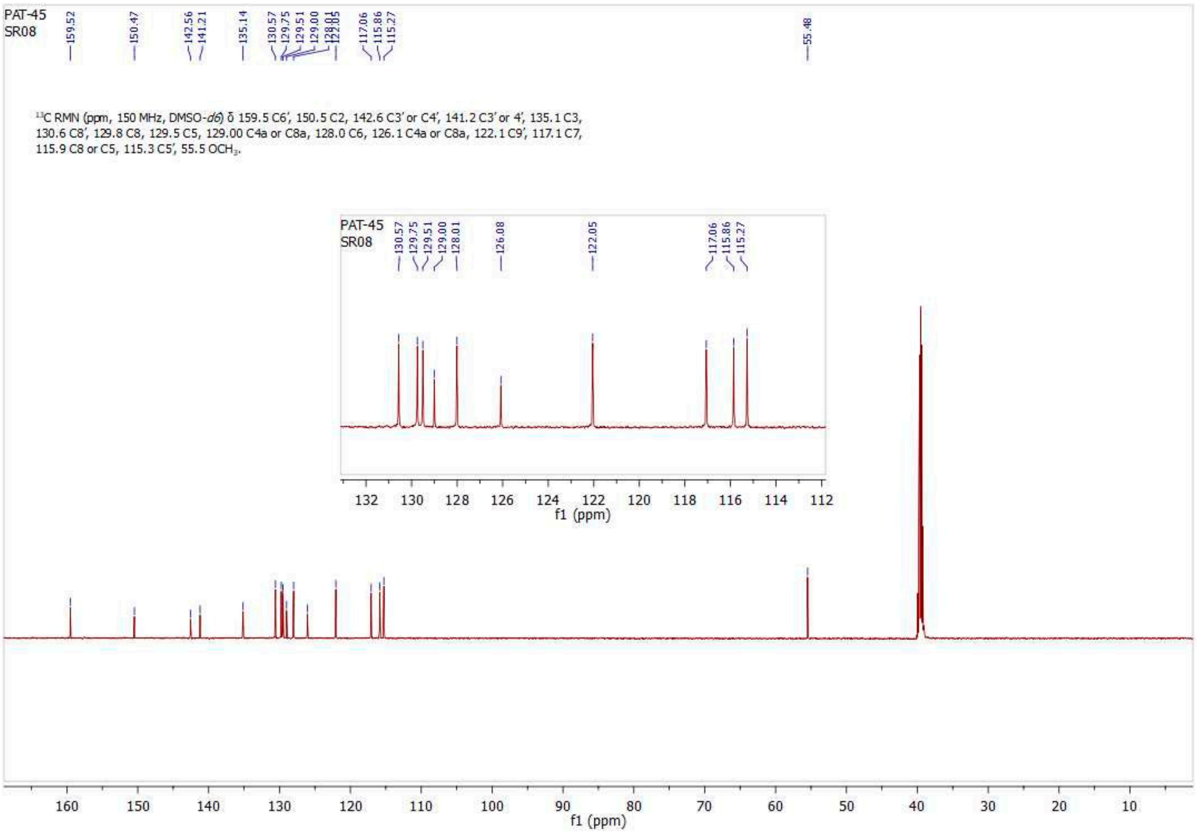

*S9. 2-(2-benzylidenehydrazinyl)-3-chloroquinoxaline (**14c**).*

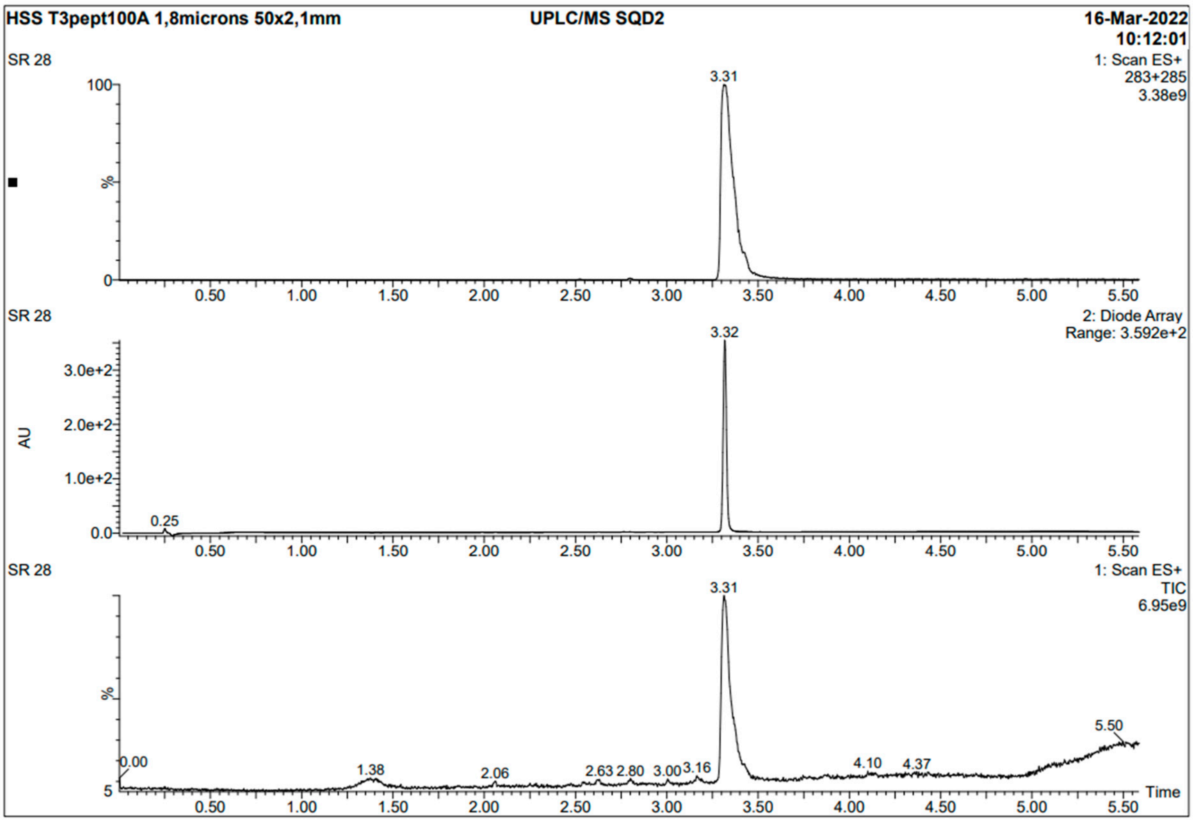

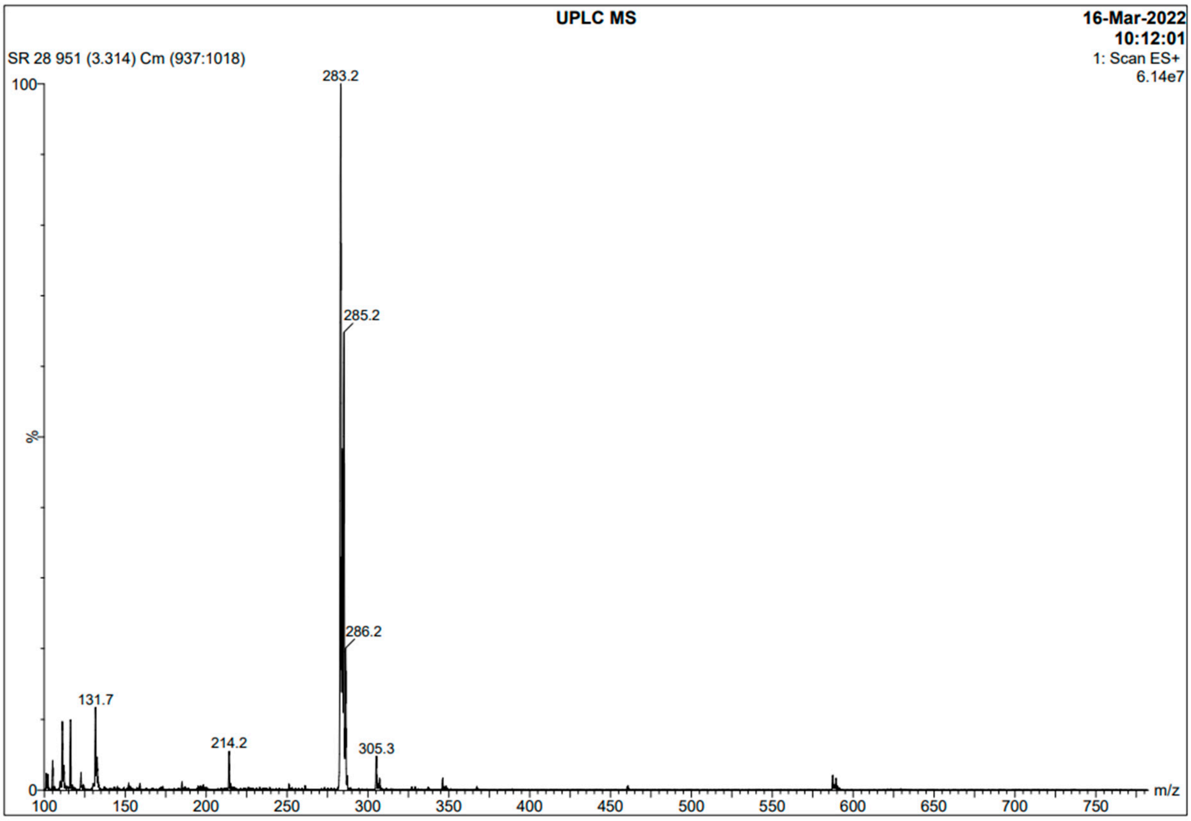
UPLC MS
16-Mar-2022
10:12:01
SR 28 951 (3.314) Cm (937:1018)
1: Scan ES+
6.14e7
283.2
285.2
286.2
131.7
214.2
305.3
%
m/z

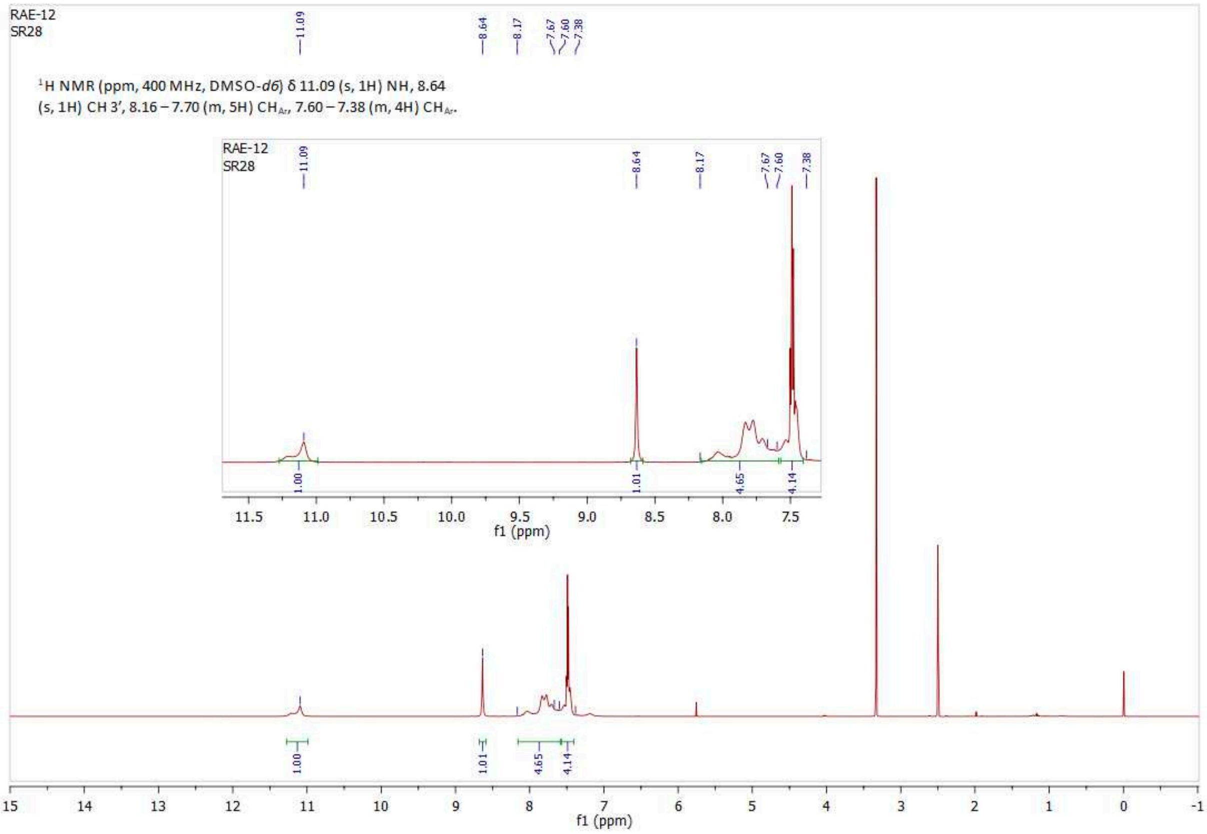
RAE-12
SR28
^{1}H NMR (ppm, 400 MHz, DMSO-d6) δ 11.09 (s, 1H) NH, 8.64 (s, 1H) CH 3′, 8.16 – 7.70 (m, 5H) CH$_{Ar}$, 7.60 – 7.38 (m, 4H) CH$_{Ar}$.
11.09
8.64
8.17
7.67
7.60
7.38
1.00
1.01
4.65
4.14
f1 (ppm)

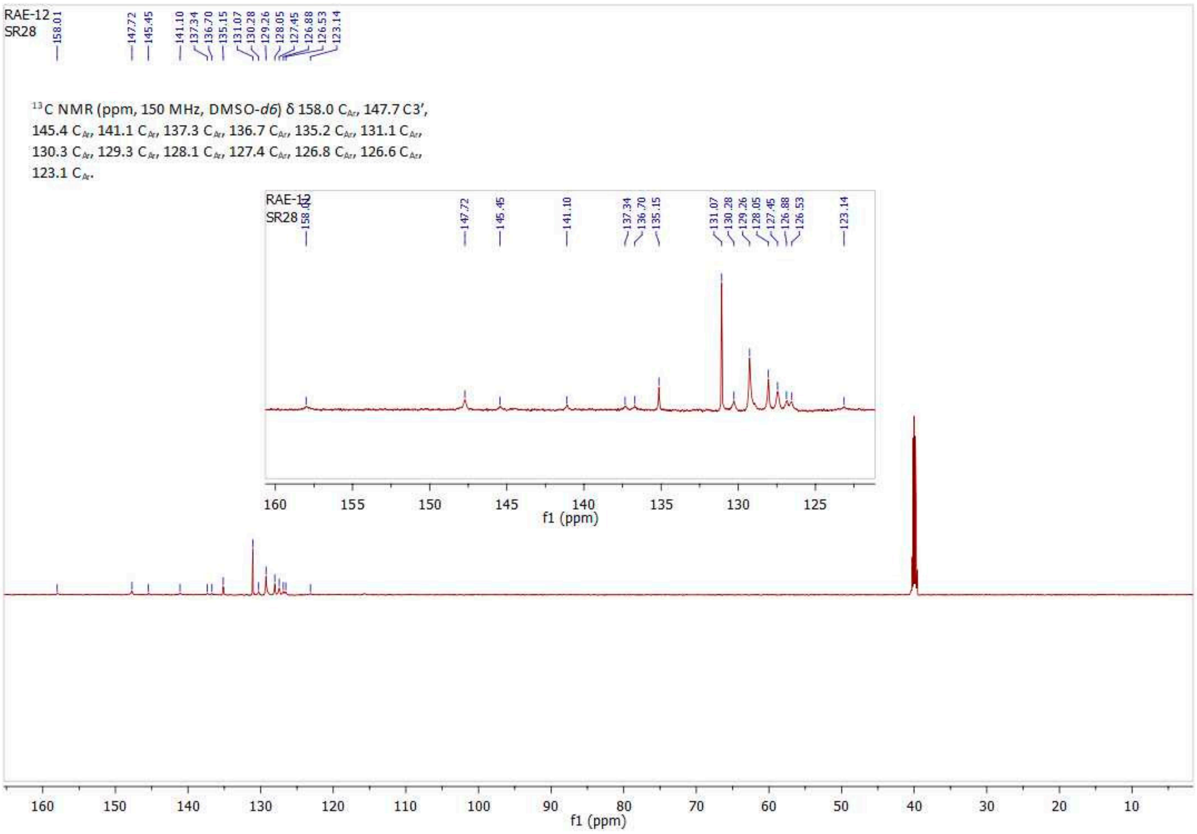

*S10. 2-chloro-3-(2-(3,4,5-trimethoxybenzylidene)hydrazinyl)quinoxaline (**14d**).*

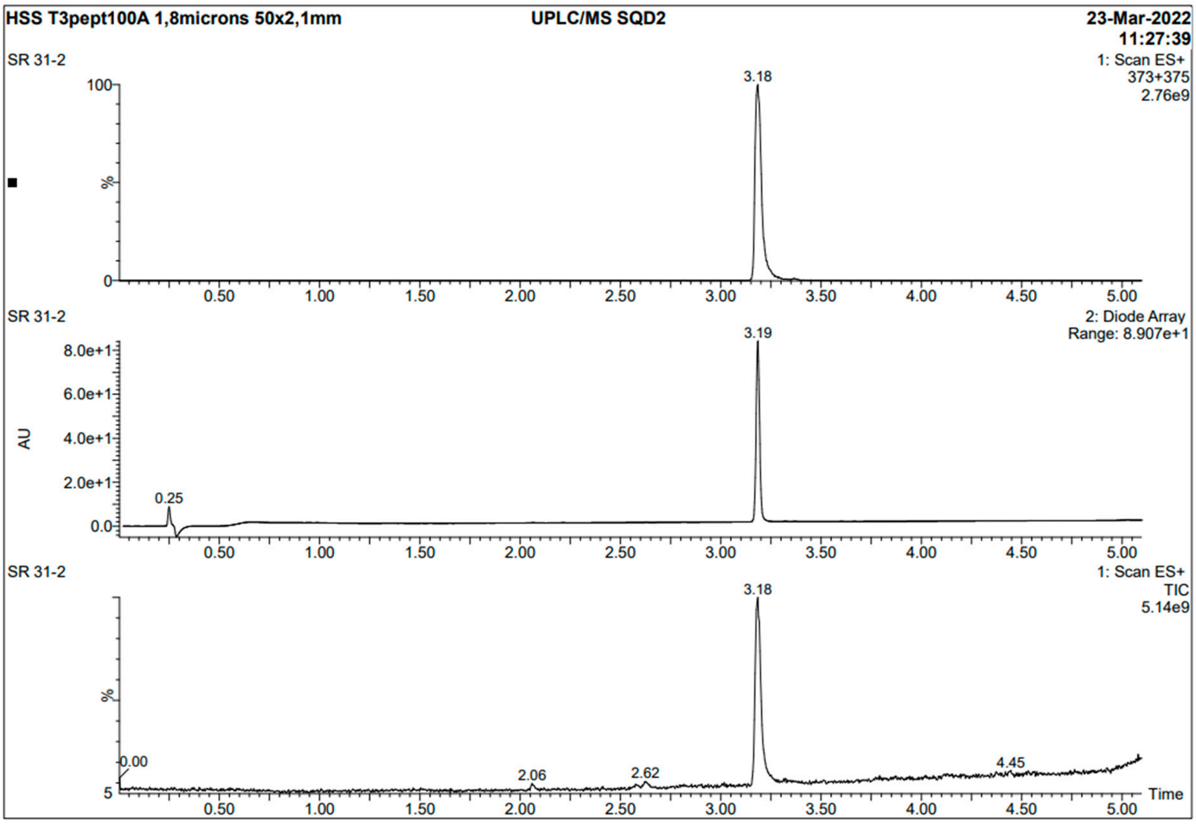

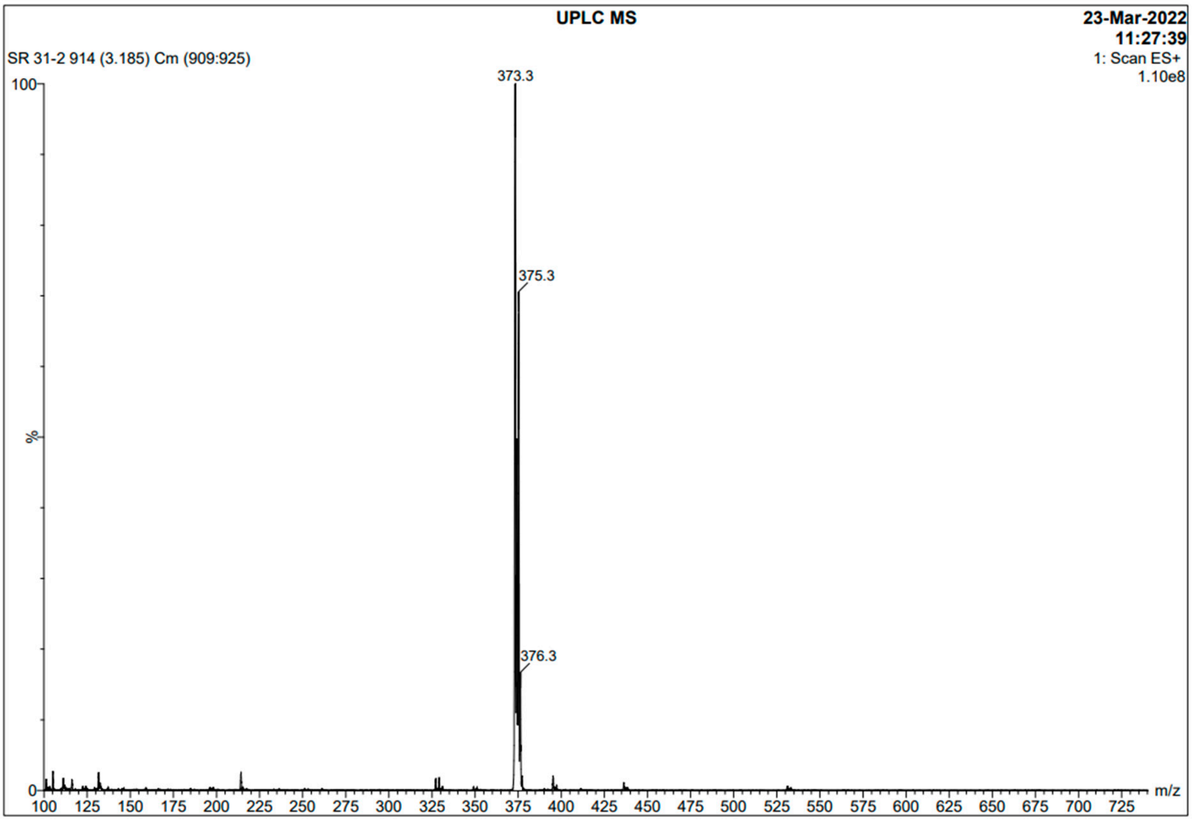
UPLC MS
23-Mar-2022
11:27:39
SR 31-2 914 (3.185) Cm (909:925)
1: Scan ES+
1.10e8
373.3
375.3
376.3
%
m/z

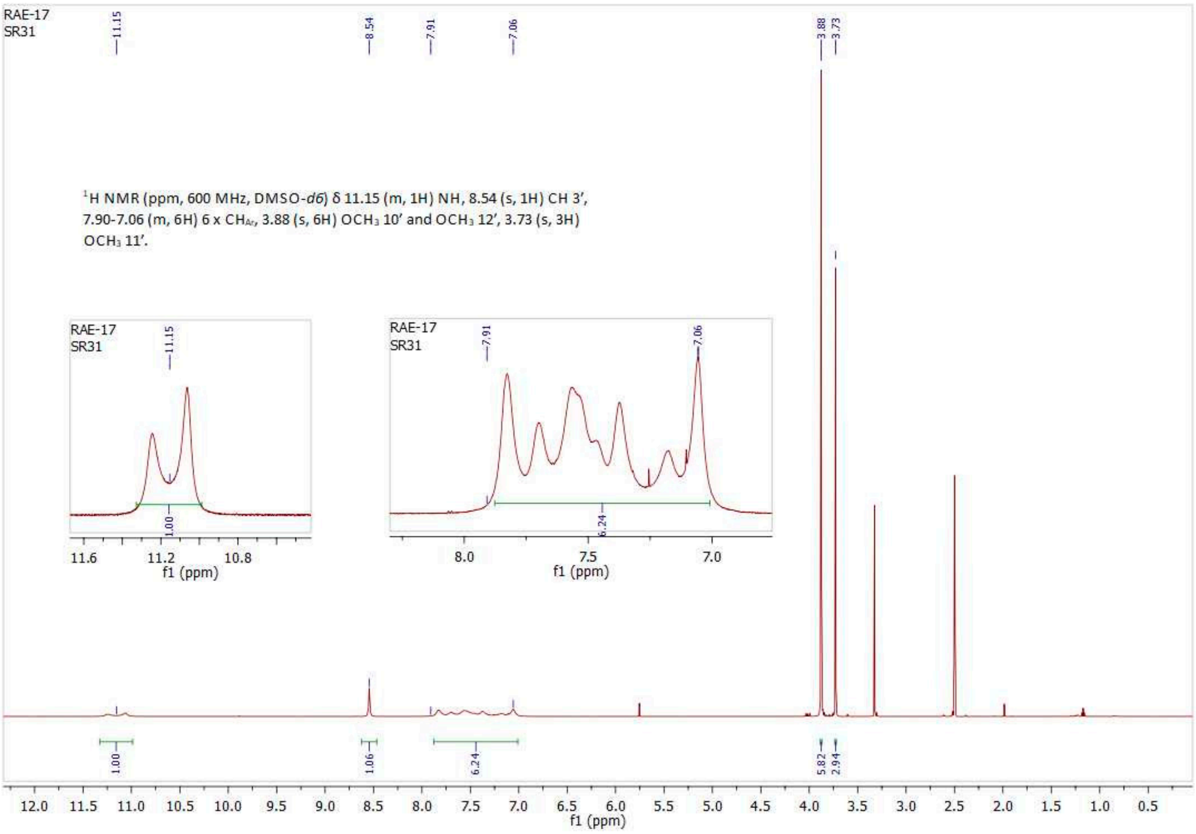
RAE-17
SR31
1H NMR (ppm, 600 MHz, DMSO-d6) δ 11.15 (m, 1H) NH, 8.54 (s, 1H) CH 3′, 7.90-7.06 (m, 6H) 6 x CHAr, 3.88 (s, 6H) OCH3 10′ and OCH3 12′, 3.73 (s, 3H) OCH3 11′.
f1 (ppm)

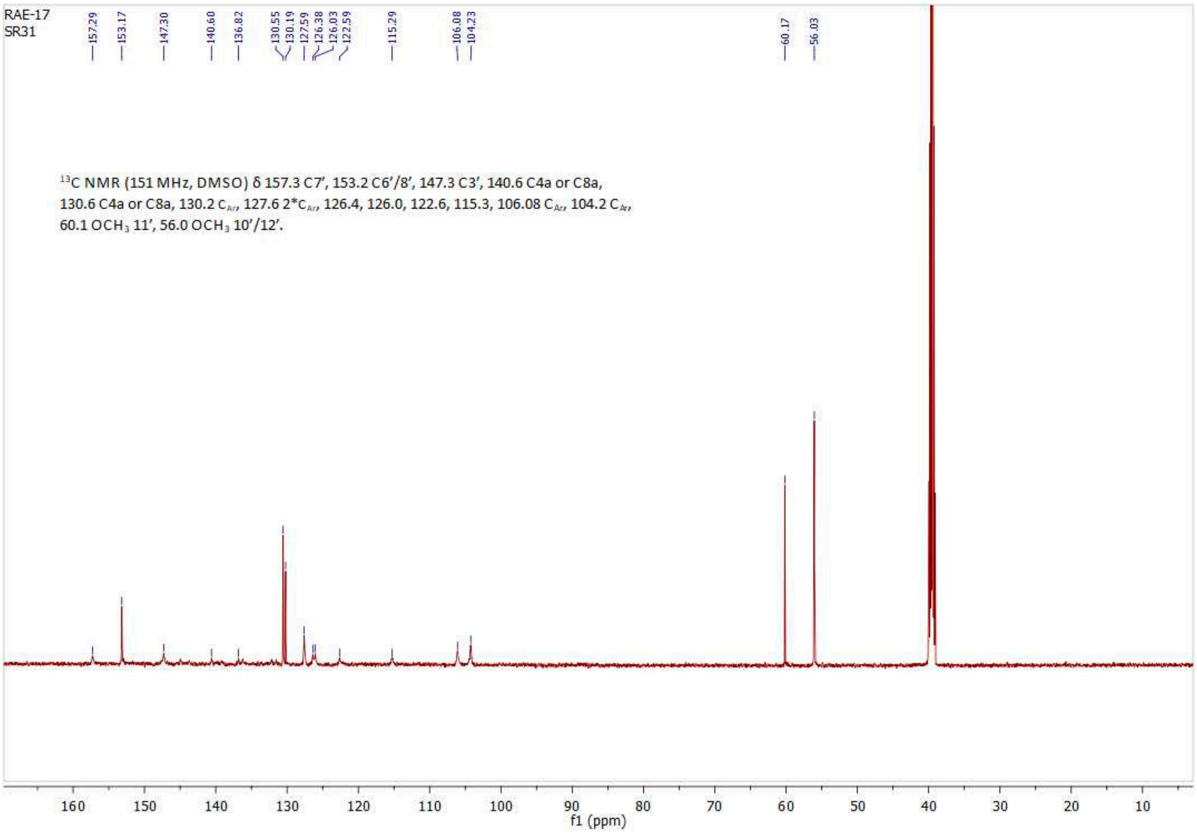

*S11. 2-chloro-3-(2-(pyridin-3-ylmethylene)hydrazinyl)quinoxaline (**14e**).*

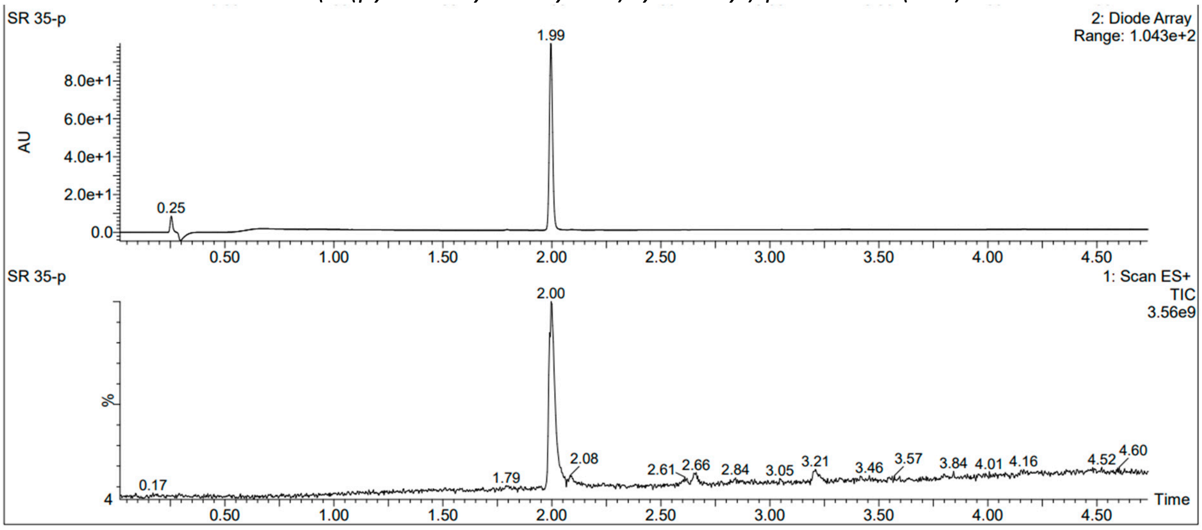

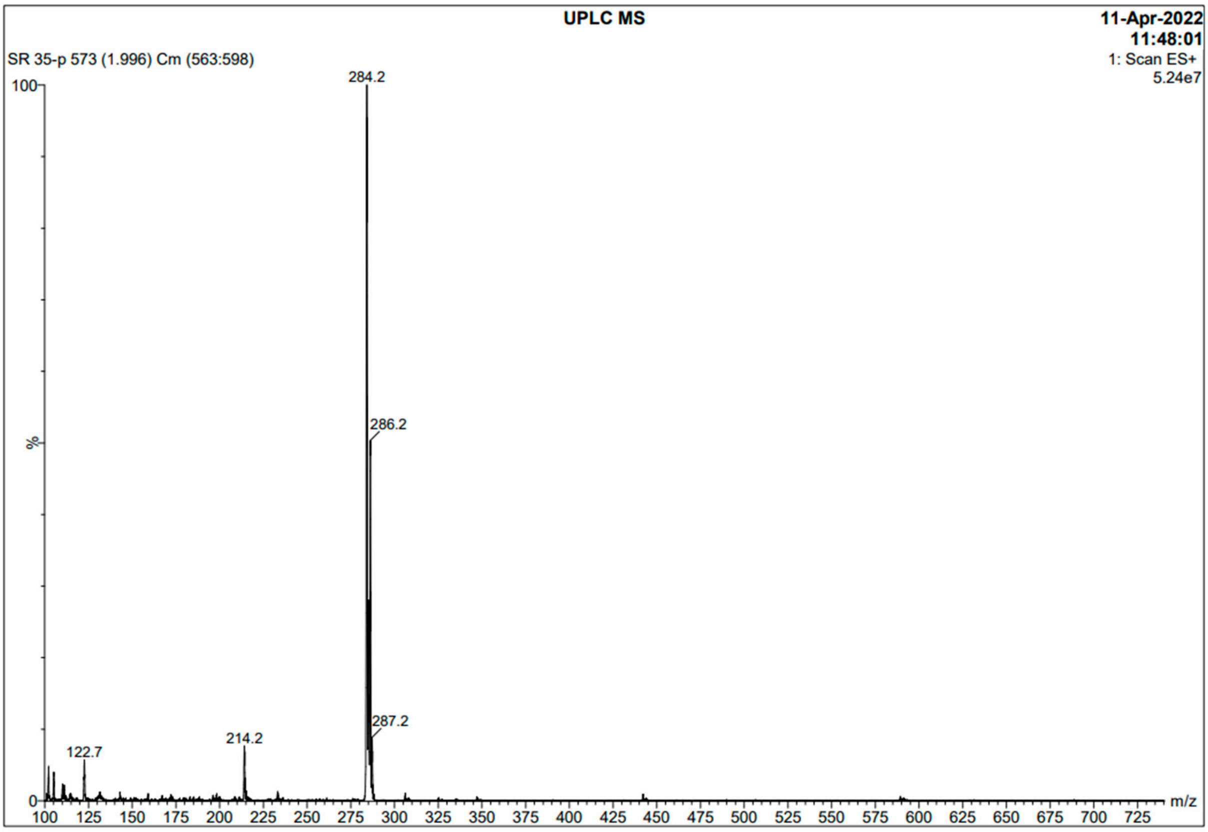
UPLC MS
11-Apr-2022
11:48:01
SR 35-p 573 (1.996) Cm (563:598)
1: Scan ES+
5.24e7
284.2
286.2
287.2
214.2
122.7
%
m/z

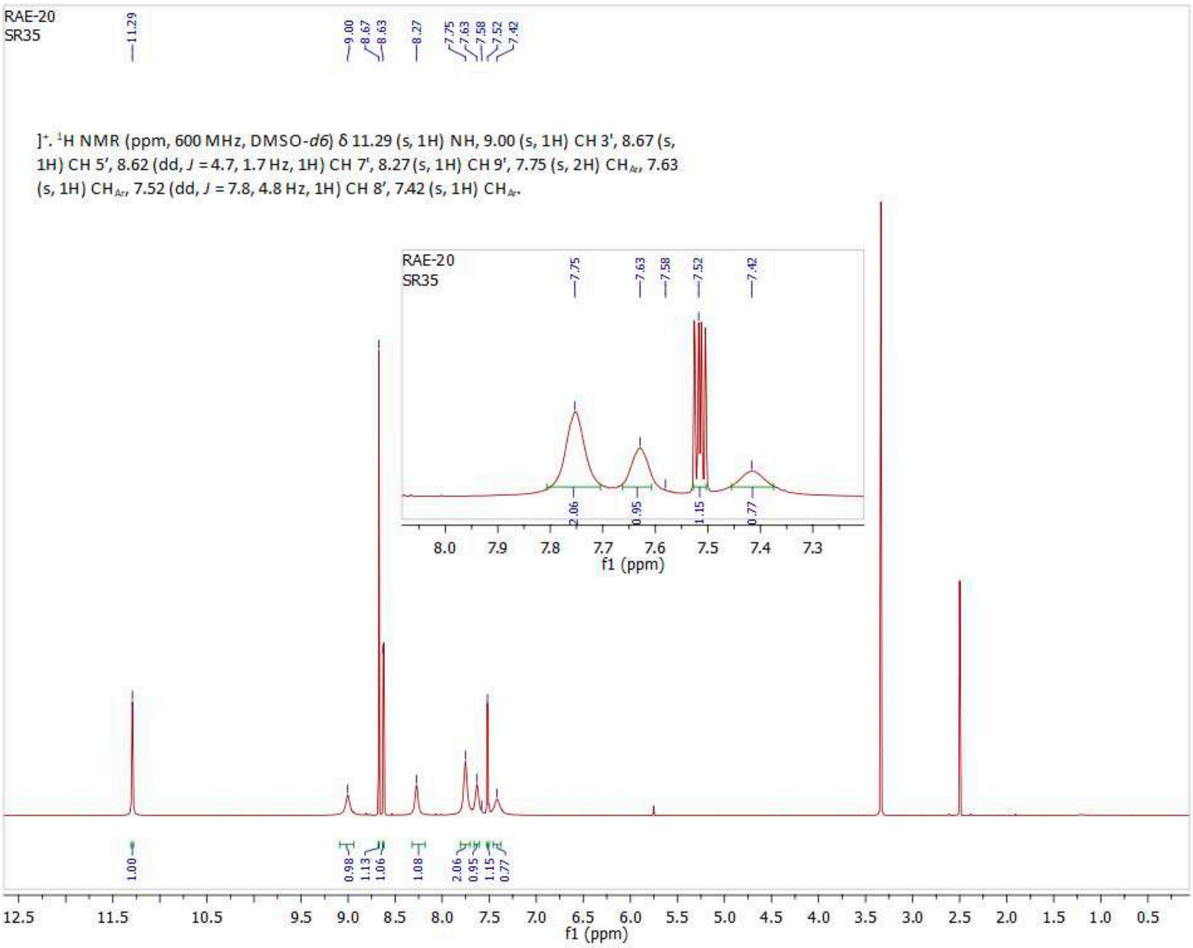
RAE-20
SR35
]+. 1H NMR (ppm, 600 MHz, DMSO-d6) δ 11.29 (s, 1H) NH, 9.00 (s, 1H) CH 3', 8.67 (s, 1H) CH 5', 8.62 (dd, J = 4.7, 1.7 Hz, 1H) CH 7', 8.27 (s, 1H) CH 9', 7.75 (s, 2H) CHAr, 7.63 (s, 1H) CHAr, 7.52 (dd, J = 7.8, 4.8 Hz, 1H) CH 8', 7.42 (s, 1H) CHAr.
f1 (ppm)

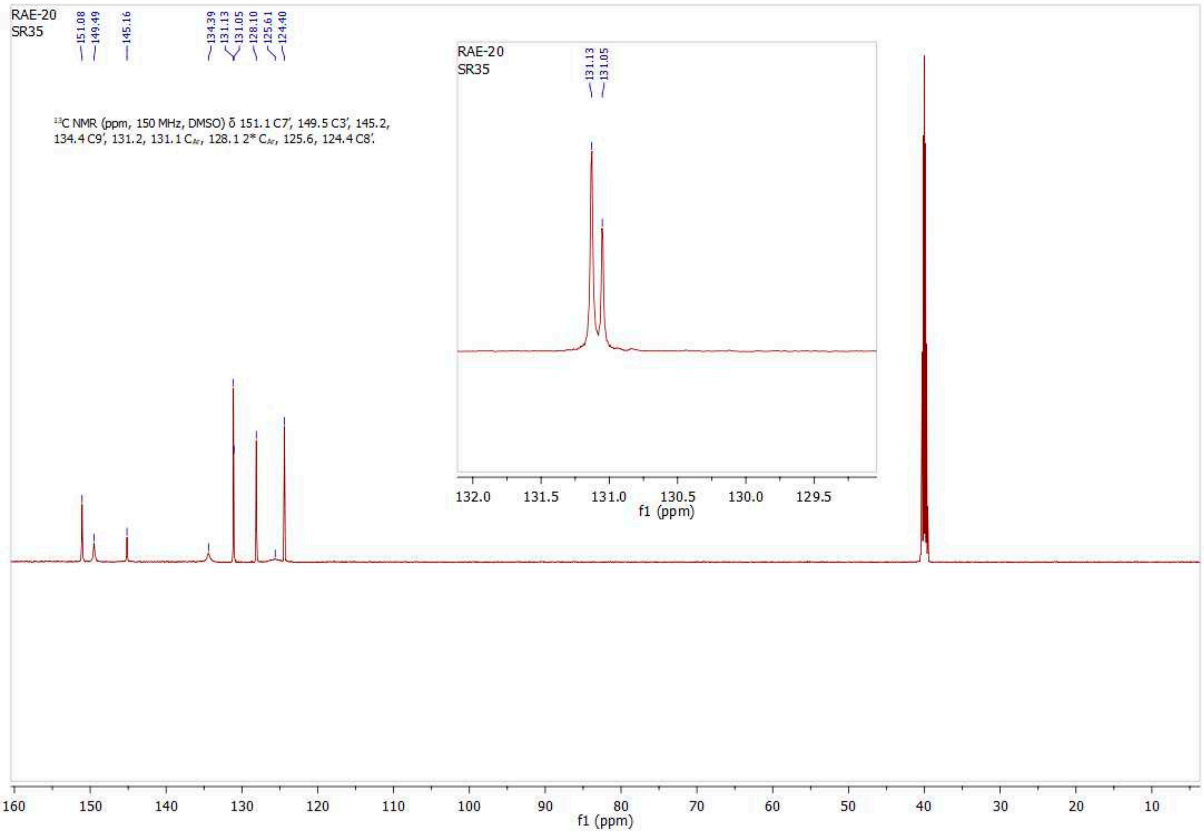

*S12. 2-chloro-3-(2-(3-phenylpropylidene)hydrazinyl)quinoxaline (**14f**).*

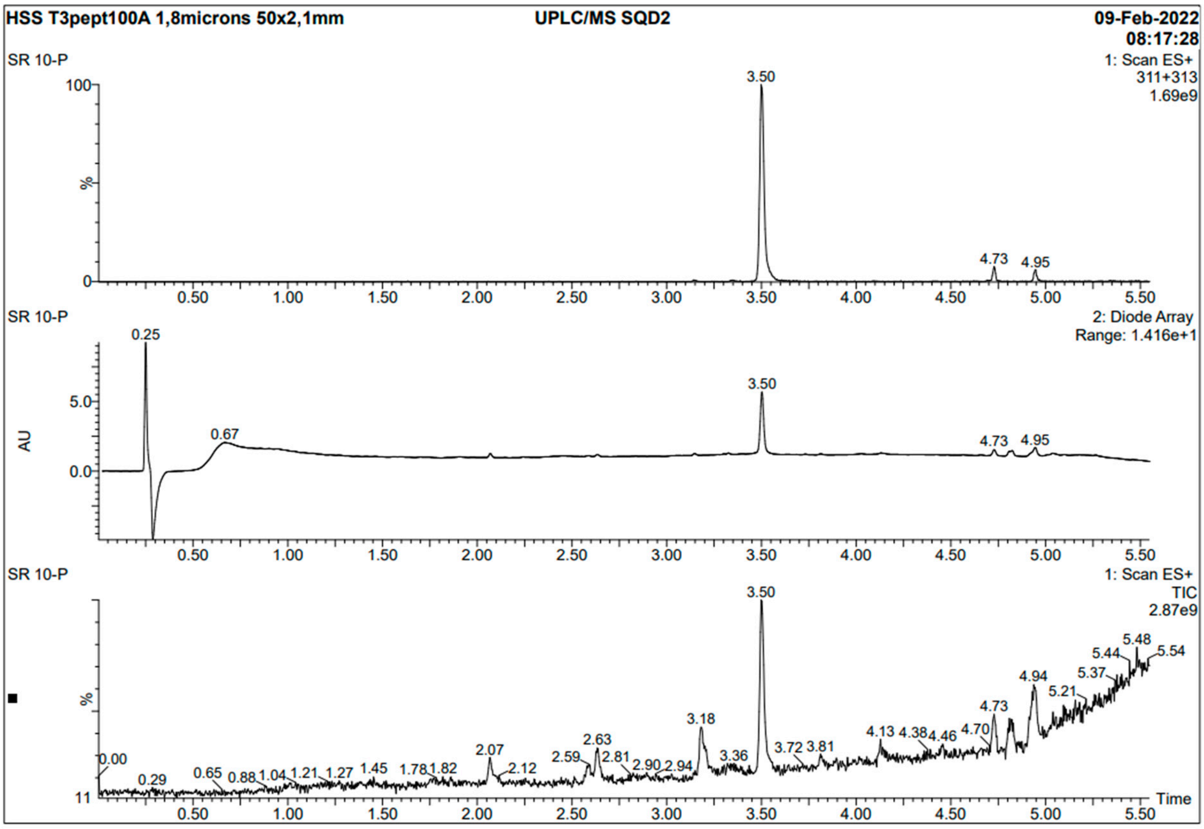

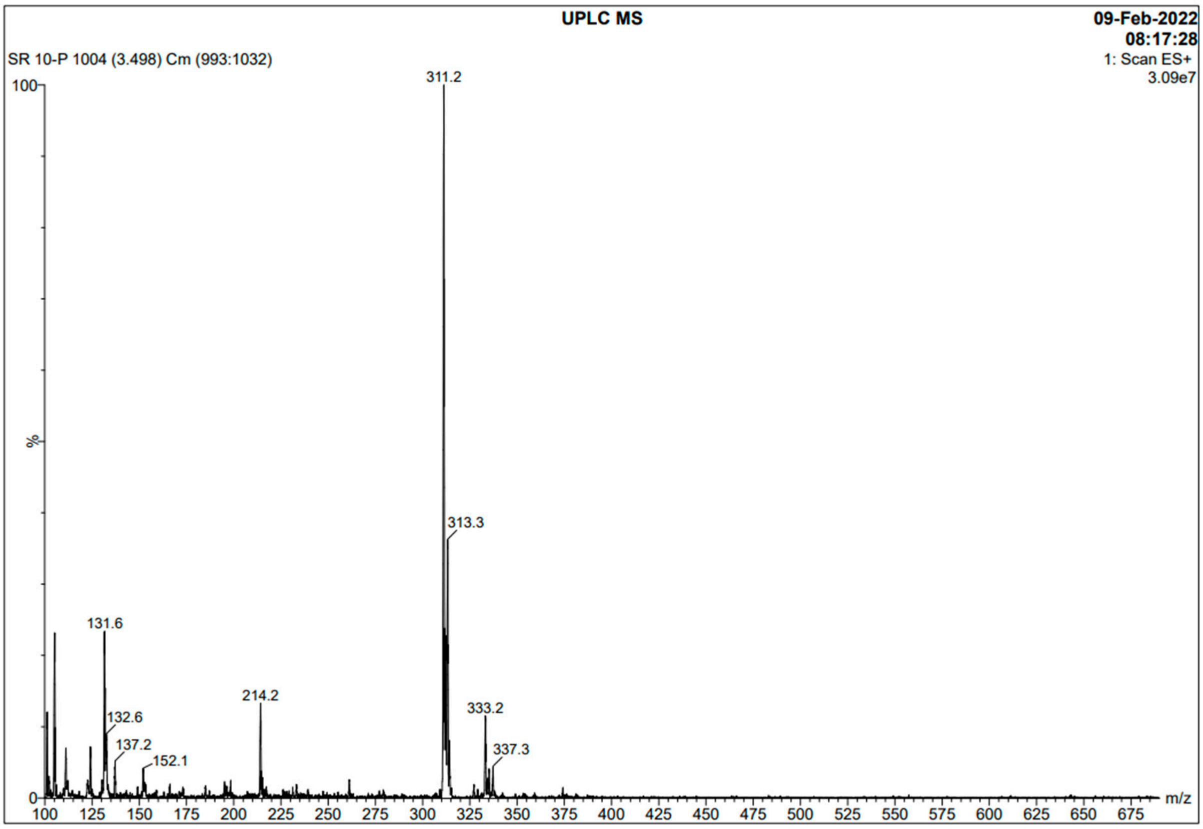
UPLC MS
09-Feb-2022
08:17:28
SR 10-P 1004 (3.498) Cm (993:1032)
1: Scan ES+
3.09e7
%
311.2
313.3
131.6
132.6
137.2
152.1
214.2
333.2
337.3
m/z

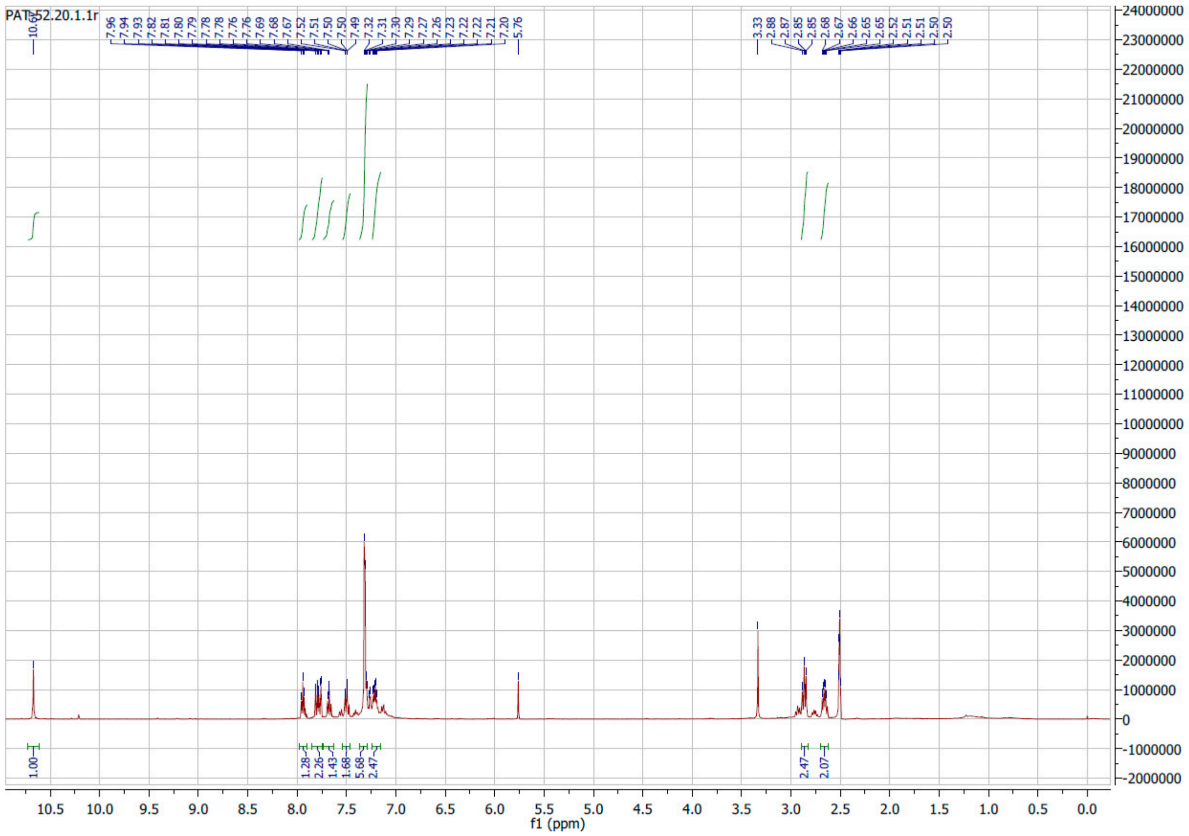
PAT-52.20.1.1r
f1 (ppm)

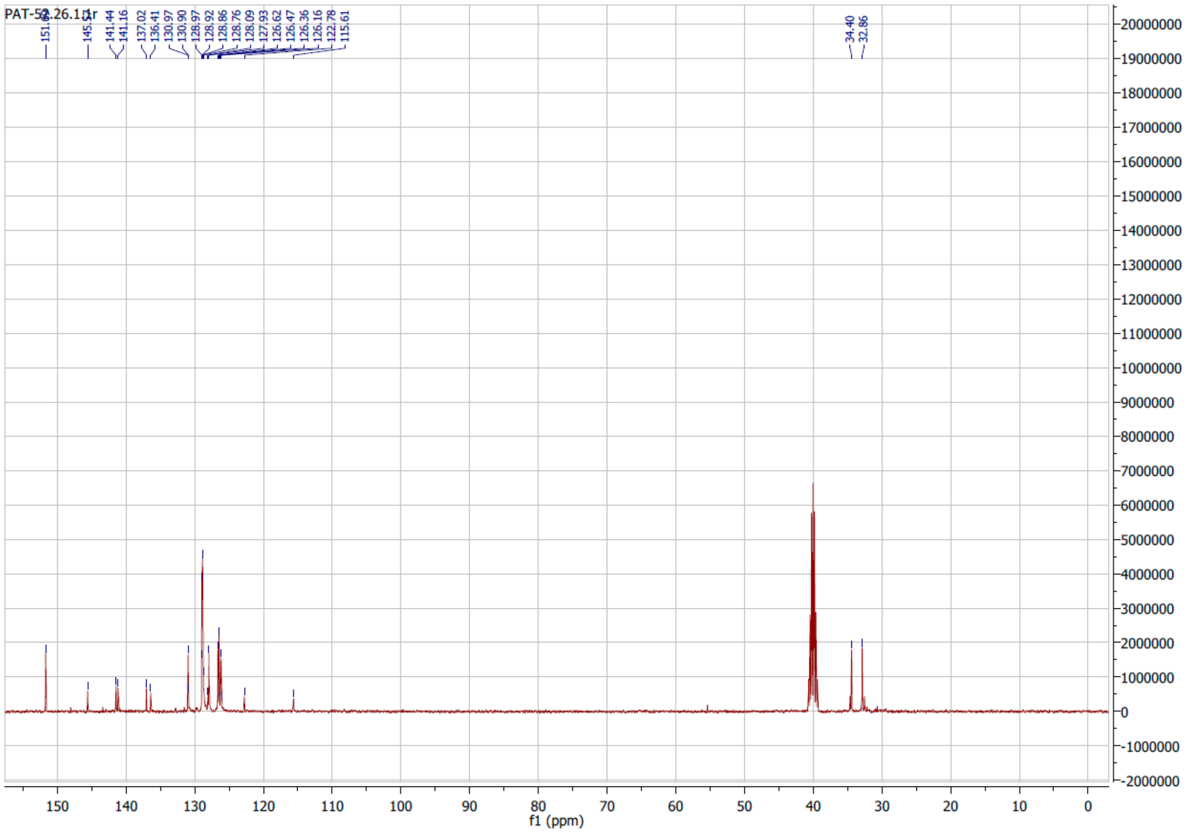

*S13. 2-chloro-3-(2-pentylidenehydrazinyl)quinoxaline (**14g**).*

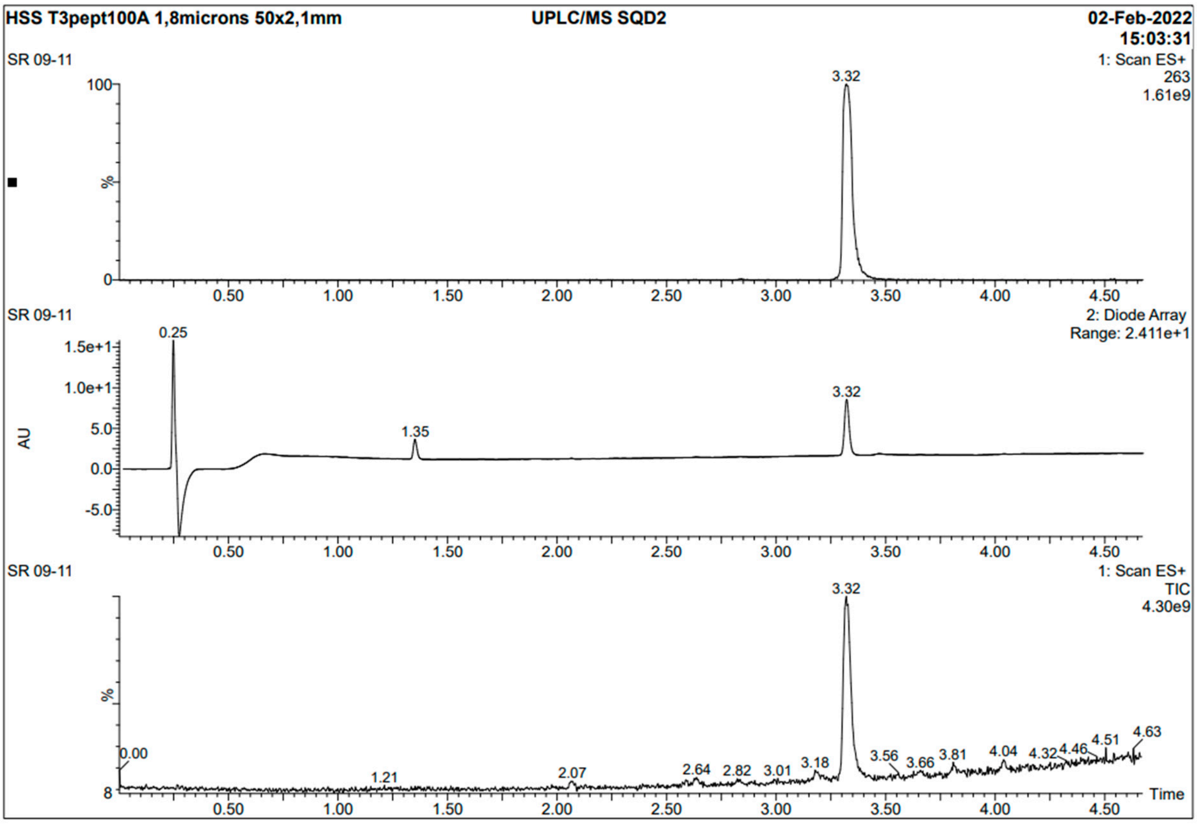

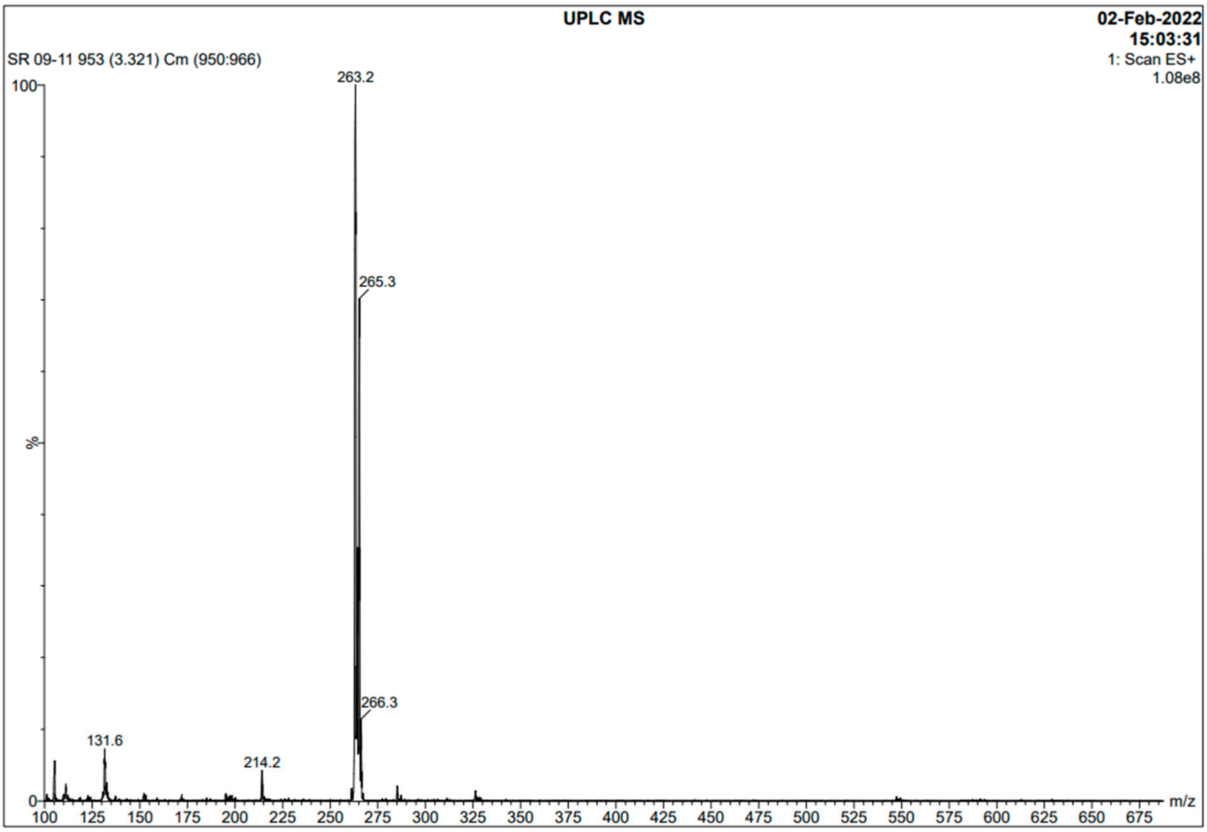
UPLC MS
02-Feb-2022
15:03:31
SR 09-11 953 (3.321) Cm (950:966)
1: Scan ES+
1.08e8
%
263.2
265.3
266.3
131.6
214.2
m/z

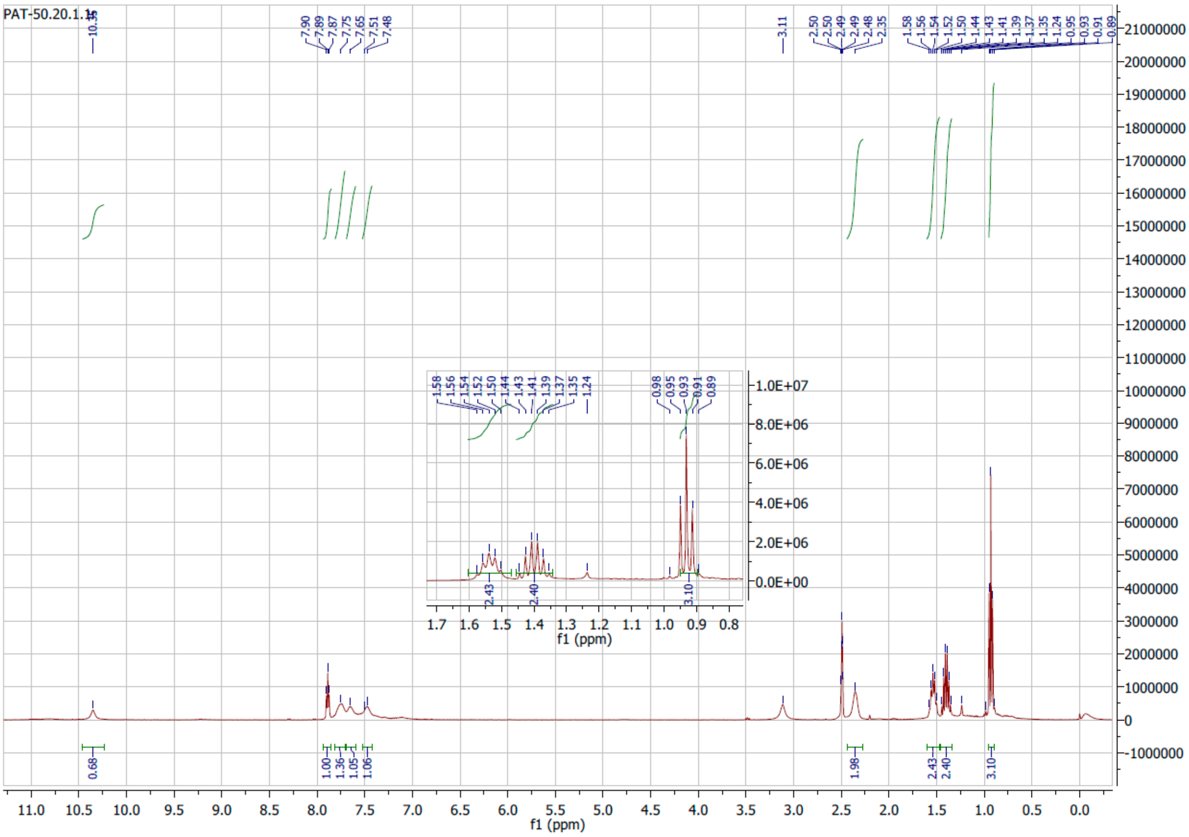
PAT-50.20.1.1
10.35
7.90
7.89
7.87
7.75
7.65
7.51
7.48
3.11
2.50
2.50
2.49
2.49
2.48
2.35
0.68
1.00
1.36
1.05
1.06
1.98
2.43
2.40
3.10
f1 (ppm)

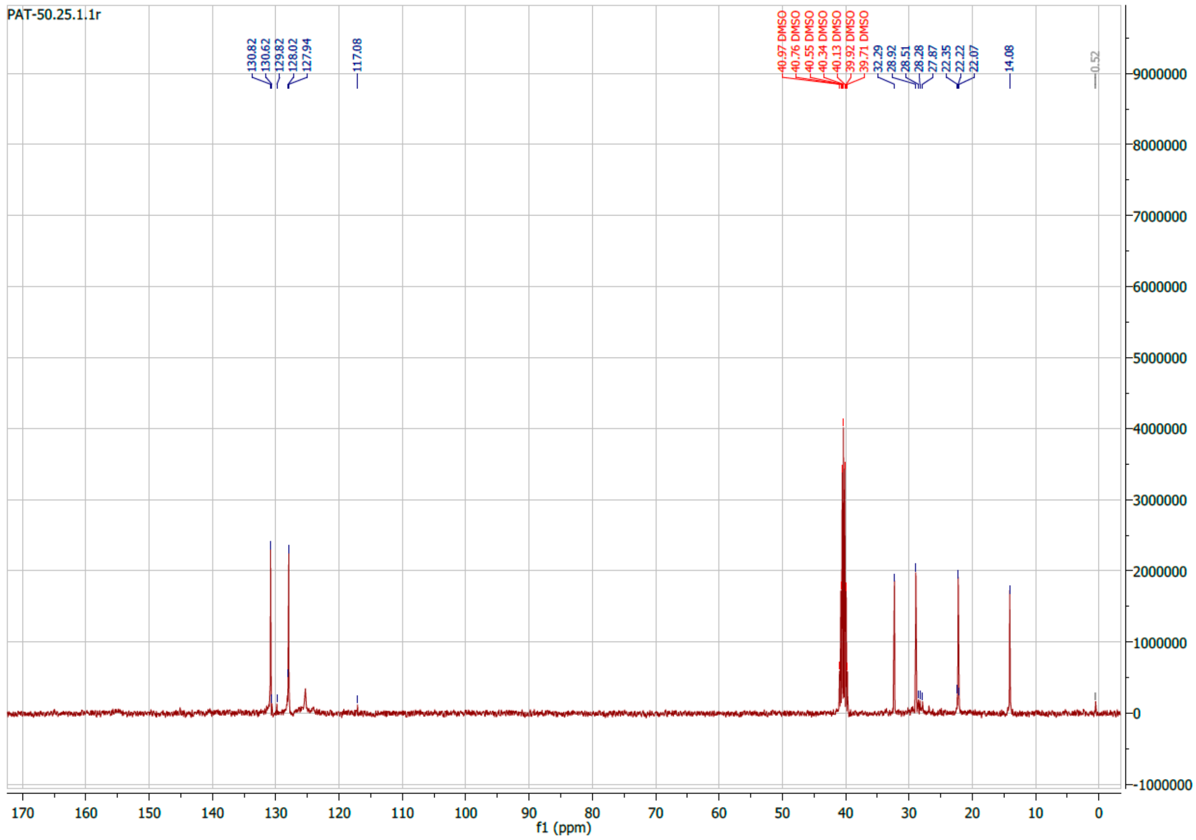

S14. 2-chloro-3-(2-(3-methylbutylidene)hydrazinyl)quinoxaline (**14h**).

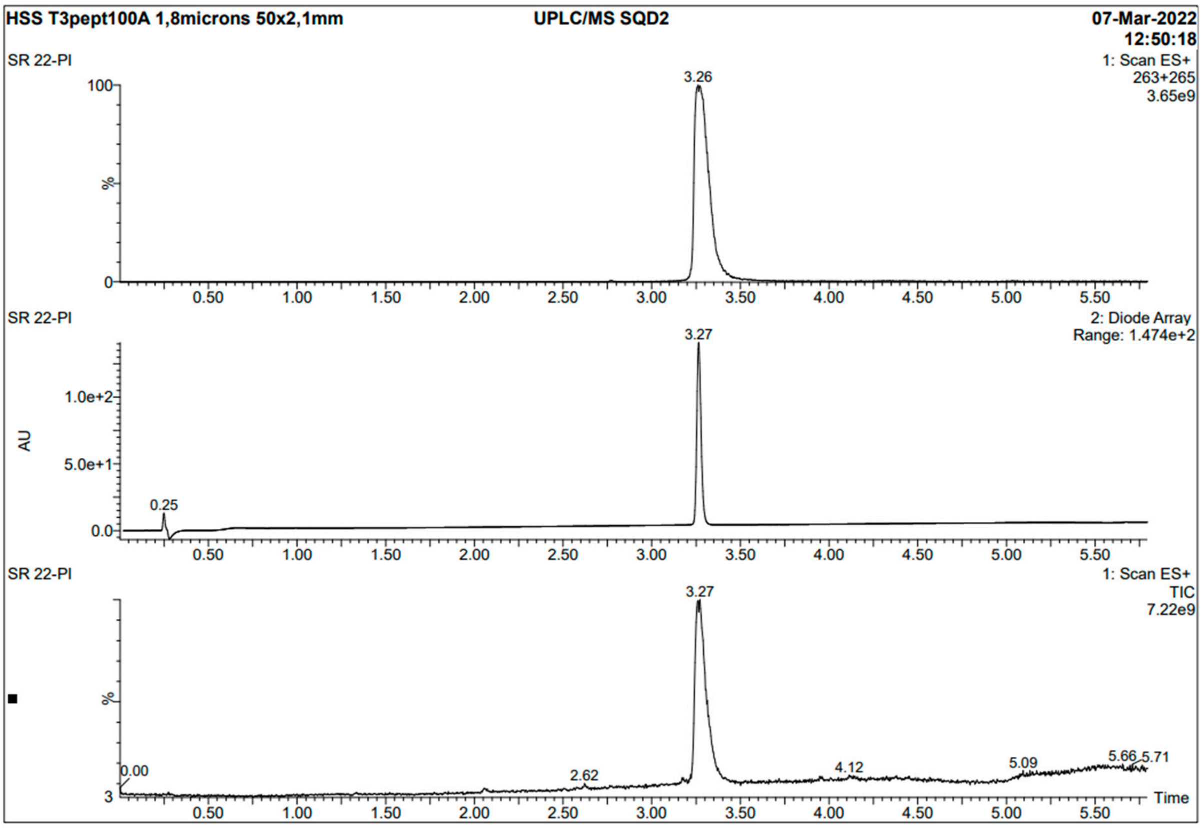

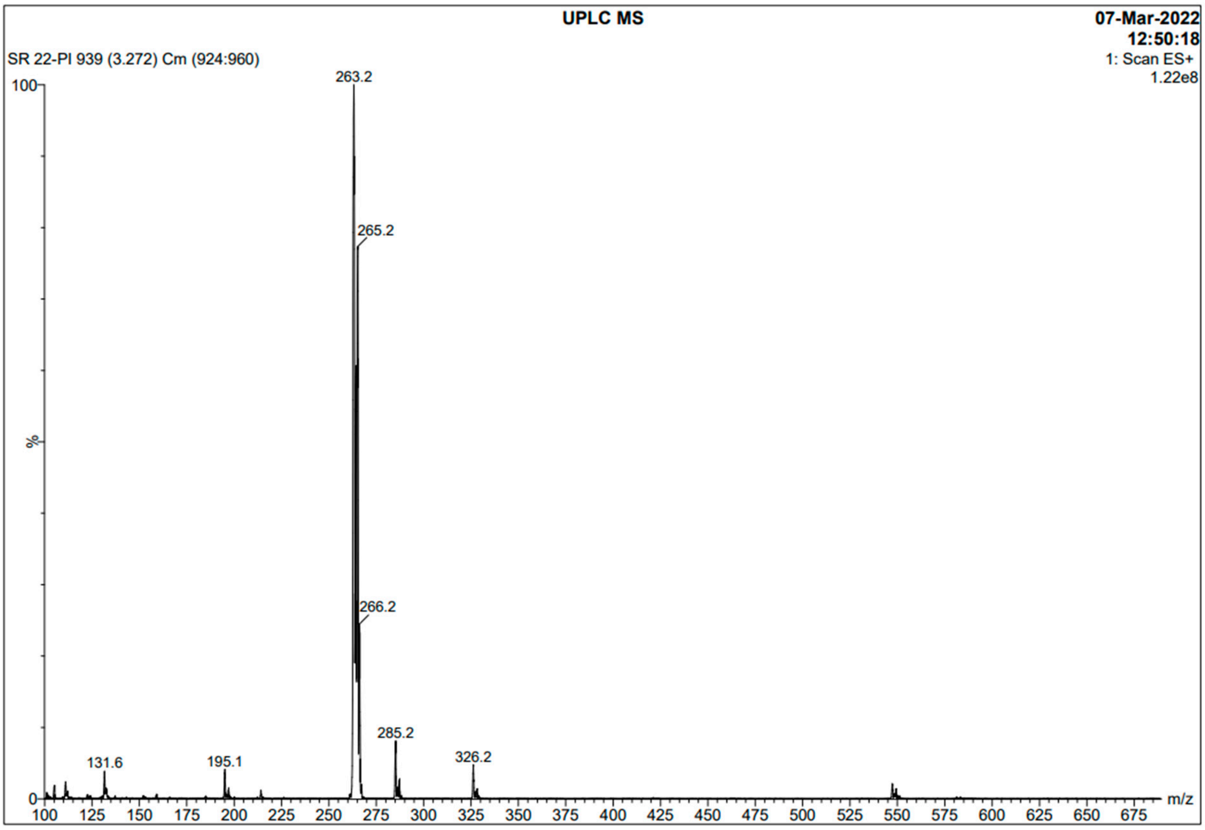
UPLC MS
07-Mar-2022
12:50:18
SR 22-PI 939 (3.272) Cm (924:960)
1: Scan ES+
1.22e8
263.2
265.2
266.2
285.2
326.2
131.6
195.1
%
m/z

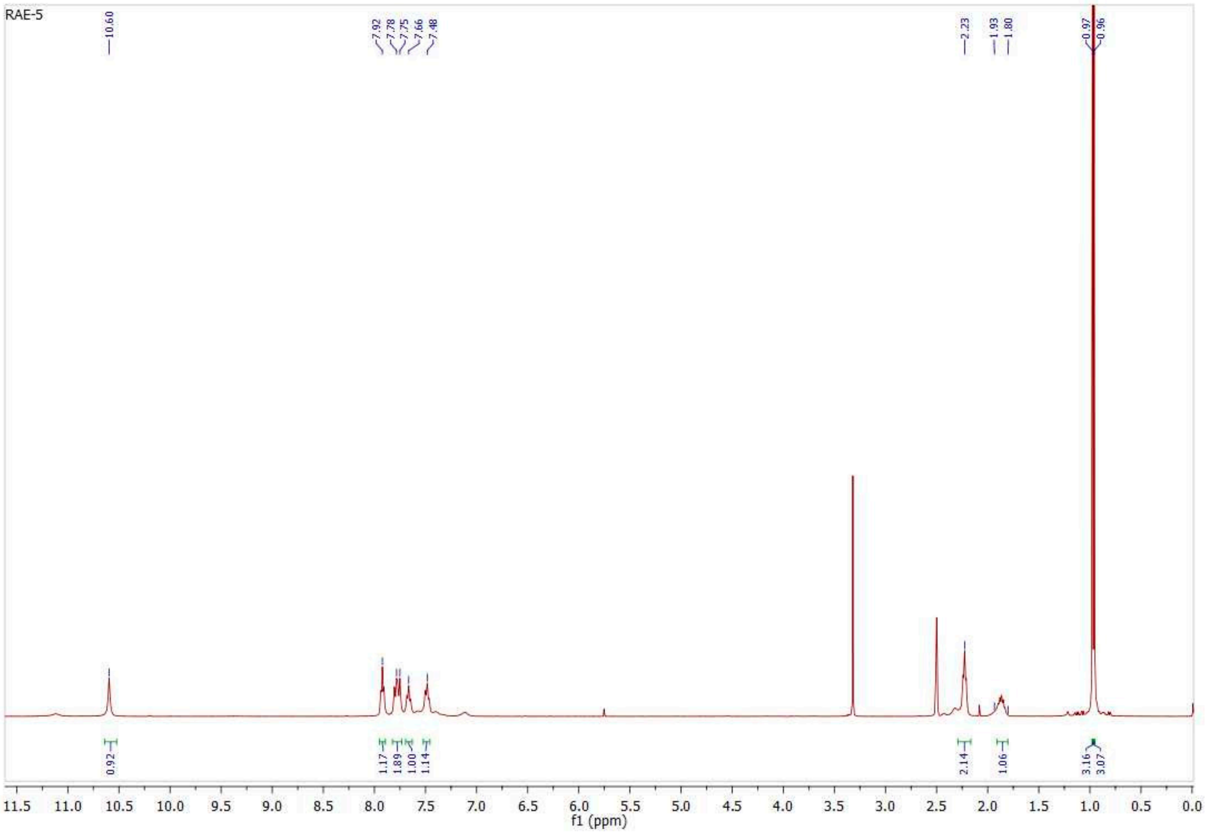
RAE-5
10.60
7.92
7.78
7.75
7.66
7.48
2.23
1.93
1.80
0.97
0.96
0.92
1.17
1.89
1.00
1.14
2.14
1.06
3.16
3.07
f1 (ppm)

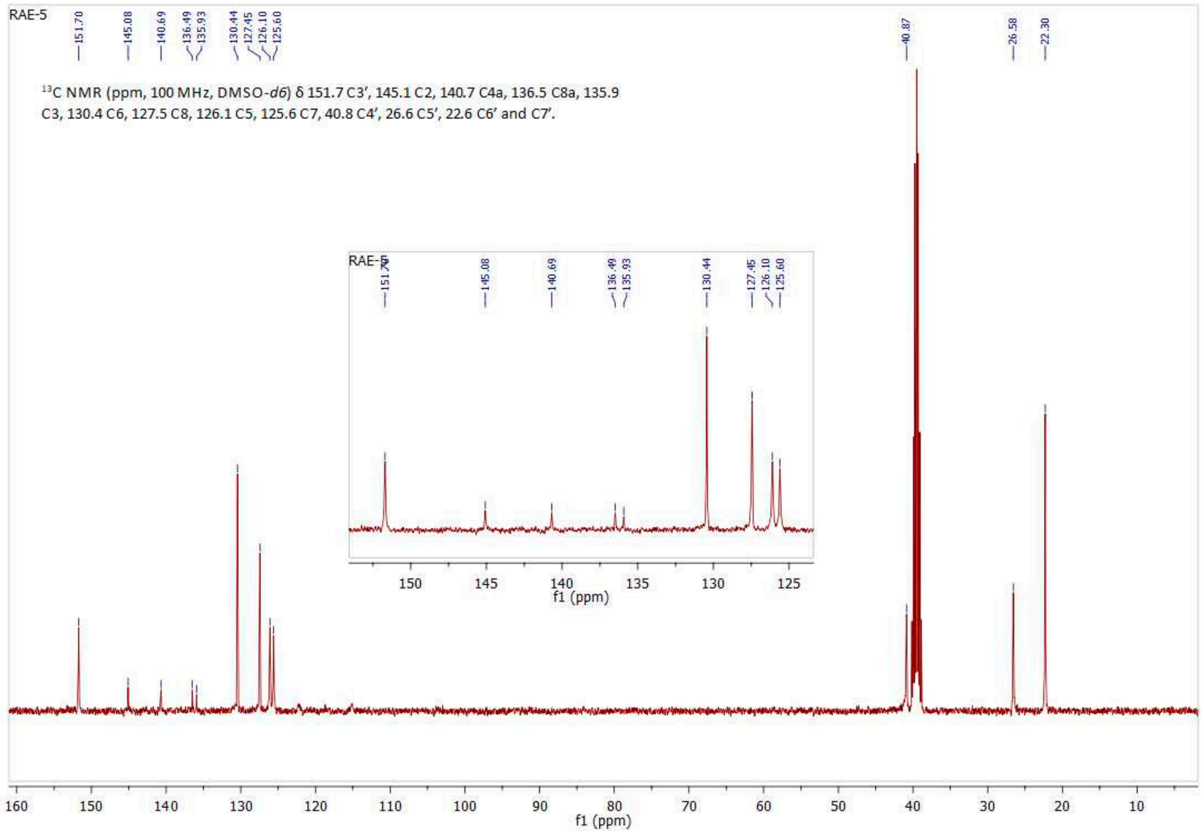

*S15. 2-chloro-3-(2-ethylidenehydrazinyl)quinoxaline (**14i**).*

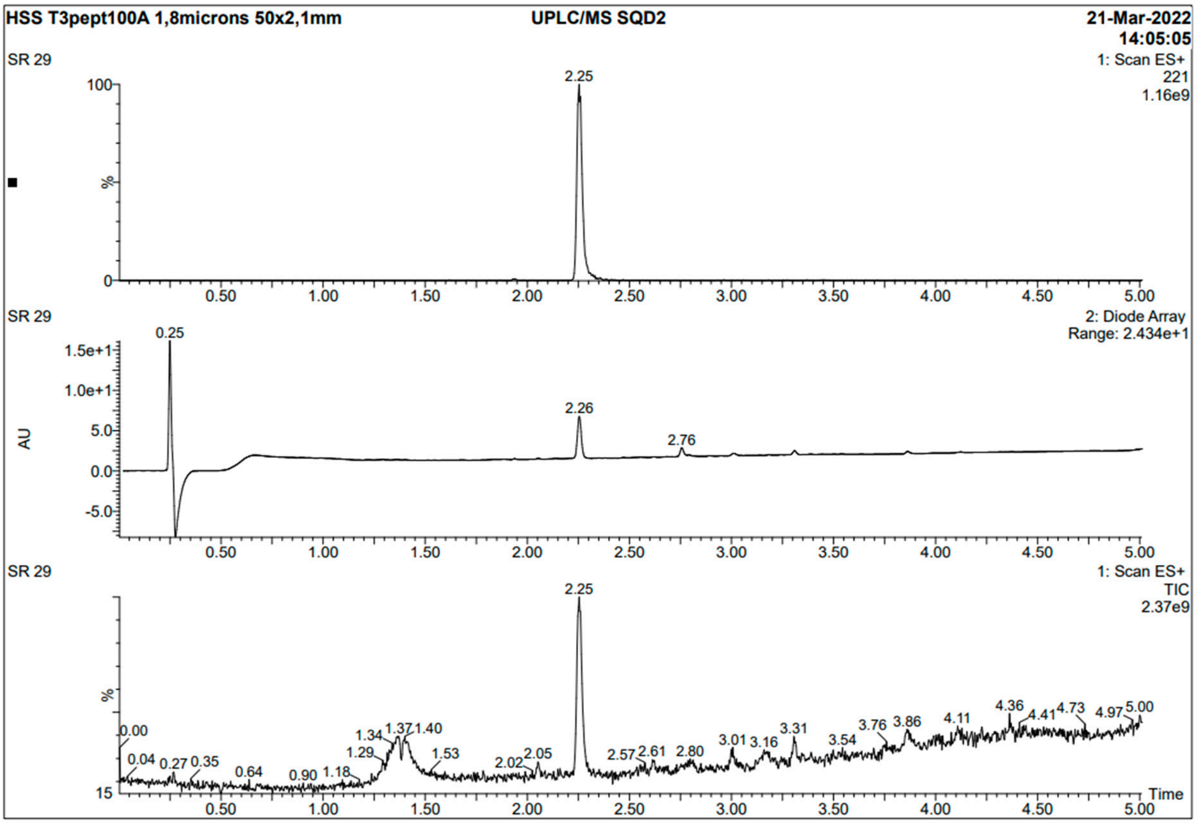

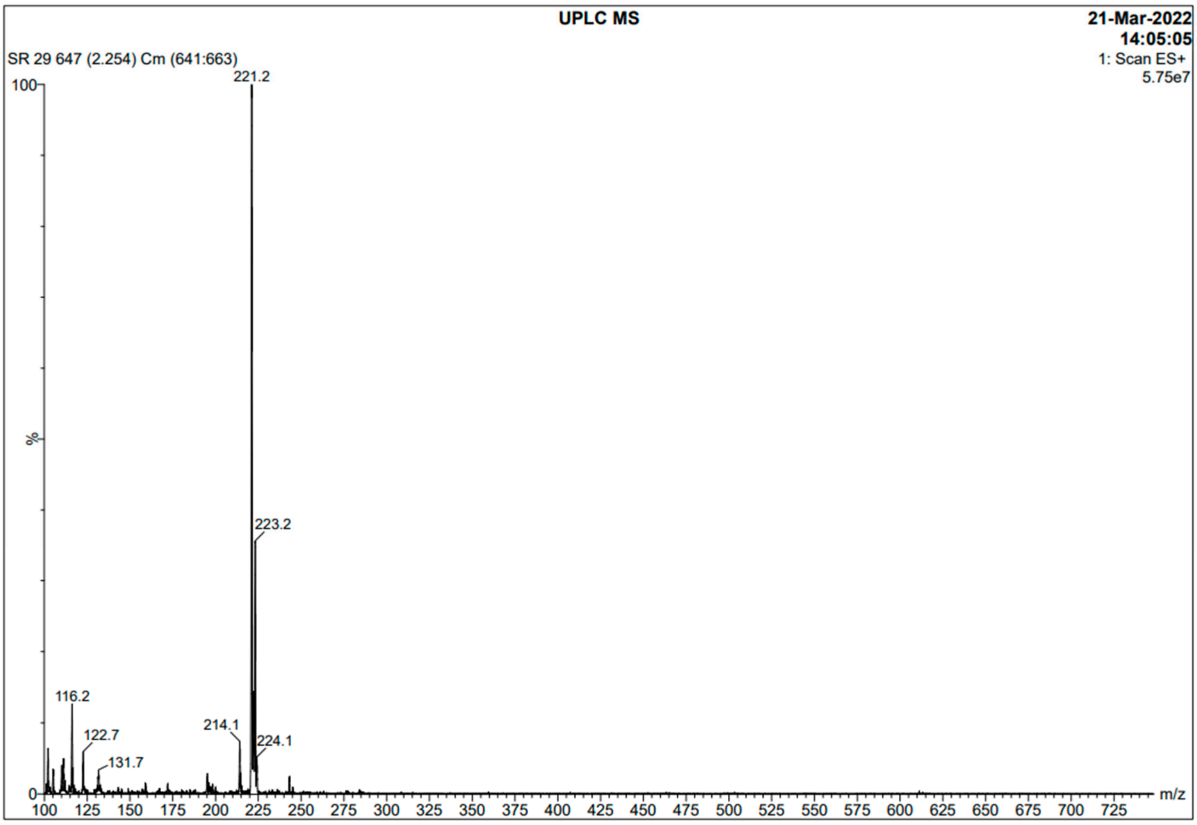

*S16. 4-chloro-1-(3,4-dimethoxyphenyl)-[1,2,4]triazolo[4,3-a]quinoxaline (**15a**).*

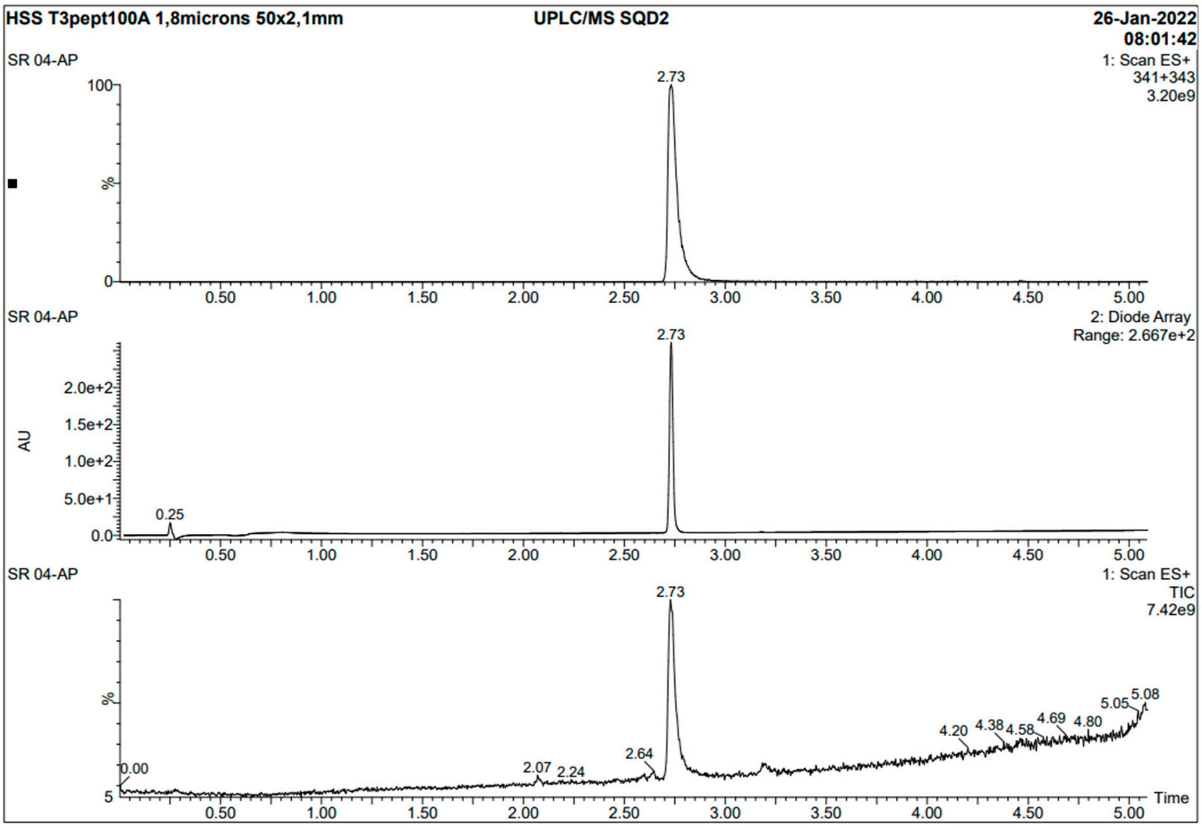

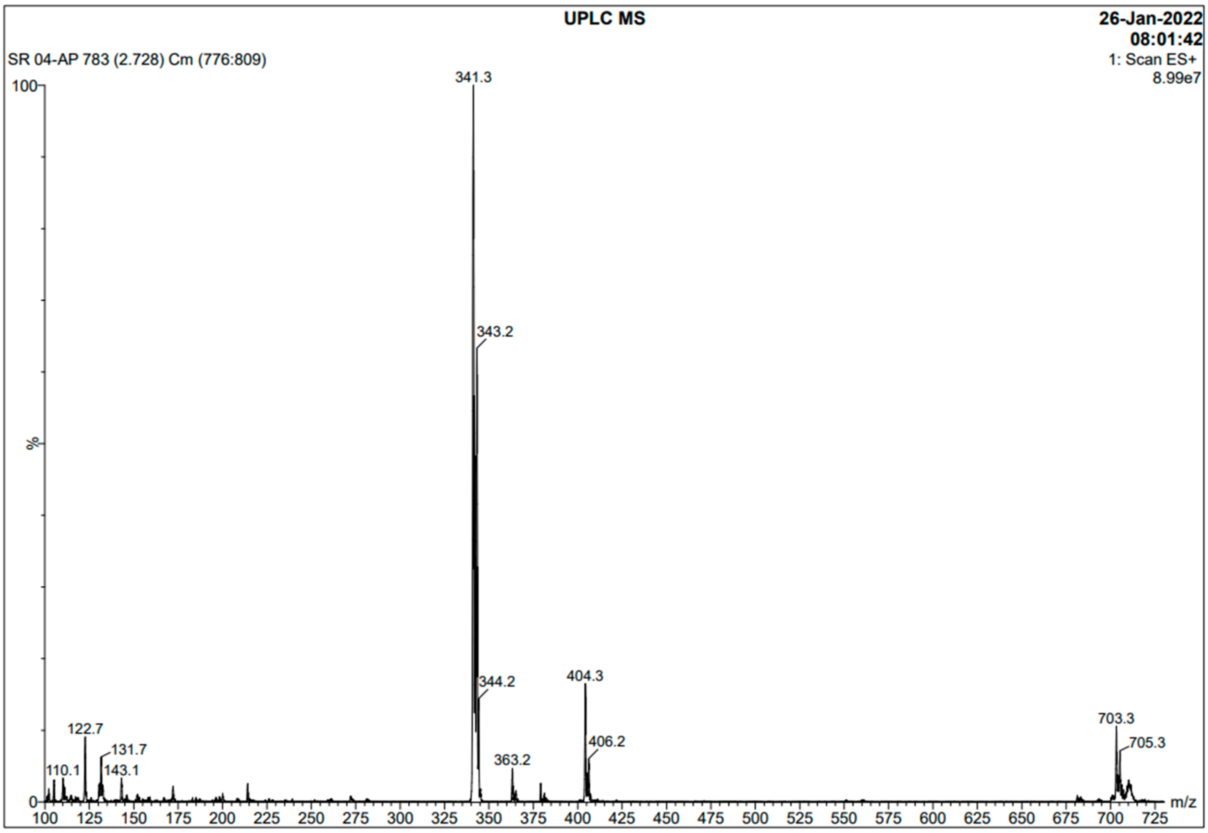
UPLC MS
26-Jan-2022
08:01:42
SR 04-AP 783 (2.728) Cm (776:809)
1: Scan ES+
8.99e7
341.3
343.2
344.2
404.3
406.2
363.2
122.7
131.7
143.1
110.1
703.3
705.3
%
m/z
100
0

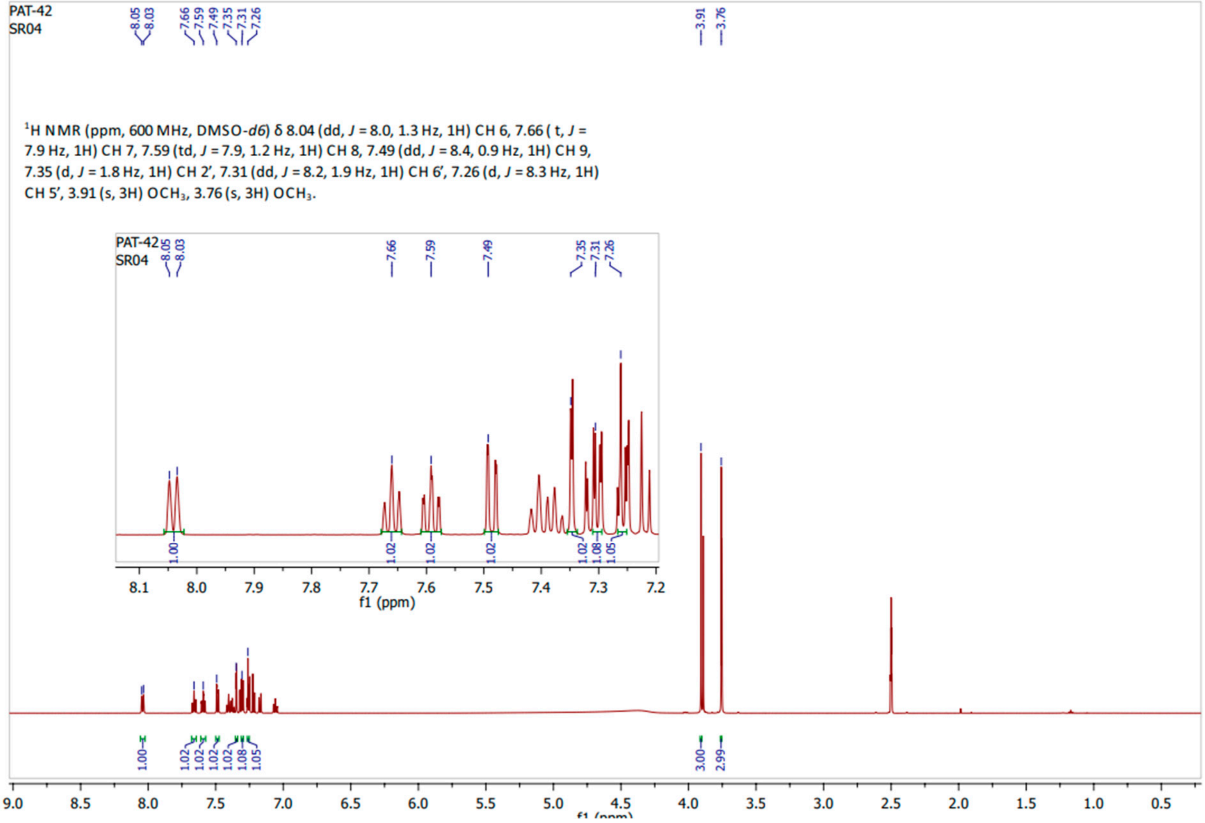
PAT-42
SR04
¹H NMR (ppm, 600 MHz, DMSO-d6) δ 8.04 (dd, J = 8.0, 1.3 Hz, 1H) CH 6, 7.66 (t, J = 7.9 Hz, 1H) CH 7, 7.59 (td, J = 7.9, 1.2 Hz, 1H) CH 8, 7.49 (dd, J = 8.4, 0.9 Hz, 1H) CH 9, 7.35 (d, J = 1.8 Hz, 1H) CH 2', 7.31 (dd, J = 8.2, 1.9 Hz, 1H) CH 6', 7.26 (d, J = 8.3 Hz, 1H) CH 5', 3.91 (s, 3H) OCH₃, 3.76 (s, 3H) OCH₃.
f1 (ppm)

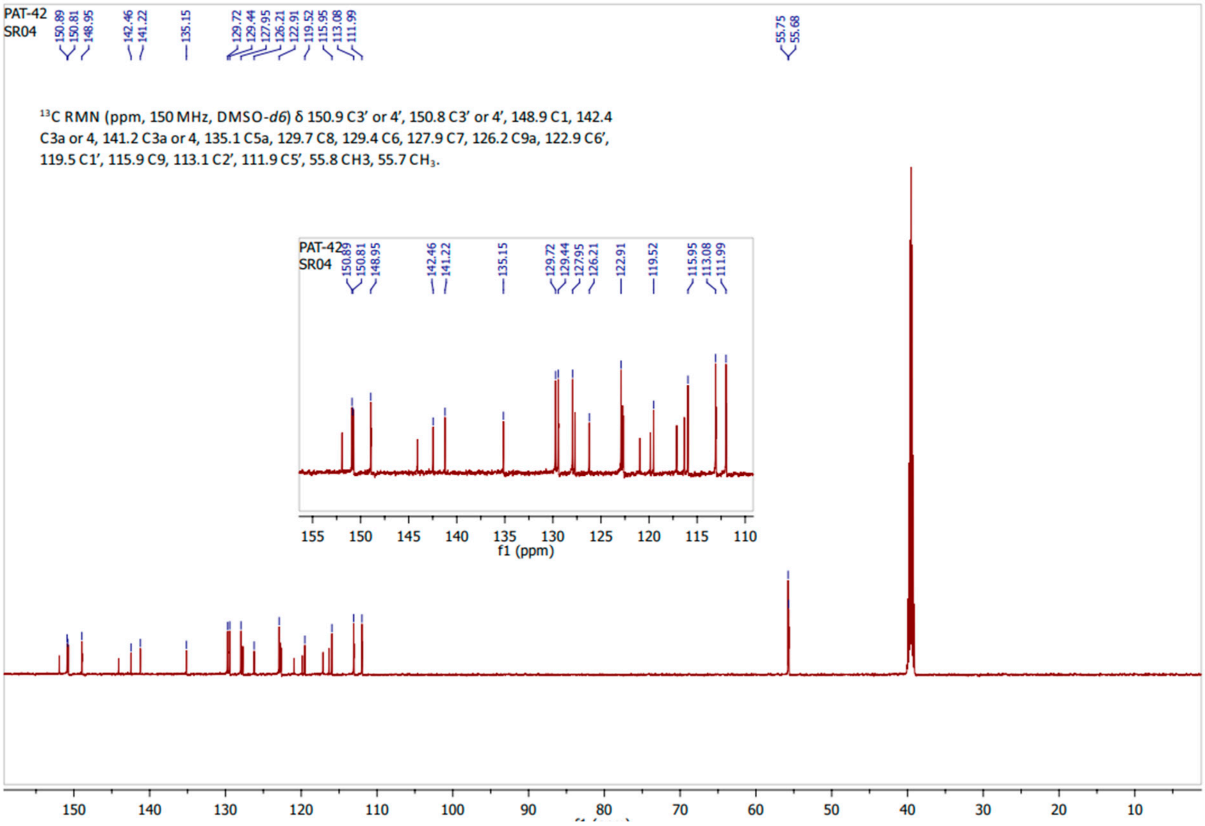

*S17. 4-chloro-1-(3-methoxyphenyl)-[1,2,4]triazolo[4,3-a]quinoxaline (**15b**).*

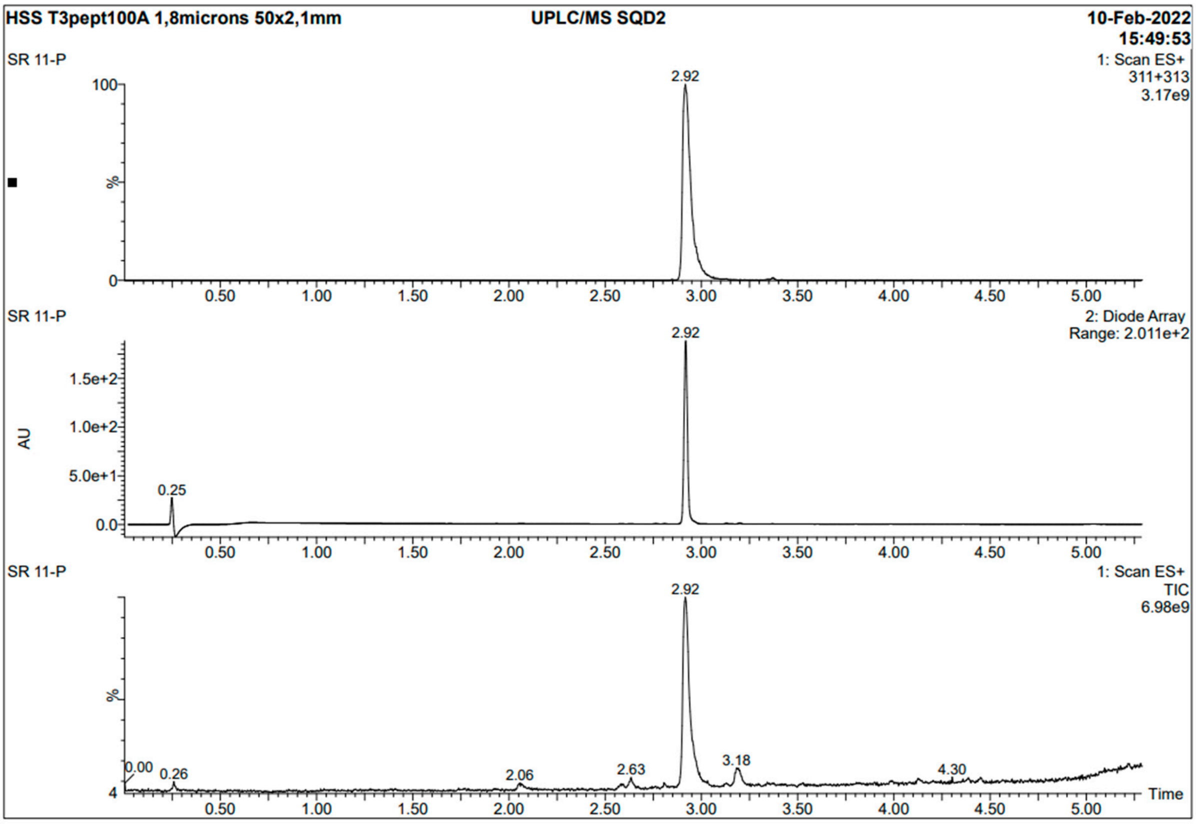

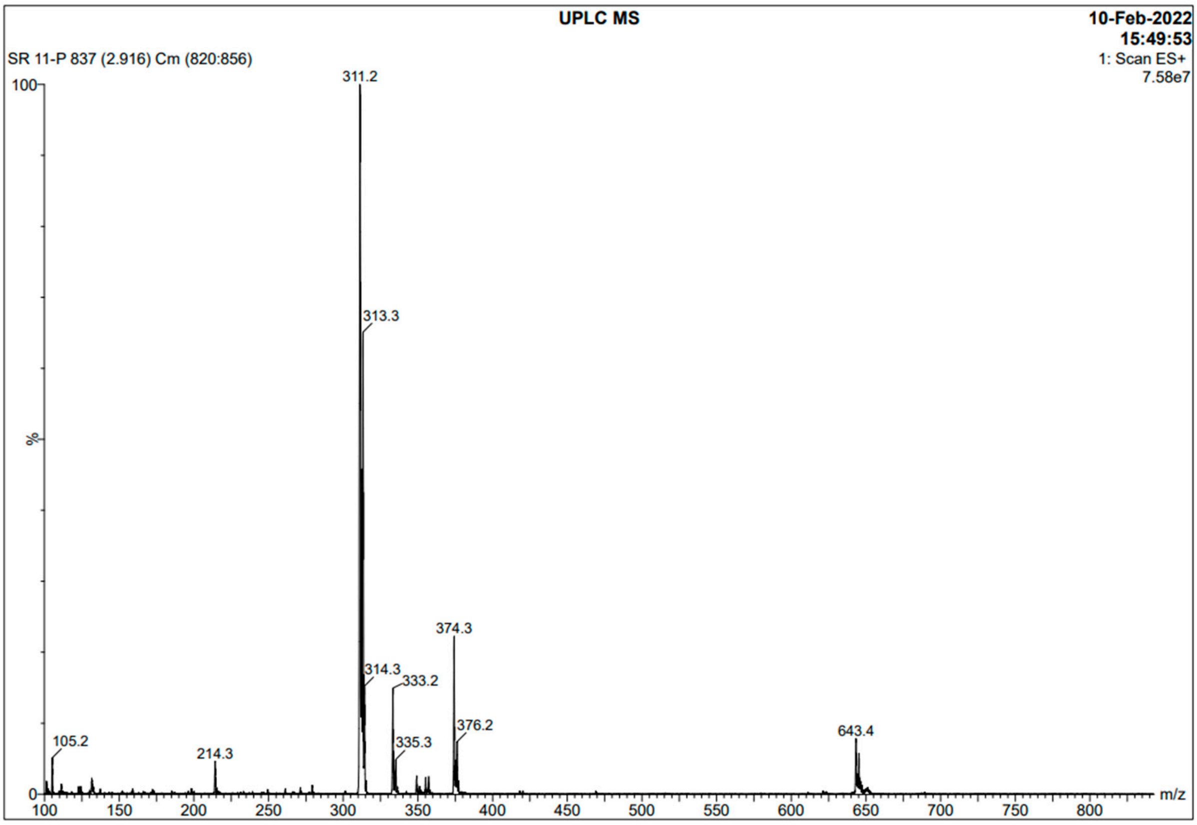

^{1}H NMR (400 MHz, DMSO) δ 8.04 (dd, J = 7.4, 0.7 Hz, 1H), 7.66 (t, J = 7.7 Hz, 1H), 7.62 (t, J = 7.7 Hz, 1H), 7.58 (t, J = 7.4 Hz, 1H), 7.40 (d, J = 8.4 Hz, 1H), 7.34 (s, 1H), 7.31 (dd, J = 7.9, 3.0 Hz, 2H), 3.83 (s, 3H).

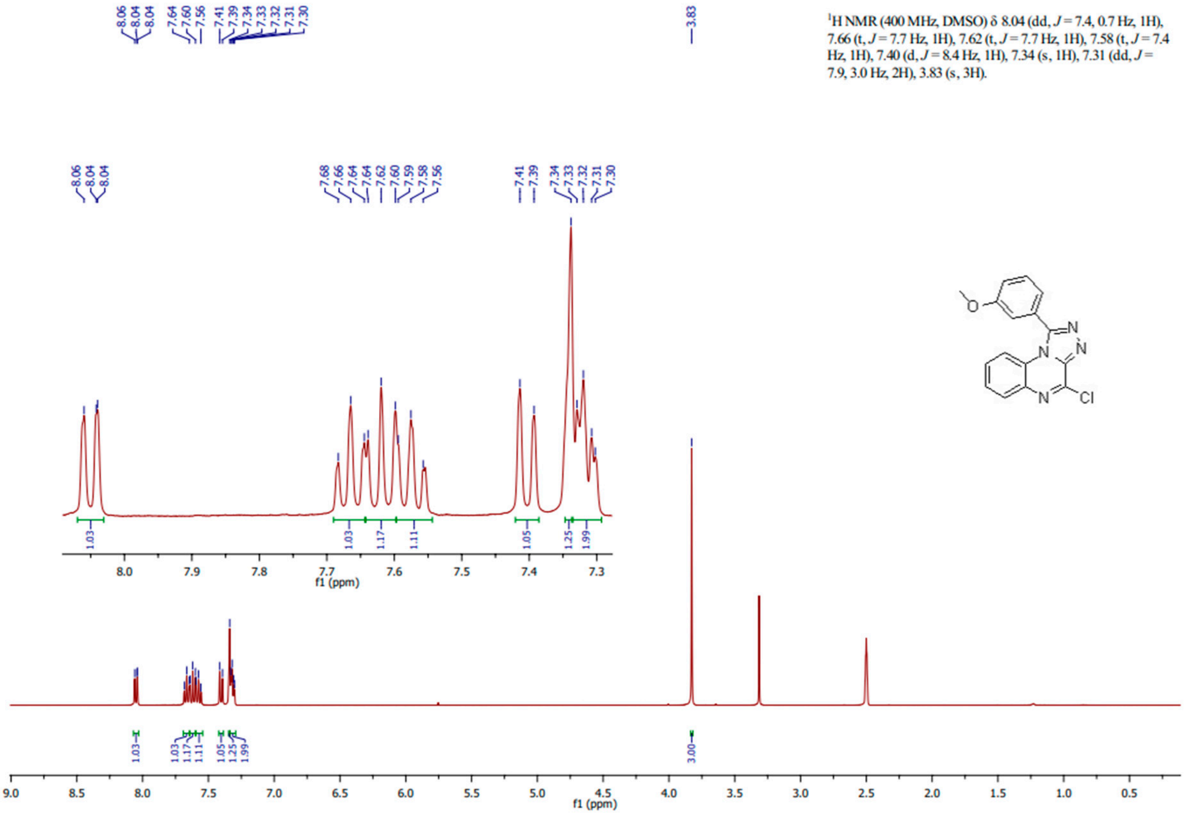

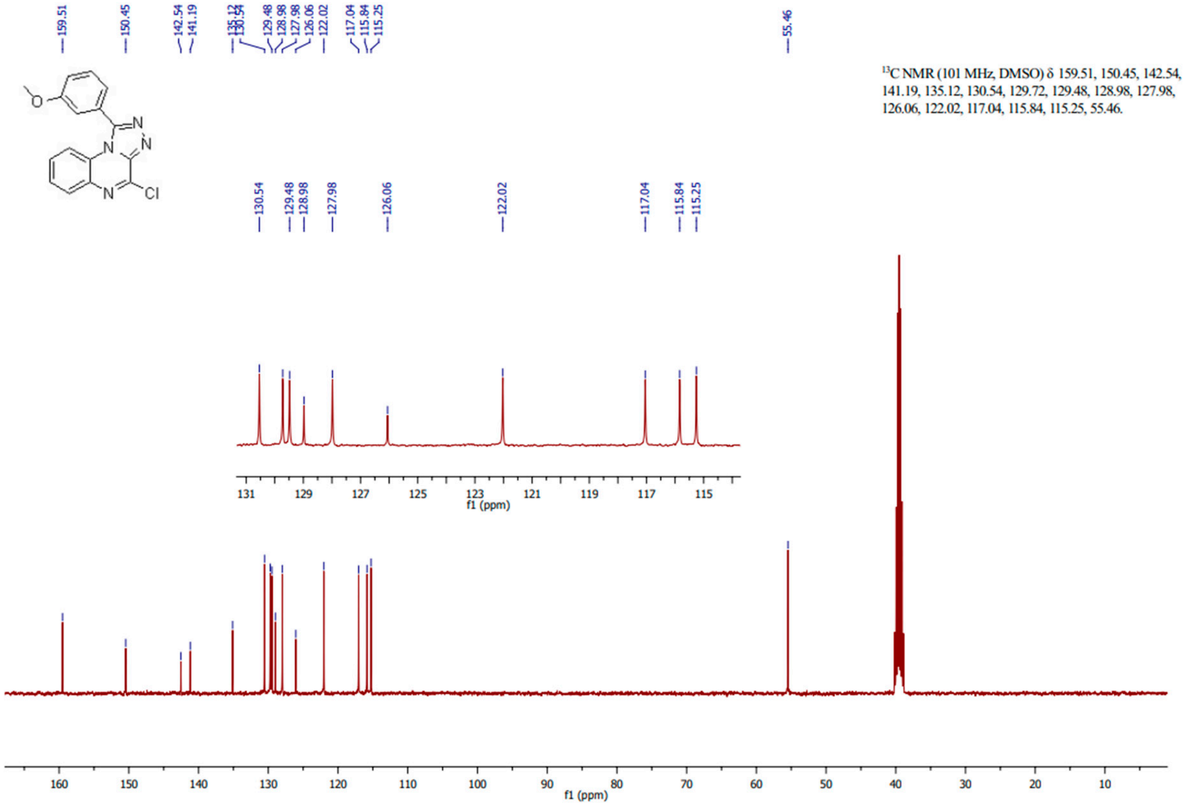

*S18. 4-chloro-1-phenyl-[1,2,4]triazolo[4,3-a]quinoxaline (**15c**).*

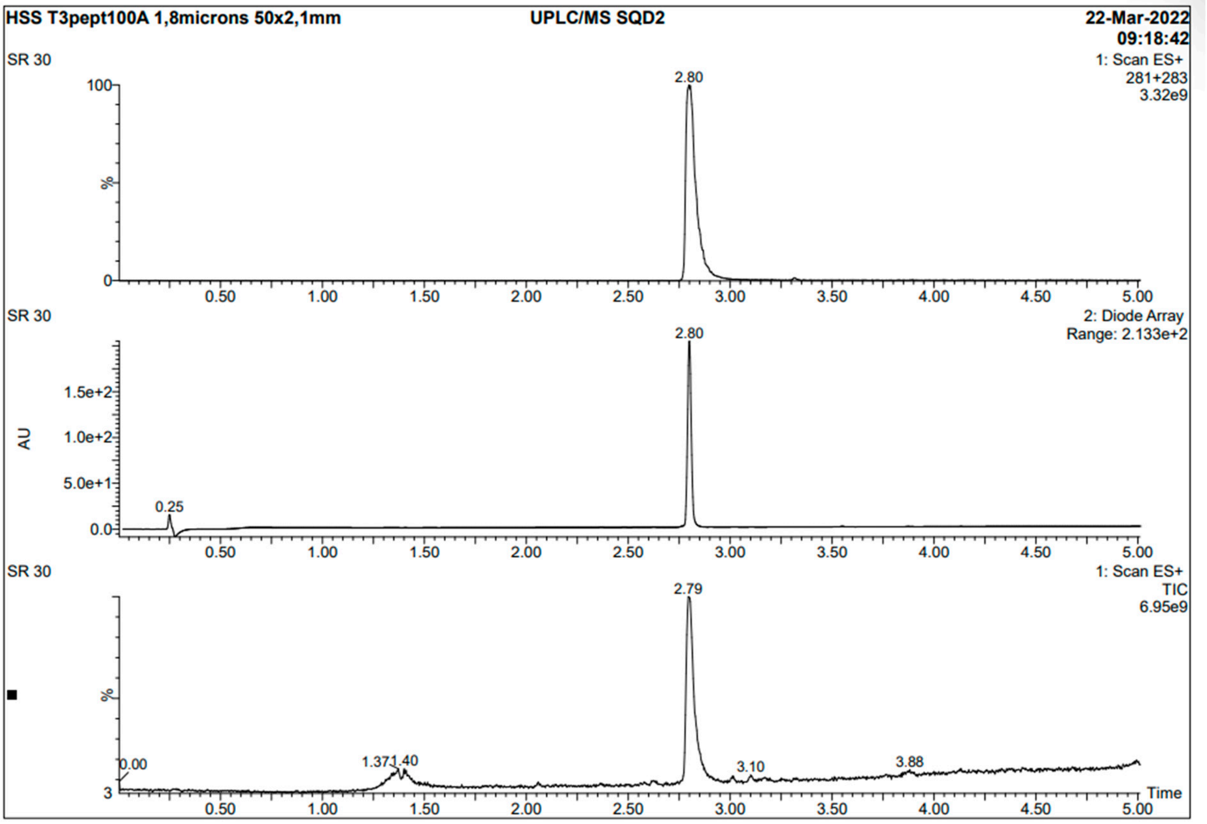

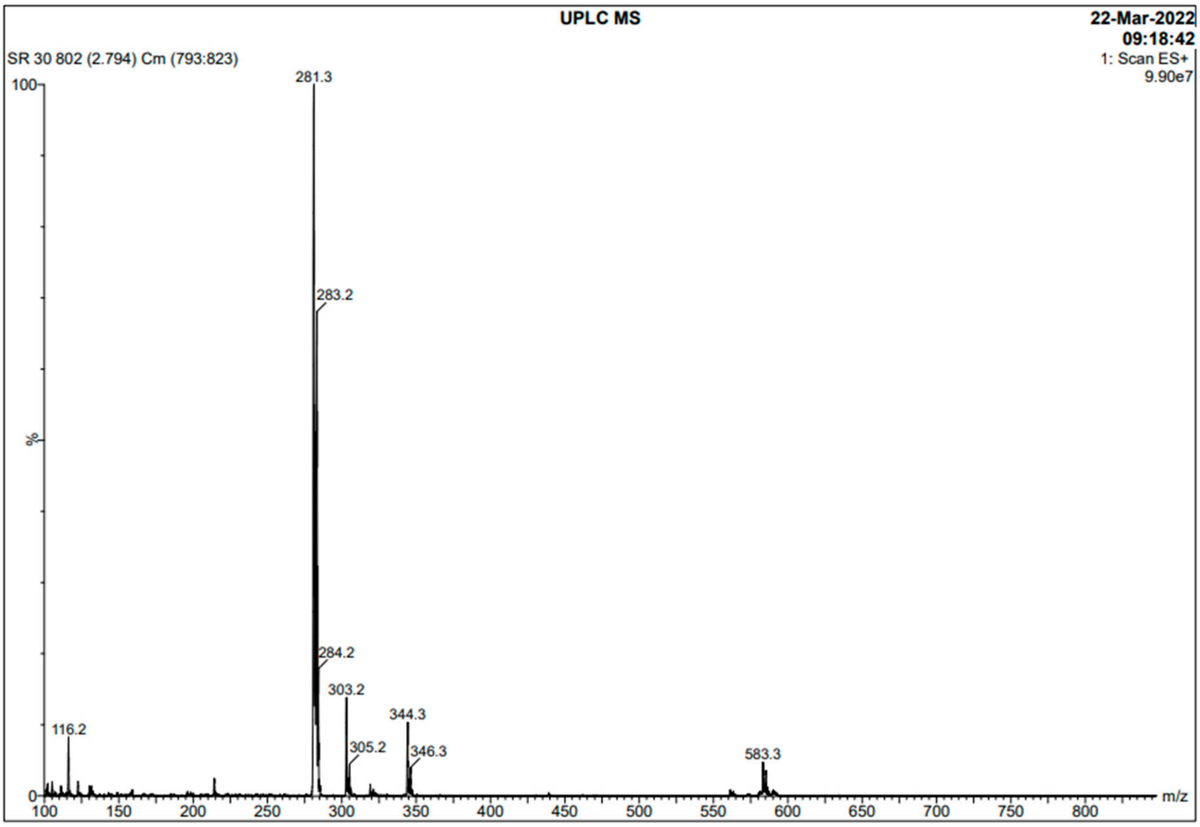
UPLC MS
22-Mar-2022
09:18:42
SR 30 802 (2.794) Cm (793:823)
1: Scan ES+
9.90e7
%
281.3
283.2
284.2
303.2
305.2
344.3
346.3
116.2
583.3
m/z

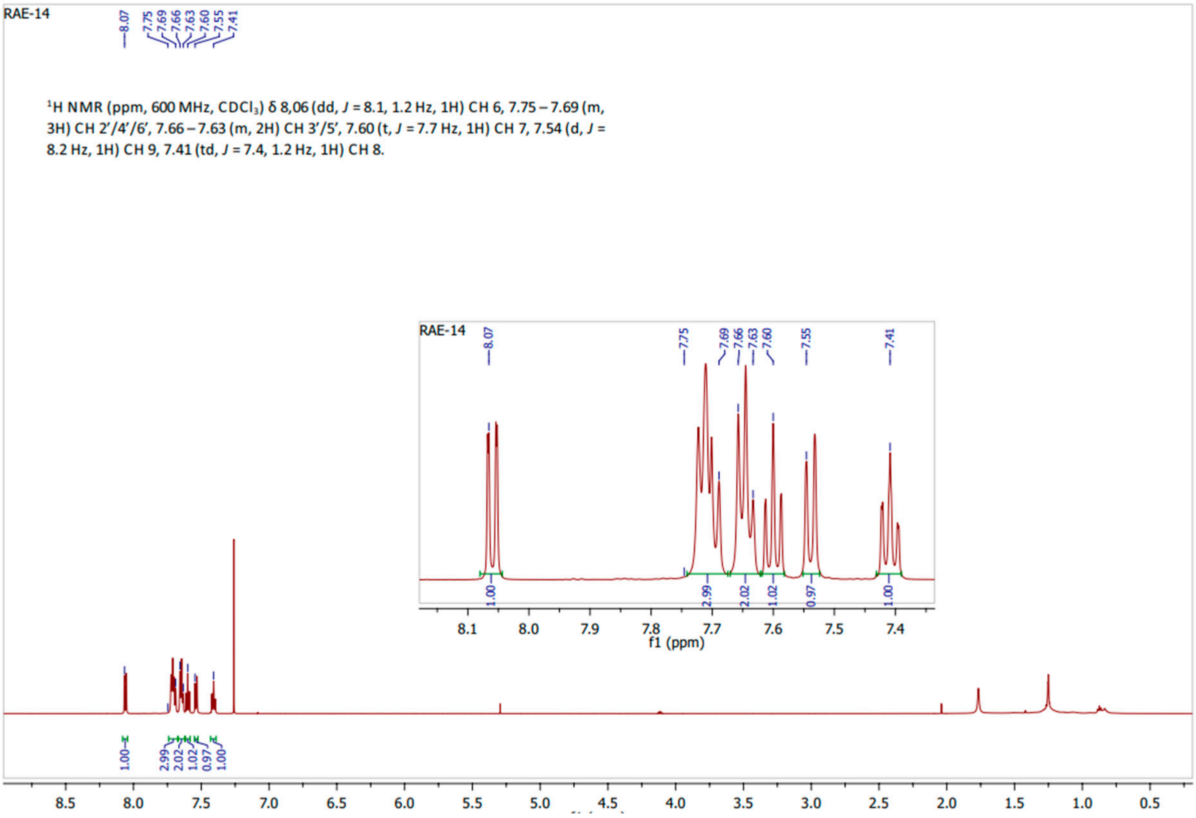
RAE-14
^{1}H NMR (ppm, 600 MHz, $CDCl_3$) δ 8,06 (dd, J = 8.1, 1.2 Hz, 1H) CH 6, 7.75 – 7.69 (m, 3H) CH 2′/4′/6′, 7.66 – 7.63 (m, 2H) CH 3′/5′, 7.60 (t, J = 7.7 Hz, 1H) CH 7, 7.54 (d, J = 8.2 Hz, 1H) CH 9, 7.41 (td, J = 7.4, 1.2 Hz, 1H) CH 8.
f1 (ppm)

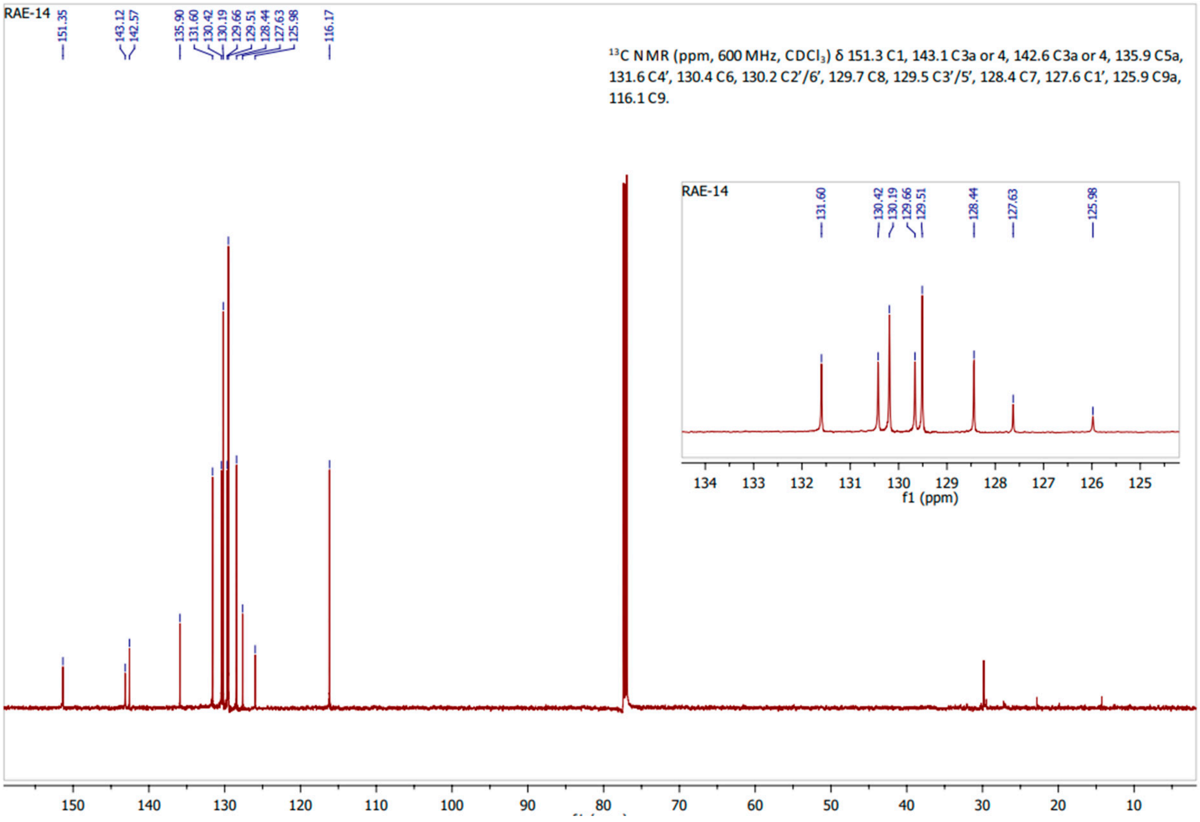

*S19. 4-chloro-1-(3,4,5-trimethoxyphenyl)-[1,2,4]triazolo[4,3-a]quinoxaline (**15d**).*

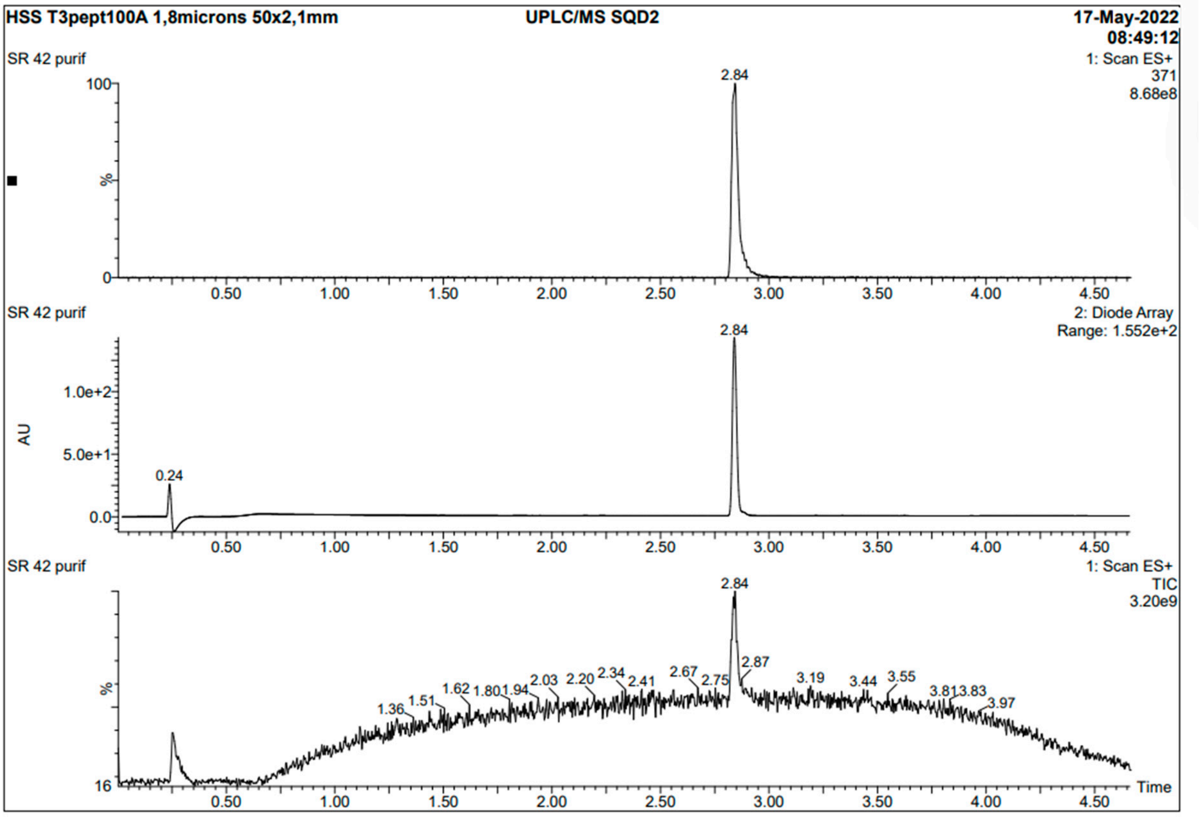

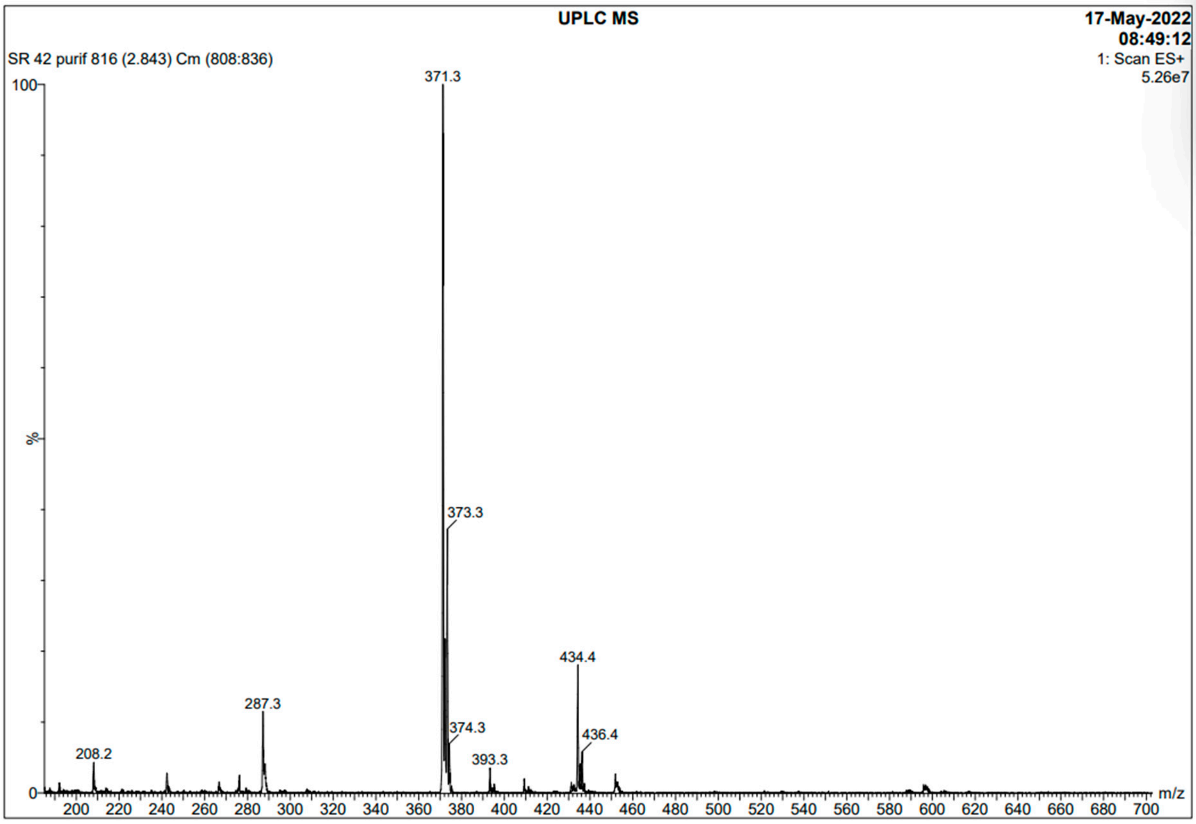
UPLC MS
17-May-2022
08:49:12
SR 42 purif 816 (2.843) Cm (808:836)
1: Scan ES+
5.26e7
100
%
0
371.3
373.3
434.4
287.3
374.3
436.4
208.2
393.3
m/z

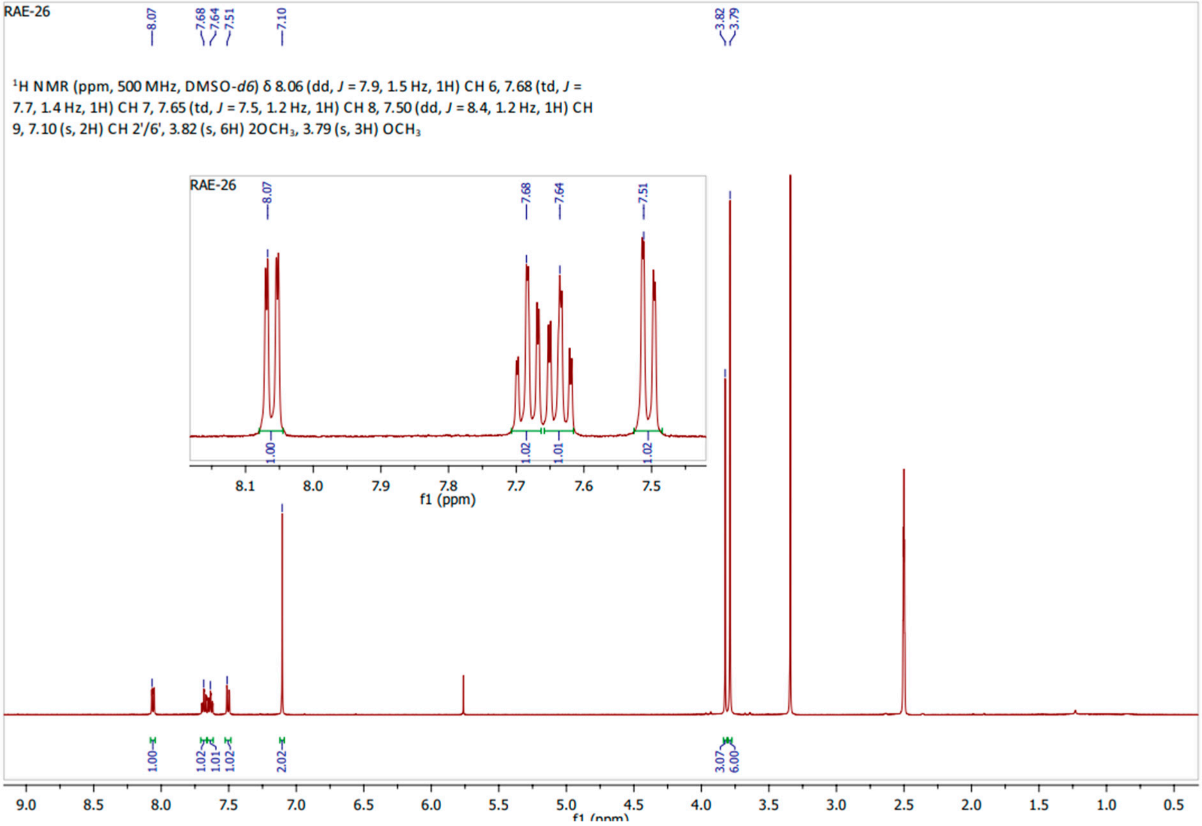
RAE-26
^{1}H NMR (ppm, 500 MHz, DMSO-*d6*) δ 8.06 (dd, *J* = 7.9, 1.5 Hz, 1H) CH 6, 7.68 (td, *J* = 7.7, 1.4 Hz, 1H) CH 7, 7.65 (td, *J* = 7.5, 1.2 Hz, 1H) CH 8, 7.50 (dd, *J* = 8.4, 1.2 Hz, 1H) CH 9, 7.10 (s, 2H) CH 2'/6', 3.82 (s, 6H) 2OCH₃, 3.79 (s, 3H) OCH₃
f1 (ppm)

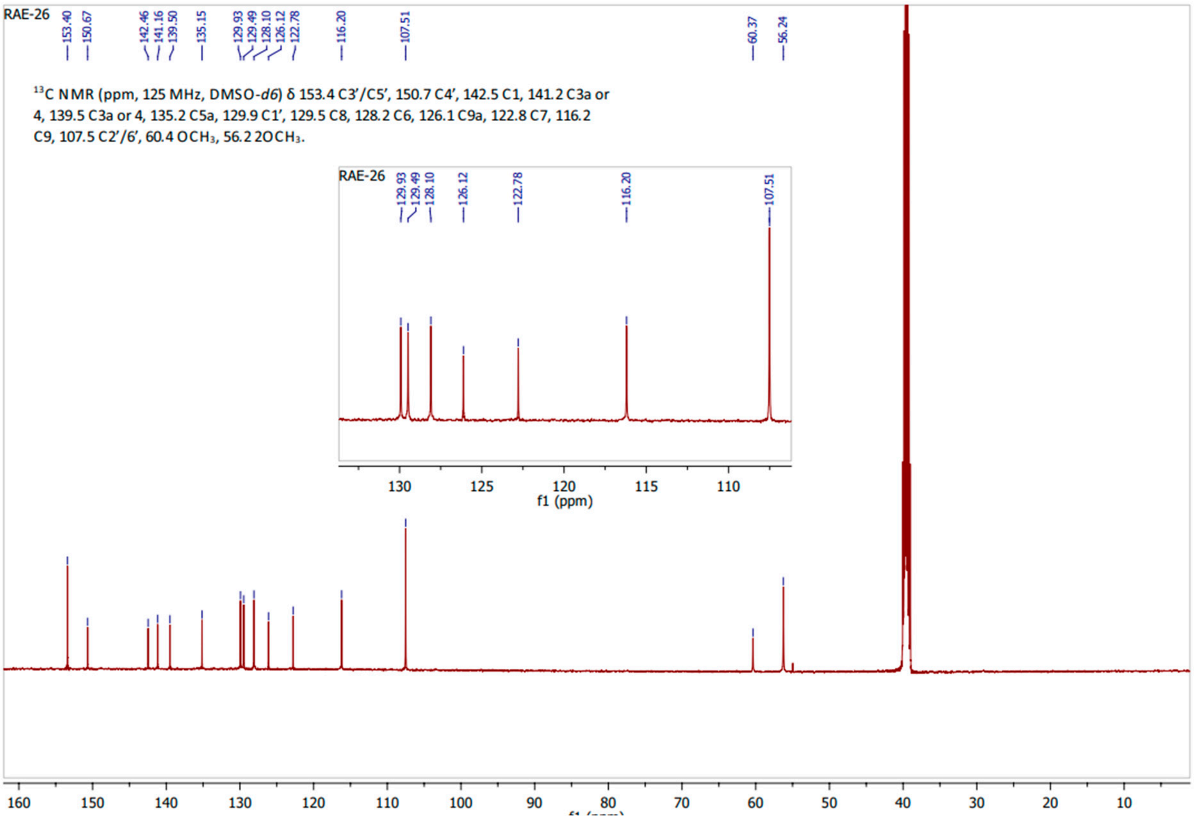

*S20. 4-chloro-1-(pyridin-3-yl)-[1,2,4]triazolo[4,3-a]quinoxaline (**15e**).*

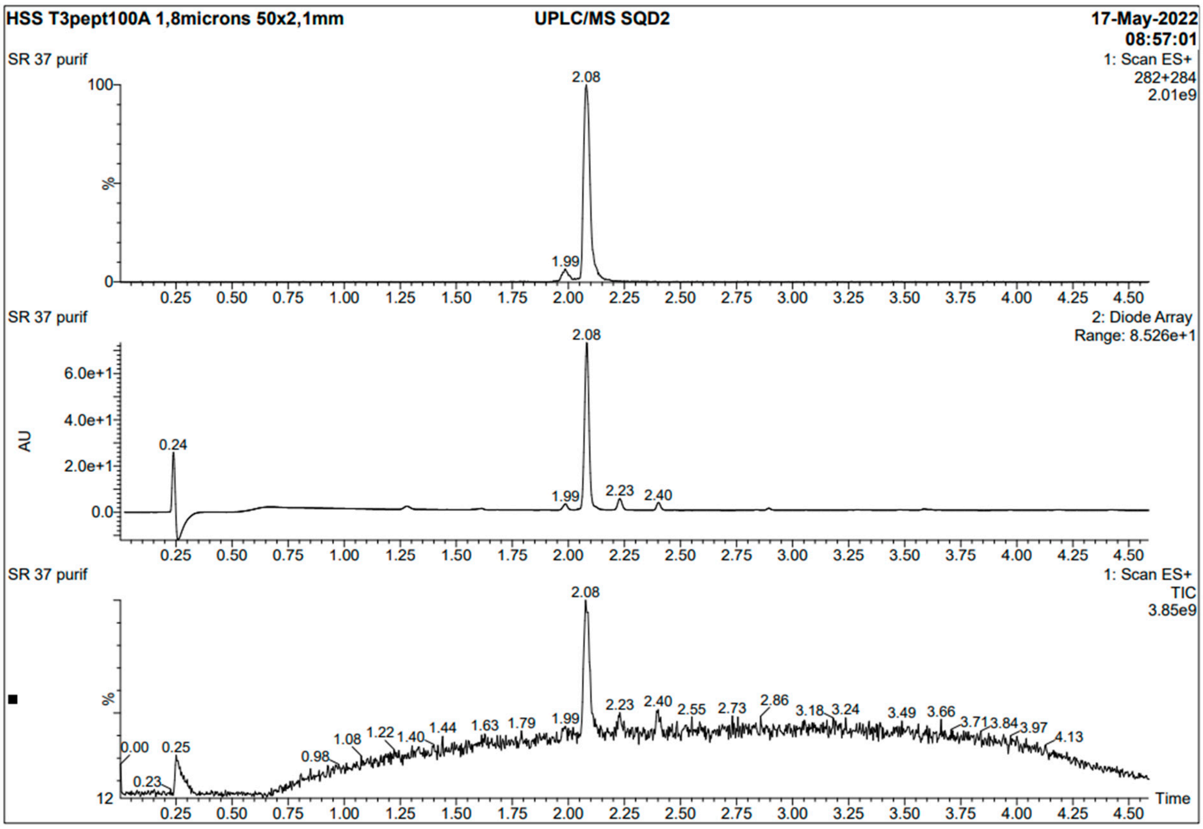

molecules

MDPI

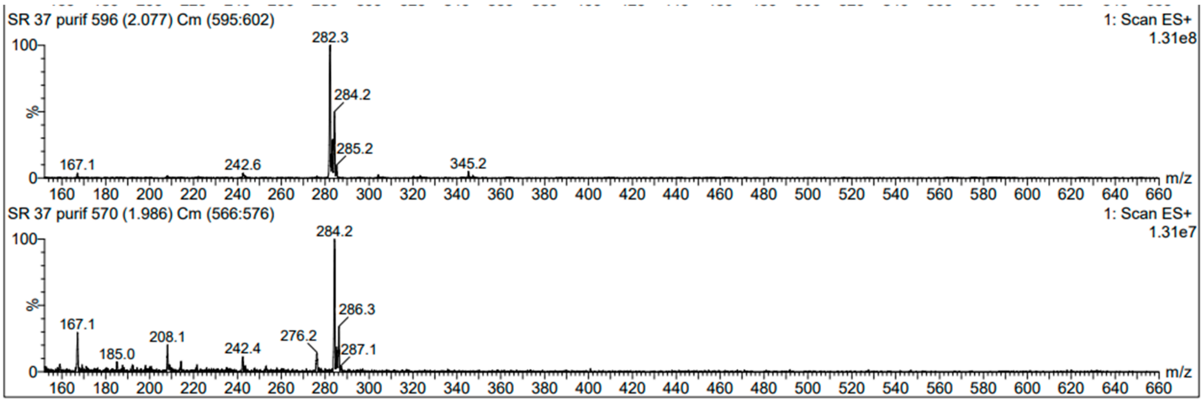

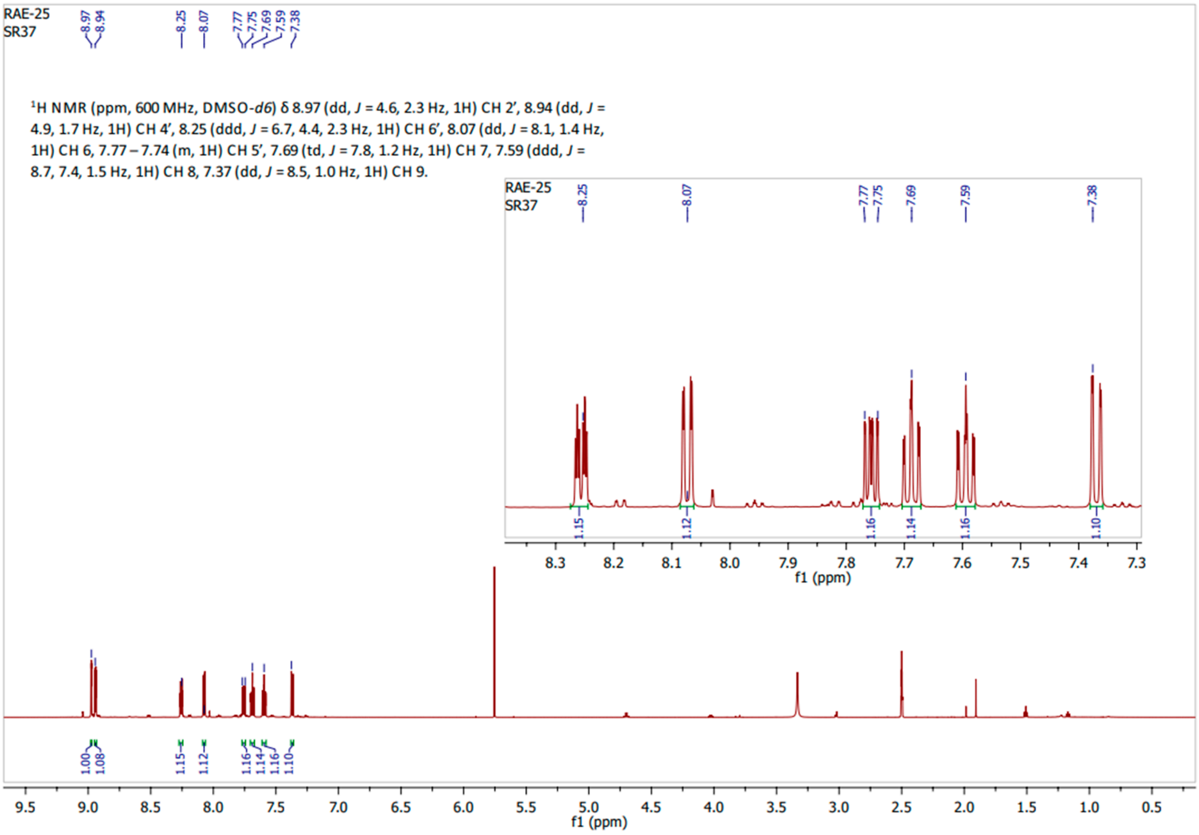

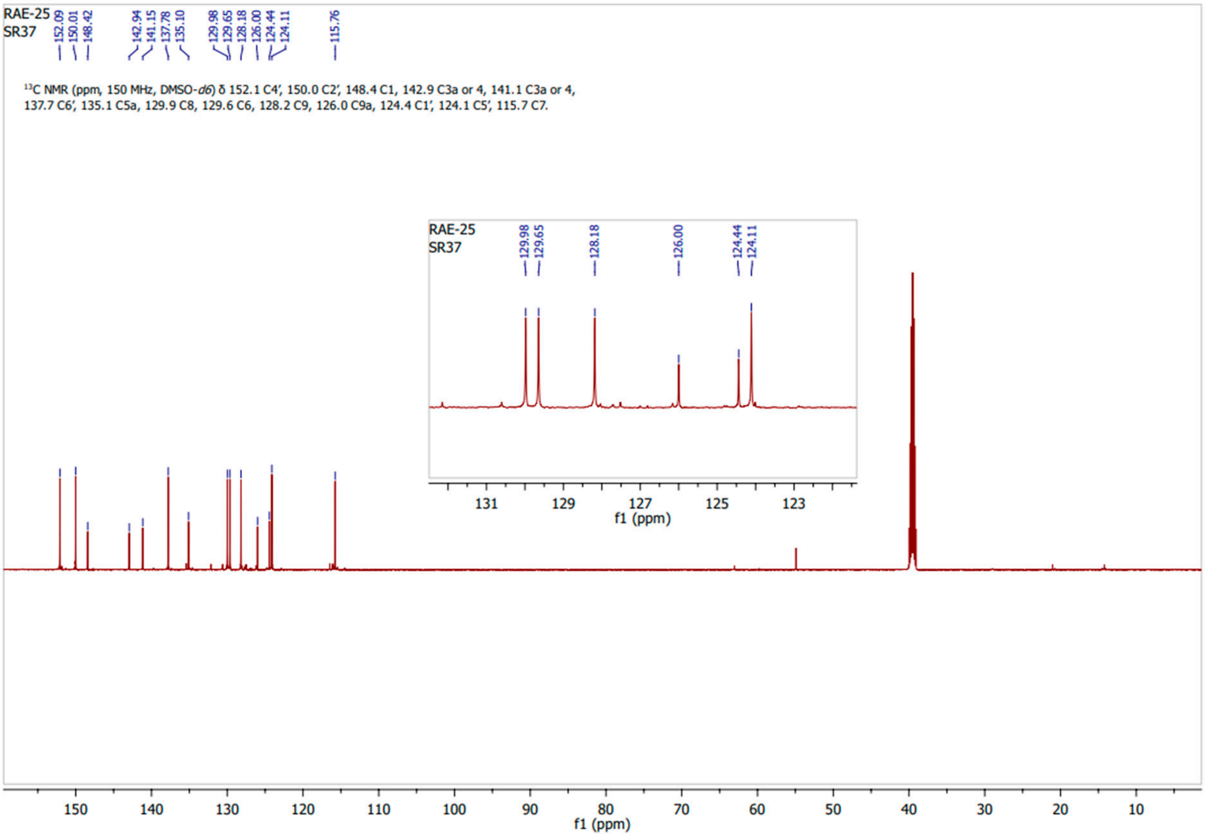

S21. 4-chloro-1-phenethyl-[1,2,4]triazolo[4,3-a]quinoxaline (**15f**).

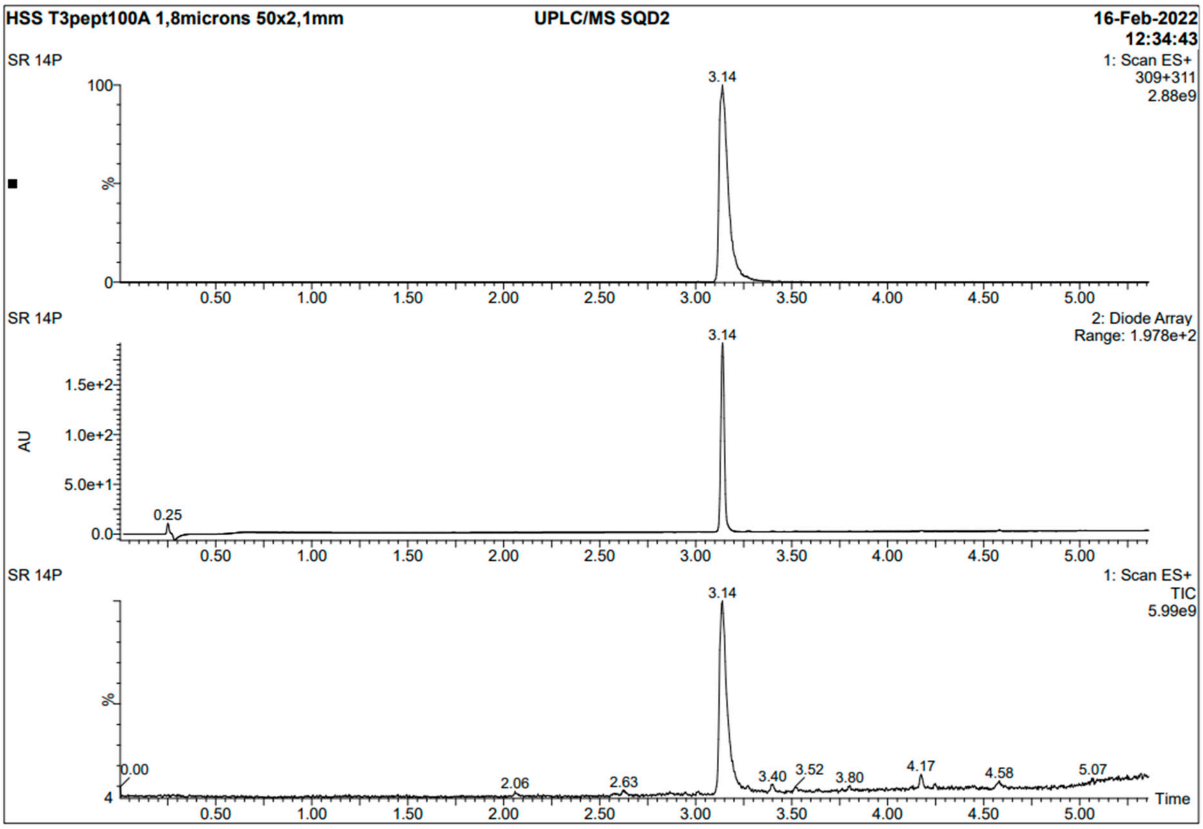

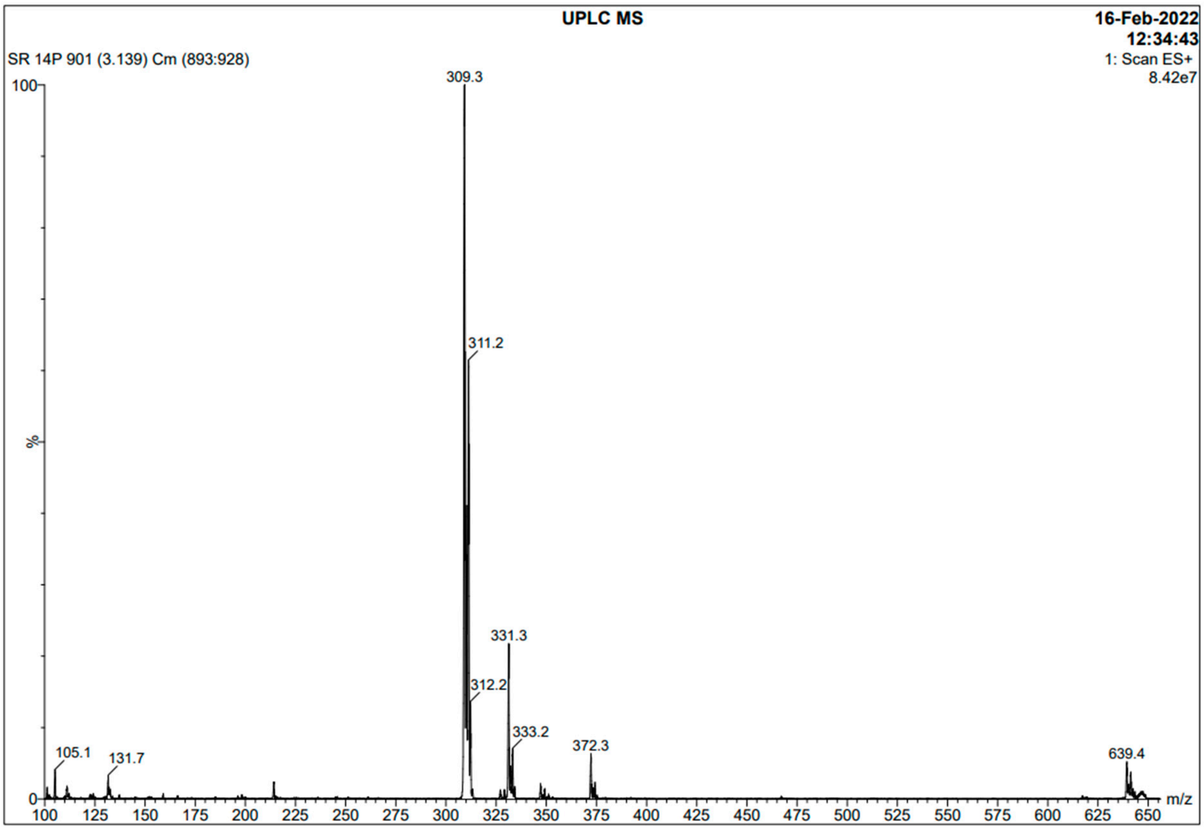
UPLC MS
16-Feb-2022
12:34:43
SR 14P 901 (3.139) Cm (893:928)
1: Scan ES+
8.42e7
309.3
311.2
331.3
312.2
333.2
372.3
105.1
131.7
639.4
%
m/z

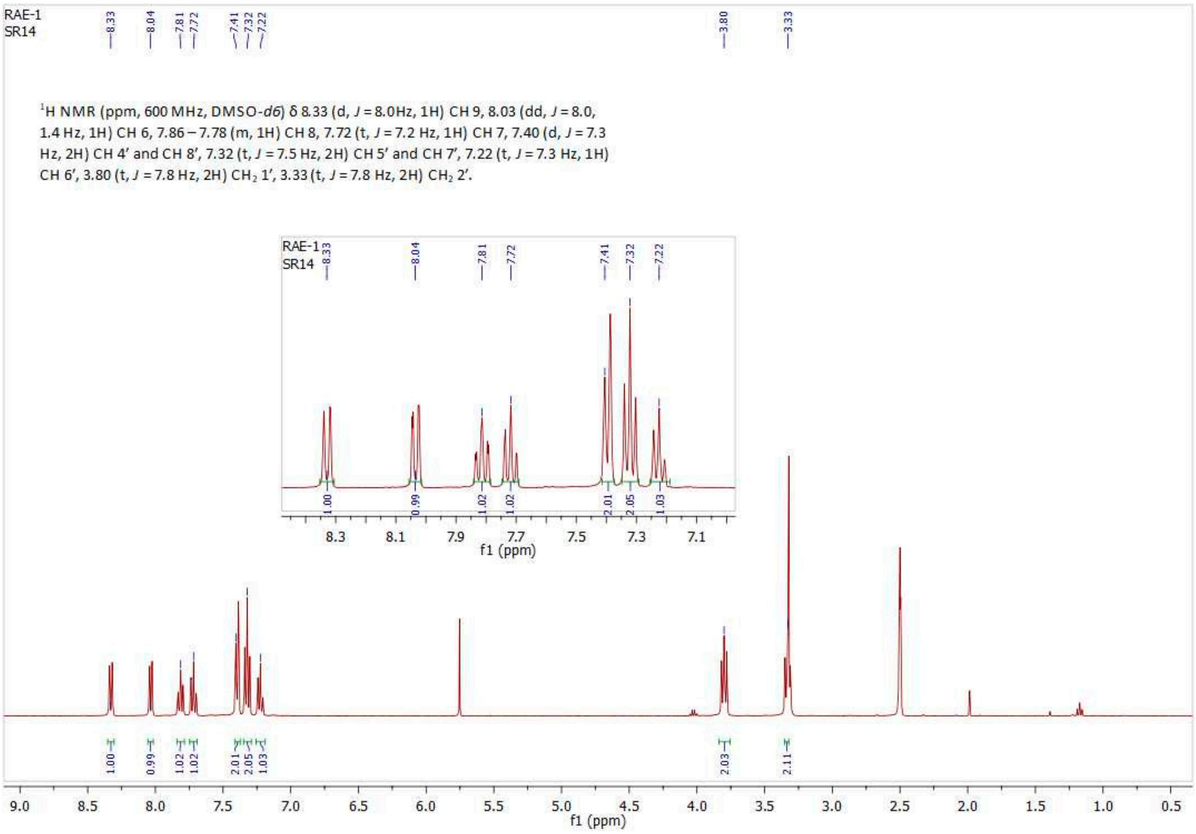
RAE-1
SR14
^{1}H NMR (ppm, 600 MHz, DMSO-d6) δ 8.33 (d, J = 8.0Hz, 1H) CH 9, 8.03 (dd, J = 8.0, 1.4 Hz, 1H) CH 6, 7.86 – 7.78 (m, 1H) CH 8, 7.72 (t, J = 7.2 Hz, 1H) CH 7, 7.40 (d, J = 7.3 Hz, 2H) CH 4′ and CH 8′, 7.32 (t, J = 7.5 Hz, 2H) CH 5′ and CH 7′, 7.22 (t, J = 7.3 Hz, 1H) CH 6′, 3.80 (t, J = 7.8 Hz, 2H) CH₂ 1′, 3.33 (t, J = 7.8 Hz, 2H) CH₂ 2′.
f1 (ppm)

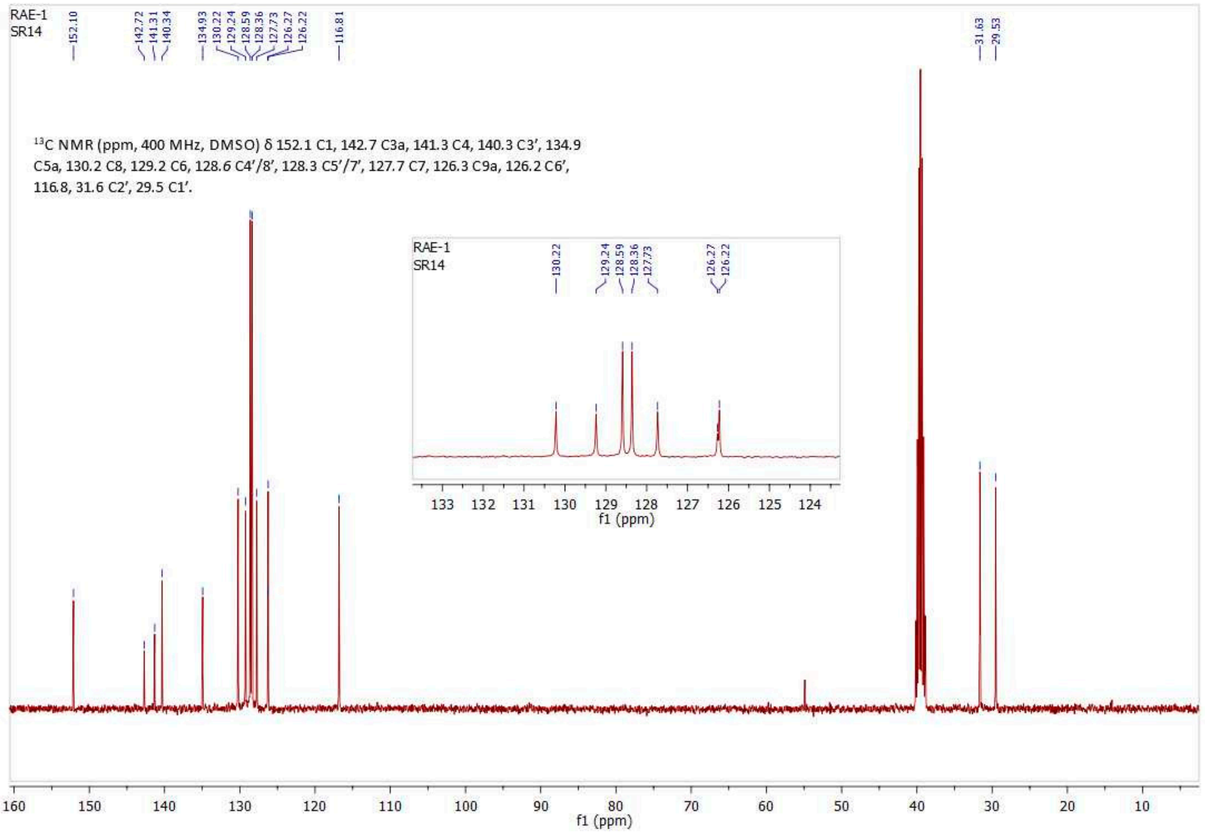

*S22. 1-butyl-4-chloro-[1,2,4]triazolo[4,3-a]quinoxaline (**15g**).*

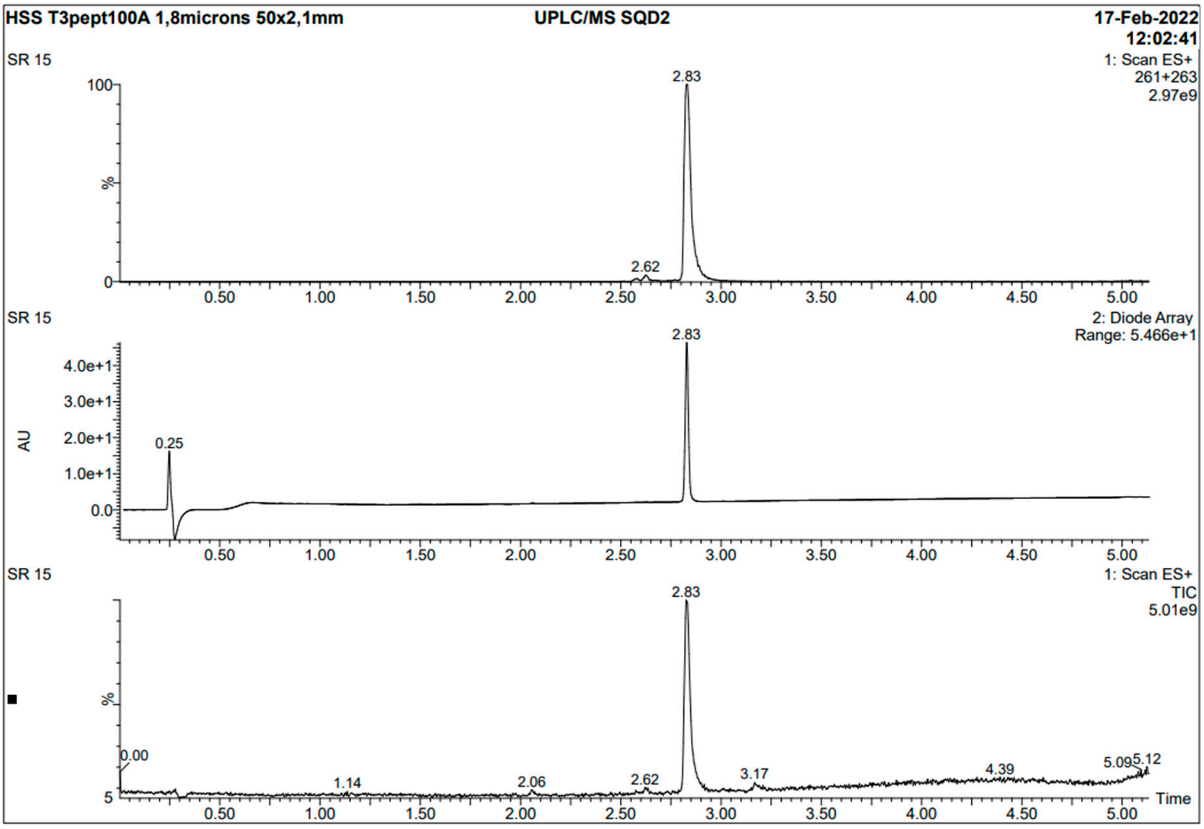

molecules

MDPI

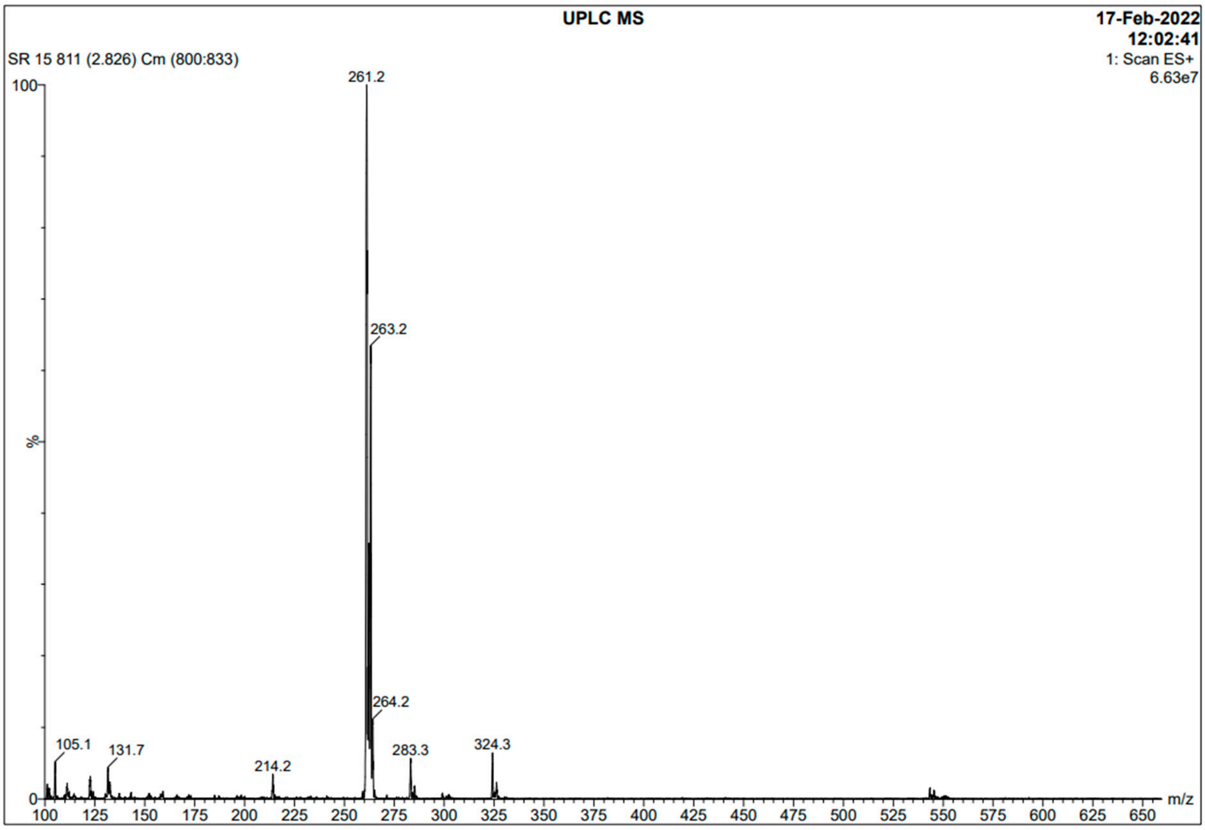

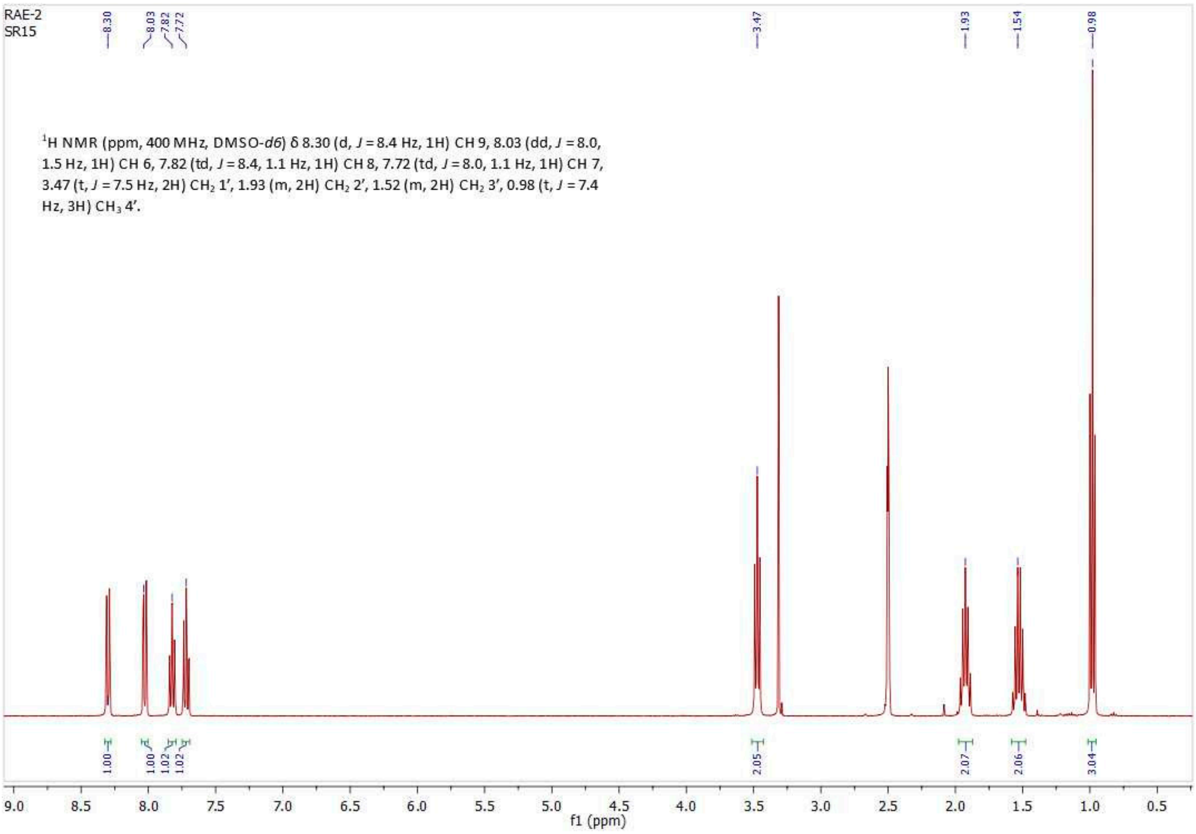

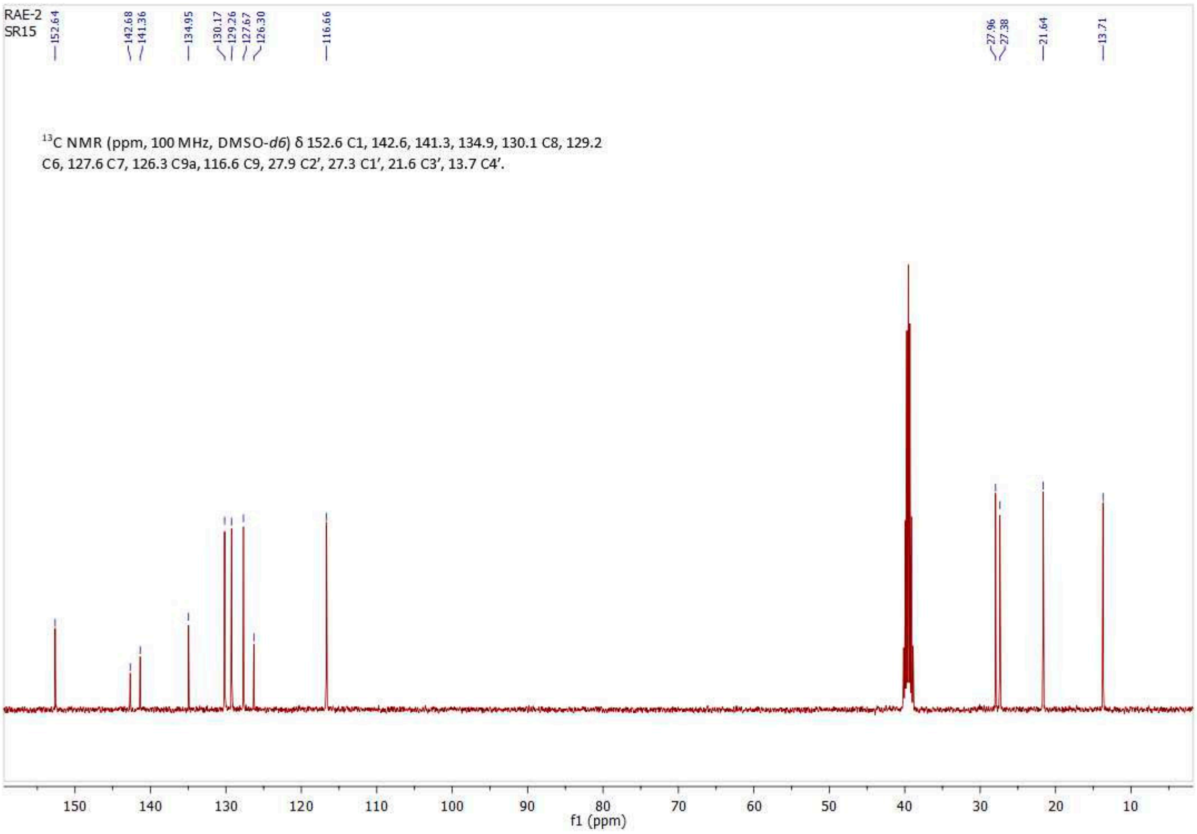

S23. *4-chloro-1-isobutyl-[1,2,4]triazolo[4,3-a]quinoxaline* (***15h***).

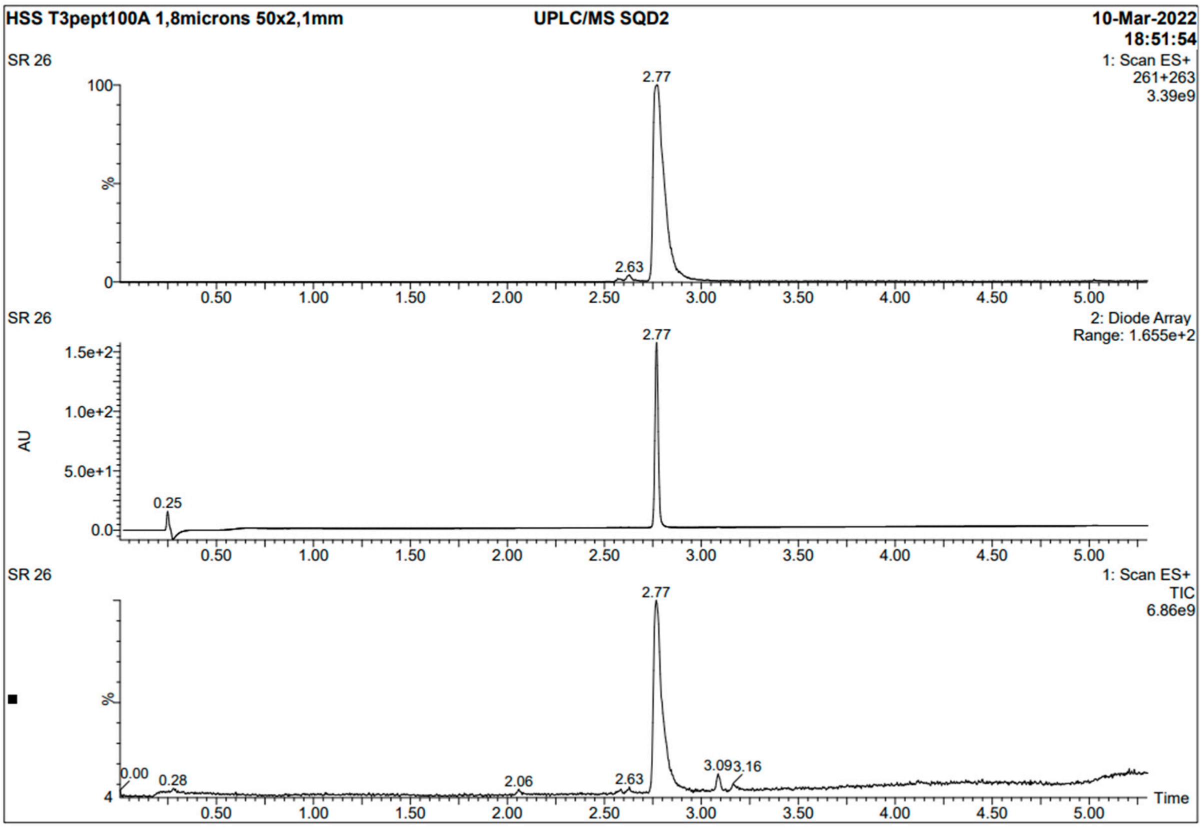

molecules

MDPI

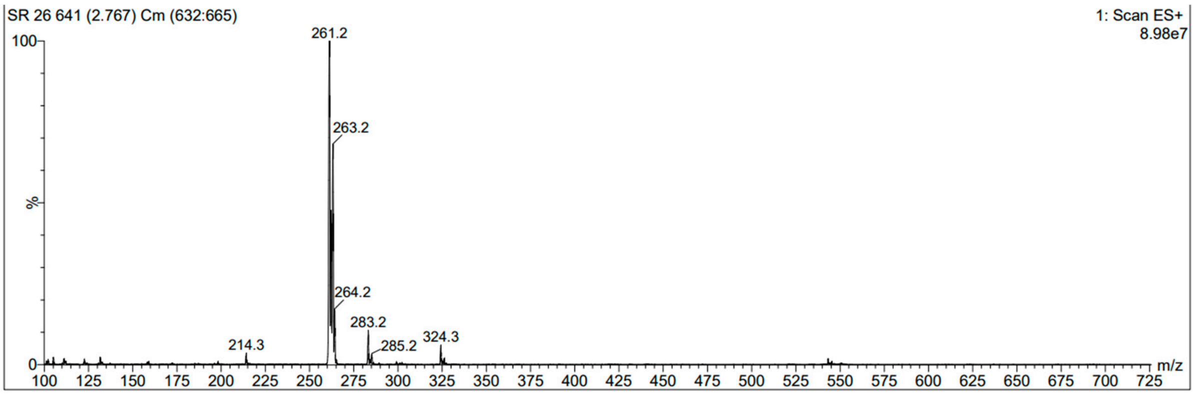

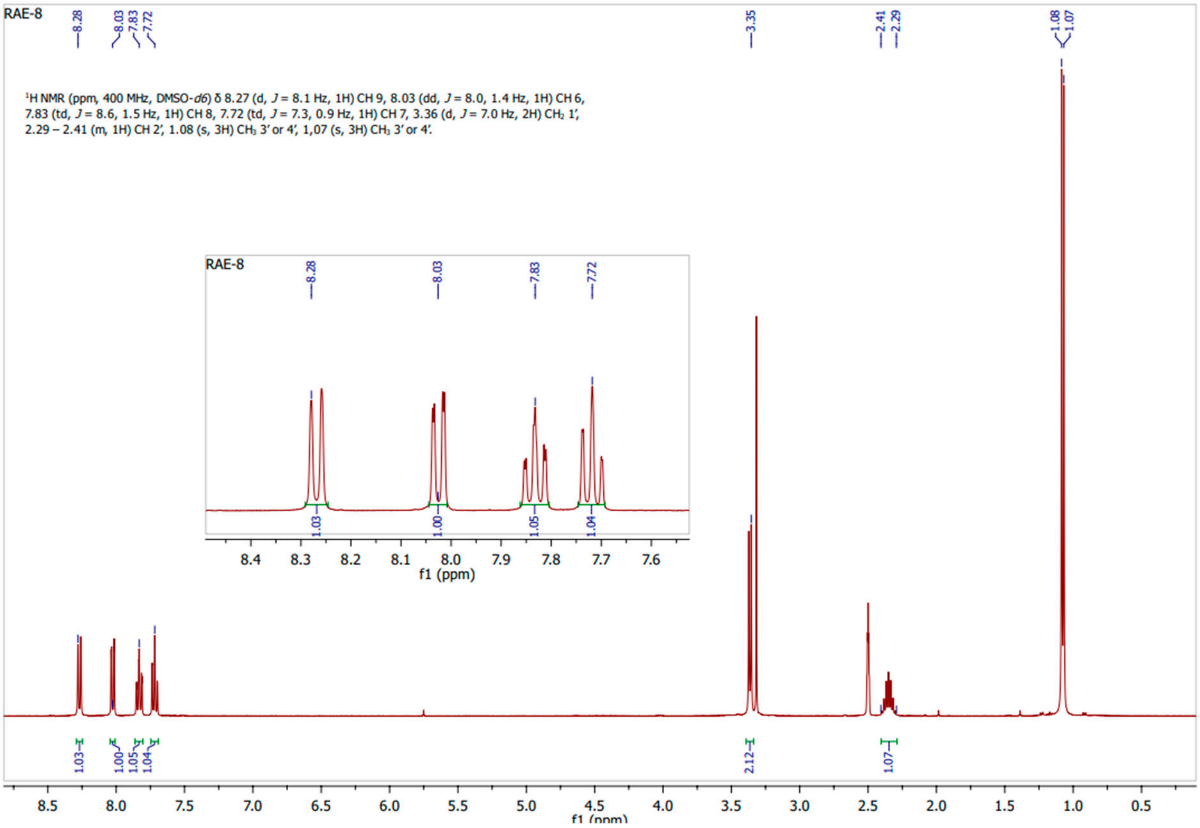

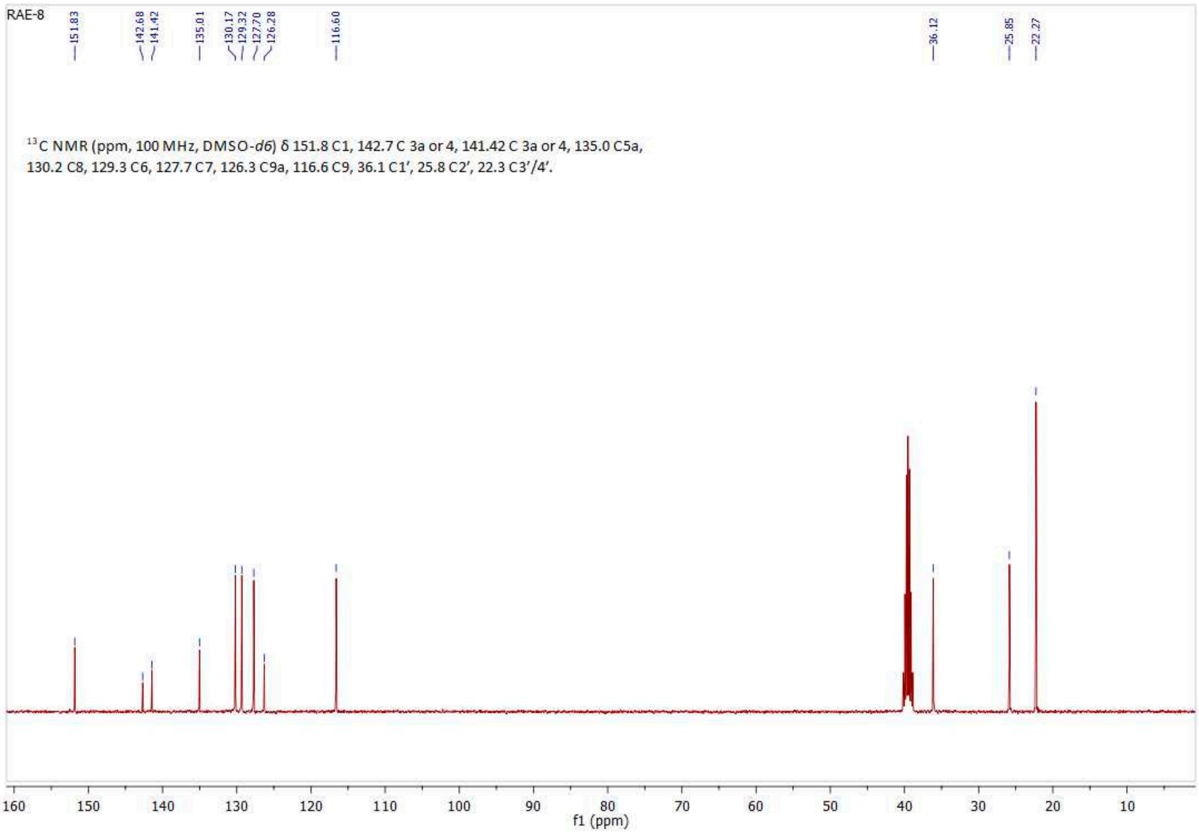

S24. 4-chloro-1-methyl-[1,2,4]triazolo[4,3-a]quinoxaline (**15i**).

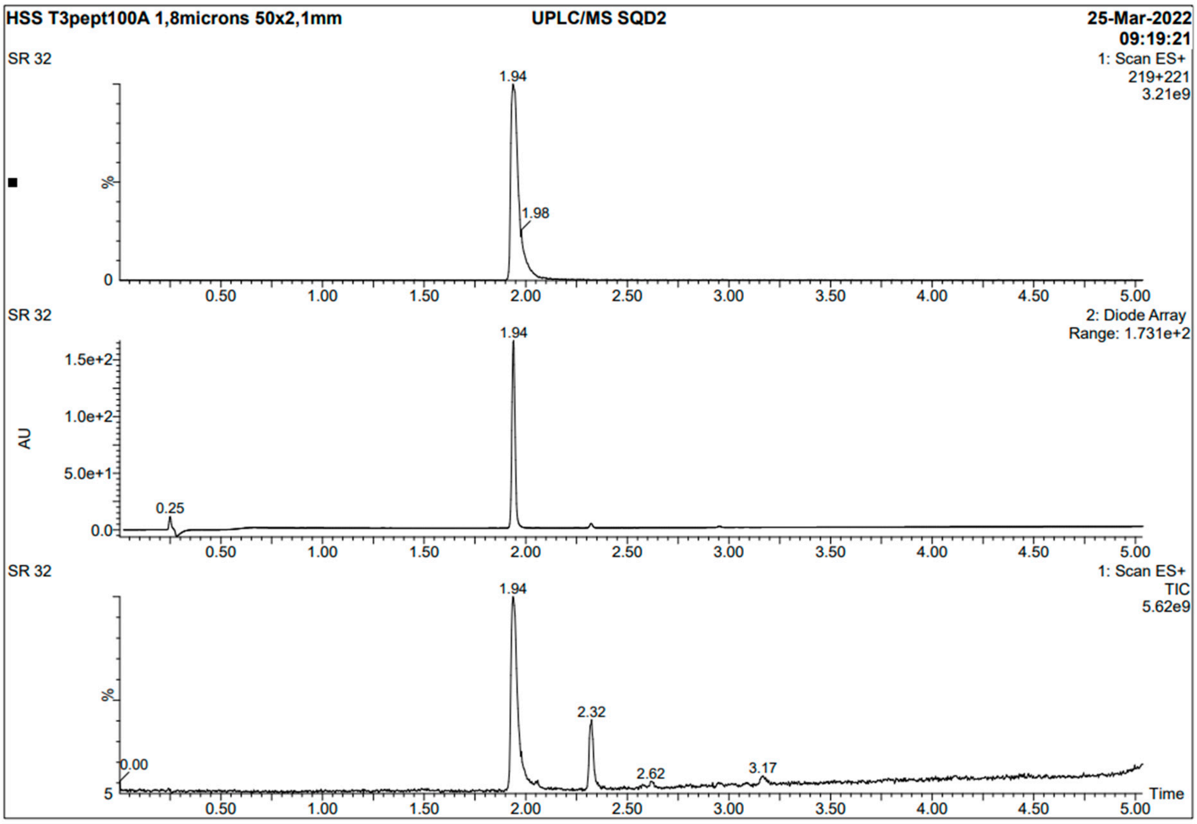

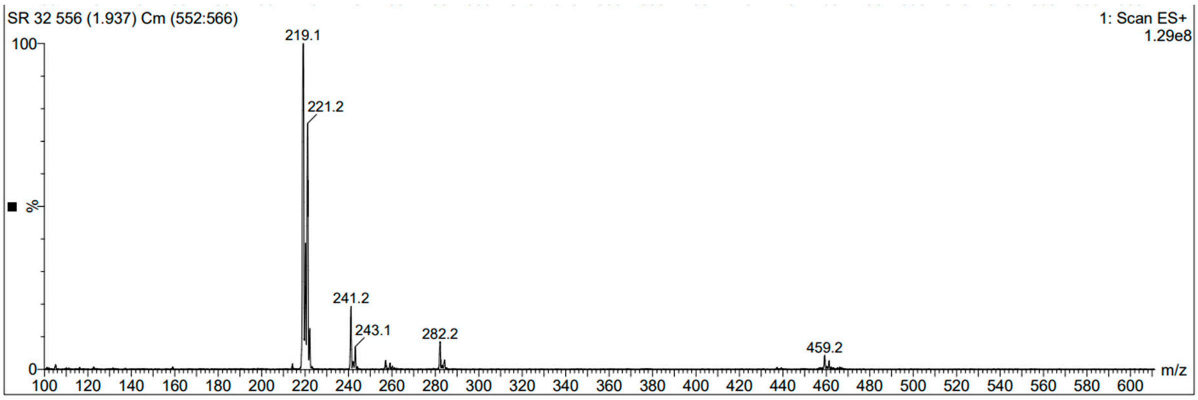

SR 32 556 (1.937) Cm (552:566)
1: Scan ES+
1.29e8
219.1
221.2
241.2
243.1
282.2
459.2
m/z
%

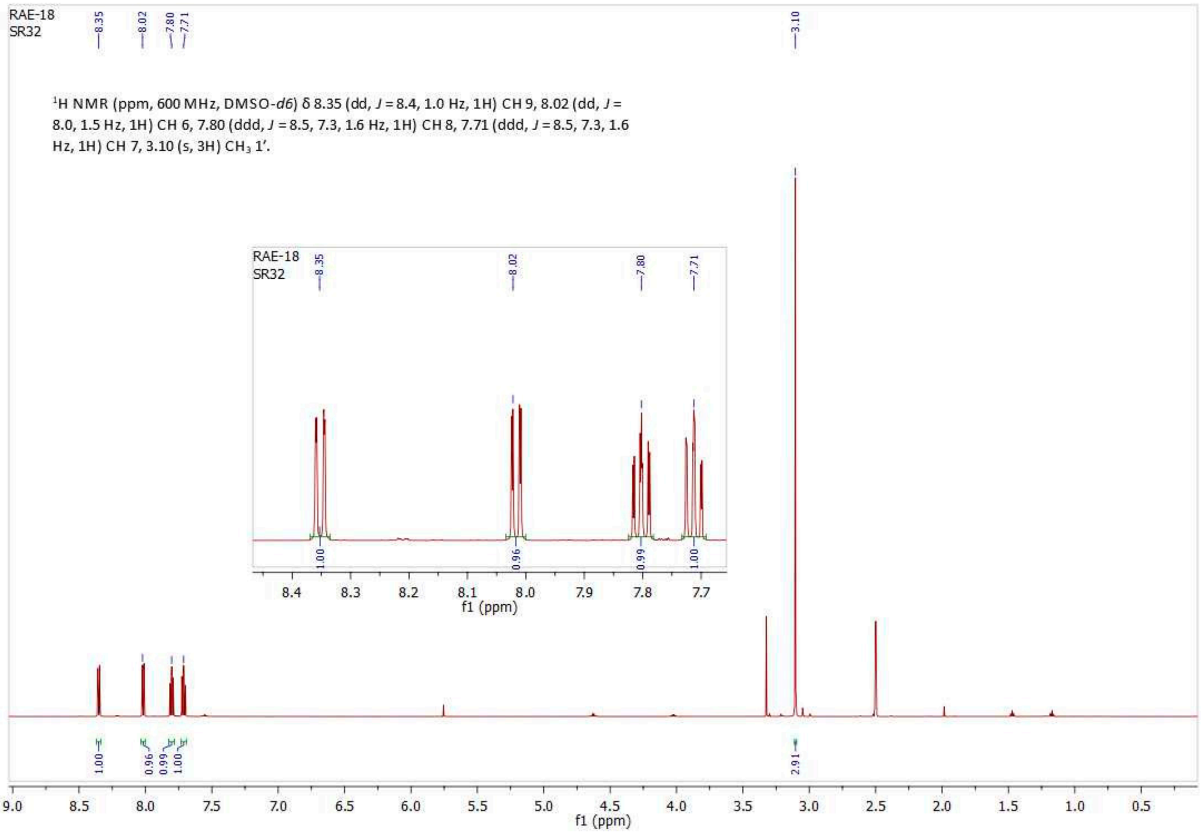

RAE-18
SR32
^{1}H NMR (ppm, 600 MHz, DMSO-d6) δ 8.35 (dd, J = 8.4, 1.0 Hz, 1H) CH 9, 8.02 (dd, J = 8.0, 1.5 Hz, 1H) CH 6, 7.80 (ddd, J = 8.5, 7.3, 1.6 Hz, 1H) CH 8, 7.71 (ddd, J = 8.5, 7.3, 1.6 Hz, 1H) CH 7, 3.10 (s, 3H) CH3 1'.
f1 (ppm)

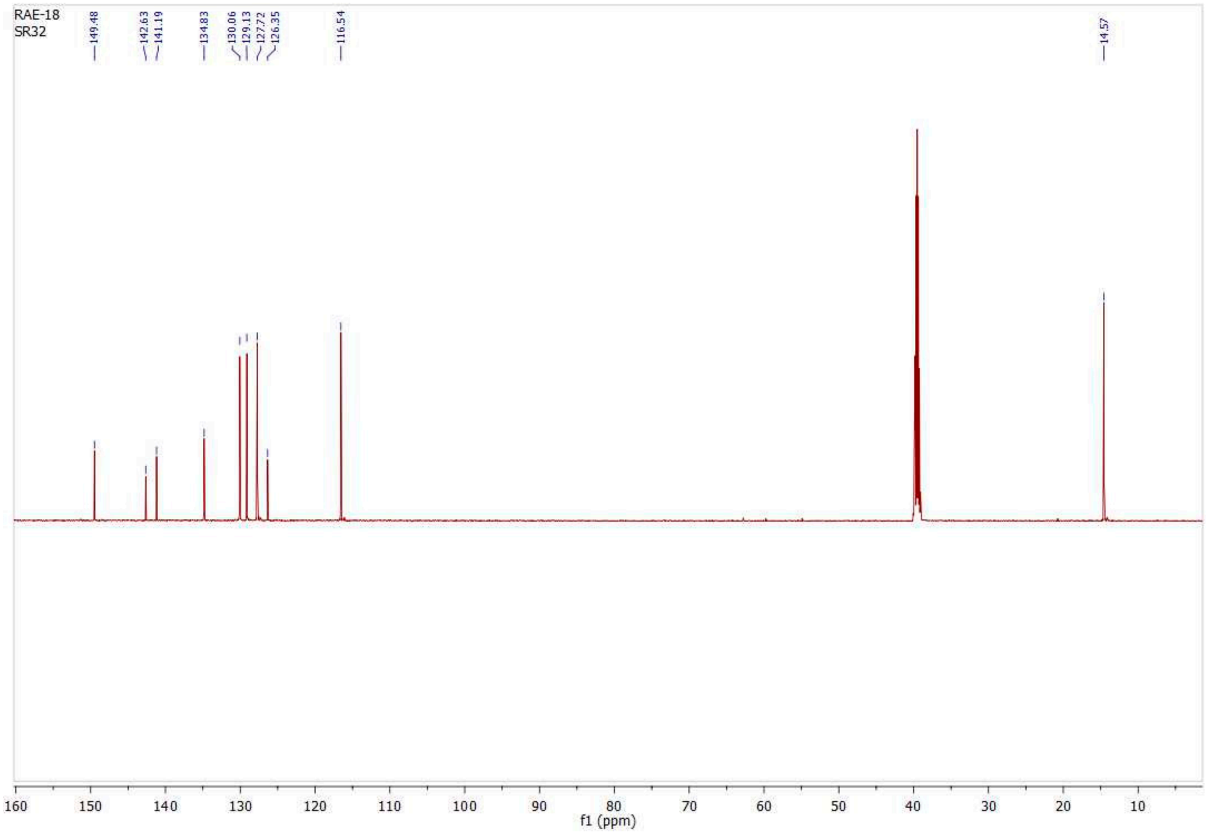

*S25. 1-(3,4-dimethoxyphenyl)-N-methyl-[1,2,4]triazolo[4,3-a]quinoxalin-4-amine (**16a**).*

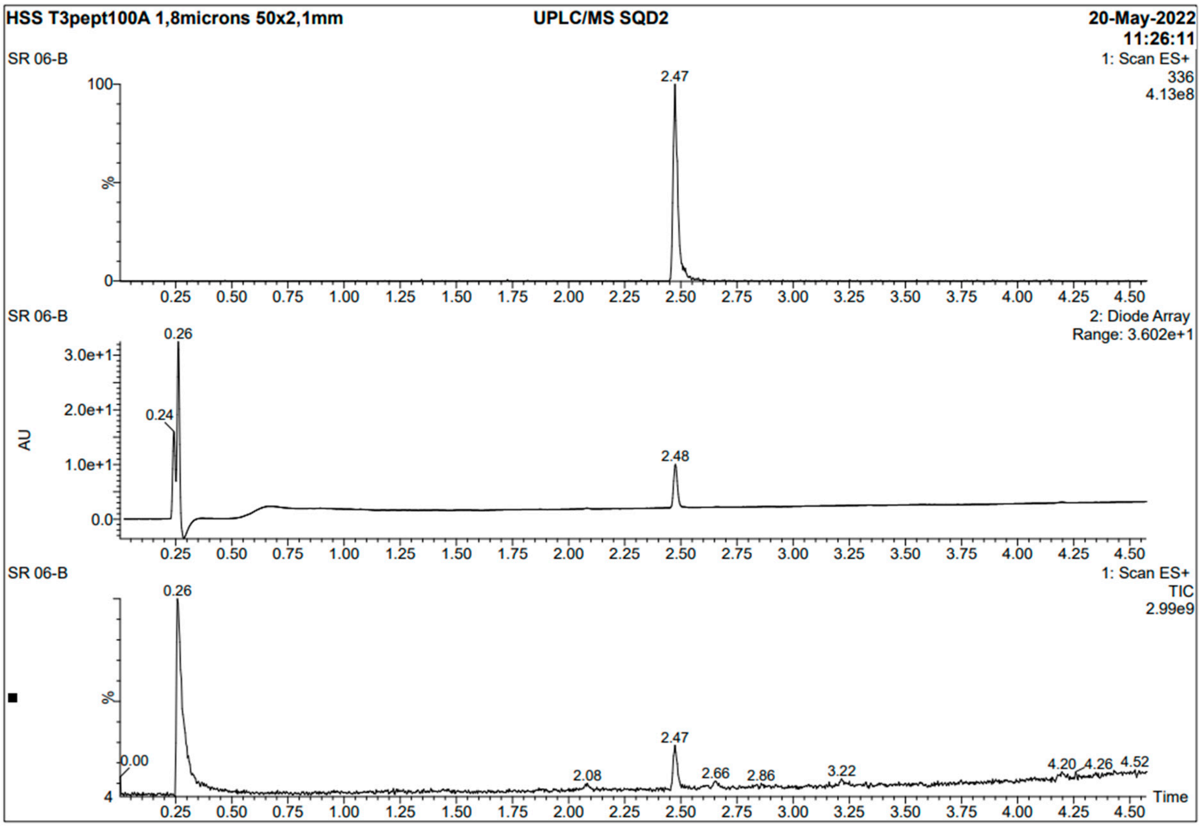

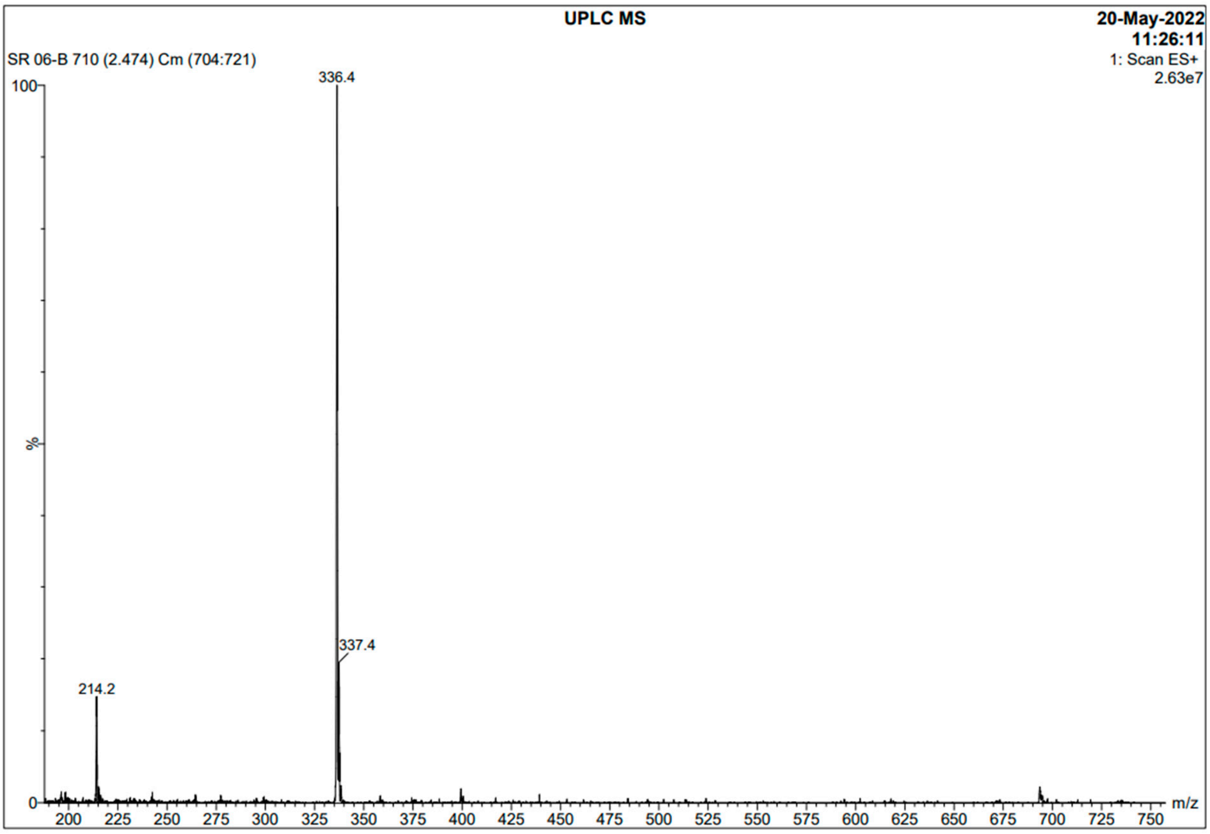
UPLC MS
20-May-2022
11:26:11
SR 06-B 710 (2.474) Cm (704:721)
1: Scan ES+
2.63e7
100
%
0
336.4
337.4
214.2
m/z
200 225 250 275 300 325 350 375 400 425 450 475 500 525 550 575 600 625 650 675 700 725 750

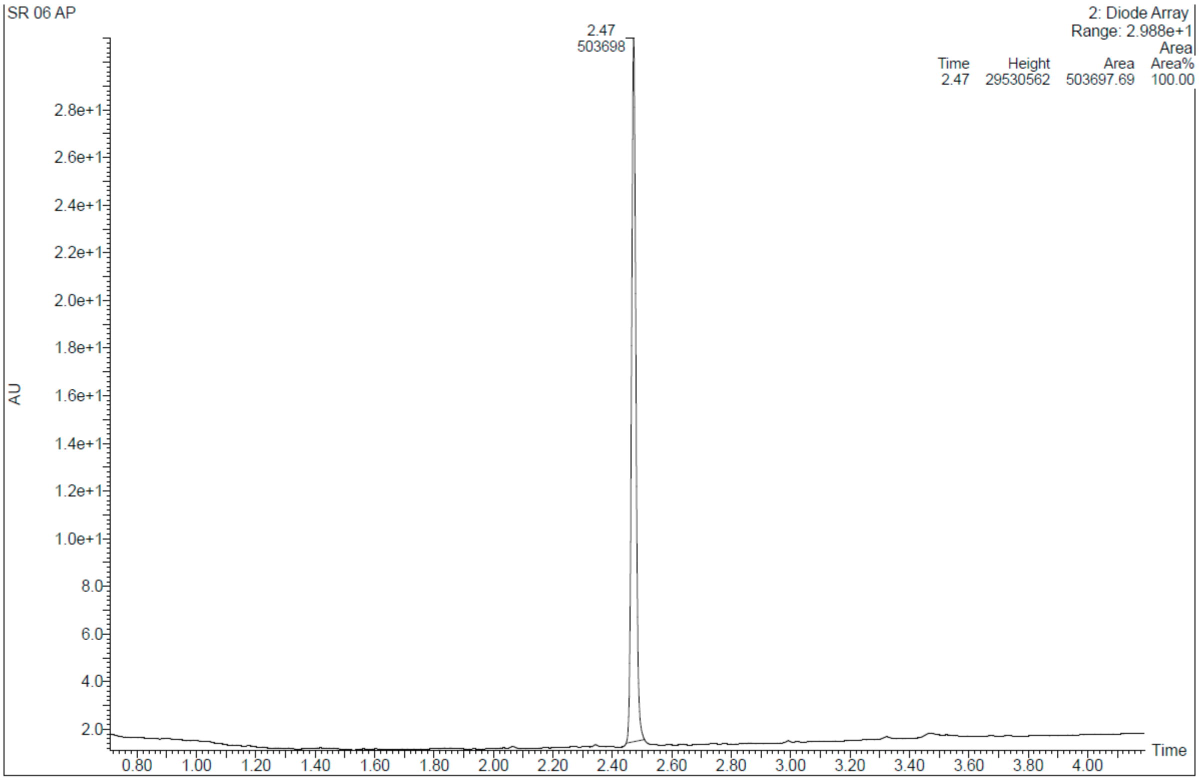
SR 06 AP
2: Diode Array
Range: 2.988e+1
Area
Time Height Area Area%
2.47 29530562 503697.69 100.00
2.47
503698
AU
2.8e+1 2.6e+1 2.4e+1 2.2e+1 2.0e+1 1.8e+1 1.6e+1 1.4e+1 1.2e+1 1.0e+1 8.0 6.0 4.0 2.0
0.80 1.00 1.20 1.40 1.60 1.80 2.00 2.20 2.40 2.60 2.80 3.00 3.20 3.40 3.60 3.80 4.00
Time

Acquisition Parameter

Source Type ESI Ion Polarity Positive Scan Begin 50 m/z Scan End 2200 m/z

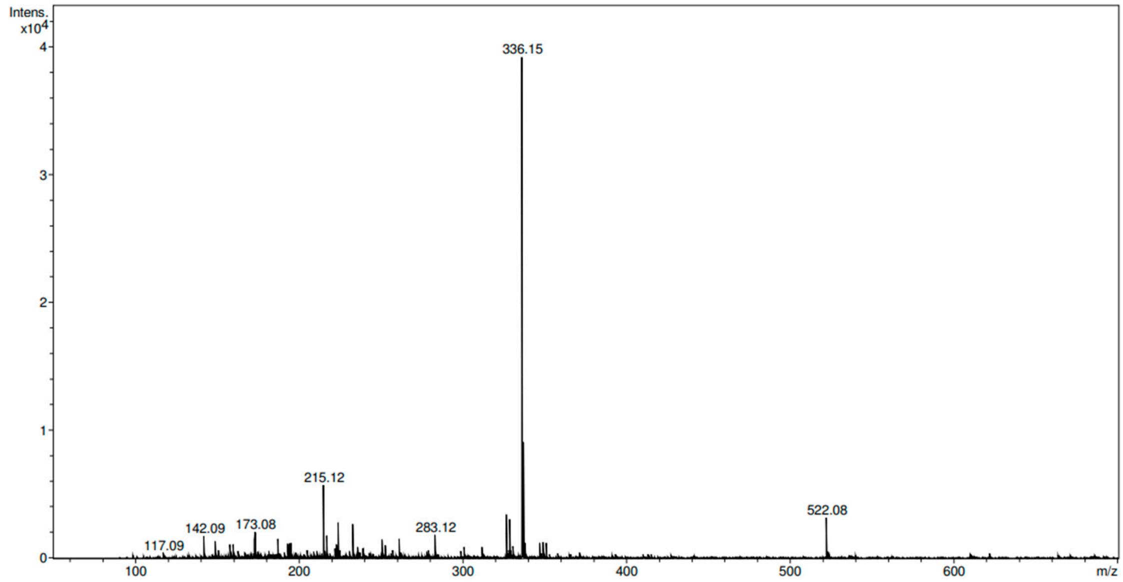

Acquisition Parameter

Source Type ESI Ion Polarity Positive Scan Begin 50 m/z Scan End 2200 m/z

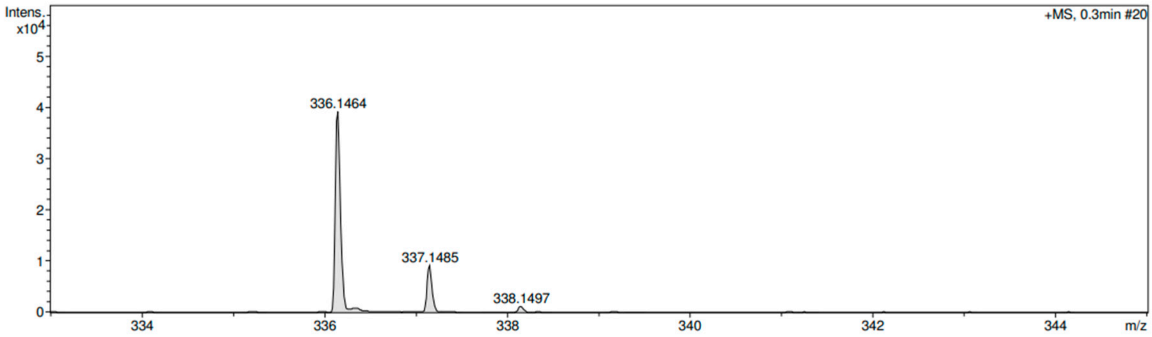

Meas. m/z	#	Ion Formula	m/z	err [ppm]	mSigma	# Sigma	Score	rdb	e⁻ Conf	N-Rule
336.1464	1	C18H18N5O2	336.1455	-2.6	9.8	1	100.00	12.5	even	ok

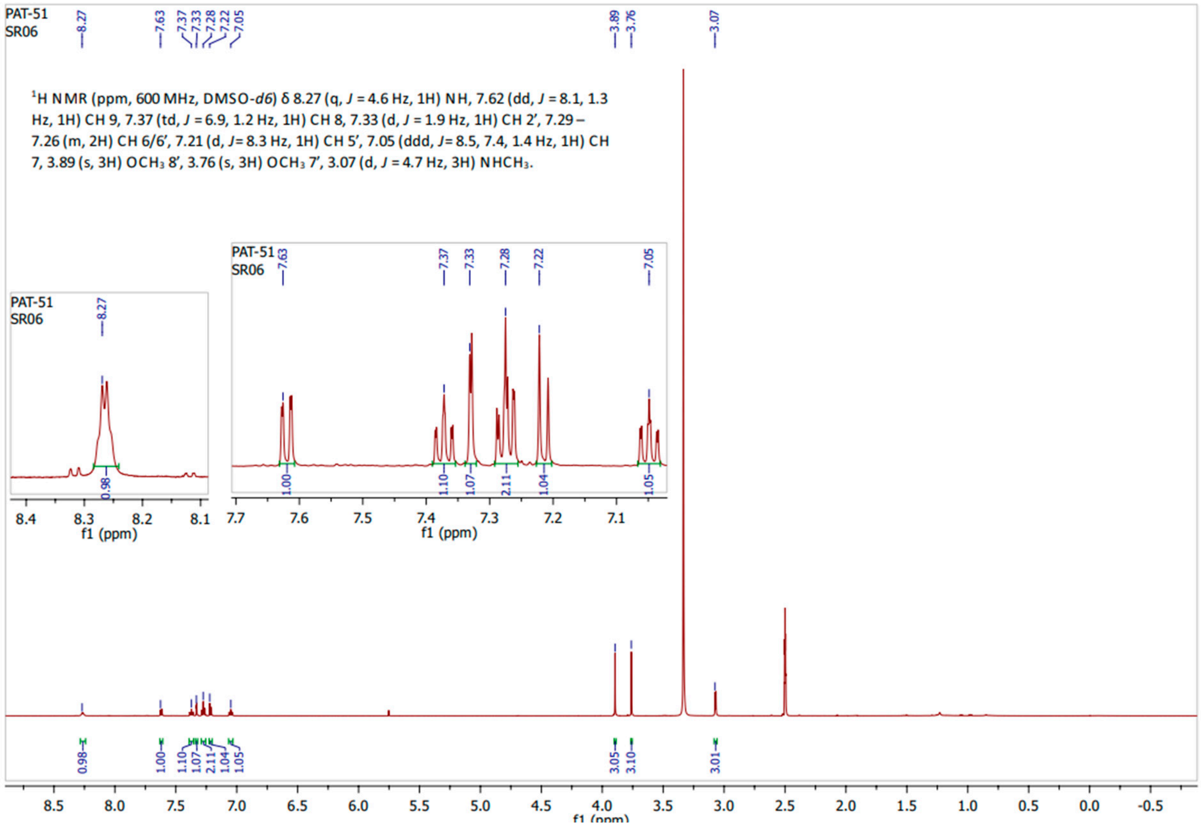
PAT-51
SR06
^{1}H NMR (ppm, 600 MHz, DMSO-d6) δ 8.27 (q, J = 4.6 Hz, 1H) NH, 7.62 (dd, J = 8.1, 1.3 Hz, 1H) CH 9, 7.37 (td, J = 6.9, 1.2 Hz, 1H) CH 8, 7.33 (d, J = 1.9 Hz, 1H) CH 2', 7.29 – 7.26 (m, 2H) CH 6/6', 7.21 (d, J= 8.3 Hz, 1H) CH 5', 7.05 (ddd, J= 8.5, 7.4, 1.4 Hz, 1H) CH 7, 3.89 (s, 3H) OCH3 8', 3.76 (s, 3H) OCH3 7', 3.07 (d, J = 4.7 Hz, 3H) NHCH3.
f1 (ppm)

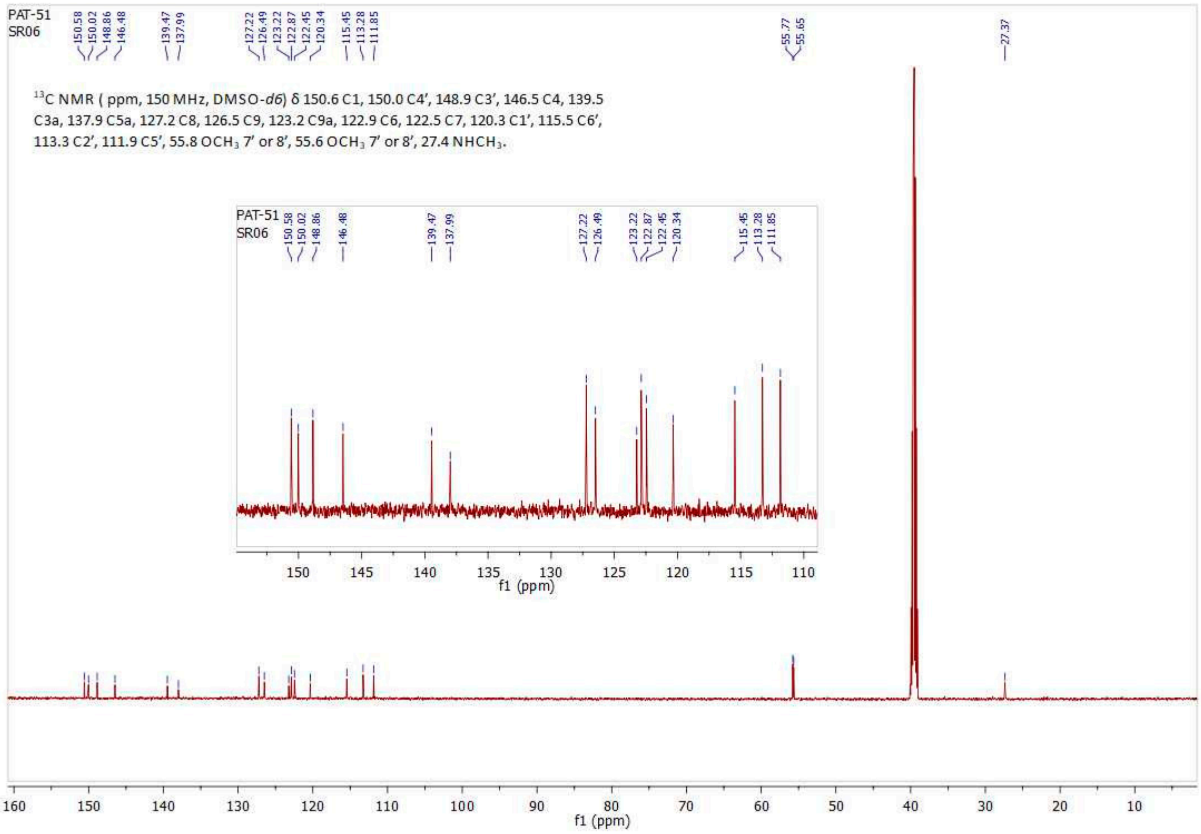
PAT-51
SR06
^{13}C NMR (ppm, 150 MHz, DMSO-d6) δ 150.6 C1, 150.0 C4', 148.9 C3', 146.5 C4, 139.5 C3a, 137.9 C5a, 127.2 C8, 126.5 C9, 123.2 C9a, 122.9 C6, 122.5 C7, 120.3 C1', 115.5 C6', 113.3 C2', 111.9 C5', 55.8 OCH3 7' or 8', 55.6 OCH3 7' or 8', 27.4 NHCH3.
f1 (ppm)

*S26. 1-(3-methoxyphenyl)-N-methyl-[1,2,4]triazolo[4,3-a]quinoxalin-4-amine (**16b**).*

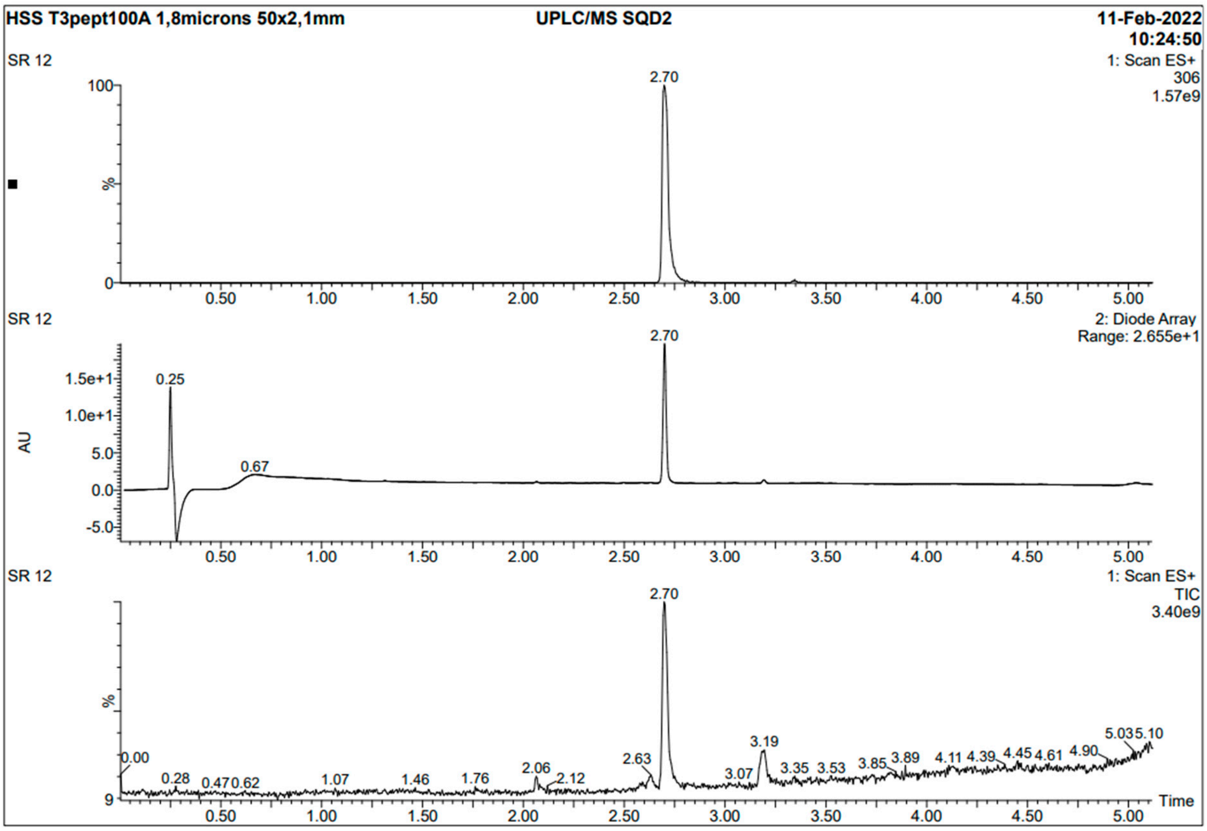

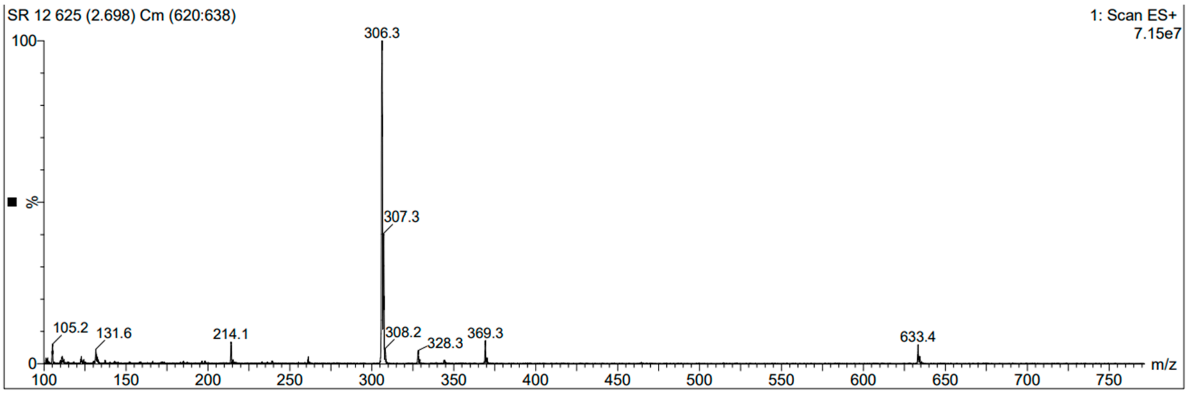

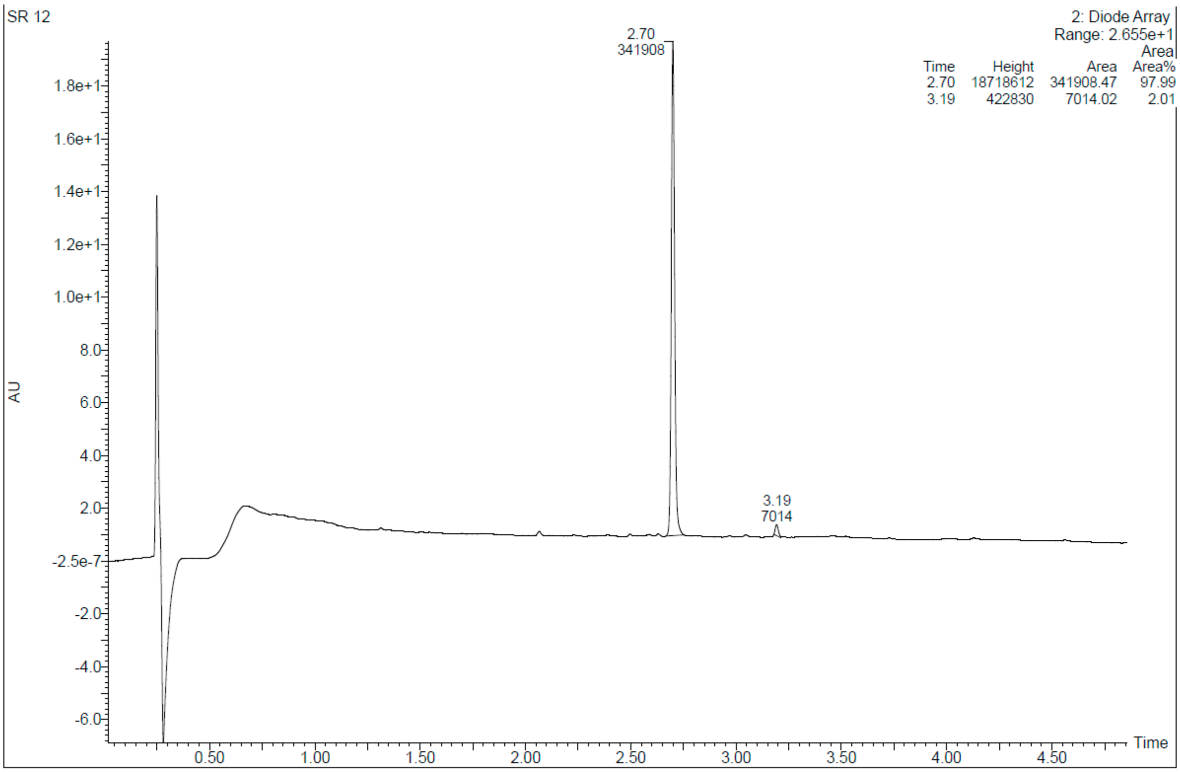

Acquisition Parameter

Source Type	ESI	Ion Polarity	Positive	Scan Begin	50 m/z	Scan End	2200 m/z

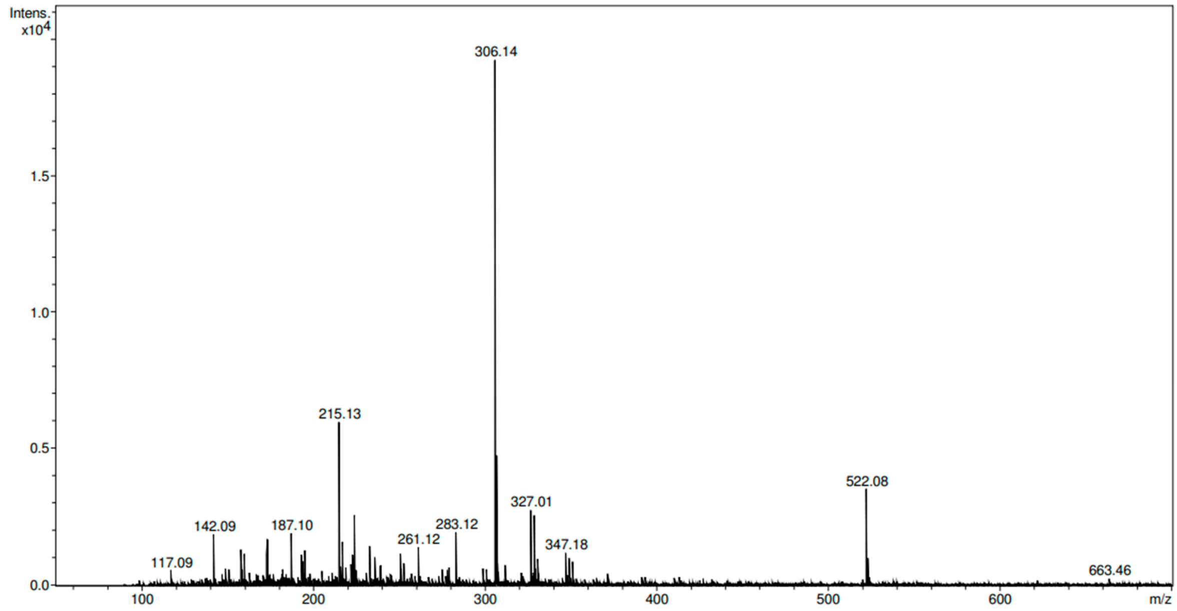

Acquisition Parameter

Source Type	ESI	Ion Polarity	Positive	Scan Begin	50 m/z	Scan End	2200 m/z

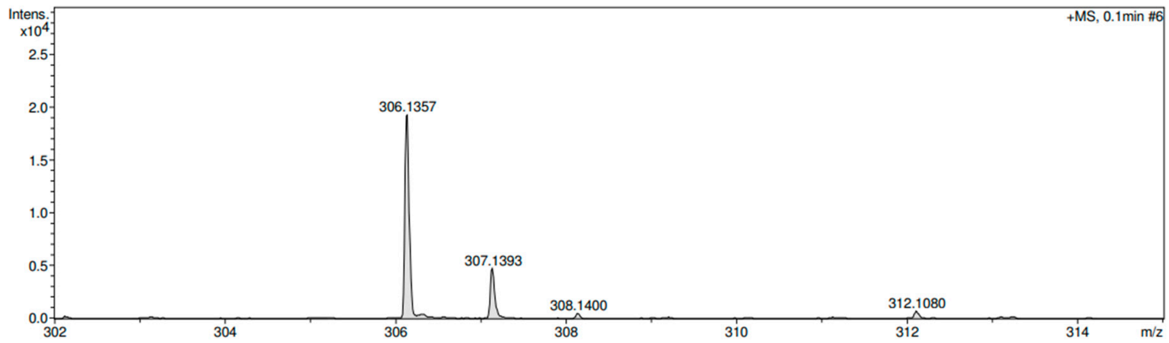

Meas. m/z	#	Ion Formula	m/z	err [ppm]	mSigma	# Sigma	Score	rdb	e⁻ Conf	N-Rule
306.1357	1	C17H16N5O	306.1349	-2.4	24.3	1	100.00	12.5	even	ok
	2	C16H20NO5	306.1336	-6.7	37.7	2	30.36	7.5	even	ok

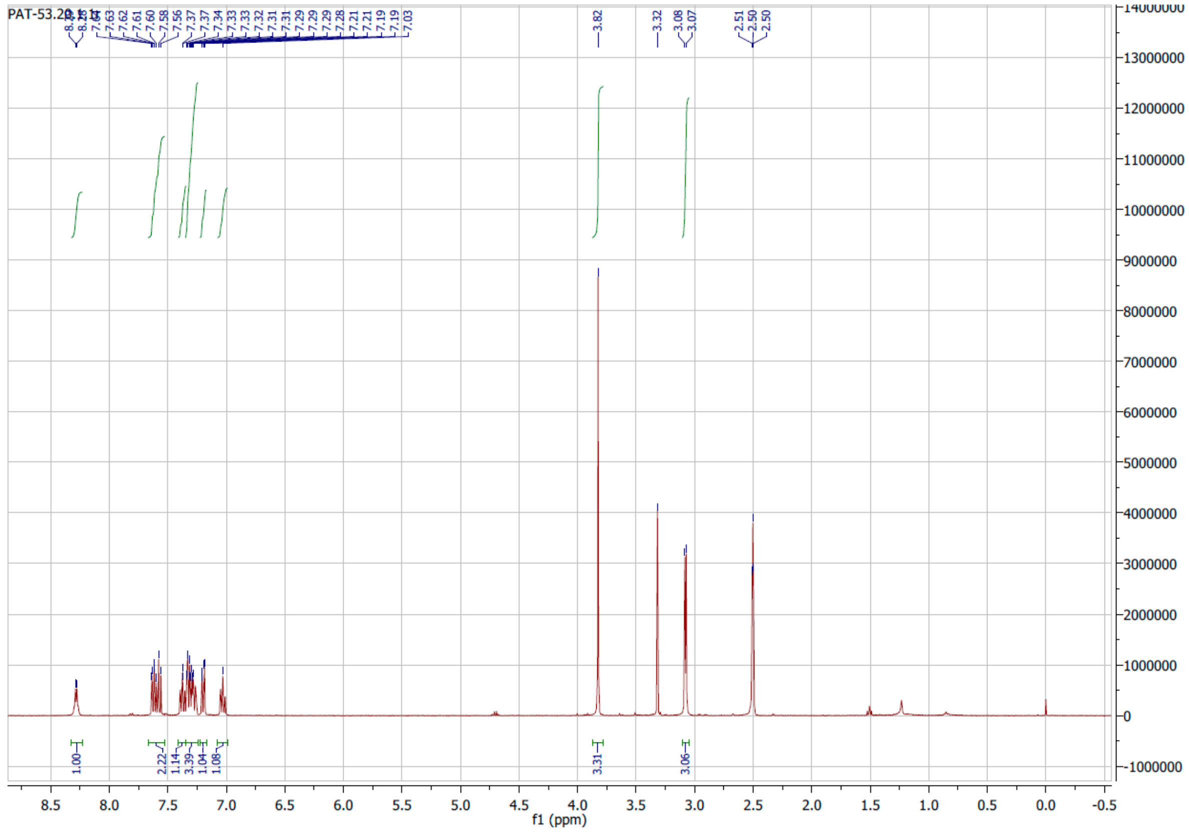

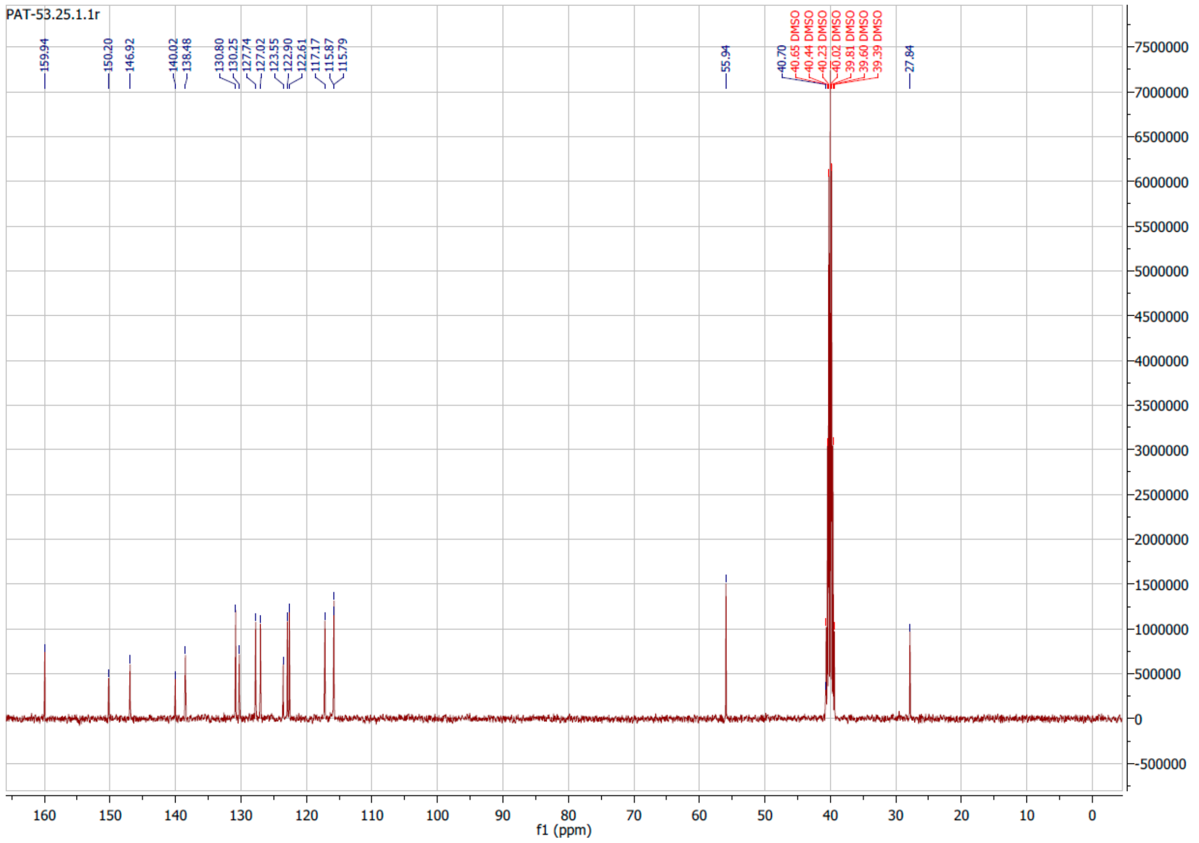

*S27. N-methyl-1-phenyl-[1,2,4]triazolo[4,3-a]quinoxalin-4-amine (**16c**).*

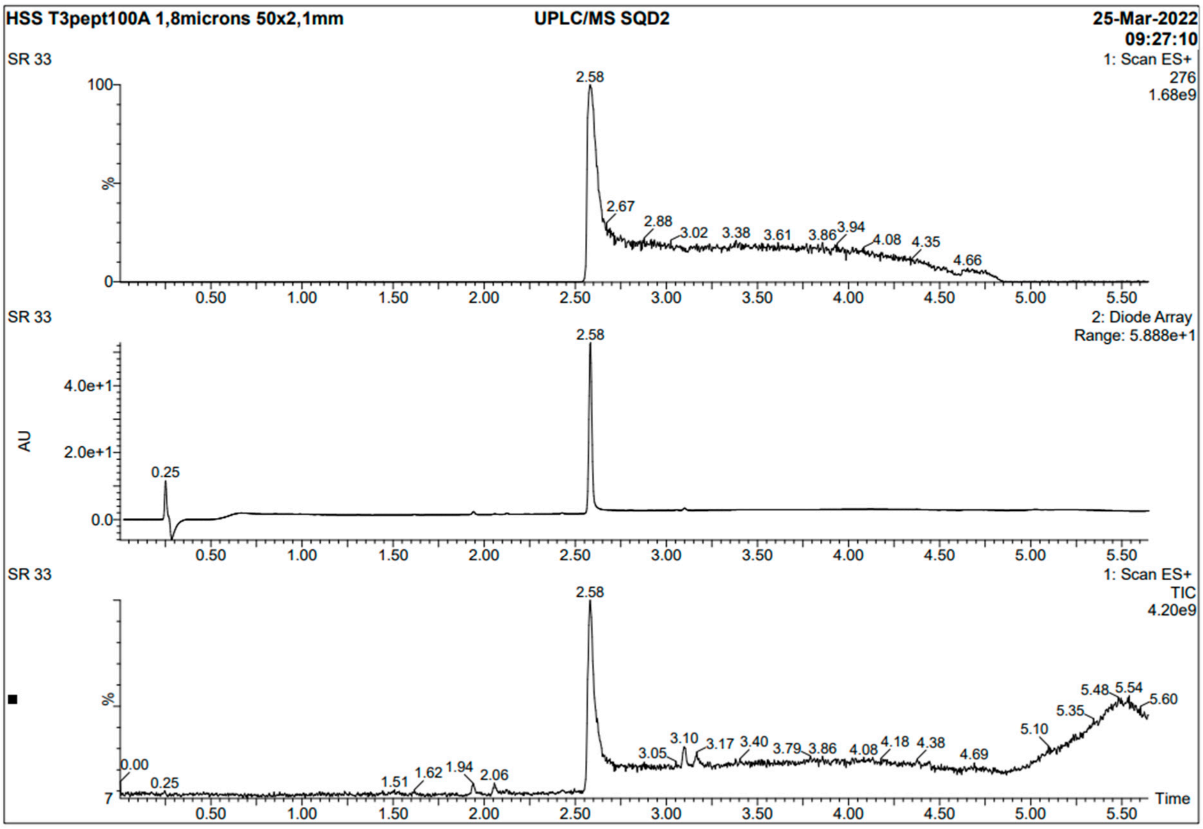

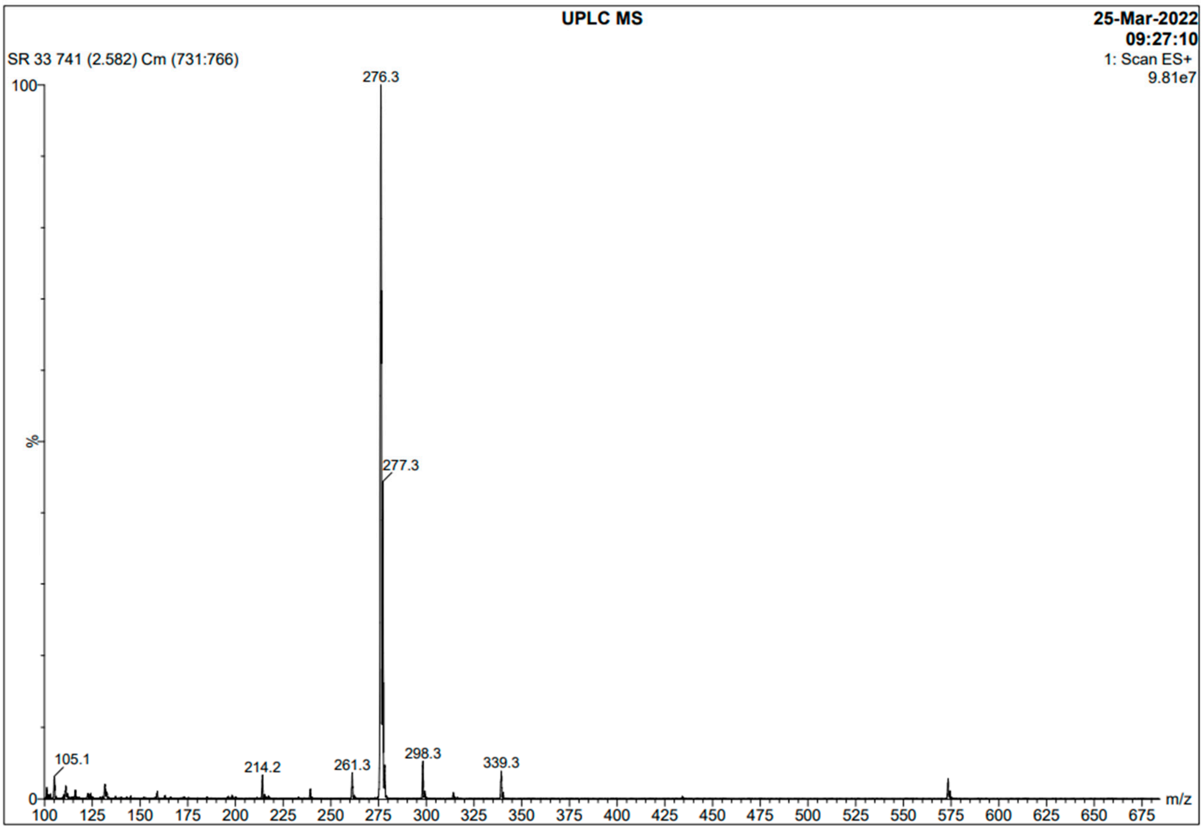

Acquisition Parameter

Source Type	ESI	Ion Polarity	Positive	Scan Begin	50 m/z	Scan End	2200 m/z

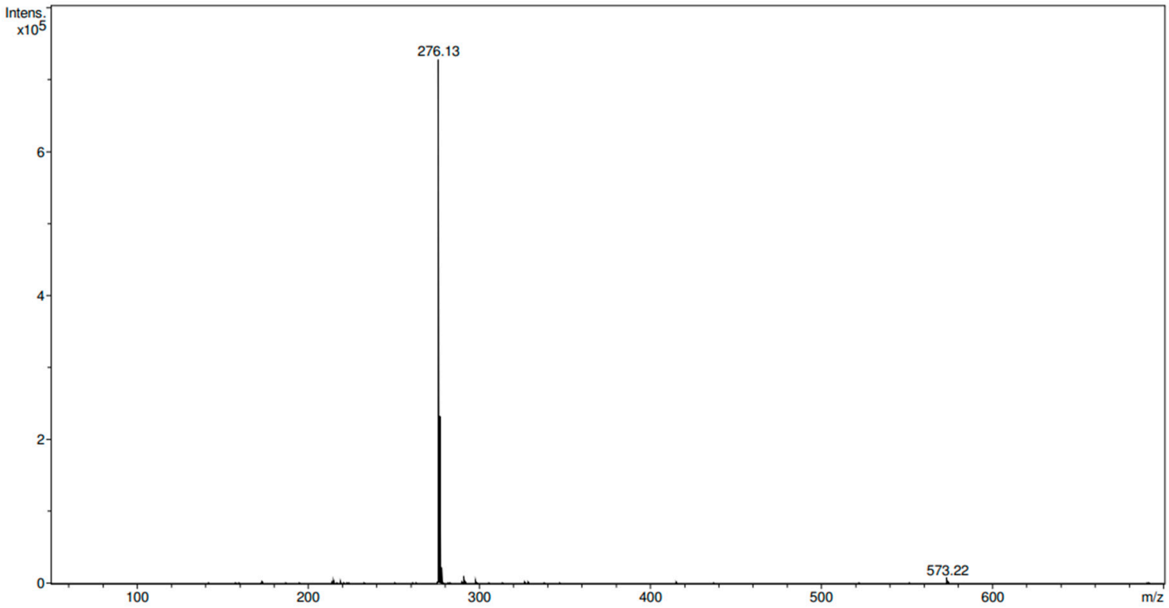

Acquisition Parameter

Source Type	ESI	Ion Polarity	Positive	Scan Begin	50 m/z	Scan End	2200 m/z

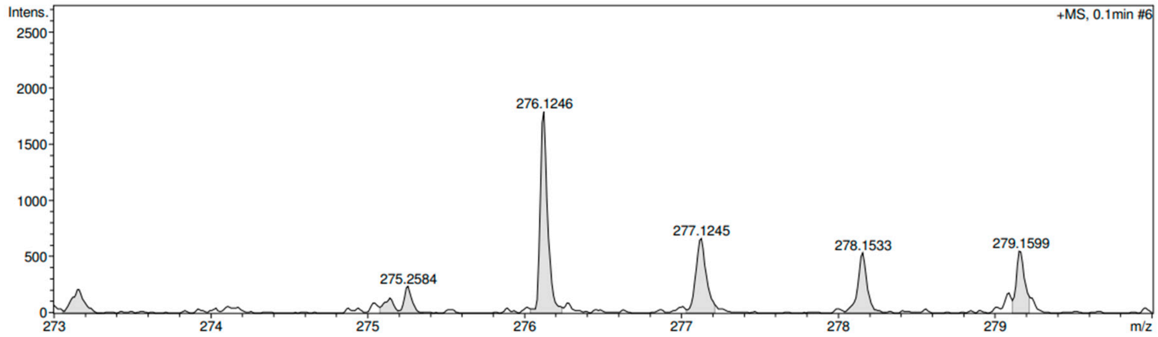

Meas. m/z	#	Ion Formula	m/z	err [ppm]	mSigma	# Sigma	Score	rdb	e⁻ Conf	N-Rule
276.1246	1	C16H14N5	276.1244	-0.7	193.1	1	100.00	12.5	even	ok
	2	C15H18NO4	276.1230	-5.6	198.9	2	29.82	7.5	even	ok

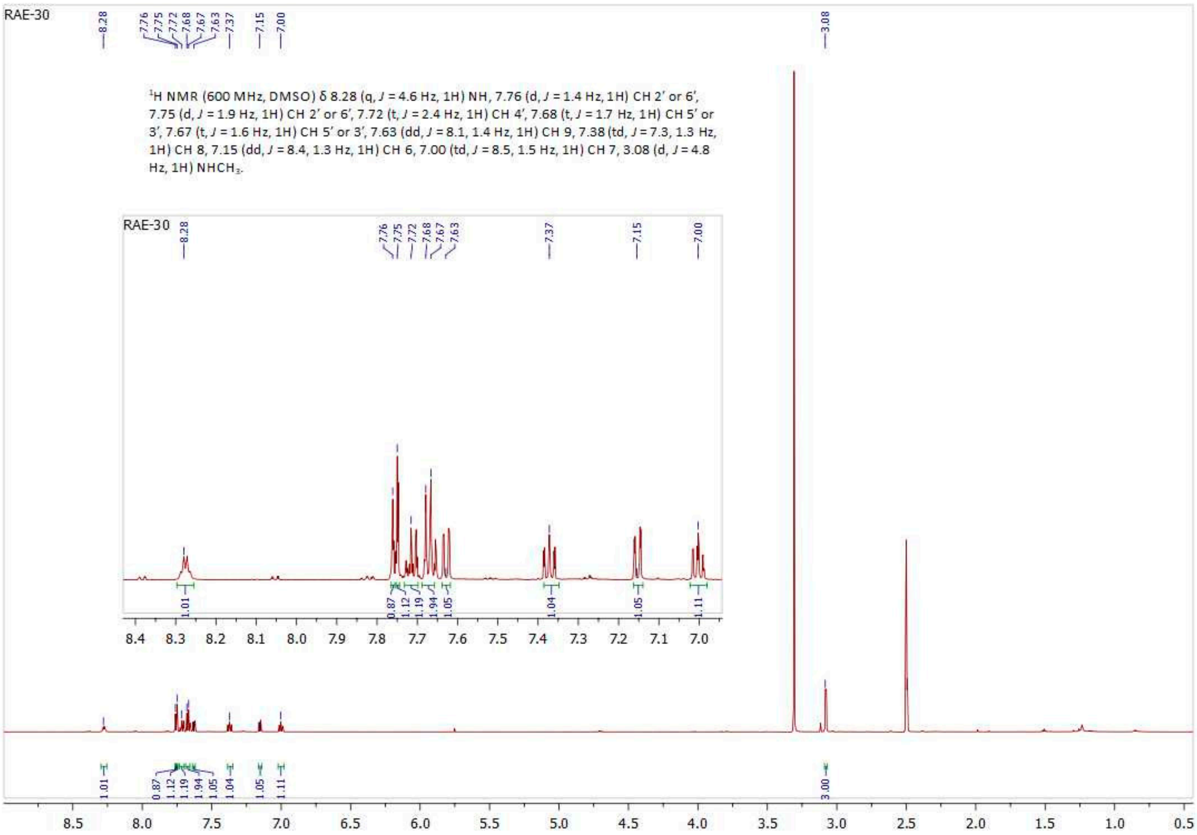

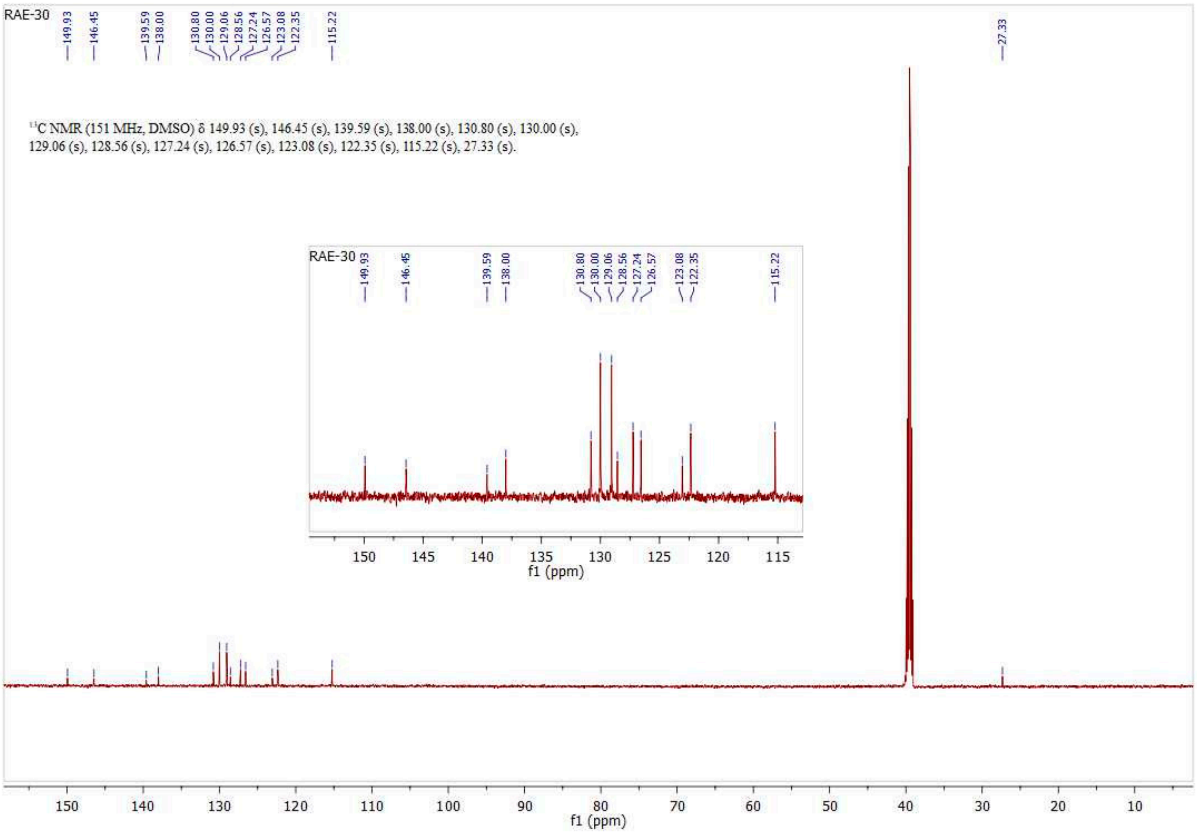

*S28. N-methyl-1-(3,4,5-trimethoxyphenyl)-[1,2,4]triazolo[4,3-a]quinoxalin-4-amine (**16d**).*

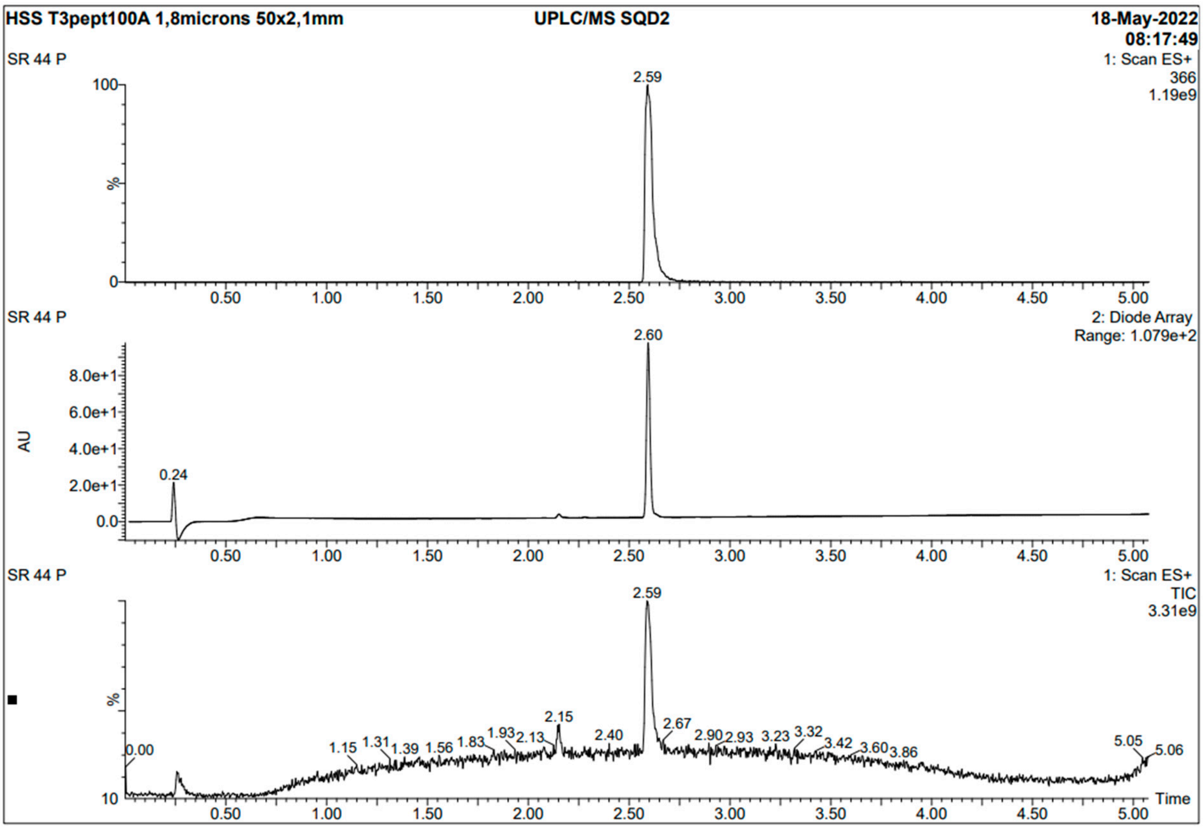

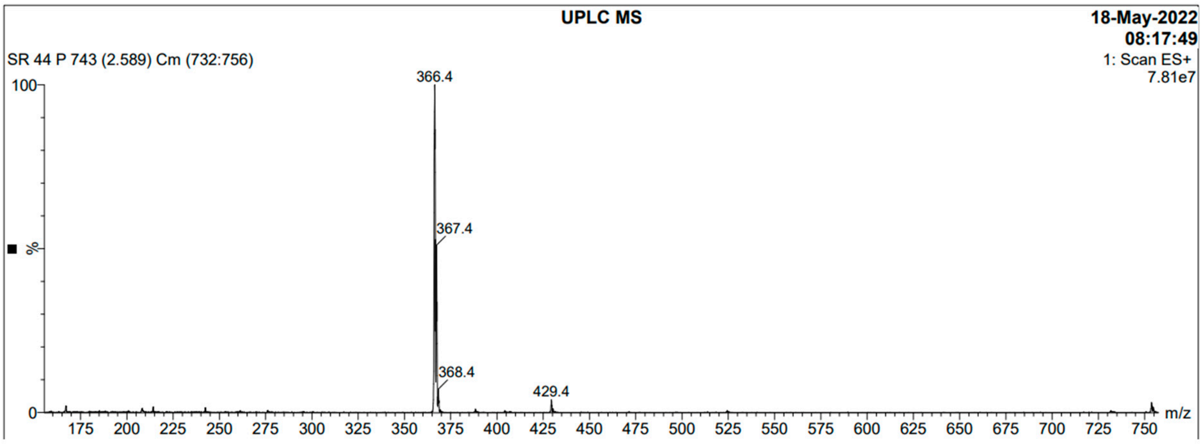

Acquisition Parameter

Source Type	ESI	Ion Polarity	Positive	Scan Begin	50 m/z	Scan End	2200 m/z

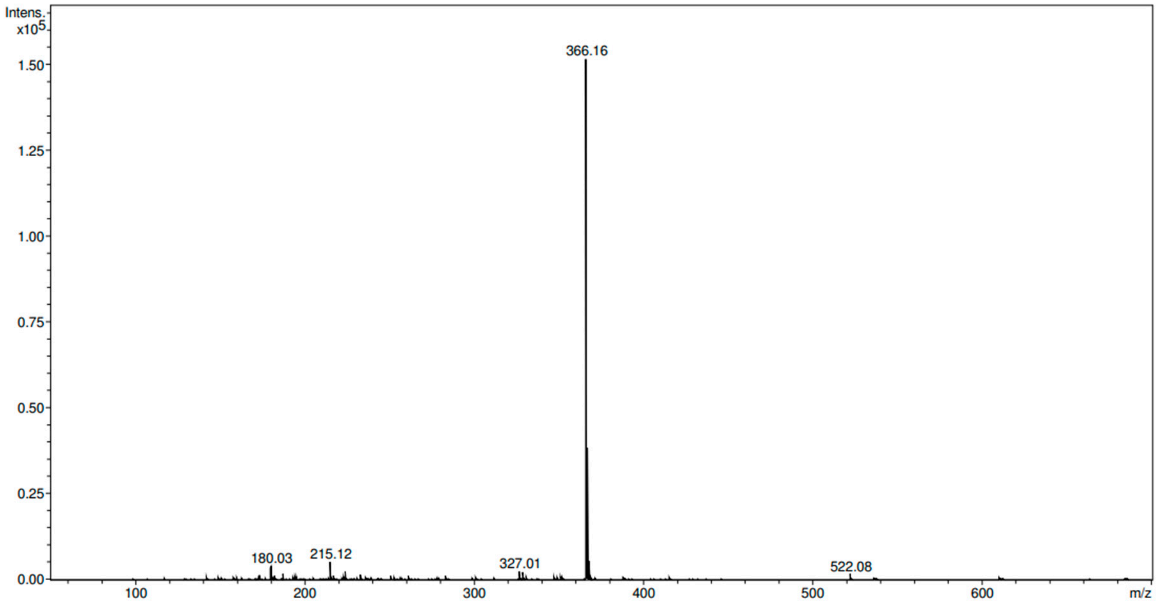

Acquisition Parameter

Source Type	ESI	Ion Polarity	Positive	Scan Begin	50 m/z	Scan End	2200 m/z

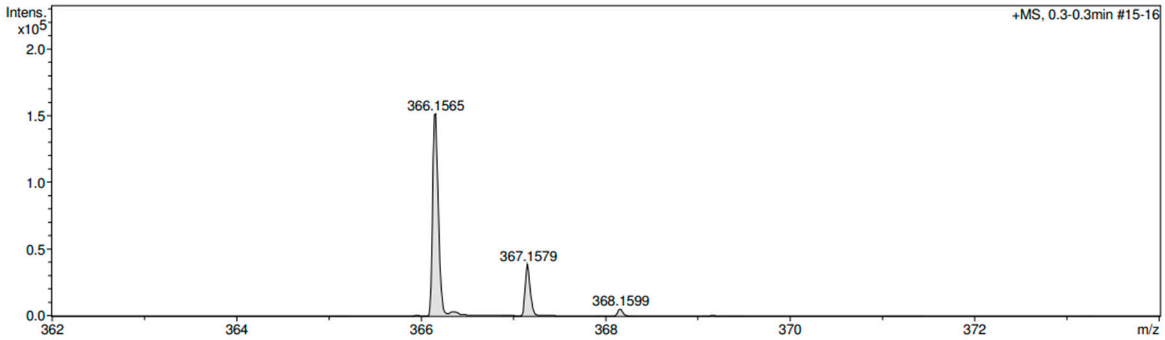

Meas. m/z	#	Ion Formula	m/z	err [ppm]	mSigma	# Sigma	Score	rdb	e⁻ Conf	N-Rule
366.1565	1	C19H20N5O3	366.1561	1.1	15.8	1	100.00	12.5	even	ok

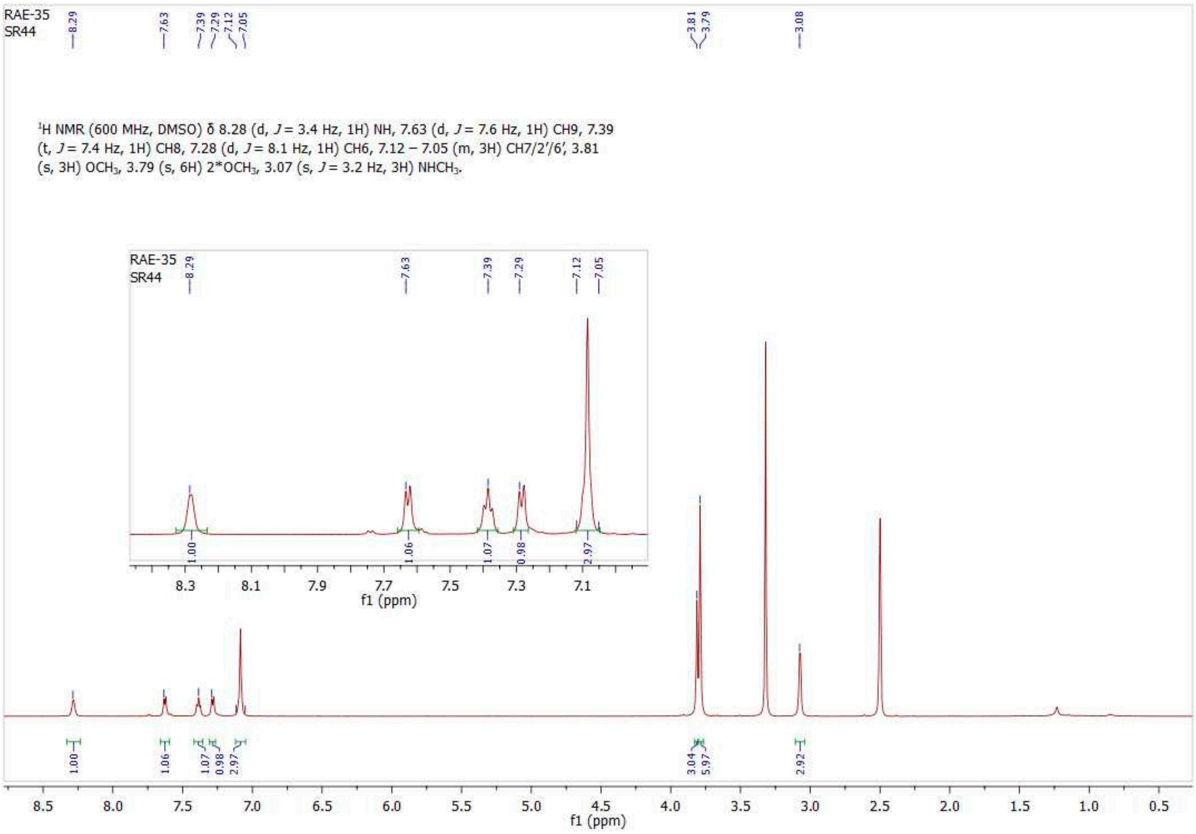
RAE-35
SR44
8.29
7.63
7.39
7.29
7.12
7.05
3.81
3.79
3.08
¹H NMR (600 MHz, DMSO) δ 8.28 (d, J = 3.4 Hz, 1H) NH, 7.63 (d, J = 7.6 Hz, 1H) CH9, 7.39 (t, J = 7.4 Hz, 1H) CH8, 7.28 (d, J = 8.1 Hz, 1H) CH6, 7.12 – 7.05 (m, 3H) CH7/2′/6′, 3.81 (s, 3H) OCH3, 3.79 (s, 6H) 2*OCH3, 3.07 (s, J = 3.2 Hz, 3H) NHCH3.
1.00
1.06
1.07
0.98
2.97
3.04
5.97
2.92
f1 (ppm)

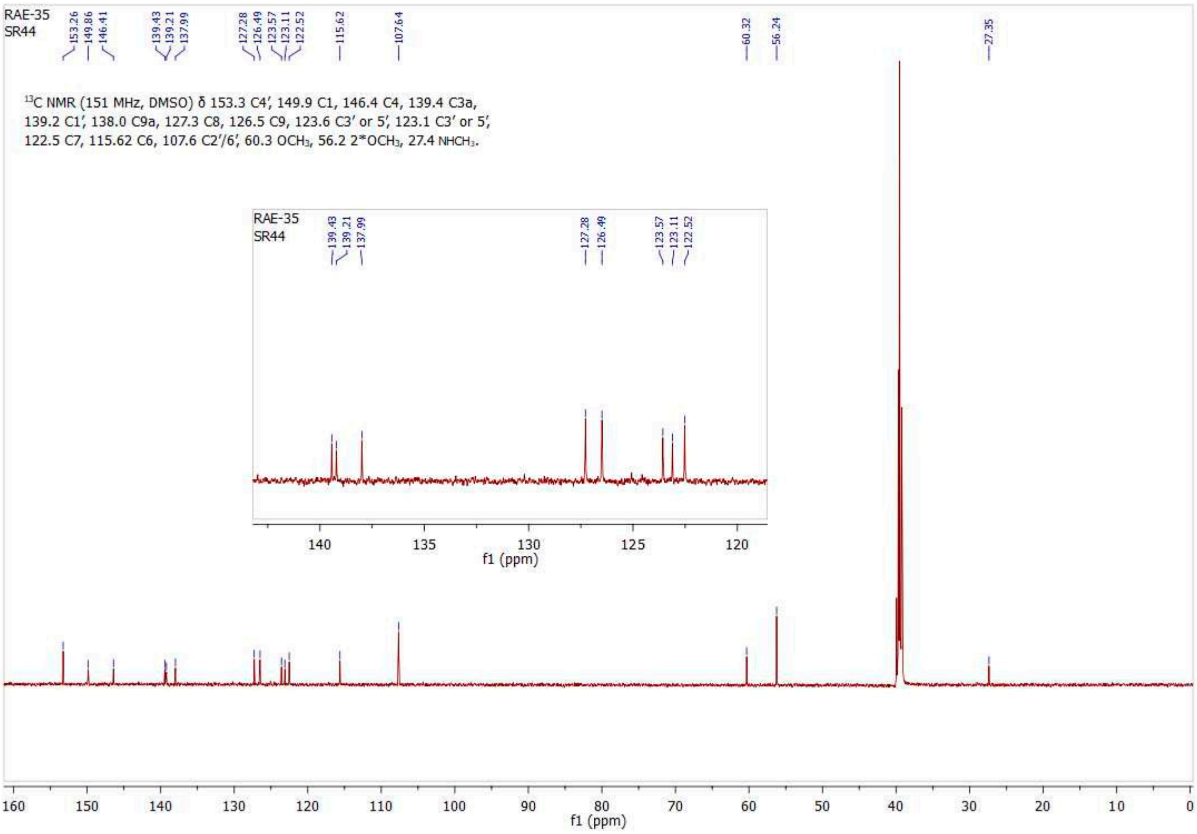
RAE-35
SR44
153.26
149.86
146.41
139.43
139.21
137.99
127.28
126.49
123.57
123.11
122.52
115.62
107.64
60.32
56.24
27.35
¹³C NMR (151 MHz, DMSO) δ 153.3 C4′, 149.9 C1, 146.4 C4, 139.4 C3a, 139.2 C1′, 138.0 C9a, 127.3 C8, 126.5 C9, 123.6 C3′ or 5′, 123.1 C3′ or 5′, 122.5 C7, 115.62 C6, 107.6 C2′/6′, 60.3 OCH3, 56.2 2*OCH3, 27.4 NHCH3.
f1 (ppm)

S29. *N-methyl-1-(pyridin-3-yl)-[1,2,4]triazolo[4,3-a]quinoxalin-4-amine* (***16e***).

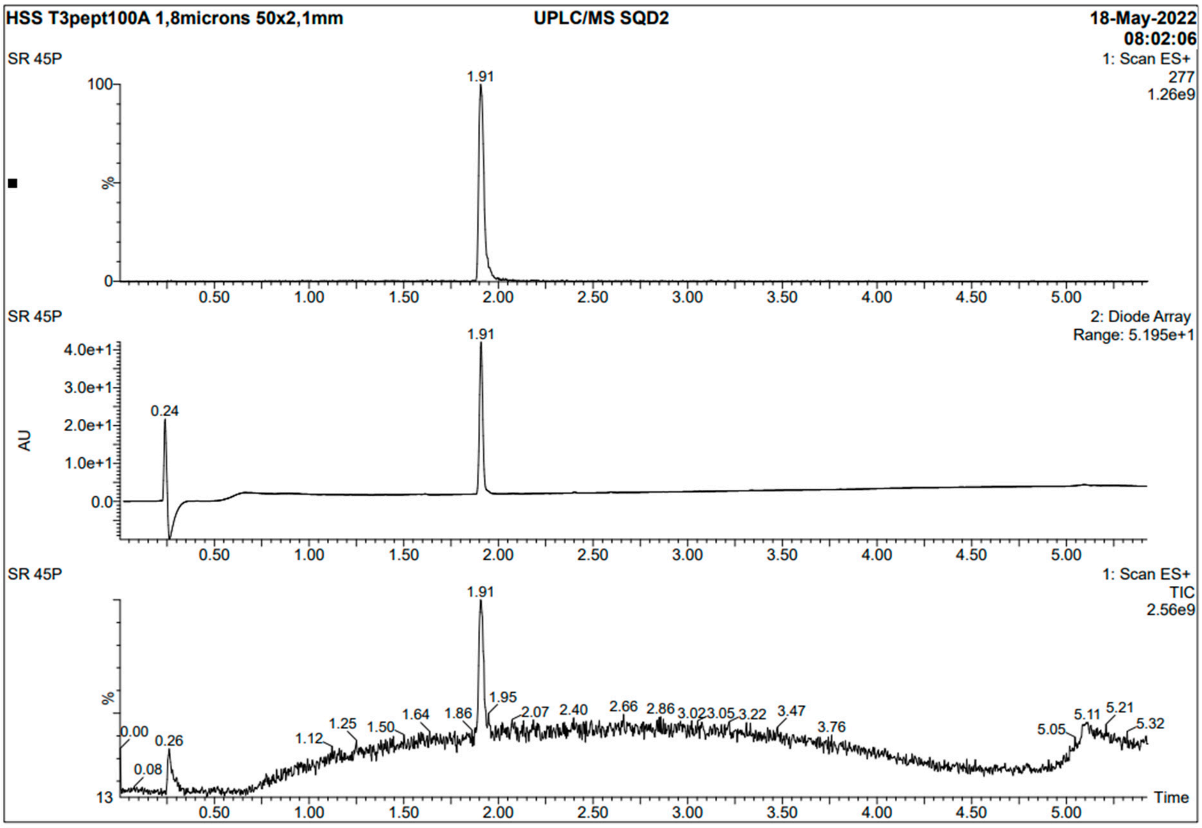

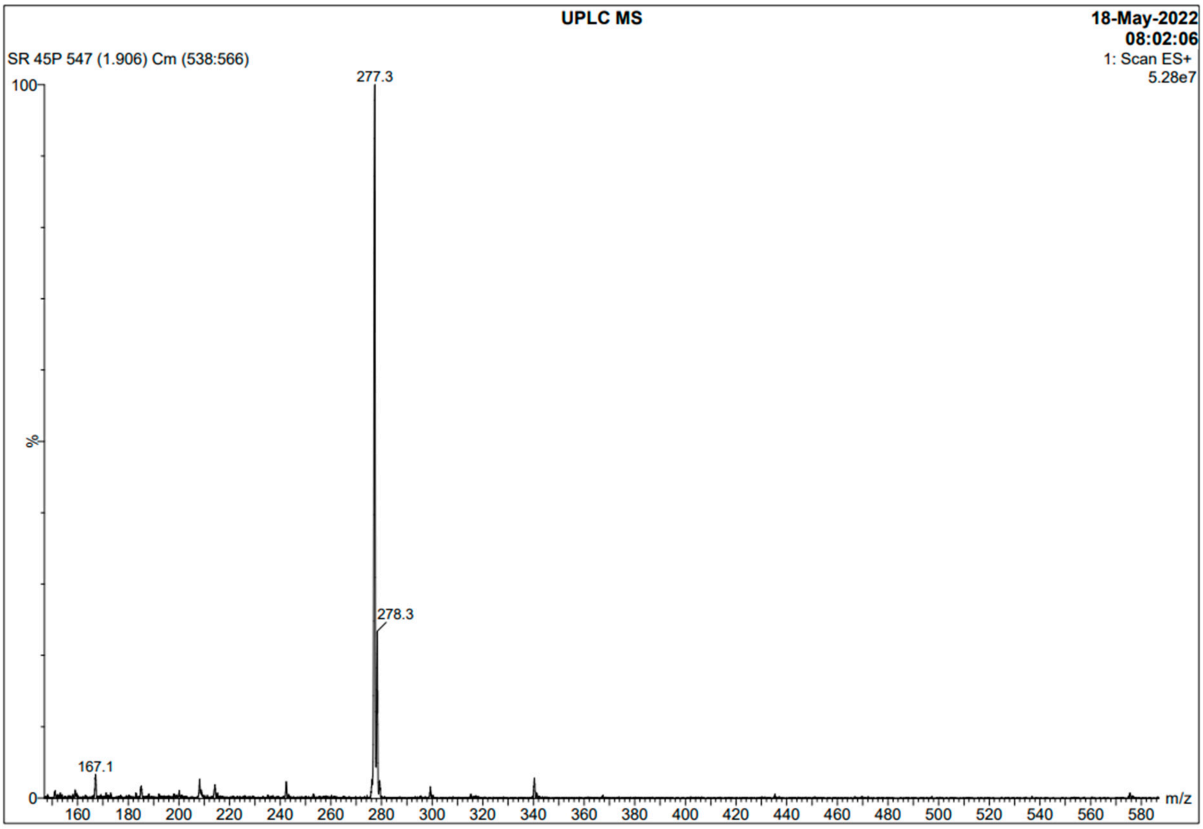

Acquisition Parameter

Source Type	ESI	Ion Polarity	Positive	Scan Begin	50 m/z	Scan End	2200 m/z

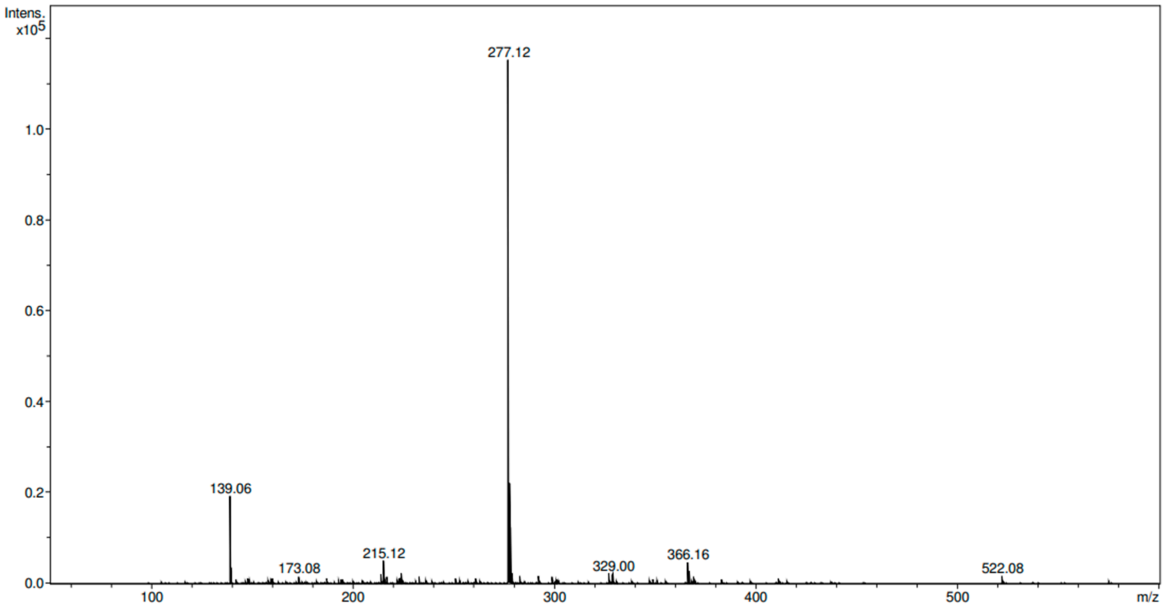

Acquisition Parameter

Source Type	ESI	Ion Polarity	Positive	Scan Begin	50 m/z	Scan End	2200 m/z

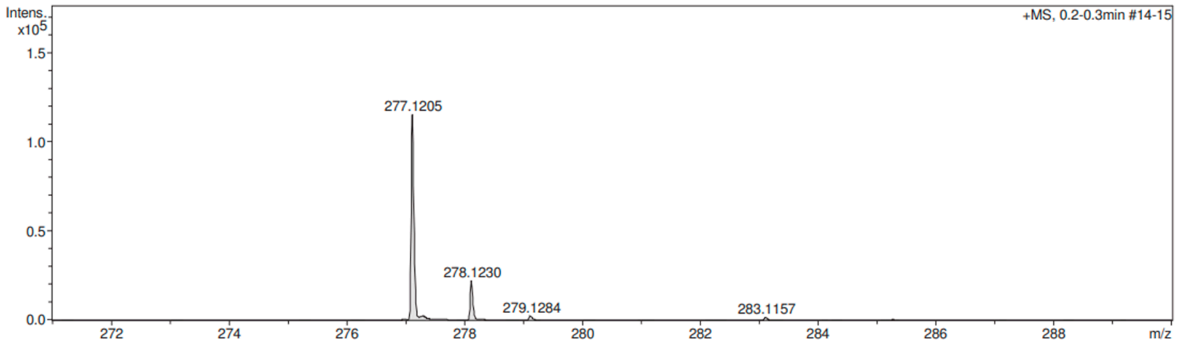

Meas. m/z	#	Ion Formula	m/z	err [ppm]	mSigma	# Sigma	Score	rdb	e⁻ Conf	N-Rule
277.1205	1	C15H13N6	277.1196	3.0	4.1	1	100.00	12.5	even	ok

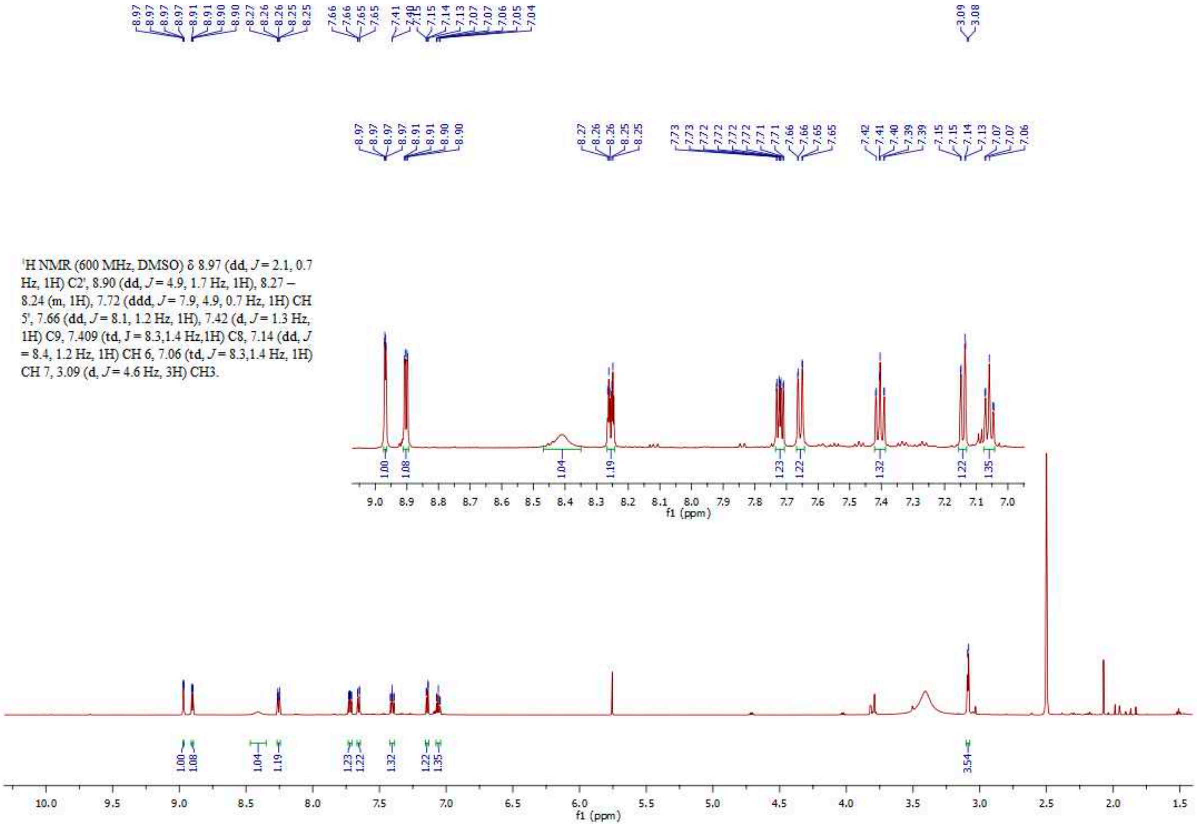

^{1}H NMR (600 MHz, DMSO) δ 8.97 (dd, J = 2.1, 0.7 Hz, 1H) C2', 8.90 (dd, J = 4.9, 1.7 Hz, 1H), 8.27 – 8.24 (m, 1H), 7.72 (ddd, J = 7.9, 4.9, 0.7 Hz, 1H) CH 5', 7.66 (dd, J = 8.1, 1.2 Hz, 1H), 7.42 (d, J = 1.3 Hz, 1H) C9, 7.409 (td, J = 8.3,1.4 Hz,1H) C8, 7.14 (dd, J = 8.4, 1.2 Hz, 1H) CH 6, 7.06 (td, J = 8.3,1.4 Hz, 1H) CH 7, 3.09 (d, J = 4.6 Hz, 3H) CH3.

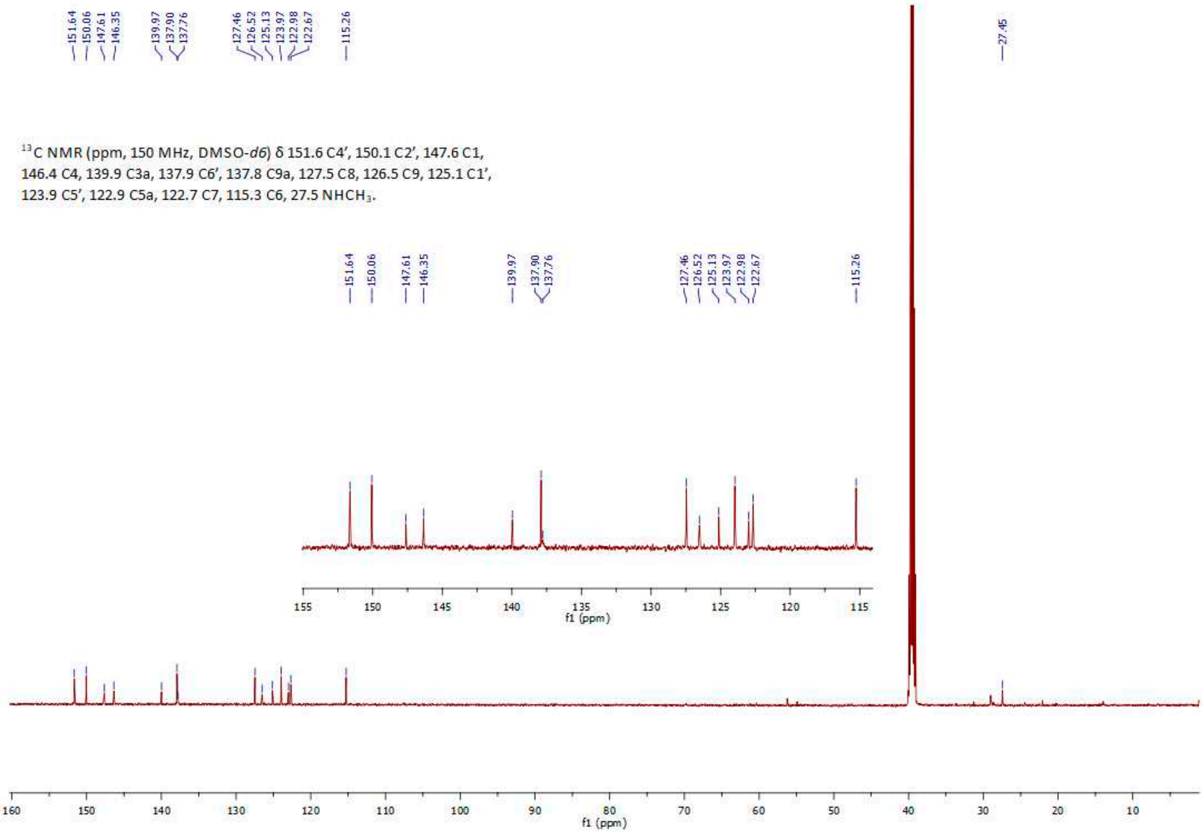

^{13}C NMR (ppm, 150 MHz, DMSO-*d6*) δ 151.6 C4′, 150.1 C2′, 147.6 C1, 146.4 C4, 139.9 C3a, 137.9 C6′, 137.8 C9a, 127.5 C8, 126.5 C9, 125.1 C1′, 123.9 C5′, 122.9 C5a, 122.7 C7, 115.3 C6, 27.5 $NHCH_3$.

S30. N-methyl-1-phenethyl-[1,2,4]triazolo[4,3-a]quinoxalin-4-amine (**16f**).

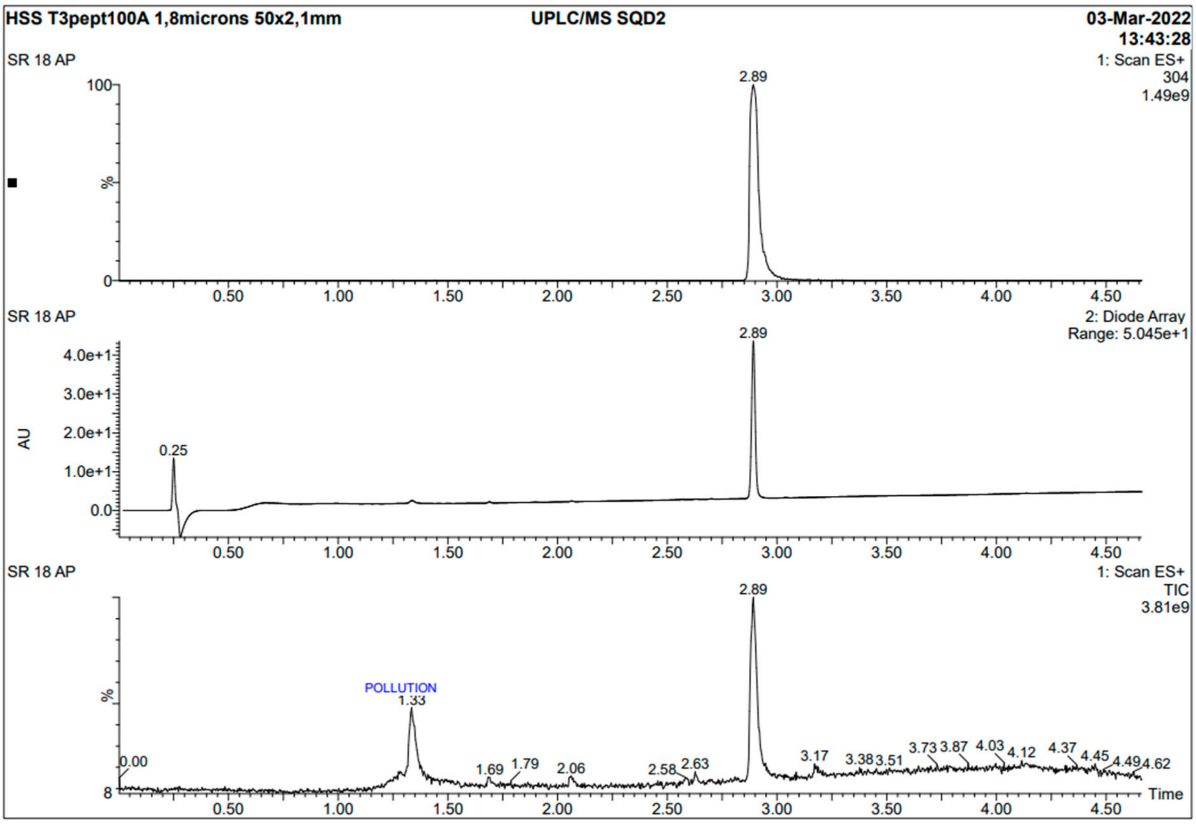

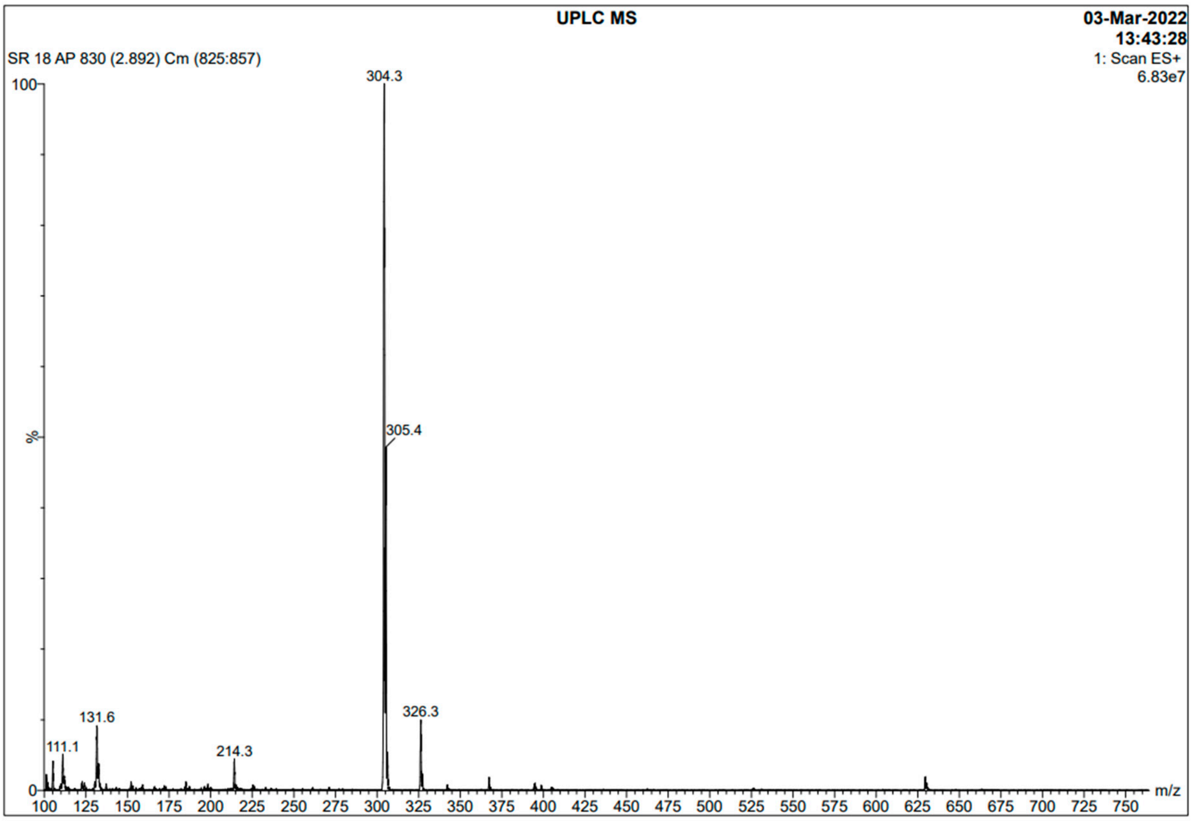

Acquisition Parameter

Source Type	ESI	Ion Polarity	Positive	Scan Begin	50 m/z	Scan End	2200 m/z

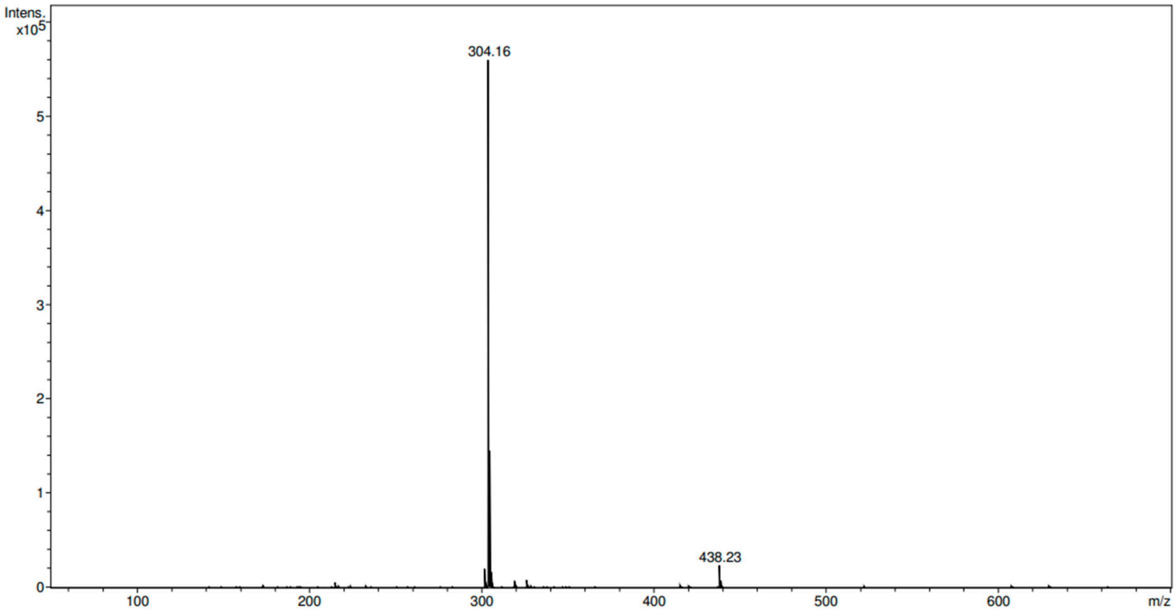

Acquisition Parameter

Source Type	ESI	Ion Polarity	Positive	Scan Begin	50 m/z	Scan End	2200 m/z

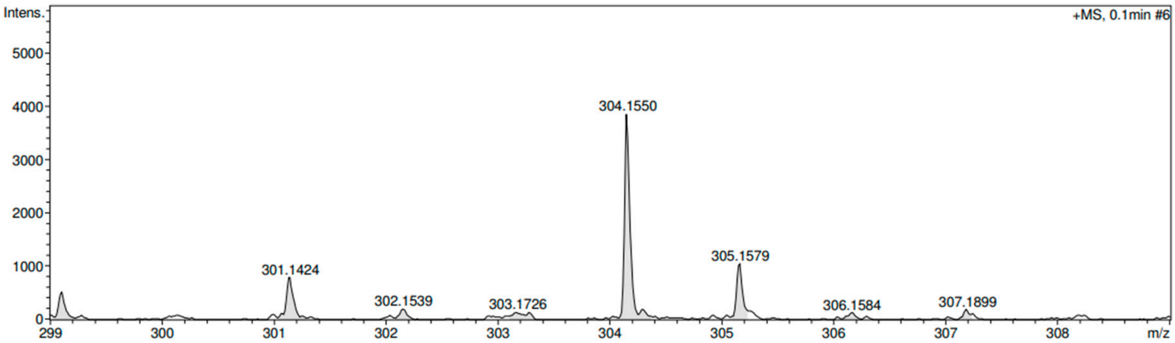

Meas. m/z	#	Ion Formula	m/z	err [ppm]	mSigma	# Sigma	Score	rdb	e⁻ Conf	N-Rule
304.1550	1	C18H18N5	304.1557	2.1	32.5	1	100.00	12.5	even	ok

molecules

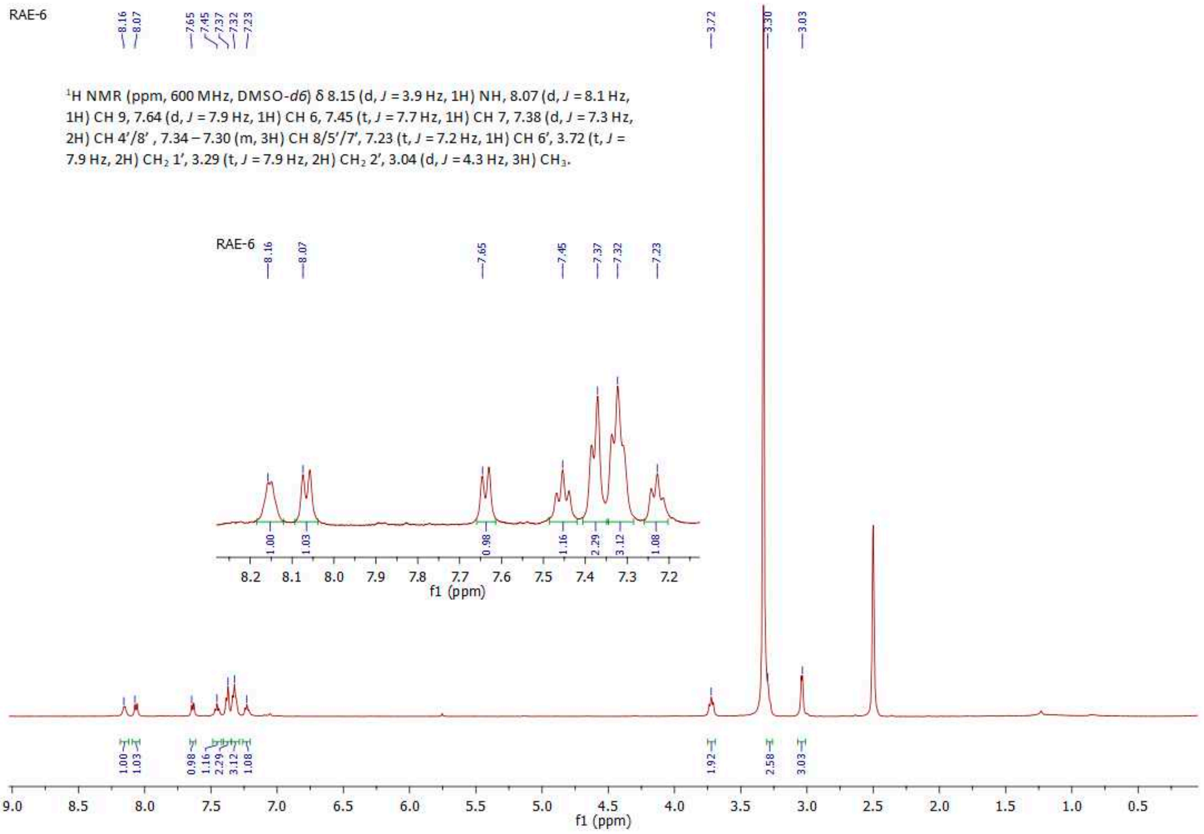
RAE-6
^{1}H NMR (ppm, 600 MHz, DMSO-*d6*) δ 8.15 (d, *J* = 3.9 Hz, 1H) NH, 8.07 (d, *J* = 8.1 Hz, 1H) CH 9, 7.64 (d, *J* = 7.9 Hz, 1H) CH 6, 7.45 (t, *J* = 7.7 Hz, 1H) CH 7, 7.38 (d, *J* = 7.3 Hz, 2H) CH 4′/8′ , 7.34 – 7.30 (m, 3H) CH 8/5′/7′, 7.23 (t, *J* = 7.2 Hz, 1H) CH 6′, 3.72 (t, *J* = 7.9 Hz, 2H) CH_2 1′, 3.29 (t, *J* = 7.9 Hz, 2H) CH_2 2′, 3.04 (d, *J* = 4.3 Hz, 3H) CH_3.
f1 (ppm)

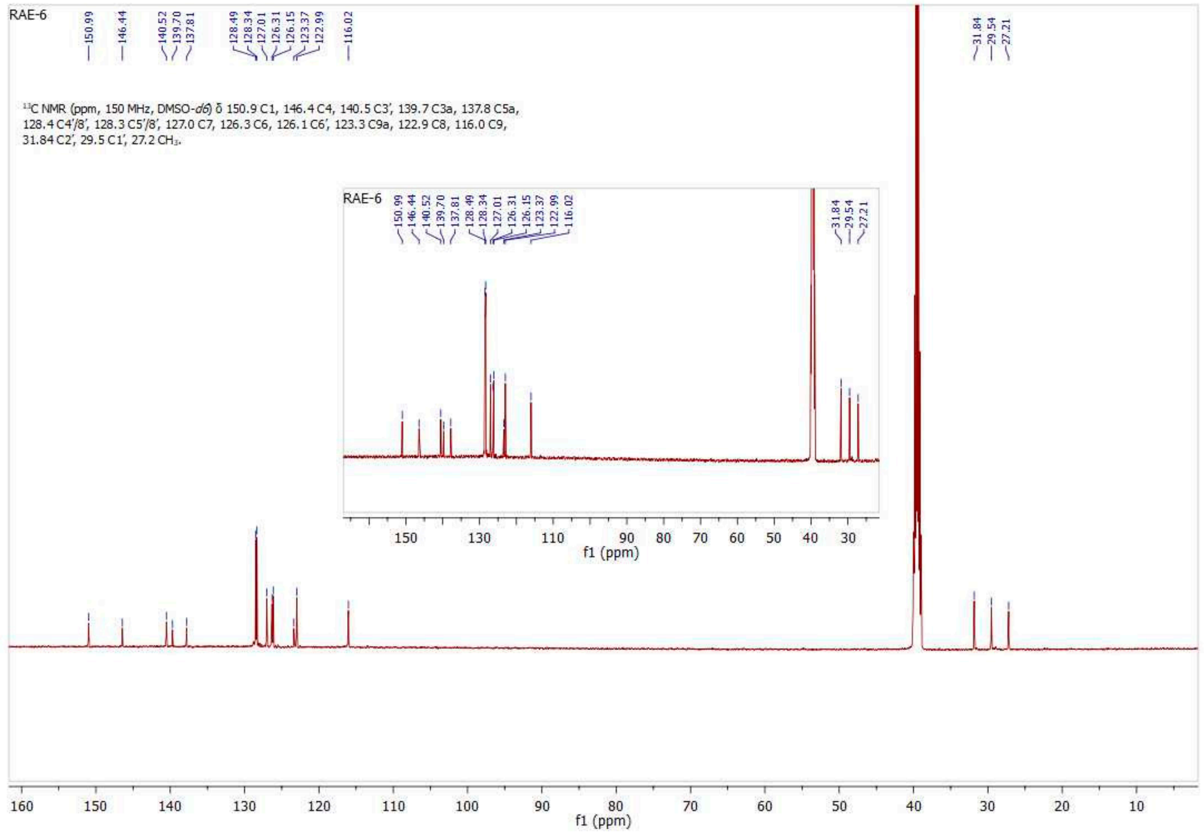
RAE-6
^{13}C NMR (ppm, 150 MHz, DMSO-*d6*) δ 150.9 C1, 146.4 C4, 140.5 C3′, 139.7 C3a, 137.8 C5a, 128.4 C4′/8′, 128.3 C5′/8′, 127.0 C7, 126.3 C6, 126.1 C6′, 123.3 C9a, 122.9 C8, 116.0 C9, 31.84 C2′, 29.5 C1′, 27.2 CH_3.
f1 (ppm)

S31. *1-butyl-N-methyl-[1,2,4]triazolo[4,3-a]quinoxalin-4-amine* (***16g***).

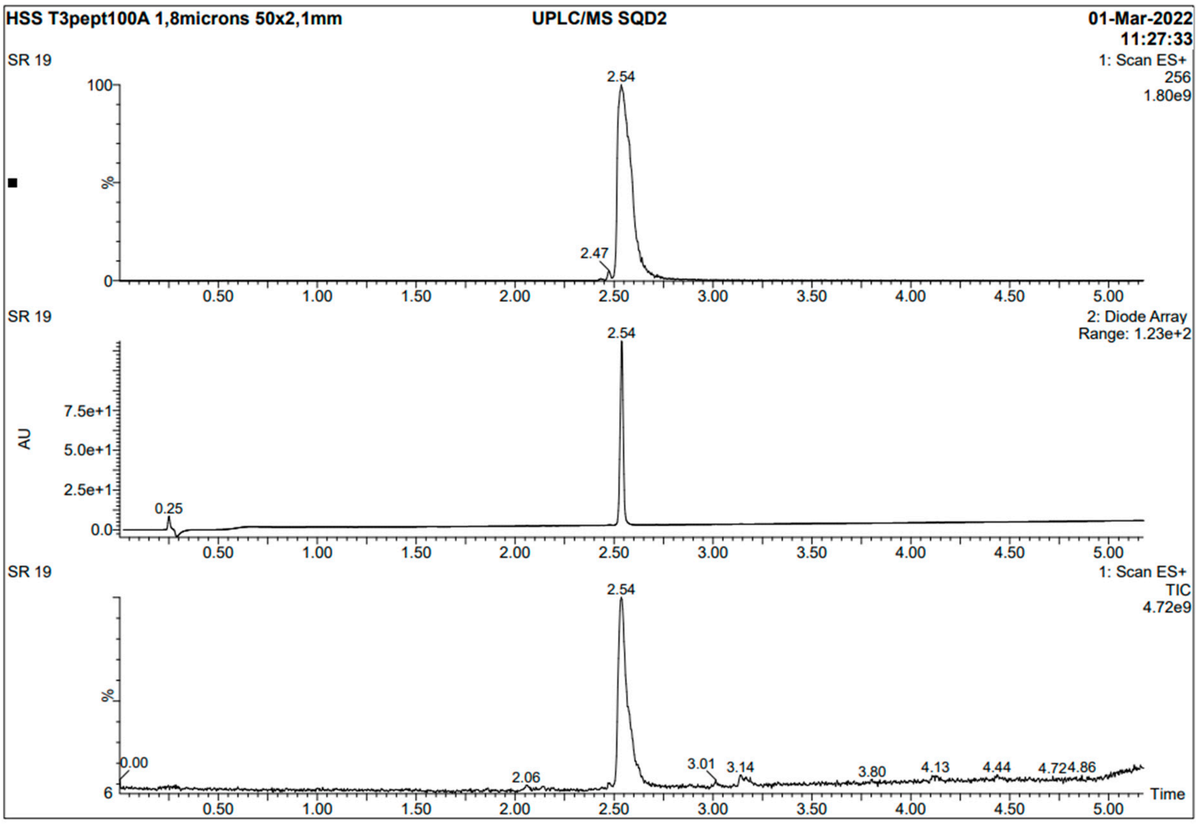

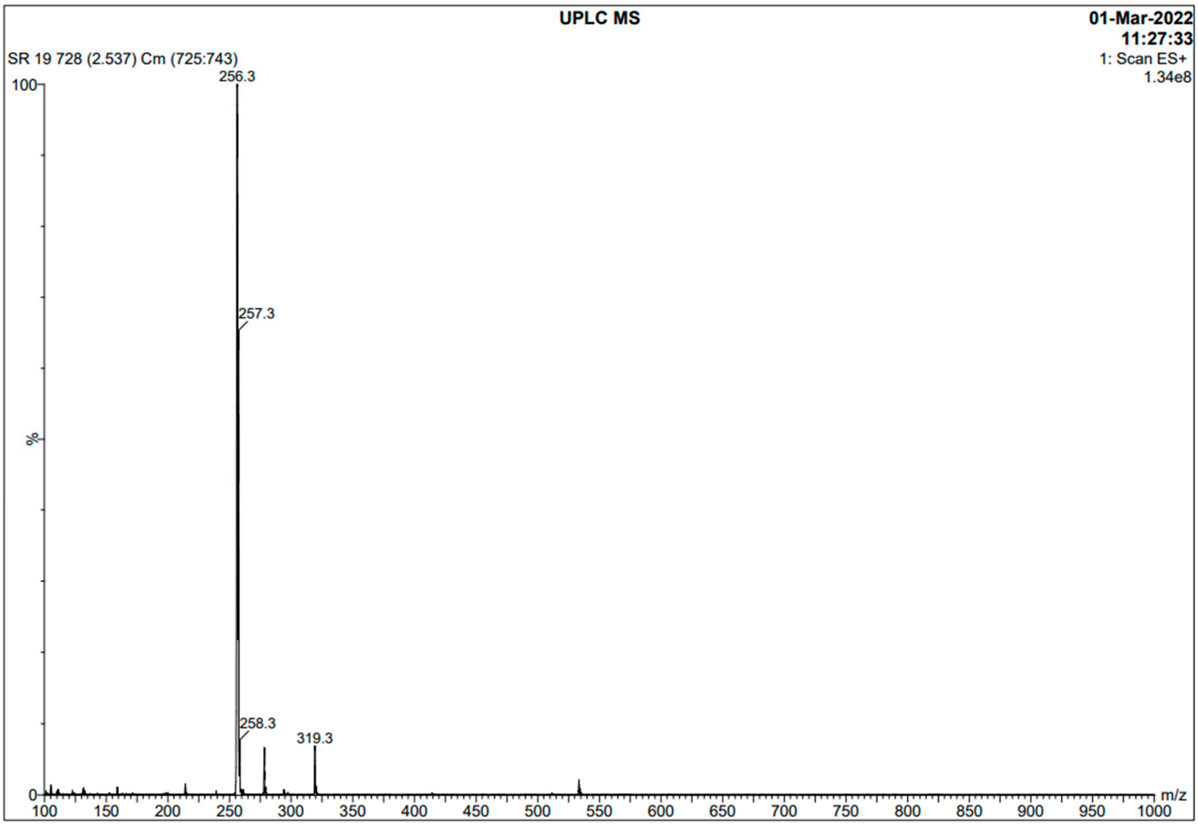

Acquisition Parameter

Source Type	ESI	Ion Polarity	Positive	Scan Begin	50 m/z	Scan End	2200 m/z

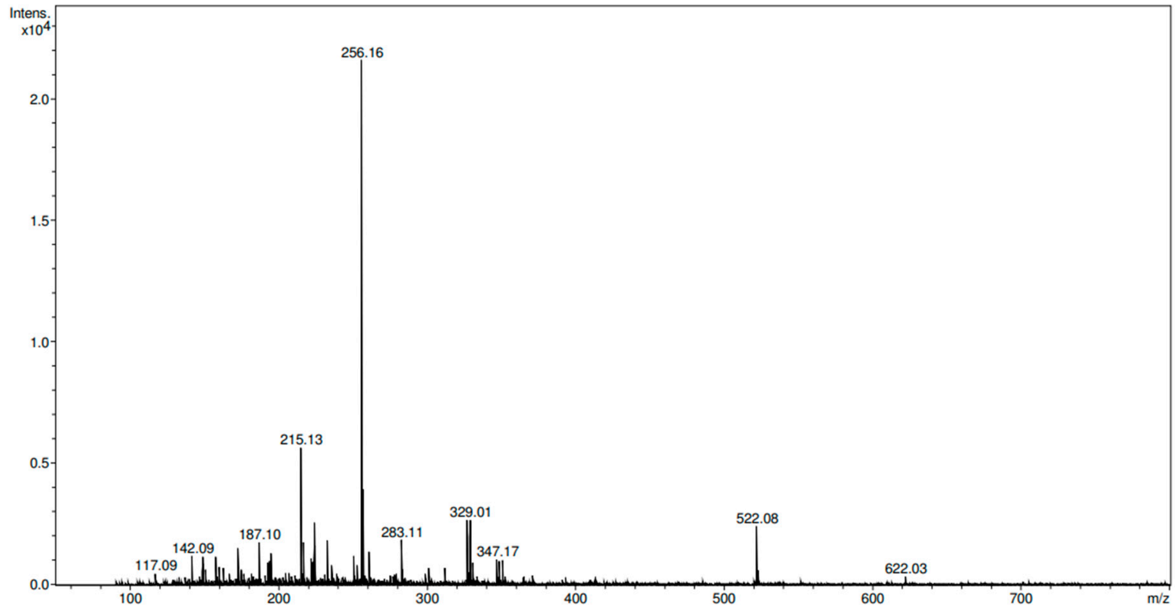

Acquisition Parameter

Source Type	ESI	Ion Polarity	Positive	Scan Begin	50 m/z	Scan End	2200 m/z

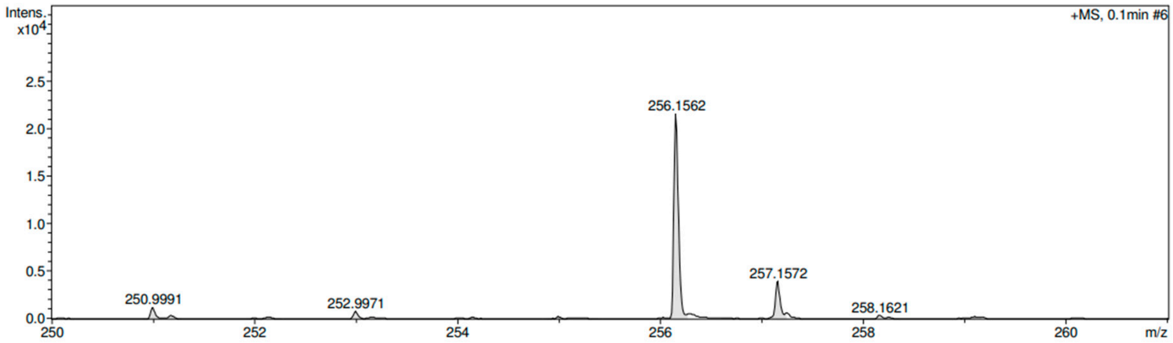

Meas. m/z	#	Ion Formula	m/z	err [ppm]	mSigma	# Sigma	Score	rdb	e⁻ Conf	N-Rule
256.1562	1	C14H18N5	256.1557	2.1	6.6	1	100.00	8.5	even	ok

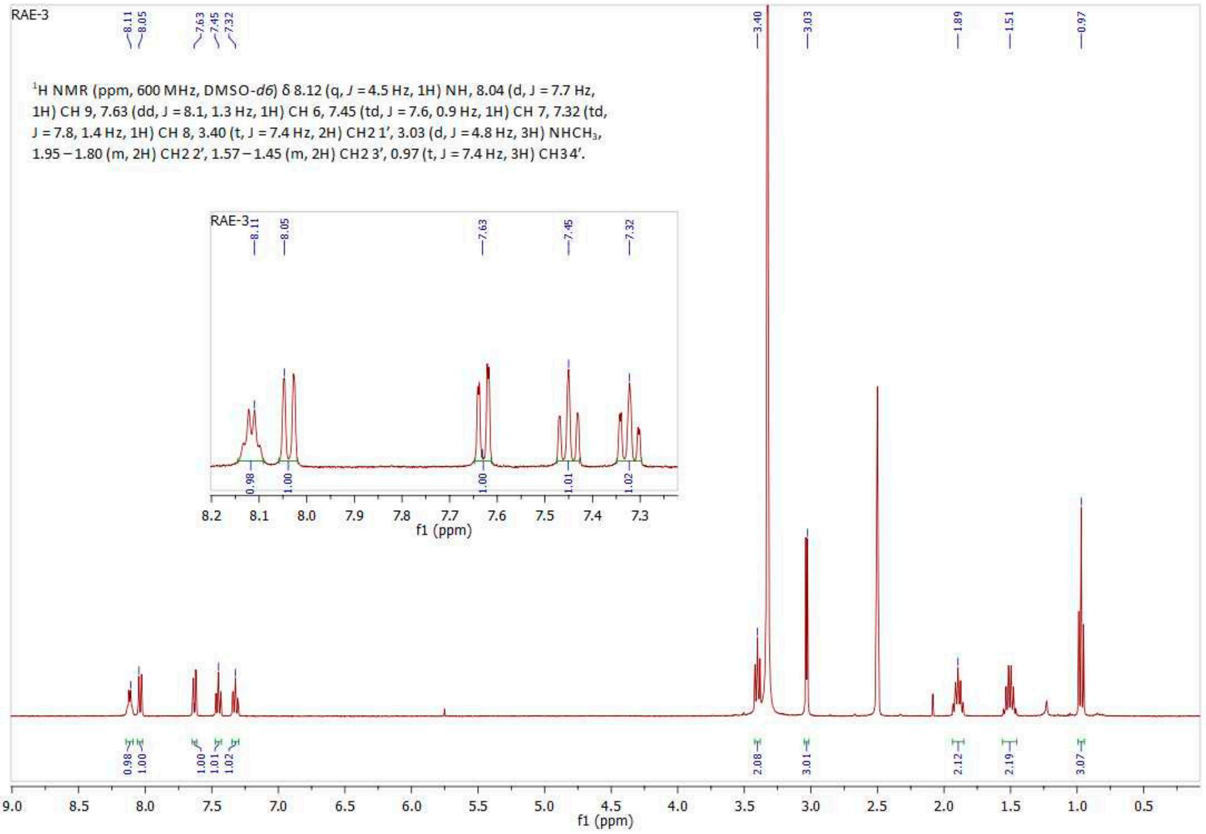
RAE-3
^{1}H NMR (ppm, 600 MHz, DMSO-d6) δ 8.12 (q, J = 4.5 Hz, 1H) NH, 8.04 (d, J = 7.7 Hz, 1H) CH 9, 7.63 (dd, J = 8.1, 1.3 Hz, 1H) CH 6, 7.45 (td, J = 7.6, 0.9 Hz, 1H) CH 7, 7.32 (td, J = 7.8, 1.4 Hz, 1H) CH 8, 3.40 (t, J = 7.4 Hz, 2H) CH2 1′, 3.03 (d, J = 4.8 Hz, 3H) NHCH3, 1.95 – 1.80 (m, 2H) CH2 2′, 1.57 – 1.45 (m, 2H) CH2 3′, 0.97 (t, J = 7.4 Hz, 3H) CH3 4′.
f1 (ppm)

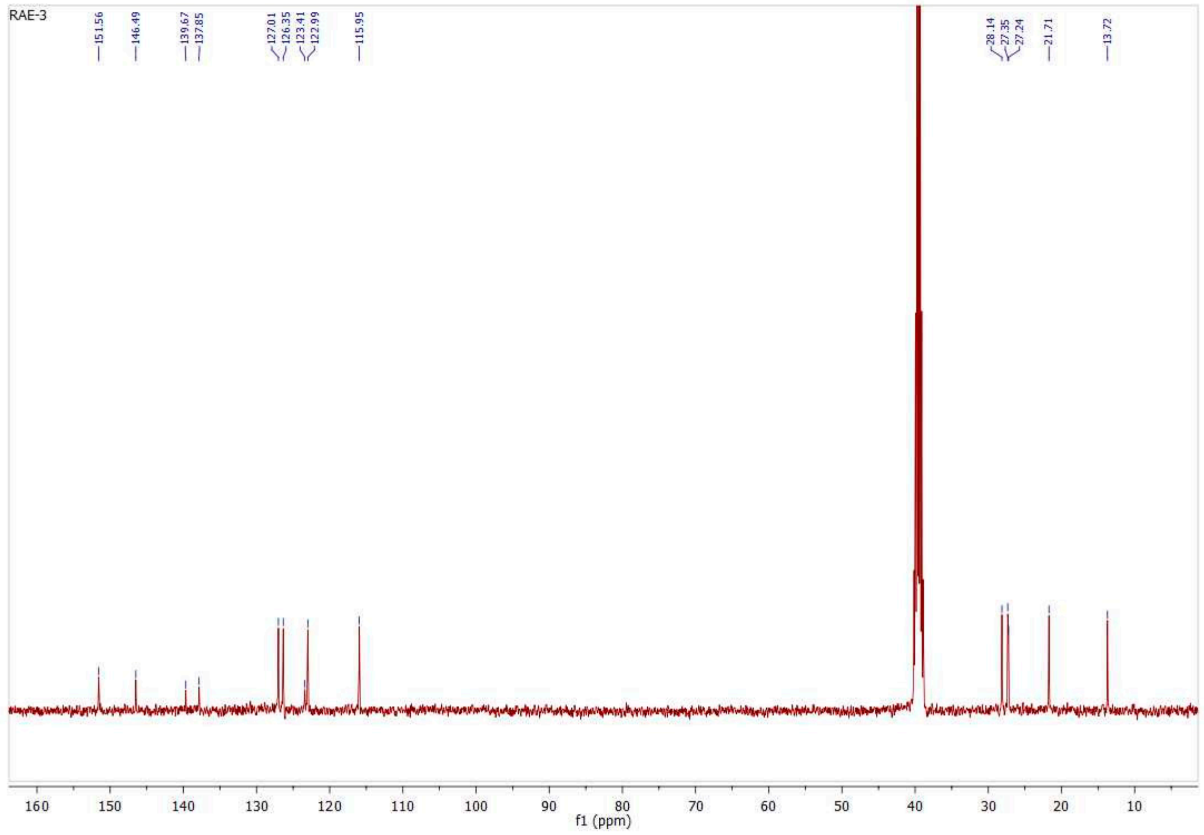
RAE-3
f1 (ppm)

S32. *1-isobutyl-N-methyl-[1,2,4]triazolo[4,3-a]quinoxalin-4-amine (**16h**).*

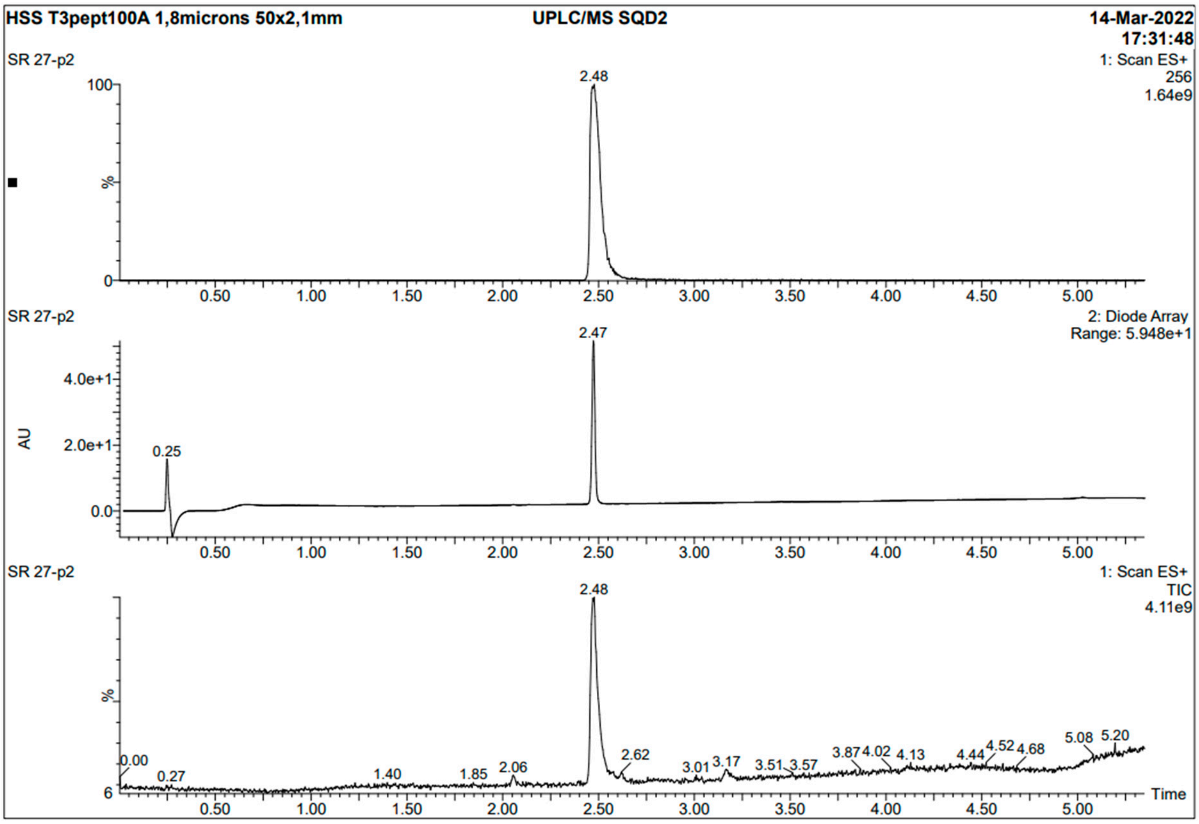

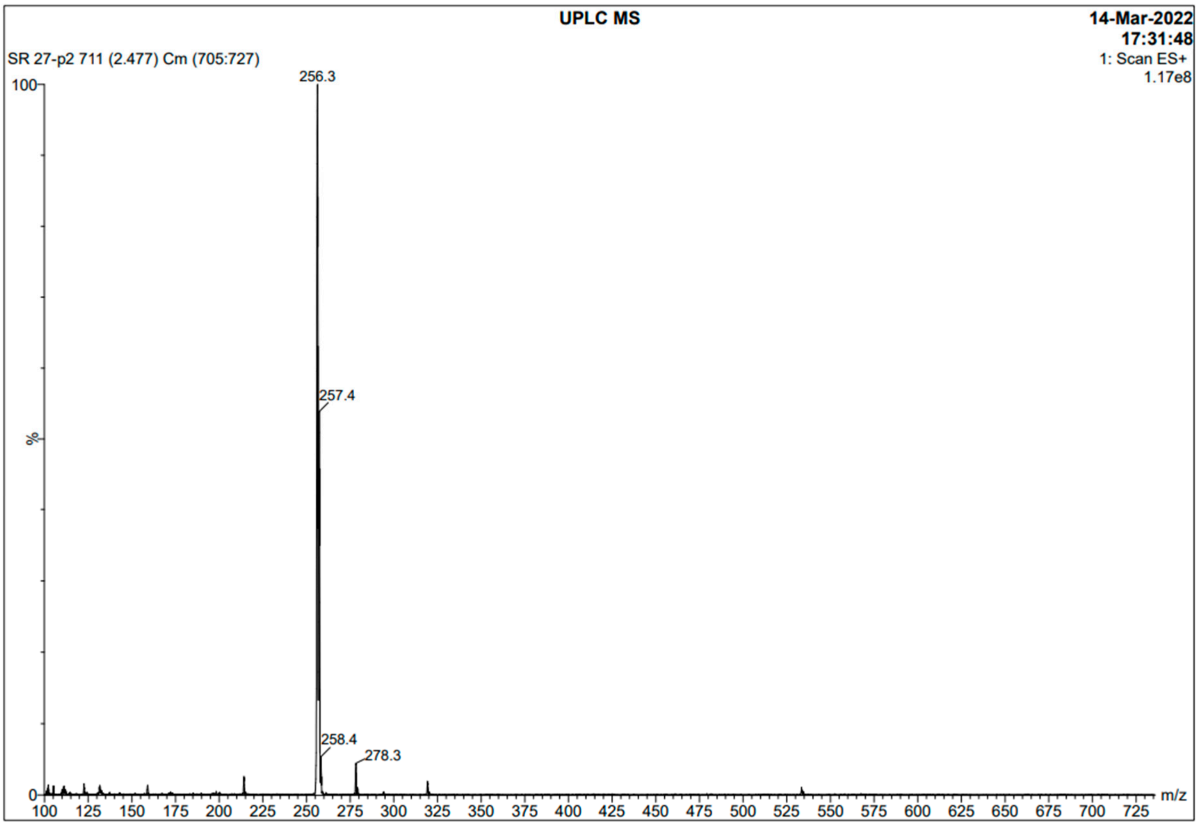

Acquisition Parameter

Source Type	ESI	Ion Polarity	Positive	Scan Begin	50 m/z	Scan End	2200 m/z

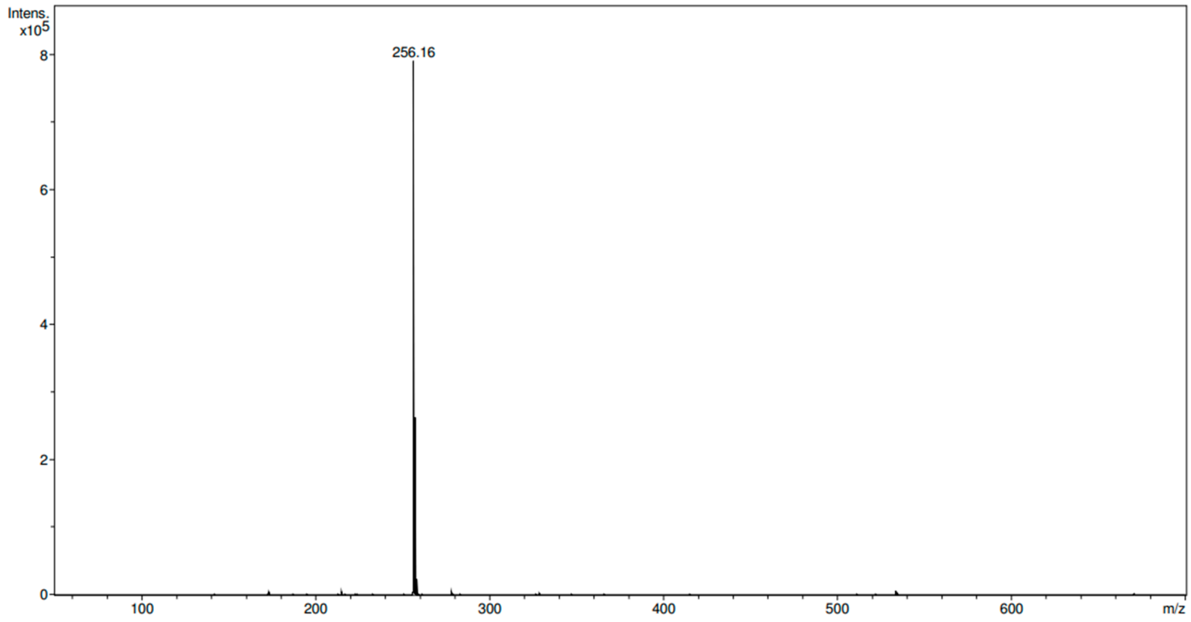

Acquisition Parameter

Source Type	ESI	Ion Polarity	Positive	Scan Begin	50 m/z	Scan End	2200 m/z

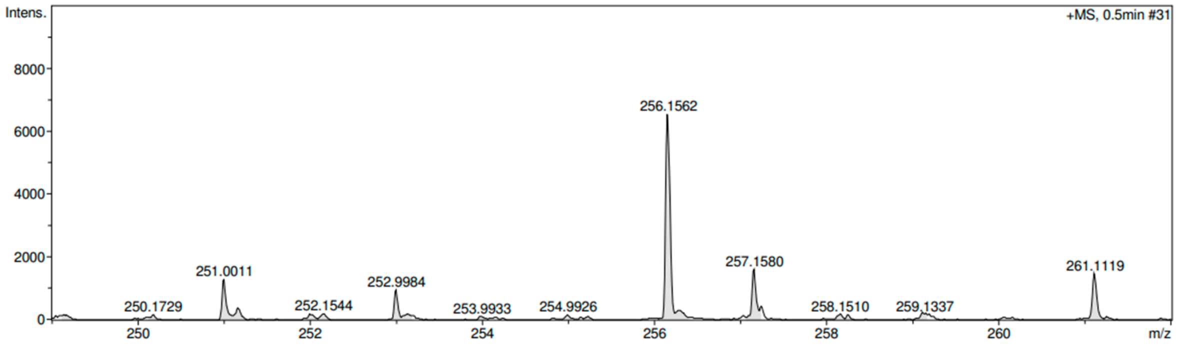

Meas. m/z	#	Ion Formula	m/z	err [ppm]	mSigma	# Sigma	Score	rdb	e⁻ Conf	N-Rule
256.1562	1	C14H18N5	256.1557	2.0	43.7	1	100.00	8.5	even	ok
	2	C13H22NO4	256.1543	-7.2	56.7	2	29.86	3.5	even	ok

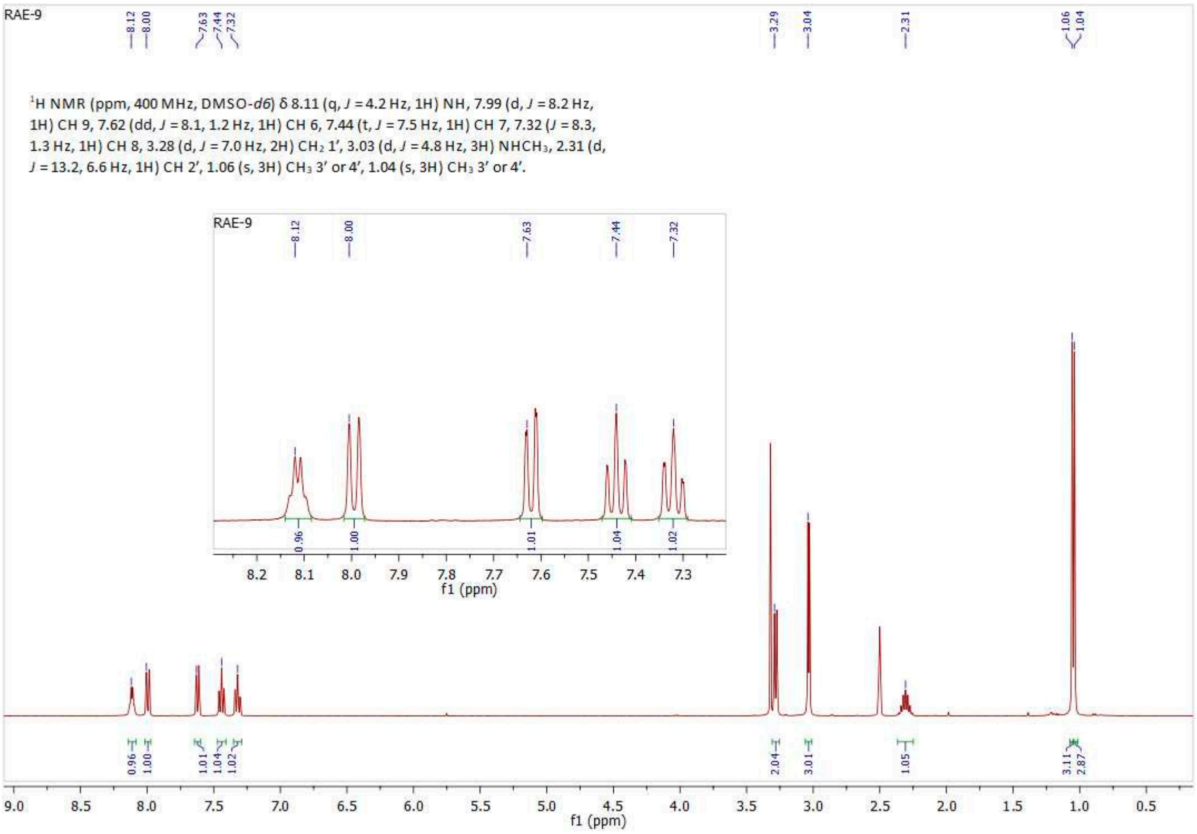
RAE-9
^{1}H NMR (ppm, 400 MHz, DMSO-*d6*) δ 8.11 (q, *J* = 4.2 Hz, 1H) NH, 7.99 (d, *J* = 8.2 Hz, 1H) CH 9, 7.62 (dd, *J* = 8.1, 1.2 Hz, 1H) CH 6, 7.44 (t, *J* = 7.5 Hz, 1H) CH 7, 7.32 (*J* = 8.3, 1.3 Hz, 1H) CH 8, 3.28 (d, *J* = 7.0 Hz, 2H) CH_2 1′, 3.03 (d, *J* = 4.8 Hz, 3H) $NHCH_3$, 2.31 (d, *J* = 13.2, 6.6 Hz, 1H) CH 2′, 1.06 (s, 3H) CH_3 3′ or 4′, 1.04 (s, 3H) CH_3 3′ or 4′.
f1 (ppm)

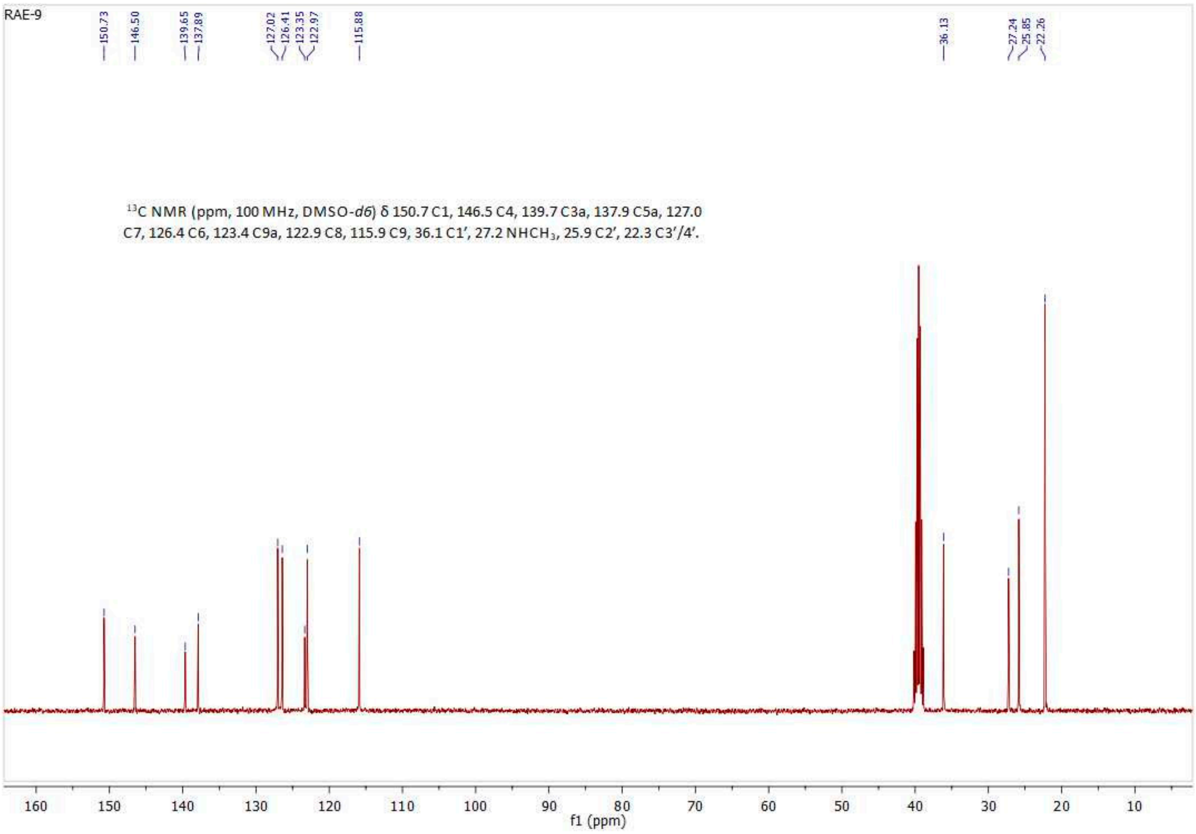
RAE-9
^{13}C NMR (ppm, 100 MHz, DMSO-*d6*) δ 150.7 C1, 146.5 C4, 139.7 C3a, 137.9 C5a, 127.0 C7, 126.4 C6, 123.4 C9a, 122.9 C8, 115.9 C9, 36.1 C1′, 27.2 $NHCH_3$, 25.9 C2′, 22.3 C3′/4′.
f1 (ppm)

S33. *N,1-dimethyl-[1,2,4]triazolo[4,3-a]quinoxalin-4-amine* (**16i**).

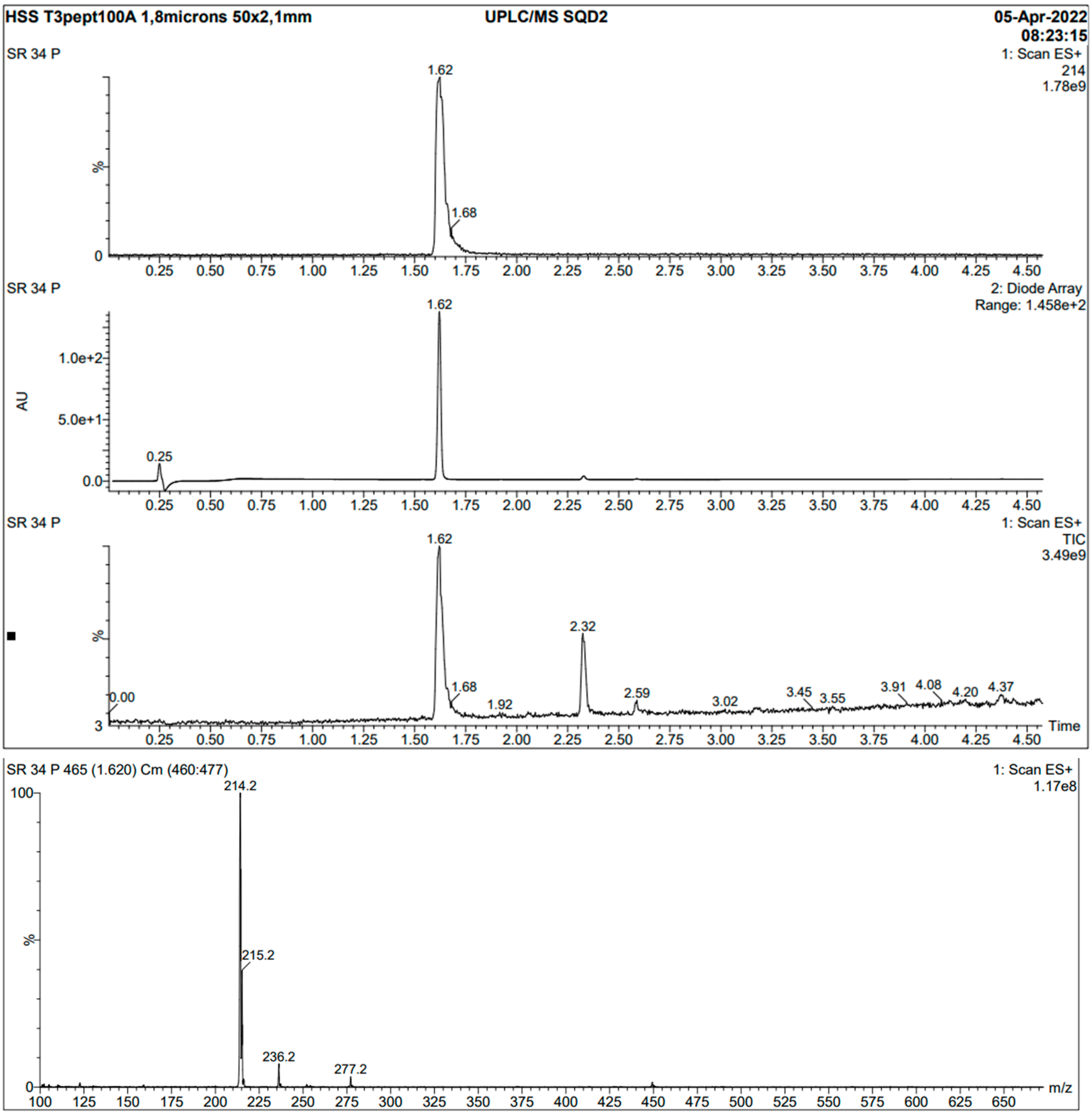

Acquisition Parameter

Source Type ESI Ion Polarity Positive Scan Begin 50 m/z Scan End 2200 m/z

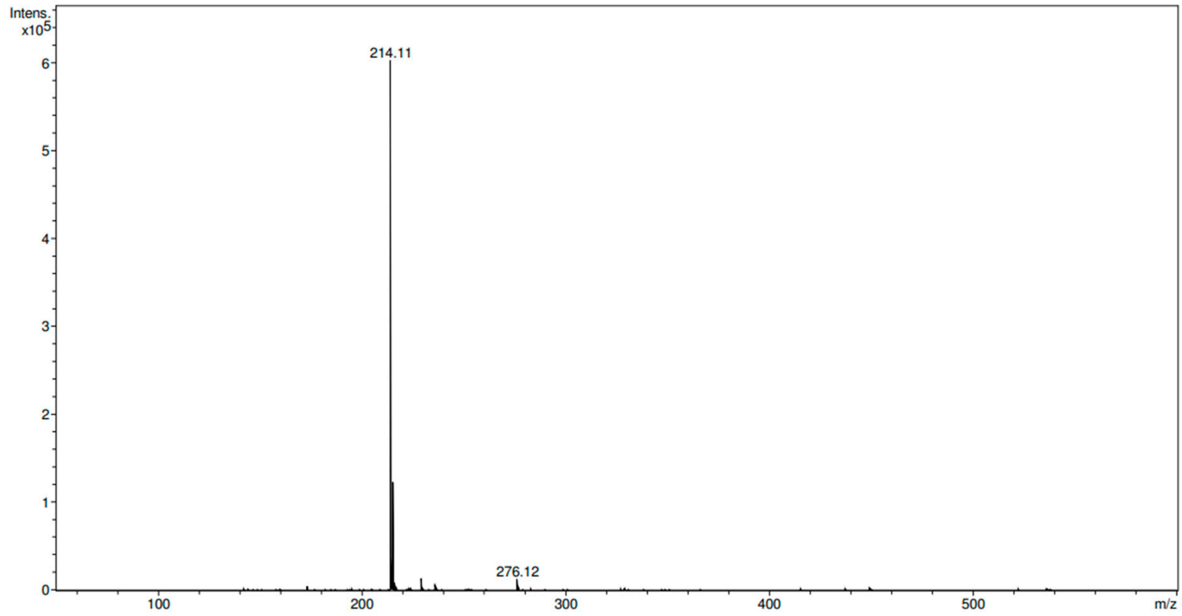

Acquisition Parameter

Source Type ESI Ion Polarity Positive Scan Begin 50 m/z Scan End 2200 m/z

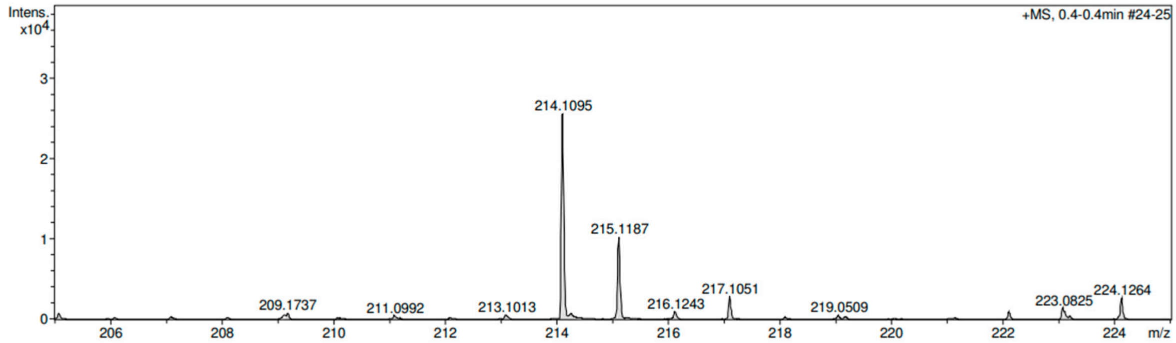

Meas. m/z	#	Ion Formula	m/z	err [ppm]	mSigma	# Sigma	Score	rdb	e⁻ Conf	N-Rule
214.1095	1	C11H12N5	214.1087	3.8	153.8	1	100.00	8.5	even	ok

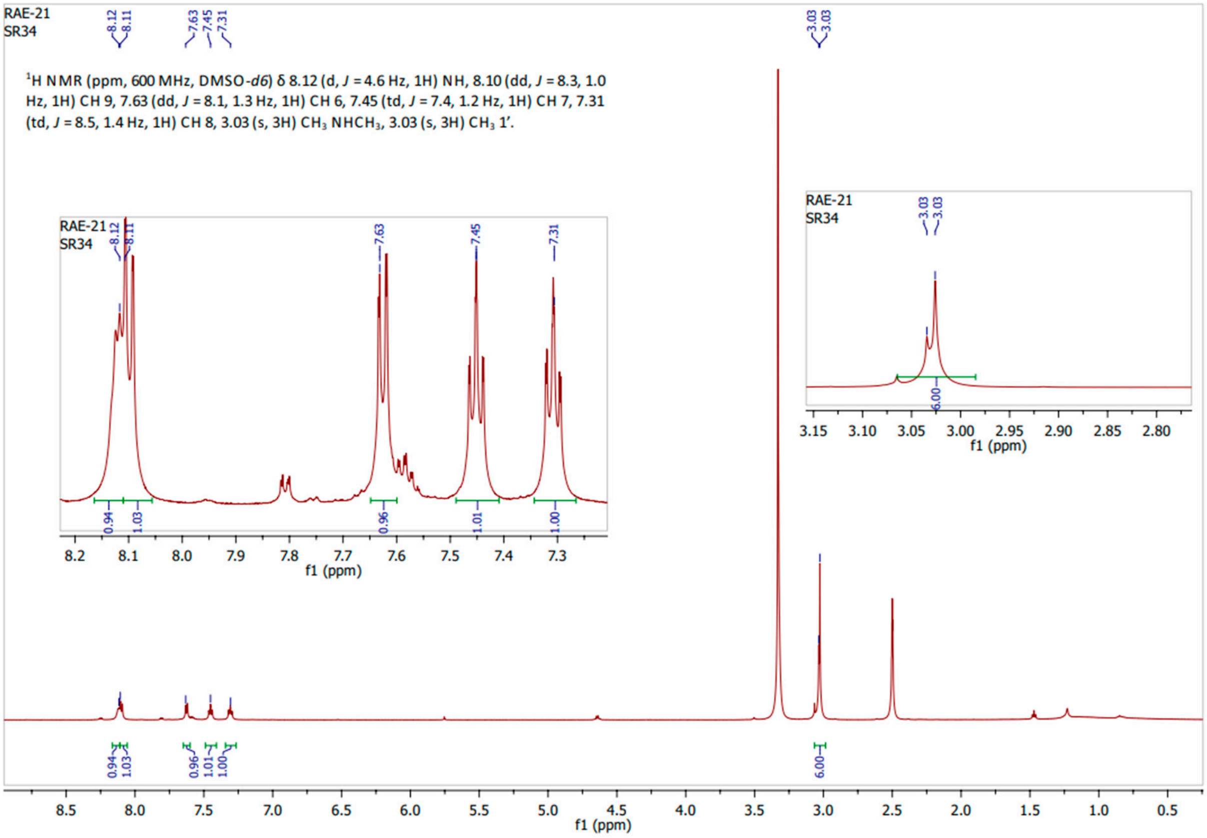
RAE-21
SR34
^{1}H NMR (ppm, 600 MHz, DMSO-d6) δ 8.12 (d, J = 4.6 Hz, 1H) NH, 8.10 (dd, J = 8.3, 1.0 Hz, 1H) CH 9, 7.63 (dd, J = 8.1, 1.3 Hz, 1H) CH 6, 7.45 (td, J = 7.4, 1.2 Hz, 1H) CH 7, 7.31 (td, J = 8.5, 1.4 Hz, 1H) CH 8, 3.03 (s, 3H) CH3 NHCH3, 3.03 (s, 3H) CH3 1'.
f1 (ppm)

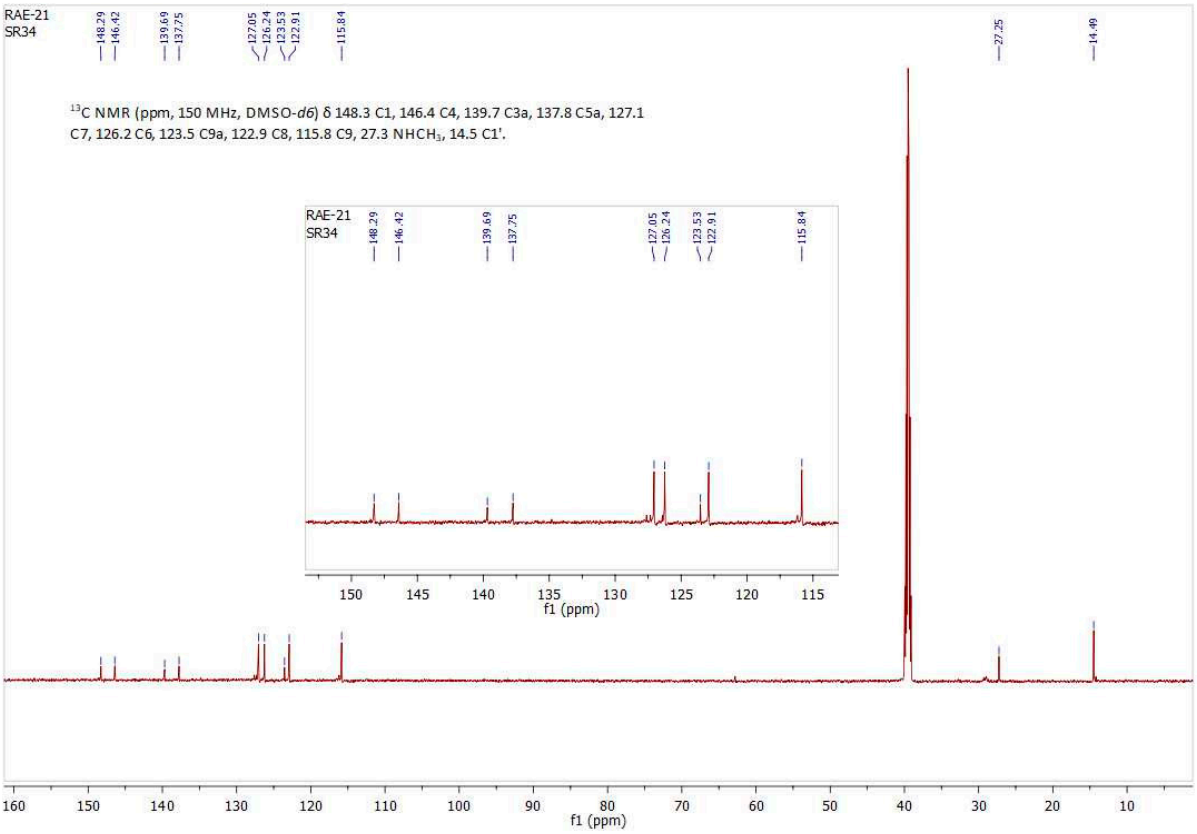
RAE-21
SR34
^{13}C NMR (ppm, 150 MHz, DMSO-d6) δ 148.3 C1, 146.4 C4, 139.7 C3a, 137.8 C5a, 127.1 C7, 126.2 C6, 123.5 C9a, 122.9 C8, 115.8 C9, 27.3 NHCH3, 14.5 C1'.
f1 (ppm)

*S34. 1-(3,4-dimethoxyphenyl)-[1,2,4]triazolo[4,3-a]quinoxalin-4-amine (**16j**).*

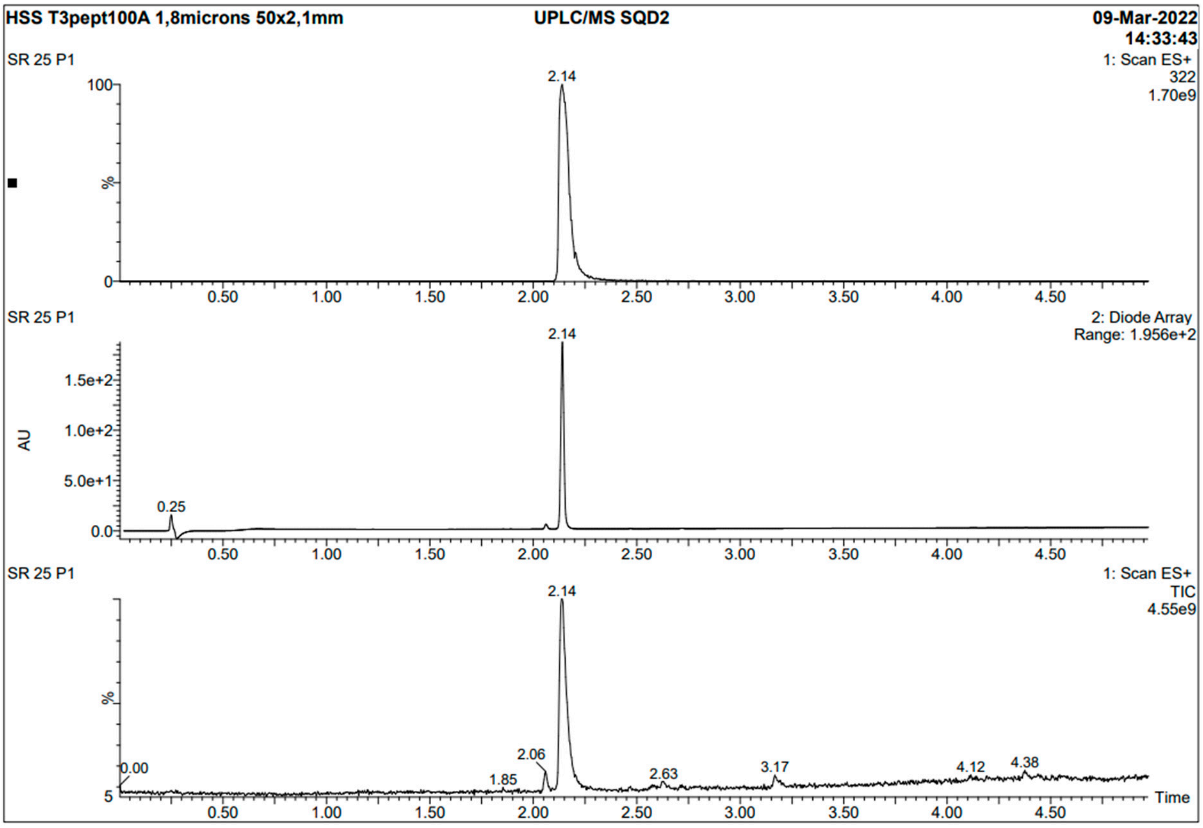

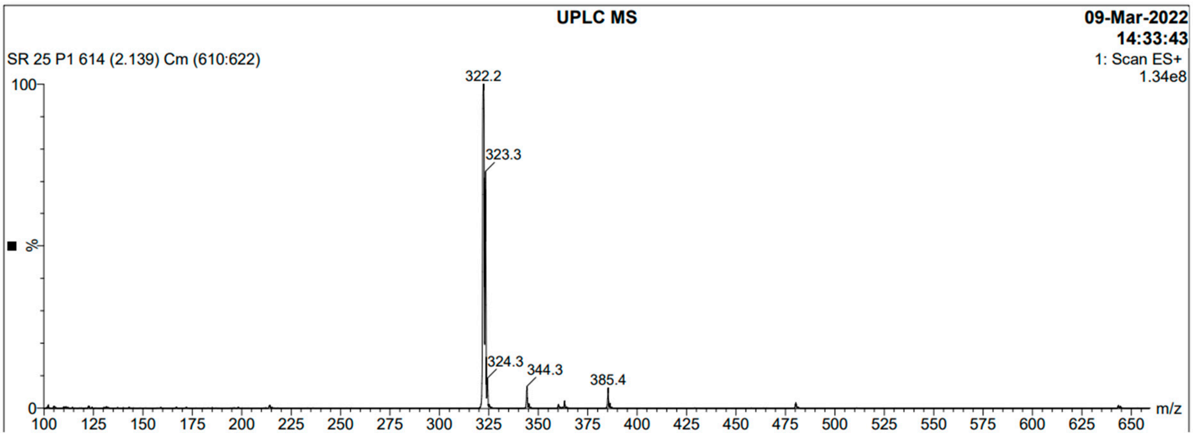

Acquisition Parameter

Source Type	ESI	Ion Polarity	Positive	Scan Begin	50 m/z	Scan End	2200 m/z

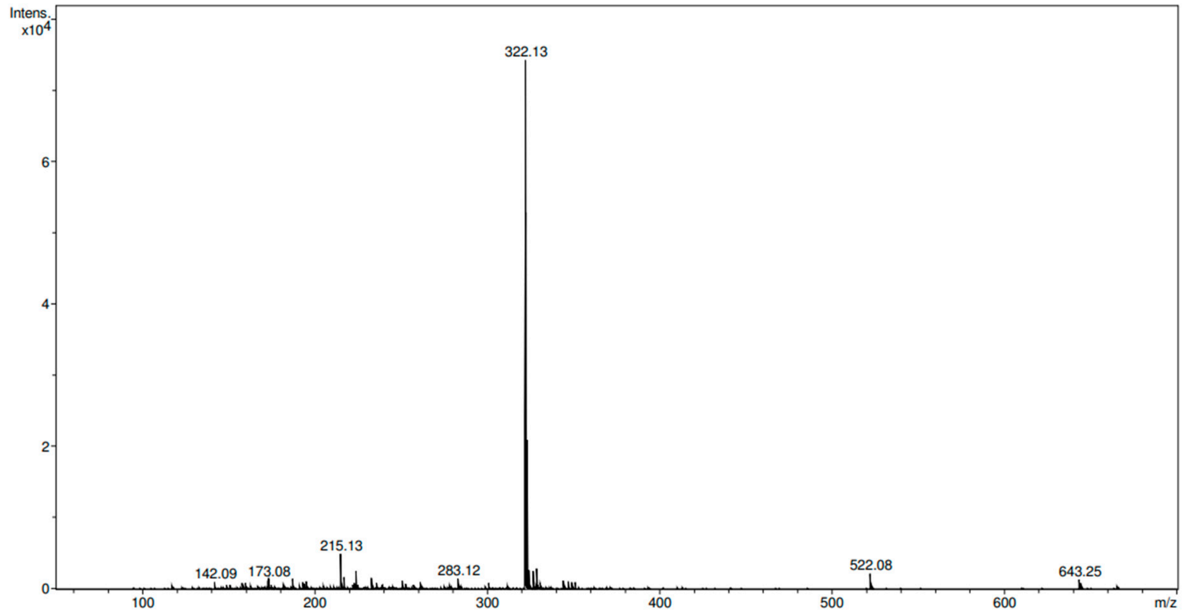

Acquisition Parameter

Source Type	ESI	Ion Polarity	Positive	Scan Begin	50 m/z	Scan End	2200 m/z

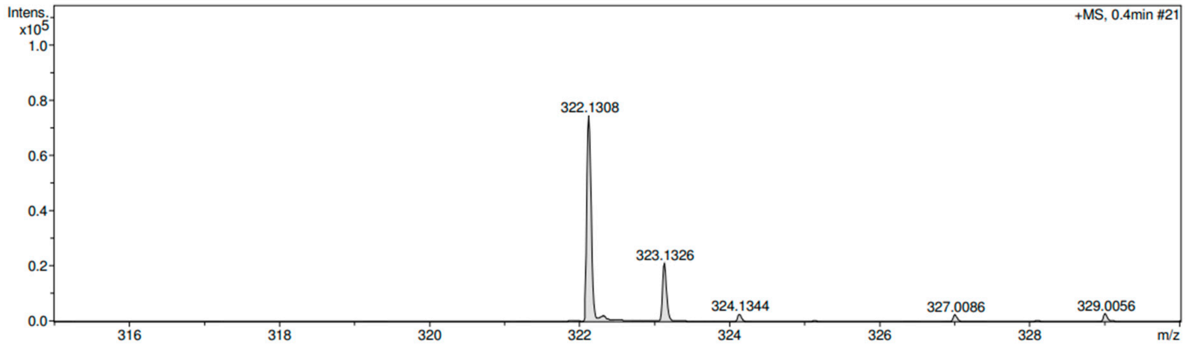

Meas. m/z	#	Ion Formula	m/z	err [ppm]	mSigma	# Sigma	Score	rdb	e⁻ Conf	N-Rule
322.1308	1	C17H16N5O2	322.1299	-3.0	45.8	2	100.00	12.5	even	ok
	2	C16H20NO6	322.1285	7.2	59.0	3	26.15	7.5	even	ok

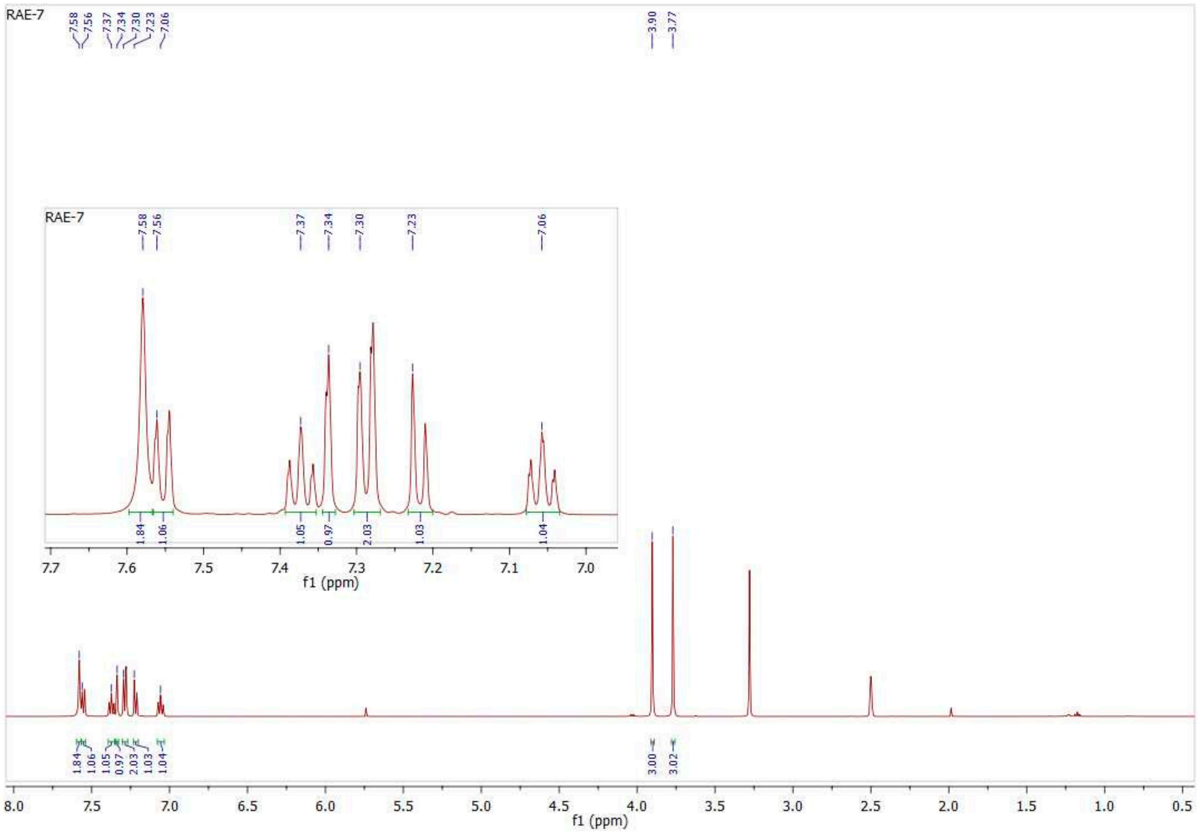
RAE-7
7.58
7.56
7.37
7.34
7.30
7.23
7.06
3.90
3.77
1.84
1.06
1.05
0.97
2.03
1.03
1.04
3.00
3.02
f1 (ppm)

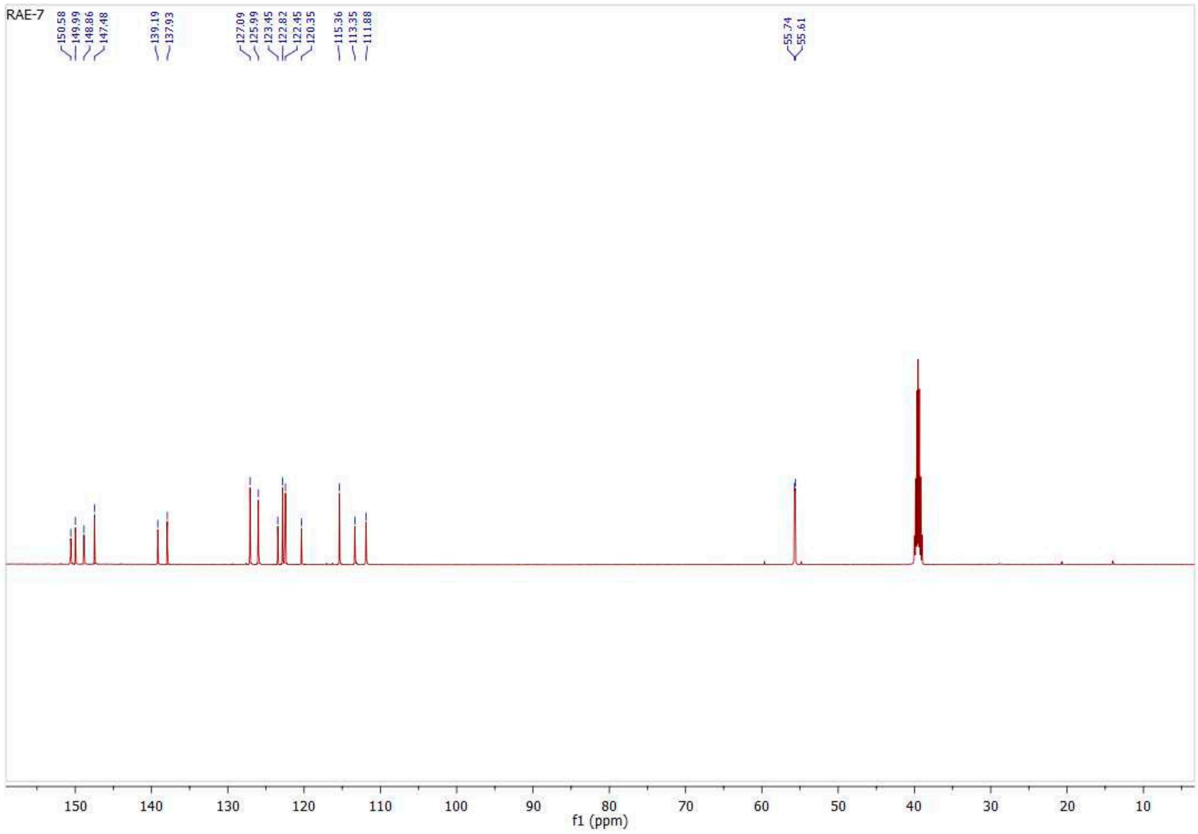
RAE-7
150.58
149.99
148.86
147.48
139.19
137.93
127.09
125.99
123.45
122.82
122.45
120.35
115.36
113.35
111.88
55.74
55.61
f1 (ppm)

S35. 4-(4-(methylamino)-[1,2,4]triazolo[4,3-a]quinoxalin-1-yl)benzene-1,2-diol (**17a**).

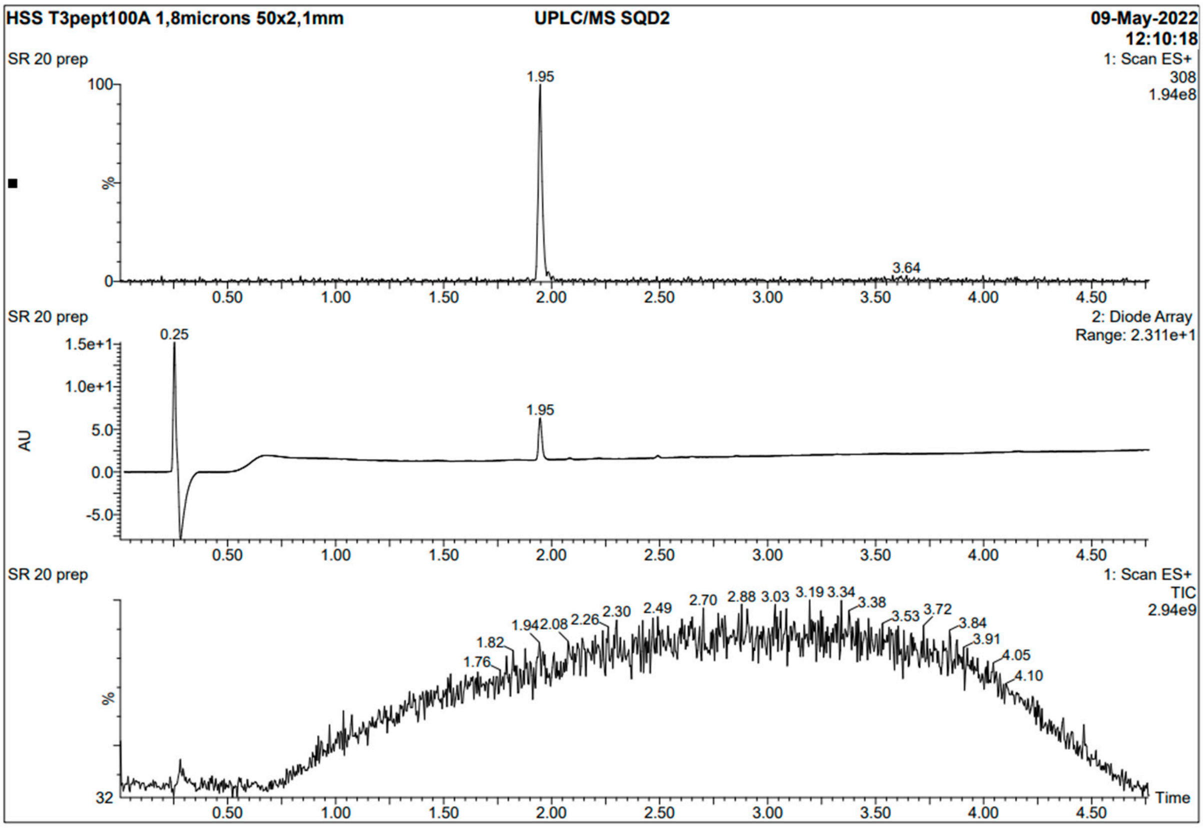

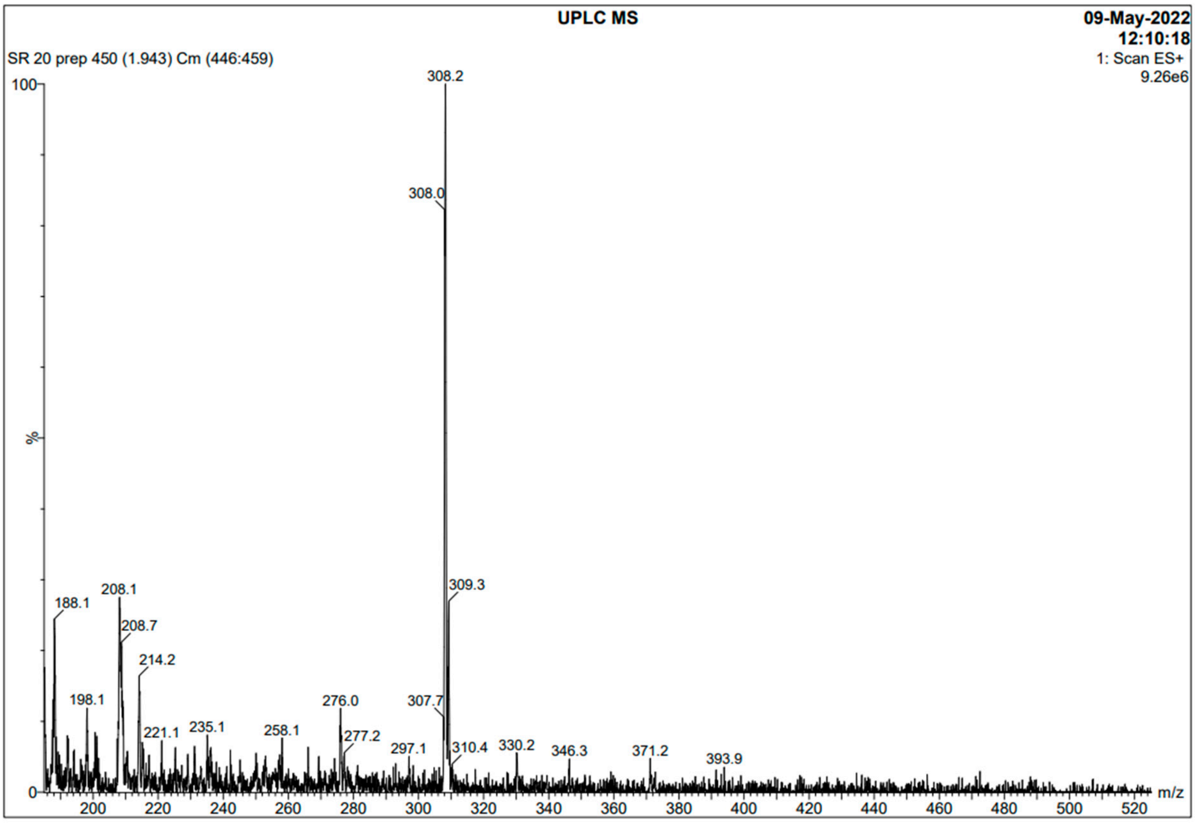

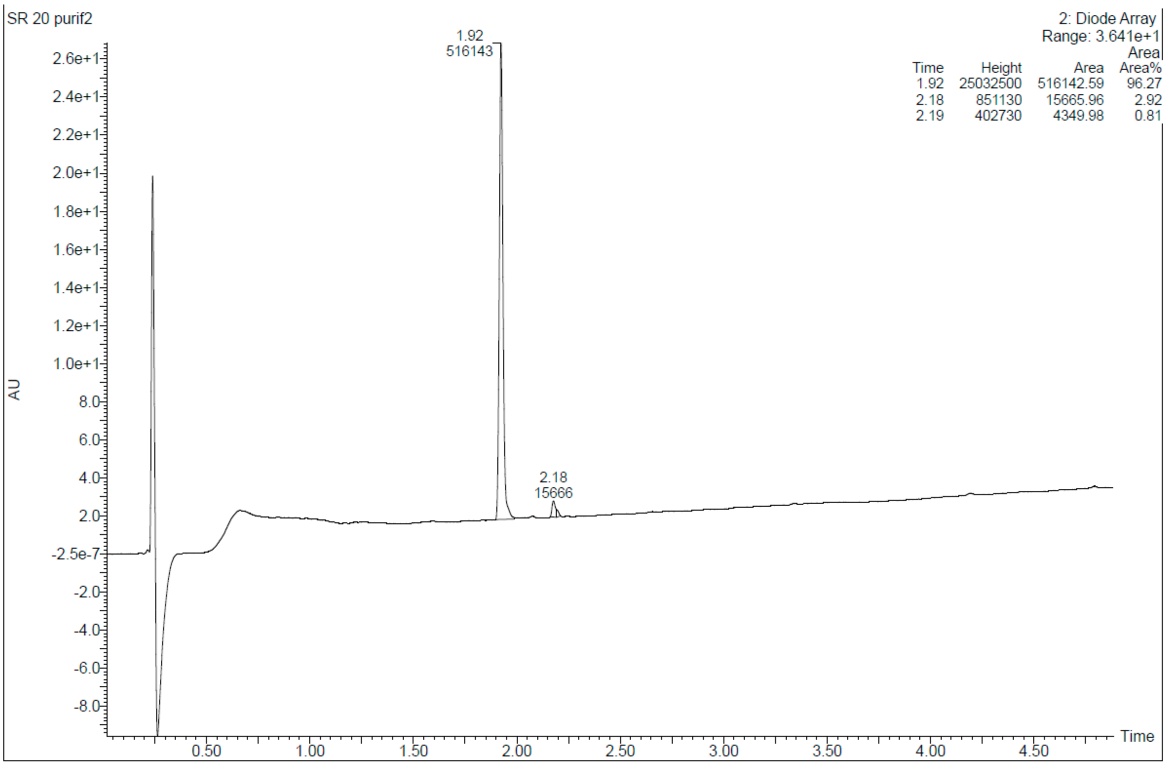

Acquisition Parameter

Source Type	ESI	Ion Polarity	Positive	Scan Begin	50 m/z	Scan End	2200 m/z

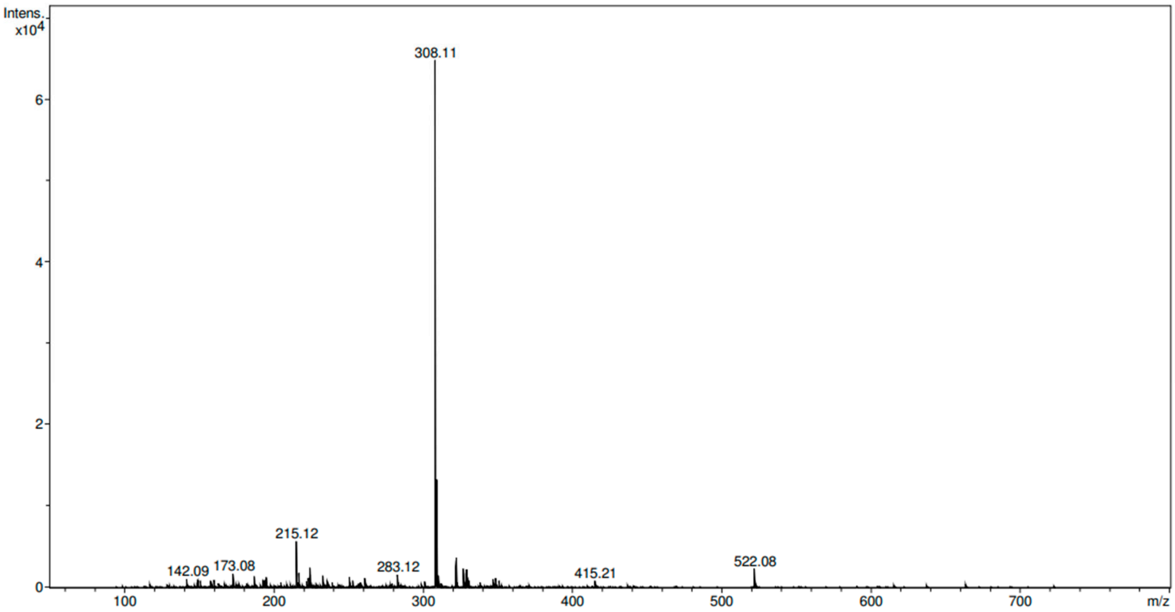

Acquisition Parameter

Source Type	ESI	Ion Polarity	Positive	Scan Begin	50 m/z	Scan End	2200 m/z

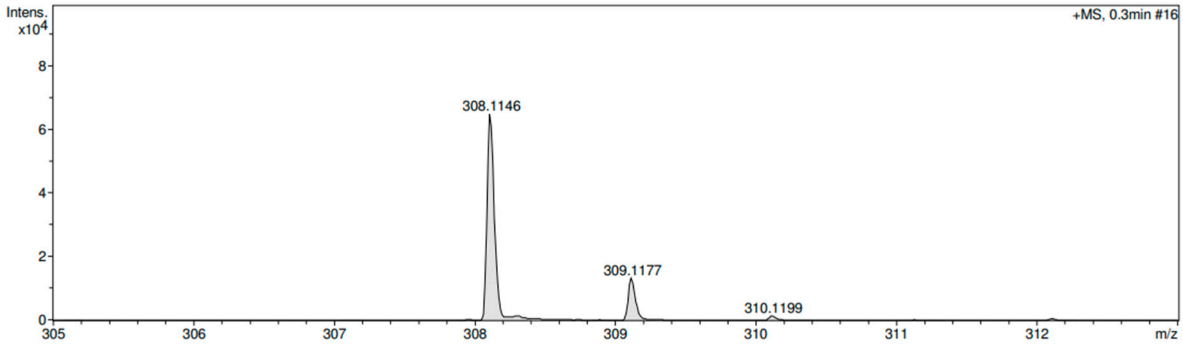

Meas. m/z	#	Ion Formula	m/z	err [ppm]	mSigma	# Sigma	Score	rdb	e⁻ Conf	N-Rule
308.1146	1	C16H14N5O2	308.1142	1.3	6.6	1	100.00	12.5	even	ok

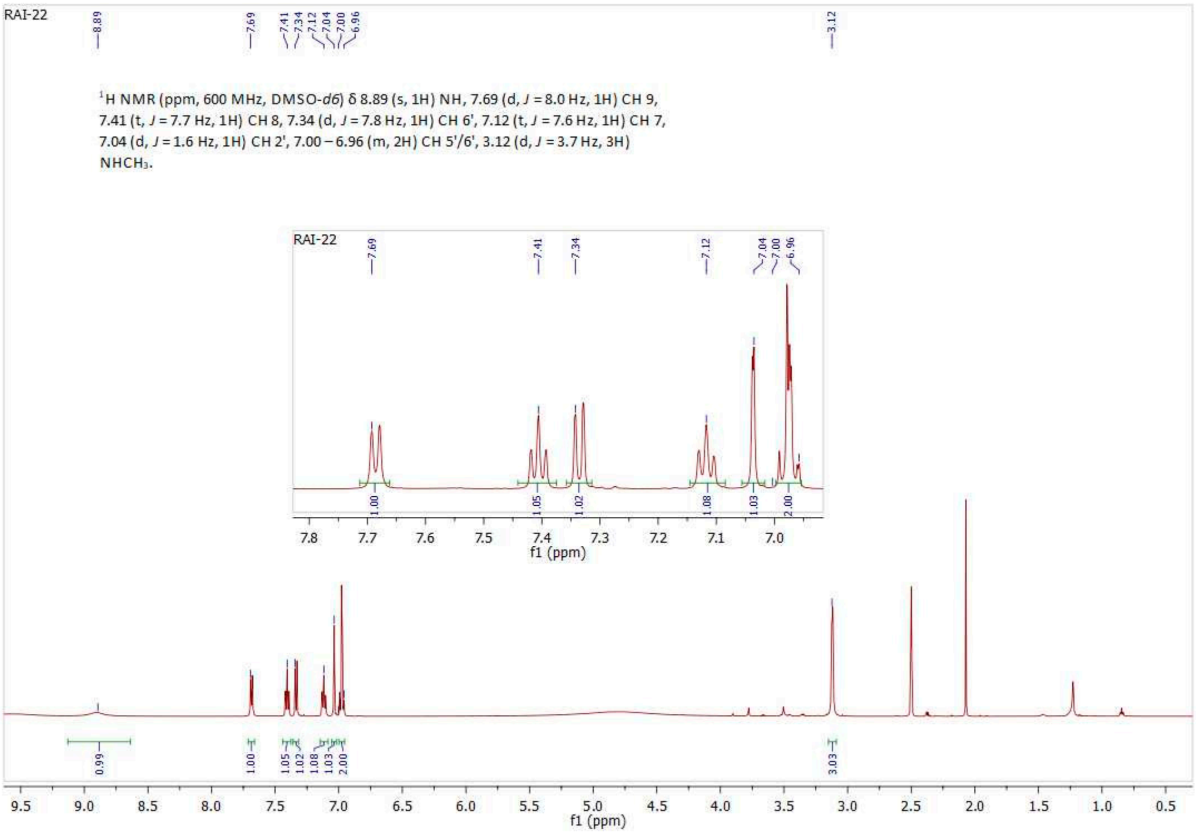

^{1}H NMR (ppm, 600 MHz, DMSO-*d6*) δ 8.89 (s, 1H) NH, 7.69 (d, *J* = 8.0 Hz, 1H) CH 9, 7.41 (t, *J* = 7.7 Hz, 1H) CH 8, 7.34 (d, *J* = 7.8 Hz, 1H) CH 6', 7.12 (t, *J* = 7.6 Hz, 1H) CH 7, 7.04 (d, *J* = 1.6 Hz, 1H) CH 2', 7.00 – 6.96 (m, 2H) CH 5'/6', 3.12 (d, *J* = 3.7 Hz, 3H) $NHCH_3$.

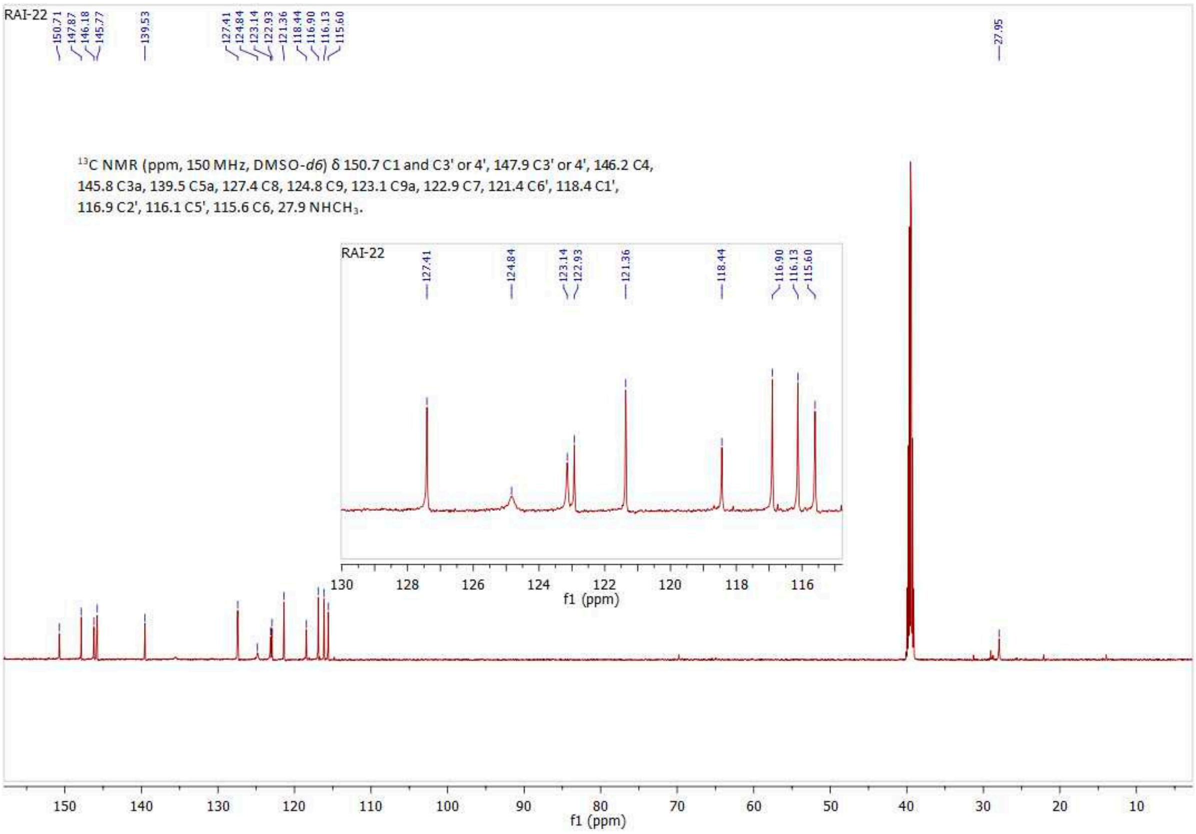
RAI-22
150.71
147.87
146.18
145.77
139.53
127.41
124.84
123.14
122.93
121.36
118.44
116.90
116.13
115.60
27.95
13C NMR (ppm, 150 MHz, DMSO-d6) δ 150.7 C1 and C3' or 4', 147.9 C3' or 4', 146.2 C4,
145.8 C3a, 139.5 C5a, 127.4 C8, 124.8 C9, 123.1 C9a, 122.9 C7, 121.4 C6', 118.4 C1',
116.9 C2', 116.1 C5', 115.6 C6, 27.9 NHCH3.
RAI-22
f1 (ppm)
f1 (ppm)
